# Mega2: validated data-reformatting for linkage and association analyses

**DOI:** 10.1186/s13029-014-0026-y

**Published:** 2014-12-05

**Authors:** Robert V Baron, Charles Kollar, Nandita Mukhopadhyay, Daniel E Weeks

**Affiliations:** Department of Human Genetics, Graduate School of Public Health, University of Pittsburgh, Pittsburgh, PA 15261 USA; Department of Oral Biology, School of Dental Medicine, University of Pittsburgh, Pittsburgh, PA 15261 USA; Department of Biostatistics, Graduate School of Public Health, University of Pittsburgh, Pittsburgh, PA 15261 USA

**Keywords:** Software, Linkage, Association, Human Genetics, Data management

## Abstract

**Background:**

In a typical study of the genetics of a complex human disease, many different analysis programs are used, to test for linkage and association. This requires extensive and careful data reformatting, as many of these analysis programs use differing input formats. Writing scripts to facilitate this can be tedious, time-consuming, and error-prone. To address these issues, the open source Mega2 data reformatting program provides validated and tested data conversions from several commonly-used input formats to many output formats.

**Results:**

Mega2, the Manipulation Environment for Genetic Analysis, facilitates the creation of analysis-ready datasets from data gathered as part of a genetic study. It transparently allows users to process genetic data for family-based or case/control studies accurately and efficiently. In addition to data validation checks, Mega2 provides analysis setup capabilities for a broad choice of commonly-used genetic analysis programs. First released in 2000, Mega2 has recently been significantly improved in a number of ways. We have rewritten it in C++ and have reduced its memory requirements. Mega2 now can read input files in LINKAGE, PLINK, and VCF/BCF formats, as well as its own specialized annotated format. It supports conversion to many commonly-used formats including SOLAR, PLINK, Merlin, Mendel, SimWalk2, Cranefoot, IQLS, FBAT, MORGAN, BEAGLE, Eigenstrat, Structure, and PLINK/SEQ. When controlled by a batch file, Mega2 can be used non-interactively in data reformatting pipelines. Support for genetic data from several other species besides humans has been added.

**Conclusions:**

By providing tested and validated data reformatting, Mega2 facilitates more accurate and extensive analyses of genetic data, avoiding the need to write, debug, and maintain one’s own custom data reformatting scripts.

Mega2 is freely available at https://watson.hgen.pitt.edu/register/.

**Electronic supplementary material:**

The online version of this article (doi:10.1186/s13029-014-0026-y) contains supplementary material, which is available to authorized users.

## Background

The gene-discovery process is very well advanced at the data-generation end with sophisticated database management systems, laboratory information management systems, and bioinformatics tools. There has also been enormous progress in terms of analytical software. However, very little has been done to facilitate the efficient transfer of data from the generation stage to the analysis stage; analysis programs have diverse and stringent requirements (not always clearly documented) on how the input data should be formatted, which is often very different from how the generated data are formatted. Researchers face the need to collect and collate genetic data from diverse sources, and this need has increased significantly as rapidly improving technology generates orders of magnitude more data. As new analysis programs come into being, data setup and organization continues to be an error-prone and very time-consuming task if performed manually, but ideal for well-tested computer automation.

In the course of a single study of the genetics of a complex disease, the optimal analysis might require use of several different programs. For example, one might want to use pedstats [1] to check for data validity, PREST [2,3] to check for relationship errors, SOLAR [4,5] to test for linkage, and Mendel [6-8] to test for association in the presence of linkage. Each provides the best possible analysis but also has its own strict input format requirements, so there is great value in being able to quickly and easily convert one’s data format as required.

To meet these needs, we developed Mega2, the Manipulation Environment for Genetic Analysis [9,10], which automates common data reformatting tasks, thereby accelerating analyses, saving time, and reducing errors. We describe here recent major updates to Mega2, which include improvements in memory efficiency, improved support for commonly used input formats such as PLINK and VCF, and addition of several more target output formats.

### Implementation

Mega2 was originally released in January 2000, and has undergone continuous revisions since. Mega2 was originally written in C, but now has been written in C++, allowing us to now use modern object-oriented programming techniques. Mega2 was designed to be used in a Unix environment, and so for extended functionality, such as plotting results with R or running generated scripts, uses a few other programs commonly available in the Unix environment, such as Perl, Awk, Python, tcsh and bash-shells, and R. Perl is used for producing formatted output such as tables and HTML reports, and R is used to create graphical output using our R “nplplot” package. The currently released version (4.7.1) of Mega2 is available in Additional file 1; for updated versions, please visit the project home page as listed in the “Availability and requirements” section.

**Figure 1:**
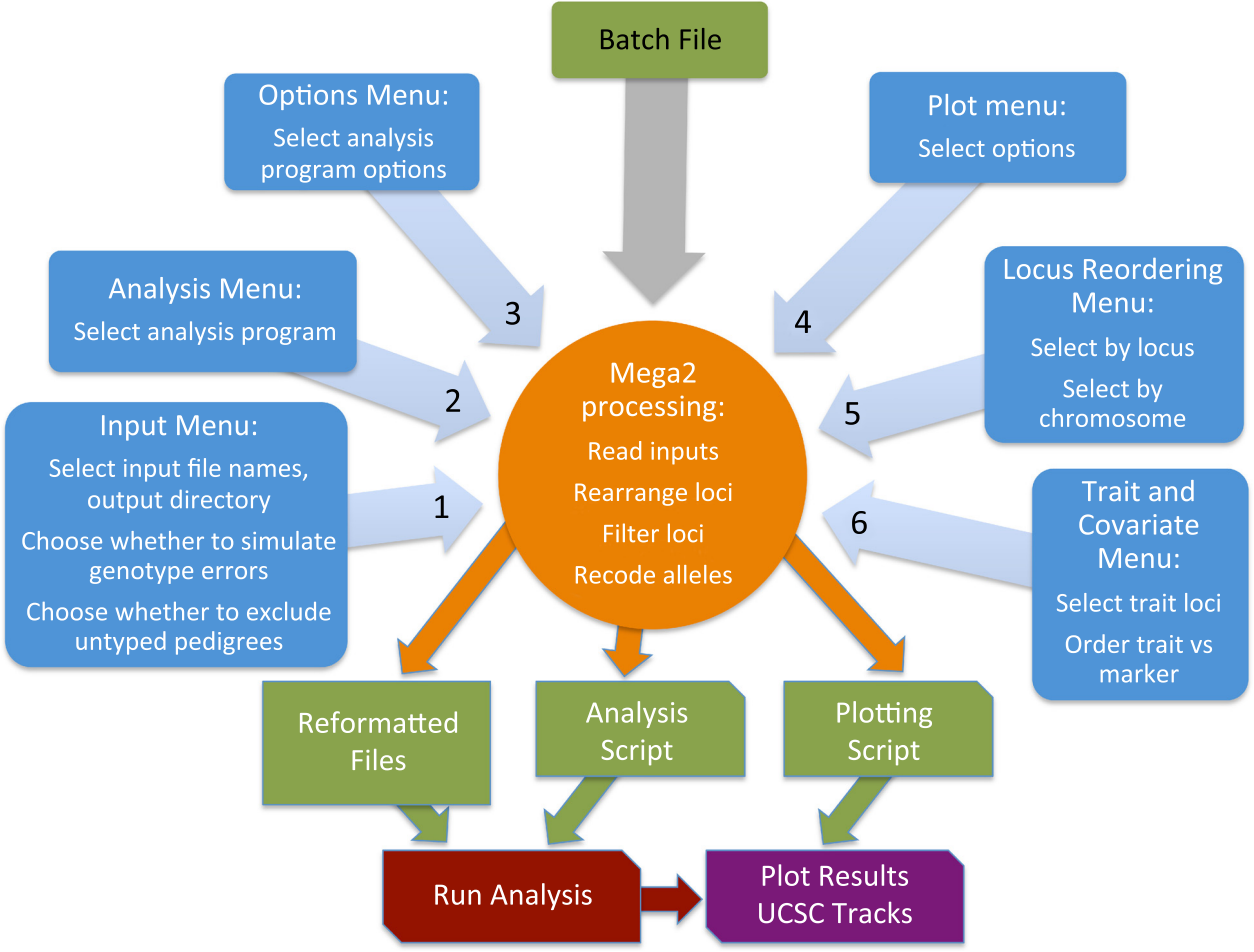
**After querying the user via a variety of menus (blue blocks), Mega2 then processes the data (orange circle), creating reformatted files and auxiliary analysis and plotting scripts (green blocks).** Mega2 can also be run in non-interactive mode using a batch file (top center).

The current Mega2 implementation transforms a matched set of pedigree, phenotype, genotype, and map (genetic and physical) information into a matched set of output files that are ready for analysis by one of many commonly-used genetic analysis tools. Accordingly, Mega2 is organized into input, error-checking, reordering, and output components, constituting a single-layered architecture, and communicating directly with each other as necessary. Mega2 is a command-line program that is typically run without arguments in an interactive mode, where a sequence of menus (Figure 1, blue blocks) are presented to the user to specify 1) input files and filters, 2) the target analysis program, 3) program-specific options, 4) plot customization options, 5) the subset of loci to be included in the output data, and 6) the trait loci and covariates. The input data can be in LINKAGE format [11-13], Mega2 annotated format, PLINK (ped or binary) format [14], or VCF/BCF format [15]. When reading from Mega2 annotated format, input files include a) a pedigree file containing sample-related pedigree, phenotype, and genotype information, b) a locus names file, and c) a map file containing chromosomal positions for marker loci. Additionally, the user may specify d) an omit file for setting selected genotypes to unknown, e) an allele-frequency file, and f) a disease-model or penetrance file. Mega2 reads (Figure 1, orange circle) and validates the input data, and creates output files to be run by the target program, possibly partitioned by chromosome and/or traits. Output usually includes (Figure 1, green blocks) scripts to run the analysis on the re-organized, re-formatted data, and may create plots of the analysis results, as well as BED custom tracks suitable for plotting in the UCSC Genome Browser, using our ‘nplplot’ R library. Detailed diagnostic measures are created in certain cases. Full logs of run-related information including error messages are generated. Mega2 can also be run in a non-interactive mode using a batch file (Figure 1, top center) containing (key, value) pairs corresponding to the choices made via the interactive menus.

## Results

Mega2 was originally written without much attention to memory efficiency, as at that time a genome-wide scan consisted of only several hundred markers. Thus, Mega2’s memory usage was initially on the order of people x allele x 8 bytes, as each person/allele combination was assigned a pointer to the allele label. For two-allele marker data, we have markedly reduced memory requirements by replacing each pair of 8 byte pointers with a 2 bit index specifying which alleles the individual has. We also allow the user to switch out of the 2 bit mode if they want to work with more highly polymorphic markers. Further memory efficiencies have been gained by not storing (unknown) genotypes for completely untyped individuals, but who are still needed to specify the pedigree structure. As a result of these improvements, Mega2 now can handle genome-wide scale data – for example, 895K two-allele markers on 3.1K people requires only 1.12 Gb of memory for Mega2 processing.

Mega2 can now read data in from a wider variety of input formats. Many researchers now have their data in PLINK-format [14], so we have extended Mega2 to support reading PLINK input files. Mega2 now directly processes PLINK ‘ped’ and binary input formats. Mega2 also supports PLINK phenotype files, as well as Mega2-format map files that specify a sex-specific genetic map. Furthermore, we recently added support for reading Variant Call Format (VCF) files and their binary compressed equivalent, BCF; most sequencing-based data are now in VCF/BCF format [15].

As can be seen in Table 1, Mega2 currently reformats data for a wide variety of target programs that perform linkage and association analysis, a popular pedigree-drawing program Cranefoot [16], as well as others that perform quality-control analyses such as computing genotyping success rates, testing for departures from Hardy-Weinberg equilibrium, etc. Mega2 is now able to generate PLINK binary output files. As data sets get larger, Mega2’s support of the PLINK binary format, both as input and output, is important because it is a common way of compactly storing large scale data and provides a succinct way to efficiently get large scale data into and out of Mega2. Recently, seven new output formats have been added (see Table 1): (1) IQLS/Idcoefs [17-19] - a program for carrying out haplotype-based association tests while properly accounting for relatedness; (2) FBAT [20] – a program for carrying out family-based association tests; (3) Morgan [21] – a package capable of many analyses, with particular strengths in the area of Monte Carlo Markov Chain analyses of family data; (4) Beagle [22] – a package capable of many analyses, including haplotyping and association testing; (5) Eigenstrat [23,24] – a program for inferring and adjusting for population substructure from genome-wide marker data while testing for association; (6) Structure [25,26] – a program for investigating population structure and admixture using genome-wide marker data; and (7) PLINK/SEQ [27] – a package for analysis of data from large-scale sequencing projects.

**Table 1:** **Mega2 currently supports 37 output targets seven new ones have been added since 2011**

SimWalk2 format [28]	SOLAR format [4,5]	Mendel format [7]
Vintage MENDEL format [8]	Vitesse format [29]	SUP format [30,31]
ASPEX format	Linkage format [11-13] Pre-makeped format [11-13]	Cranefoot format [16]
GeneHunter-Plus format [32]	Testing loci for HWE	Mega2 annotated format
GeneHunter format [33,34]	Allegro format [35]	IQLS/Idcoefs format [17,36] (*added 6*/*11*)
Conversion to nuclear families	MLBQTL format [37]	PLINK format [14] (*binary added 1*/*13*)
SLINK format [30,31,38]	SAGE format [39]	FBAT format [20] (*added 1*/*13*)
SPLINK format [40]	Merlin/SimWalk2-NPL format	Morgan format [21] (*added 6*/*13*)
Homogeneity analyses	PREST format [2,3]	Beagle format [22,41-43] (*added 6*/*13*)
SIMULATE format [44]	PAP format [45,46]	Eigenstrat format [23,24] (*added 6*/*13*)
Genotype/phenotype/segregation summaries	Merlin format [47]	Structure format [25,26,48] (*added 6*/*13*)
Old SAGE format	Loki format [49]	PLINK/SEQ format [27] (*added 10*/*13*)

The Mega2 distribution package has been updated to provide greater ease of installation and compatibility with many Unix environments. It contains added support for migration of legacy input data to our updated formats.

## Discussion

In applied data analysis, a thorough analysis often requires the use of multiple different programs, many of which have their own precise input format requirements. Reformatting programs such as Mega2 can markedly accelerate analyses by providing accurate, quick, and error-free conversion routines. This need has been recognized in the area of population genetics, where several reformatting programs have been written [50-52], including one that converts to 52 different formats [51]. In the area of human genetics, limited reformatting options have been made available as part of larger database systems. For example, the GeneLink database system [53] initially exported into LINKAGE [11-13,54], GAS [55], or RelCheck [56,57] formats, while the Integrated Genotyping System [58] exported into several formats, including Merlin [47], GeneHunter [33,34], QTDT [59], and Transmit [60] formats. However, these database systems can be difficult to install and maintain. Other more stand-alone approaches to reformatting in this area include SIB-PAIR [61], by David Duffy, which is a command-line oriented program that can create locus and pedigree files in a variety of formats, such as FISHER [8], GAS [55], Genehunter [33,34], LINKAGE [11-13,54], LOKI [49], MENDEL [6], MERLIN [47], PAP [45,46] and SAGE [39]. SIB-PAIR appears to require very detailed line-by-line commands that would make it harder to use than Mega2 for most users. Another program is fcGENE [62] (available from SourceForge), which is focused on converting PLINK-format data for imputation (MaCH [63], IMPUTE [64], BEAGLE [22,41-43], BIMBAM [65]), and then converting the resulting imputed data into the following formats: PLINK [14], SNPTEST [64], HAPLOVIEW [66], EIGENSOFT [23,24], GenABEL [67], and VCF [15]. While fcGENE is fast and easy to use, it is currently limited (e.g., it does not accept VCF or LINKAGE format as input, it only supports a single dichotomous phenotype, it does not support selection by chromosome, etc.).

ALOHOMORA [68] provides an elegant interface for carrying out linkage analyses of Affymetrix 10K single nucleotide polymorphism (SNP) genotype data. This program actually uses Mega2 as its internal reformatting engine for some of its options.

PLINK [14] is an association analysis toolset that has a variety of data management and filtering options for handling large-scale SNP data. The main focus of PLINK is population-based unrelated samples, with some support for family-based association testing. PLINK only exports data in a few limited formats. We have used PLINK on our family data to carry out data cleaning, but then still needed Mega2 to reformat the data in order to carry out analyses using other external programs. In our experience, it is difficult to use PLINK on family data while maintaining the original pedigree structures upon output, as PLINK favors automatically filtering out individuals with low genotyping success rates (such as untyped founders).

From this brief survey of currently available data reformatting software, two things are immediately apparent: many researchers have recognized the need for providing one’s data in many different formats; and Mega2, which is free, open source, and available on Unix, Windows, and Macintosh platforms, is well-positioned to continue to fill this need.

## Conclusion

When carrying out quality control and statistical analyses for a genetic study of a human disease, one quickly discovers that data organization and analysis set-up is a critical, time-consuming, and extremely tedious task. Furthermore, one often needs to use several different analysis programs, each with its own idiosyncratic input format requirements. To meet these needs, we developed Mega2, taking the time to carefully understand the precise (sometimes poorly documented) requirements of each target format, implementing our data reformatting pipeline in tested and well-documented code. Mega2’s tested and validated data conversion options expands the universe of possible analyses for the average researcher by removing the hurdle of having to tediously write, check, debug, and maintain their own conversion scripts.

### Availability and requirements

**Project name:** Mega2

**Project home page:**https://watson.hgen.pitt.edu/register/

**Operating systems:** Linux, Macintosh OS X, Windows, Solaris

**Programming language:** C++

**Other requirements:** R, Perl, Python, awk, bash, and csh

**License:** GNU GPL v3

**Any restrictions to use by non-academics:** None.

## Additional file

Additional file 1:
**A zipped archive containing the Mega2 version 4.7.1 distribution package; both source and binary executables are included.**
