# Supplementary material for: Mega2: validated data-reformatting for linkage and association analyses
Source: Source Code Biol Med. 2014 Dec 5;9:26. doi: 10.1186/s13029-014-0026-y (PMC4269913; doi:10.1186/s13029-014-0026-y)
Supplement: Additional file 1: — A zipped archive containing the Mega2 version 4.7.1 distribution package; both source and binary executables are included. [file 13029_2014_26_MOESM1_ESM.zip › mega2_v4.7.1_src/example_output_annotated/MEGA2.KEYS.html]

 


 MEGA2.KEYS 


```
-----------------------------------------------------
        Mega2 version 4.7.0
Run date:                  2014-7-22-13-08
This file created on       Tue Jul 22 13:08:32 2014
Input file names
#       Pedigree file:               pedin.annotated
#          Locus file:               names.annotated
#            Map file:               map.annotated
#      Frequency file:               frequency.annotated
#     Penetrance file:               penetrance.annotated
  Untyped pedigree option: Include all pedigrees whether typed or not
Mendelianly-inconsistent genotypes included in output.
Half-typed individuals' genotypes included in output.
---------------------------------------------

INPUT                               OUTPUT   
Numeric     Unique           
NPed  NPer  Ped   ID:      Loop id  Pedigree  Person 
1     1     1     1_1_1             1         1_1    
1     2     1     1_1_2             1         1_2    
1     3     1     1_1_3             1         1_3    
1     4     1     1_1_4             1         1_4    
1     5     1     1_1_5             1         1_5    
1     6     1     1_1_6             1         1_6    
1     7     1     1_1_7             1         1_7    
1     8     1     1_1_8             1         1_8    
1     9     1     1_1_9             1         1_9    
1     10    1     1_1_10            1         1_10   
1     11    1     1_1_11            1         1_11   
2     1     2     2_2_1             2         2_1    
2     2     2     2_2_2             2         2_2    
2     3     2     2_2_3             2         2_3    
2     4     2     2_2_4             2         2_4    
2     5     2     2_2_5             2         2_5    
2     6     2     2_2_6             2         2_6    
2     7     2     2_2_7             2         2_7    
2     8     2     2_2_8          2  2         2_8    
2     11    2     2_2_8          2  2         2_8    
2     9     2     2_2_9             2         2_9    
2     10    2     2_2_10            2         2_10
```
