# Supplementary material for: Mega2: validated data-reformatting for linkage and association analyses
Source: Source Code Biol Med. 2014 Dec 5;9:26. doi: 10.1186/s13029-014-0026-y (PMC4269913; doi:10.1186/s13029-014-0026-y)
Supplement: Additional file 1: — A zipped archive containing the Mega2 version 4.7.1 distribution package; both source and binary executables are included. [file 13029_2014_26_MOESM1_ESM.zip › mega2_v4.7.1_src/example_output_annotated/MEGA2.LOG.html]

 


 MEGA2.LOG 


```
==========================================================
                          MEGA2 4.7.0
     Copyright (C) 1999-2014 Robert Baron, Charles P. Kollar,
     Nandita Mukhopadhyay, Lee Almasy, Mark Schroeder, William P. Mulvihill,
     Daniel E. Weeks, and University of Pittsburgh

     Last updated: Jul 22 2014, 12:55:24 , valid until June 15, 2015.
     Compiled with gcc version 4.2.1 Compatible Apple LLVM 5.1 (clang-503.0.40)

     Mega2 comes with ABSOLUTELY NO WARRANTY.
     See LICENSE.txt for terms of copying, modifying & redistributing Mega2.
==========================================================
NOTE: If you have previously used explicit numbers for sex chromosomes, BEWARE!
We have changed the numbers to be compatible with PLINK. 23 still codes for X,
but 24 codes for Y and 25 Codes for XY.

Running Mega2 in batch mode from MEGA2.BATCH.annotated
Input filenames and missing value indicator read in from batch file.
Analysis option read in from batch file.
Markers, chromosome(s) and read in from batch file.
Trait selection(s) read in from batch file.
Keyword Input_PLINK_Map_File not in batch file, PLINK map file assumed to be unspecified.
Keyword Input_Omit_File not in batch file, Omit file assumed to be unspecified.
Keyword Input_Aux_File not in batch file, Aux file assumed to be unspecified.
Keyword Input_Phenotype_File not in batch file, Phenotype file assumed to be unspecified.
Keyword Input_Path not in batch file, using default '.' (current directory).
-----------------------------------------------------
        Mega2 version 4.7.0
Run date:                  2014-7-22-13-08
This file created on       Tue Jul 22 13:08:32 2014
Input file names
#       Pedigree file:               pedin.annotated
#          Locus file:               names.annotated
#            Map file:               map.annotated
#      Frequency file:               frequency.annotated
#     Penetrance file:               penetrance.annotated
  Untyped pedigree option: Include all pedigrees whether typed or not
Mendelianly-inconsistent genotypes included in output.
Half-typed individuals' genotypes included in output.
---------------------------------------------

===========================================================
Analysis option: Mendel7+.
Input Format: Mega2 format with header
Pedigree, names and map file specified as Mega2 format.
Input files will be read in as Mega2 format files.
Reading Mega2 format names file: names.annotated
Found 1 possible maps.
Done reading map file: map.annotated

===========================================================
Total number of loci =  5
2 trait loci 
      1 Affection status locus: 
                TRAIT
      1 Quantitative locus: 
                Q1 
      3 Marker loci 
Number of loci found per chromosome (chromosome:number)
   5:3
===========================================================
Reading Mega2 format frequency file: frequency.annotated
Reading Mega2 format penetrance file: penetrance.annotated
Reading Mega2 format pedigree file: pedin.annotated
===========================================================
Input pedigree data contains:
Input pedigree file is in post-makeped format.
                                                Marker Genotypes
                                                Fully    Half
     Pedigrees   People   Males   Females       Typed    Typed     Total
TOTAL        2       22      10        12          66        0        66
Typed        2       22      10        12
Untyped      0        0       0         0
===========================================================
Pedigree exclusion option : Include all pedigrees whether typed or not.
Recoding pedigree genotypes ... 
===========================================================
Pedigree data summary after recoding:
Input pedigree file is in post-makeped format.
                                                Marker Genotypes
                                                Fully    Half
     Pedigrees   People   Males   Females       Typed    Typed     Total
TOTAL        2       22      10        12          66        0        66
Typed        2       22      10        12
Untyped      0        0       0         0
===========================================================
Connecting loops in pedigree 2...
Ped 2: Loop-breaker ids 2_2_8 and 2_2_11
        Saving 2_2_8, deleting 2_2_11
Selected map Map.
Selected chromosome 5
Output will combine markers and the following selected traits:
                TRAIT [MARKERS]
After selecting traits and covariates
1 trait locus 
      1 Affection status locus: 
                TRAIT
===========================================================
Pedigree statistics after selecting chromosomes and marker loci:
                                                Marker Genotypes
                                                Fully    Half
     Pedigrees   People   Males   Females       Typed    Typed     Total
TOTAL        2       21       9        12          63        0        63
Typed        2       21       9        12
Untyped      0        0       0         0
===========================================================
Person id in output pedigree file = Individual id
===========================================================
Pedigree id in output pedigree file = Pedigree number
===========================================================
Mega2 created the following file(s) for Mendel7+:
        Definition file:      mendel_locus.05
        Pedigree file:        mendel_ped.05
        Map file:             mendel_map.05
        Control file:         mendel_control.05
===========================================================
Output is in ../example_output_annotated
===========================================================
If you use Mega2 as part of a published work, please reference 
 Mukhopadhyay N, Almasy L, Schroeder M, Mulvihill WP, Weeks DE (2005)
 Mega2: data-handling for facilitating genetic linkage and association analyses.
 Bioinformatics. 2005 May 15;21(10):2556-7, PMID: 15746282
as well as the version used, which is currently Version 4.7.0
===========================================================
See run summaries in current directory .
   MEGA2.LOG, MEGA2.ERR, MEGA2.KEYS
```
