# Supplementary material for: Mega2: validated data-reformatting for linkage and association analyses
Source: Source Code Biol Med. 2014 Dec 5;9:26. doi: 10.1186/s13029-014-0026-y (PMC4269913; doi:10.1186/s13029-014-0026-y)
Supplement: Additional file 1: — A zipped archive containing the Mega2 version 4.7.1 distribution package; both source and binary executables are included. [file 13029_2014_26_MOESM1_ESM.zip › mega2_v4.7.1_src/example_output_annotated/MEGA2run.html]

 Mega2 Log for Run Jul 22 2014 13:08 


<BODY TEXT="#133b66" BGCOLOR="#ece4ee" LINK="#85050f" ALINK="#991e26" VLINK="#133b66">
This document requires a browser that can display frames.
</body>
