# Supplementary material for: Mega2: validated data-reformatting for linkage and association analyses
Source: Source Code Biol Med. 2014 Dec 5;9:26. doi: 10.1186/s13029-014-0026-y (PMC4269913; doi:10.1186/s13029-014-0026-y)
Supplement: Additional file 1: — A zipped archive containing the Mega2 version 4.7.1 distribution package; both source and binary executables are included. [file 13029_2014_26_MOESM1_ESM.zip › mega2_v4.7.1_src/example_output_ped/MEGA2.LOG.html]

 


 MEGA2.LOG 


```
==========================================================
                          MEGA2 4.7.0
     Copyright (C) 1999-2014 Robert Baron, Charles P. Kollar,
     Nandita Mukhopadhyay, Lee Almasy, Mark Schroeder, William P. Mulvihill,
     Daniel E. Weeks, and University of Pittsburgh

     Last updated: Jul 22 2014, 12:55:24 , valid until June 15, 2015.
     Compiled with gcc version 4.2.1 Compatible Apple LLVM 5.1 (clang-503.0.40)

     Mega2 comes with ABSOLUTELY NO WARRANTY.
     See LICENSE.txt for terms of copying, modifying & redistributing Mega2.
==========================================================
NOTE: If you have previously used explicit numbers for sex chromosomes, BEWARE!
We have changed the numbers to be compatible with PLINK. 23 still codes for X,
but 24 codes for Y and 25 Codes for XY.

Running Mega2 in batch mode from MEGA2.BATCH.ped
Input filenames and missing value indicator read in from batch file.
Analysis option read in from batch file.
Markers, chromosome(s) and read in from batch file.
Trait selection(s) read in from batch file.
Keyword Input_Locus_File not in batch file, Locus file assumed to be unspecified.
Keyword Input_PLINK_Map_File not in batch file, PLINK map file assumed to be unspecified.
Keyword Input_Omit_File not in batch file, Omit file assumed to be unspecified.
Keyword Input_Aux_File not in batch file, Aux file assumed to be unspecified.
Keyword Input_Path not in batch file, using default '.' (current directory).
-----------------------------------------------------
        Mega2 version 4.7.0
Run date:                  2014-7-22-13-08
This file created on       Tue Jul 22 13:08:32 2014
Input file names
#       Pedigree file:               ped.ped
#            Map file:               ped.map
#      Frequency file:               ped.frequency
#     Penetrance file:               ped.penetrance
# PLINK Phenotype file:               ped.phe
  Untyped pedigree option: Include all pedigrees whether typed or not
Mendelianly-inconsistent genotypes included in output.
Half-typed individuals' genotypes included in output.
---------------------------------------------

===========================================================
Analysis option: Mendel7+.
Input Format: PLINK PED format (ped)
Pedigree and map files specified as PLINK format.
omit, penetrance, and frequency files are always in Mega2 format.
Input files will be read in as PLINK or Mega2 format files as appropriate.
reading phenotype file ped.phe ... (1 columns)
Reading PLINK map file for names: ped.map
Reading map file ped.map ... (4 columns)
Found 2 possible maps.
Done reading map file: ped.map

===========================================================
Total number of loci =  5
2 trait loci 
      1 Affection status locus: 
                TRAIT
      1 Quantitative locus: 
                Q1 
      3 Marker loci 
Number of loci found per chromosome (chromosome:number)
   5:3
===========================================================
Reading Mega2 format frequency file: ped.frequency
Reading Mega2 format penetrance file: ped.penetrance
WARNING: Missing QTL phenotype value specified by Value_Missing_Quant_On_Input will be ignored.
Missing QTL phenotype value read from --missing_phenotype (-99.990000).
Reading PLINK .ped file: ped.ped (12 columns).
Reading PLINK format ped file: ped.ped (13 columns)
3 (of 3) markers to be included from ped.mapReading pedigree information from ped.ped
21 individuals read from ped.ped
21 individuals with nonmissing phenotypes
0 cases, 0 controls, 0 missing
9 males, 12 females, 0 of unspecified sex
8 founders, 13 non-founders found
===========================================================
Input pedigree data contains:
Input pedigree file is in PLINK-fam format. 
                                                Marker Genotypes
                                                Fully    Half
     Pedigrees   People   Males   Females       Typed    Typed     Total
TOTAL        2       21       9        12          63        0        63
Typed        2       21       9        12
Untyped      0        0       0         0
===========================================================
Pedigree exclusion option : Include all pedigrees whether typed or not.
Recoding pedigree genotypes ... 
===========================================================
Pedigree data summary after recoding:
Input pedigree file is in PLINK-fam format. 
                                                Marker Genotypes
                                                Fully    Half
     Pedigrees   People   Males   Females       Typed    Typed     Total
TOTAL        2       21       9        12          63        0        63
Typed        2       21       9        12
Untyped      0        0       0         0
===========================================================
Selected map Map.
Selected chromosome 5
Output will combine markers and the following selected traits:
                TRAIT [MARKERS]
After selecting traits and covariates
1 trait locus 
      1 Affection status locus: 
                TRAIT
===========================================================
Pedigree statistics after selecting chromosomes and marker loci:
                                                Marker Genotypes
                                                Fully    Half
     Pedigrees   People   Males   Females       Typed    Typed     Total
TOTAL        2       21       9        12          63        0        63
Typed        2       21       9        12
Untyped      0        0       0         0
===========================================================
Person id in output pedigree file = Individual id
===========================================================
Pedigree id in output pedigree file = Premakeped pedigree number.
===========================================================
Mega2 created the following file(s) for Mendel7+:
        Definition file:      mendel_locus.05
        Pedigree file:        mendel_ped.05
        Map file:             mendel_map.05
        Control file:         mendel_control.05
===========================================================
Output is in ../example_output_ped
===========================================================
If you use Mega2 as part of a published work, please reference 
 Mukhopadhyay N, Almasy L, Schroeder M, Mulvihill WP, Weeks DE (2005)
 Mega2: data-handling for facilitating genetic linkage and association analyses.
 Bioinformatics. 2005 May 15;21(10):2556-7, PMID: 15746282
as well as the version used, which is currently Version 4.7.0
===========================================================
See run summaries in current directory .
   MEGA2.LOG, MEGA2.ERR, MEGA2.KEYS
```
