# Supplementary material for: Mega2: validated data-reformatting for linkage and association analyses
Source: Source Code Biol Med. 2014 Dec 5;9:26. doi: 10.1186/s13029-014-0026-y (PMC4269913; doi:10.1186/s13029-014-0026-y)
Supplement: Additional file 1: — A zipped archive containing the Mega2 version 4.7.1 distribution package; both source and binary executables are included. [file 13029_2014_26_MOESM1_ESM.zip › mega2_v4.7.1_src/example_output_post/MEGA2.ERR.html]

 


 MEGA2.ERR 


```
==========================================================
                          MEGA2 4.7.0
     Copyright (C) 1999-2014 Robert Baron, Charles P. Kollar,
     Nandita Mukhopadhyay, Lee Almasy, Mark Schroeder, William P. Mulvihill,
     Daniel E. Weeks, and University of Pittsburgh

     Last updated: Jul 22 2014, 12:55:24 , valid until June 15, 2015.
     Compiled with gcc version 4.2.1 Compatible Apple LLVM 5.1 (clang-503.0.40)

     Mega2 comes with ABSOLUTELY NO WARRANTY.
     See LICENSE.txt for terms of copying, modifying & redistributing Mega2.
==========================================================
NOTE: If you have previously used explicit numbers for sex chromosomes, BEWARE!
We have changed the numbers to be compatible with PLINK. 23 still codes for X,
but 24 codes for Y and 25 Codes for XY.

-----------------------------------------------------
        Mega2 version 4.7.0
Run date:                  2014-7-22-13-08
This file created on       Tue Jul 22 13:08:32 2014
Input file names
#       Pedigree file:               pedin.ex
#          Locus file:               datain.ex
#            Map file:               map.ex
#           Omit file:               omit.ex
  Untyped pedigree option: Include all pedigrees whether typed or not
Mendelianly-inconsistent genotypes included in output.
Half-typed individuals' genotypes included in output.
---------------------------------------------

WARNING: Inheritance checks may have been inadequate, 
WARNING: Reason: One or more sibships with untyped parents.
WARNING: Please verify with a program for checking Mendelian inconsistencies
WARNING: such as Pedcheck.
```
