# Supplementary material for: Mega2: validated data-reformatting for linkage and association analyses
Source: Source Code Biol Med. 2014 Dec 5;9:26. doi: 10.1186/s13029-014-0026-y (PMC4269913; doi:10.1186/s13029-014-0026-y)
Supplement: Additional file 1: — A zipped archive containing the Mega2 version 4.7.1 distribution package; both source and binary executables are included. [file 13029_2014_26_MOESM1_ESM.zip › mega2_v4.7.1_src/example_output_post/MEGA2.LOG.html]

 


 MEGA2.LOG 


```
==========================================================
                          MEGA2 4.7.0
     Copyright (C) 1999-2014 Robert Baron, Charles P. Kollar,
     Nandita Mukhopadhyay, Lee Almasy, Mark Schroeder, William P. Mulvihill,
     Daniel E. Weeks, and University of Pittsburgh

     Last updated: Jul 22 2014, 12:55:24 , valid until June 15, 2015.
     Compiled with gcc version 4.2.1 Compatible Apple LLVM 5.1 (clang-503.0.40)

     Mega2 comes with ABSOLUTELY NO WARRANTY.
     See LICENSE.txt for terms of copying, modifying & redistributing Mega2.
==========================================================
NOTE: If you have previously used explicit numbers for sex chromosomes, BEWARE!
We have changed the numbers to be compatible with PLINK. 23 still codes for X,
but 24 codes for Y and 25 Codes for XY.

Running Mega2 in batch mode from MEGA2.BATCH.post
Input filenames and missing value indicator read in from batch file.
Analysis option read in from batch file.
Markers, chromosome(s) and read in from batch file.
Trait selection(s) read in from batch file.
Keyword Input_PLINK_Map_File not in batch file, PLINK map file assumed to be unspecified.
Keyword Input_Frequency_File not in batch file, Frequency file assumed to be unspecified.
Keyword Input_Penetrance_File not in batch file, Penetrance file assumed to be unspecified.
Keyword Input_Aux_File not in batch file, Aux file assumed to be unspecified.
Keyword Input_Phenotype_File not in batch file, Phenotype file assumed to be unspecified.
Keyword Input_Path not in batch file, using default '.' (current directory).
-----------------------------------------------------
        Mega2 version 4.7.0
Run date:                  2014-7-22-13-08
This file created on       Tue Jul 22 13:08:32 2014
Input file names
#       Pedigree file:               pedin.ex
#          Locus file:               datain.ex
#            Map file:               map.ex
#           Omit file:               omit.ex
  Untyped pedigree option: Include all pedigrees whether typed or not
Mendelianly-inconsistent genotypes included in output.
Half-typed individuals' genotypes included in output.
---------------------------------------------

===========================================================
Analysis option: CRANEFOOT.
Input Format: Linkage format
Pedigree, names and map file specified as LINKAGE format.
Input files will be read in as LINKAGE format files.
===========================================================
Checking format of pedigree file pedin.ex.
Pedigree file pedin.ex will be read in as post-makeped format.
Reason:
Observed 0s in gender column (5th col).
First occurrence is in line 8.
Observed values > 2 in gender column (5th col).
First occurrence is in line 2.
===========================================================
Locus file is in Linkage format
Reading in map file map.ex.
Data read in from map file.

===========================================================
Locus file is in LINKAGE format.
Total number of loci =  4
1 trait locus 
      1 Affection status locus: 
                TRAIT
      3 Marker loci 
Number of loci found per chromosome (chromosome:number)
   5:2, 6:1
===========================================================
Data read in from pedigree file:
All pedigree records have "Ped" and "Per" fields.
All pedigree records have "ID" fields.
===========================================================
Input pedigree data contains:
Input pedigree file is in post-makeped format.
                                                Marker Genotypes
                                                Fully    Half
     Pedigrees   People   Males   Females       Typed    Typed     Total
TOTAL        2       22      10        12          66        0        66
Typed        2       22      10        12
Untyped      0        0       0         0
===========================================================
Reading omit file omit.ex ...
Untyped all individuals in pedigree 1 at all marker loci.
Untyped individual 10 in pedigree 2 at locus M2.
Untyped all individuals in pedigree 2 at locus M1.
Pedigree exclusion option : Include all pedigrees whether typed or not.
===========================================================
After reading in omit file and excluding untyped pedigrees :
------------------------------------------------------------
Completely untyped pedigrees:
1 
Input pedigree file is in post-makeped format.
                                                Marker Genotypes
                                                Fully    Half
     Pedigrees   People   Males   Females       Typed    Typed     Total
TOTAL        2       22      10        12          21        0        66
Typed        1       11       5         6
Untyped      1       11       5         6
===========================================================
Connecting loops in pedigree 2...
Ped 2: Loop-breaker ids 2_8 and 2_11
        Saving 2_8, deleting 2_11
Selected map Map.
You have selected the following chromosome(s) :
  05 06
===========================================================
Output will combine markers and the following selected traits:
                TRAIT [MARKERS]
After selecting traits and covariates
1 trait locus 
      1 Affection status locus: 
                TRAIT
WARNING: Inheritance checks may have been inadequate, 
WARNING: Reason: One or more sibships with untyped parents.
WARNING: Please verify with a program for checking Mendelian inconsistencies
WARNING: such as Pedcheck.
===========================================================
===========================================================
Pedigree statistics after selecting chromosomes and marker loci:
------------------------------------------------------------
Completely untyped pedigrees:
1 
                                                Marker Genotypes
                                                Fully    Half
     Pedigrees   People   Males   Females       Typed    Typed     Total
TOTAL        2       21       9        12          19        0        63
Typed        1       10       4         6
Untyped      1       11       5         6
===========================================================
Output file names set to defaults.
Mega2 created the following file(s) for CRANEFOOT:
    CRANEFOOT pedigree file:           crnft_ped.05
    CRANEFOOT control file:            crnft_control.05
    CRANEFOOT pedigree file:           crnft_ped.06
    CRANEFOOT control file:            crnft_control.06
    C-shell script:                    crnft_shell.all.sh
Output is in ../example_output_post
===========================================================
If you use Mega2 as part of a published work, please reference 
 Mukhopadhyay N, Almasy L, Schroeder M, Mulvihill WP, Weeks DE (2005)
 Mega2: data-handling for facilitating genetic linkage and association analyses.
 Bioinformatics. 2005 May 15;21(10):2556-7, PMID: 15746282
as well as the version used, which is currently Version 4.7.0
===========================================================
See run summaries in current directory .
   MEGA2.LOG, MEGA2.ERR, MEGA2.KEYS
```
