# Supplementary material for: Mega2: validated data-reformatting for linkage and association analyses
Source: Source Code Biol Med. 2014 Dec 5;9:26. doi: 10.1186/s13029-014-0026-y (PMC4269913; doi:10.1186/s13029-014-0026-y)
Supplement: Additional file 1: — A zipped archive containing the Mega2 version 4.7.1 distribution package; both source and binary executables are included. [file 13029_2014_26_MOESM1_ESM.zip › mega2_v4.7.1_src/example_output_post/MEGA2links.html]

 List of files 


**Run date:**  Jul 22 2014 13:08   
 **Run label:**  2014-7-22-13-08

## Log files

|  |
| --- |
| MEGA2.LOG |
| MEGA2.ERR |
| MEGA2.KEYS |

## Input files

|  |  |
| --- | --- |
| Locus file | datain.ex |
| Pedigree file | pedin.ex |
| Map file | map.ex |
| Omit file | omit.ex |
| Batch file | MEGA2.BATCH.post |
