# Supplementary material for: Mega2: validated data-reformatting for linkage and association analyses
Source: Source Code Biol Med. 2014 Dec 5;9:26. doi: 10.1186/s13029-014-0026-y (PMC4269913; doi:10.1186/s13029-014-0026-y)
Supplement: Additional file 1: — A zipped archive containing the Mega2 version 4.7.1 distribution package; both source and binary executables are included. [file 13029_2014_26_MOESM1_ESM.zip › mega2_v4.7.1_src/example_output_post/MEGA2outputfiles.html]

 List of output files 


## Output files

|  |  |  |  |
| --- | --- | --- | --- |
| crnft\_ped.05 | crnft\_control.05 | crnft\_ped.06 | crnft\_control.06 |
| crnft\_shell.all.sh |
