# Supplementary material for: Mega2: validated data-reformatting for linkage and association analyses
Source: Source Code Biol Med. 2014 Dec 5;9:26. doi: 10.1186/s13029-014-0026-y (PMC4269913; doi:10.1186/s13029-014-0026-y)
Supplement: Additional file 1: — A zipped archive containing the Mega2 version 4.7.1 distribution package; both source and binary executables are included. [file 13029_2014_26_MOESM1_ESM.zip › mega2_v4.7.1_src/example_output_pre/MEGA2.KEYS.html]

 


 MEGA2.KEYS 


```
-----------------------------------------------------
        Mega2 version 4.7.0
Run date:                  2014-7-22-13-08
This file created on       Tue Jul 22 13:08:32 2014
Input file names
#       Pedigree file:               pedin.pre.05
#          Locus file:               datain.05
#            Map file:               map.05
  Untyped pedigree option: Include all pedigrees whether typed or not
Mendelianly-inconsistent genotypes included in output.
Half-typed individuals' genotypes included in output.
---------------------------------------------

INPUT                                         OUTPUT   
Numeric     Original    Unique        
NPed  NPer  OPed  OPer  Ped   ID:    Loop id  Pedigree  Person 
1     1     1     1     1     1_1             1         1      
1     2     1     2     1     1_2             1         2      
1     3     1     3     1     1_3             1         3      
1     4     1     4     1     1_4             1         4      
1     5     1     5     1     1_5             1         5      
1     6     1     6     1     1_6             1         6      
1     7     1     7     1     1_7             1         7      
1     8     1     8     1     1_8             1         8      
1     9     1     9     1     1_9             1         9      
1     10    1     10    1     1_10            1         10     
1     11    1     11    1     1_11            1         11     
2     1     2     1     2     2_1             2         1      
2     2     2     2     2     2_2             2         2      
2     3     2     3     2     2_3             2         3      
2     4     2     4     2     2_4             2         4      
2     5     2     5     2     2_5             2         5      
2     6     2     6     2     2_6             2         6      
2     7     2     7     2     2_7             2         7      
2     8     2     8     2     2_8             2         8      
2     9     2     9     2     2_9             2         9      
2     10    2     10    2     2_10            2         10
```
