# Supplementary material for: Mega2: validated data-reformatting for linkage and association analyses
Source: Source Code Biol Med. 2014 Dec 5;9:26. doi: 10.1186/s13029-014-0026-y (PMC4269913; doi:10.1186/s13029-014-0026-y)
Supplement: Additional file 1: — A zipped archive containing the Mega2 version 4.7.1 distribution package; both source and binary executables are included. [file 13029_2014_26_MOESM1_ESM.zip › mega2_v4.7.1_src/mega2_html/Mega2_overview.pdf]

Mega2 (Manipulation Environment for Genetic Analysis)

C.P. Kollar[1], R. V. Baron[1], N. Mukhopadhyay[2], D. E. Weeks[1,3] 1) Department of Human Genetics; 2) Department of Oral Biology, 3) Department of Biostatistics, University of Pittsburgh, Pittsburgh, PA.

What is Mega2:

Data reformatting tool for genetic analysis

Motivation:

• Need to use more than one analysis type in a study.

• Analysis data formats differ and writing custom transformation scripts is error prone.

Features:

• Analysis-ready datasets for 37 different genetic analysis programs.

• Allows filtering of data.

• Eliminates the need to write custom data transformation scripts.

Recent improvements:

• Addition of 11 new analysis output formats.

• Speed and memory footprint for large-scale data handling.

• Examples of improved performance:

2.2K individuals, 1 trait, 12K markers: average processing time of 10.5 sec.

3.1K individuals, 895K markers: now fits in only 1.1 Gb RAM.

Availability:

• Open source (C++) implementation freely available.

• Runs on Linux, Mac, Solaris, and Windows (binaries available for popular platforms)

• Full documentation.

• Prompt friendly technical support.

• Under continuous development and maintenance.

• Available from: http://watson.hgen.pitt.edu/register/

Input

Mega2	Linkage	PLINK	VCF

Processing

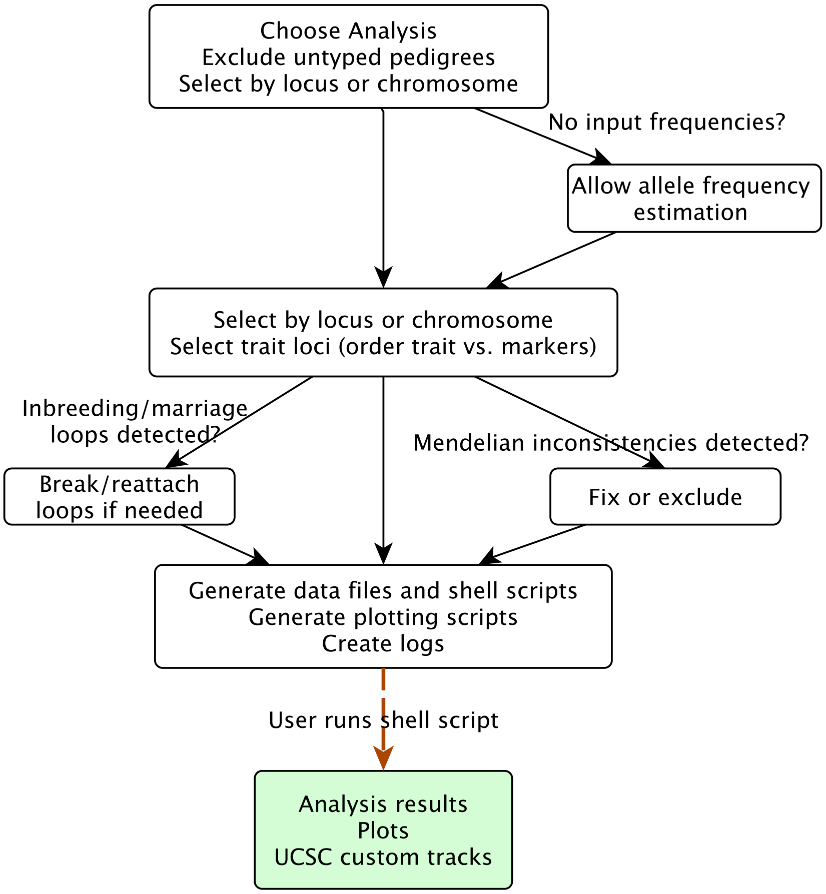

Output/Analysis

Recently Added

PLINK/SEQ	Structure	Eigenstrat	Beagle
Morgan	FBAT	IQLS	Mega2 Annot
Cranefoot	PLINK	SUP	Mendel
Loki	Merlin	PAP	PREST
SimWalk2-NPL	Pre-makeped	SAGE	MLBQTL
Allegro	HWE test	Linkage	Vitesse
SOLAR	SIMULATE	Homogeneity	SPLINK
SLINK	Nuclear	GeneHunter+	SimWalk2

Reference: Mukhopadhyay N, Almasy L, Schroeder M, Mulvihill WP, Weeks DE (2005) Mega2: data-handling for facilitating genetic linkage and association analysis. Bioinformatics. 2005 May 15; 21(10):2556-7. PMID: 15746282

Acknowledgments: NIH/NIGMS grant R01 GM076667 (PI: Weeks)
