# Supplementary material for: Mega2: validated data-reformatting for linkage and association analyses
Source: Source Code Biol Med. 2014 Dec 5;9:26. doi: 10.1186/s13029-014-0026-y (PMC4269913; doi:10.1186/s13029-014-0026-y)
Supplement: Additional file 1: — A zipped archive containing the Mega2 version 4.7.1 distribution package; both source and binary executables are included. [file 13029_2014_26_MOESM1_ESM.zip › mega2_v4.7.1_src/mega2_html/mega2_documentation.html]

xml version="1.0" encoding="UTF-8"?


Mega2Manipulation Environment for Genetic AnalysesA data-handling program for facilitatinggenetic linkage and association analyses


# Mega2 “Manipulation Environment for Genetic Analyses” A data-handling program for facilitating genetic linkage and association analyses

## Oct 17, 2014

|  |
| --- |
| *Daniel E. Weeks* |
| Department of Human Genetics, University of Pittsburgh |
| Copyright © 1997-2014 by the University of Pittsburgh |
| Current Programmer: *Robert V. Baron* |
| Previous Programmers: *Charles P. Kollar, Nandita Mukhopadhyay, Lee Almasy, Mark Schroeder, William P. Mulvihill.* |
| *Jia Jia and Xinyu Tang helped with quality assurance testing.* |
| *Xinyu Tang also helped with utility development.* |

\hrule

***Note:*** This documentation is available in PDF form from https://watson.hgen.pitt.edu/docs/mega2\_html/Mega2\_Documentation.pdf and it is also included in the mega2\_html folder of the Mega2 distribution.

\hrule

Table of Contents

Section 1: Introduction

Subsection 1.1: Graphical overview of Mega2

Subsection 1.2: Supported formats

Subsubsection 1.2.1: Input formats

Subsubsection 1.2.2: Output formats

Section 2: Download Mega2

Section 3: Recent improvements and changes

Subsection 3.1: Enhancements in Mega2 version 4.7.1

Subsection 3.2: Enhancements in Mega2 version 4.7.0

Subsection 3.3: Enhancements in Mega2 version 4.6.2

Subsection 3.4: Enhancements in Mega2 version 4.6.1

Section 4: Quick start - get Mega2 running in minutes

Subsection 4.1: The fast, easy way to set up your files for Mega2

Subsection 4.2: Tips on more complex data

Subsection 4.3: Word of caution regarding input file names

Section 5: Citing Mega2

Section 6: Support, bug reports, and feedback

Subsection 6.1: Mega2 feedback

Subsection 6.2: Mega2 Google Group

Subsection 6.3: Bug reports

Section 7: Contact information

Section 8: Installation

Subsection 8.1: Download Instructions

Subsubsection 8.1.1: Mega2 Bitbucket repository

Subsection 8.2: Prerequisites for Mega2

Subsubsection 8.2.1: R

Subsubsection 8.2.2: R Libraries

Subsection 8.3: Installing Mega2 from a Binary Package

Subsubsection 8.3.1: Contents of a binary package

Subsubsection 8.3.2: Mega2 Binaries

Subsubsection 8.3.3: Installing Mega2

Subsection 8.4: Compiling and Installing Mega2 from Source

Subsection 8.5: Running Mega2 on Your Data

Subsection 8.6: Mega2 Documentation

Subsection 8.7: License Agreement

Subsection 8.8: Feedback and Bug Reports

Subsection 8.9: Macintosh-specific installation issues

Subsubsection 8.9.1: Introduction to Command line programs for Mac users

Subsubsection 8.9.2: Xcode compilation environment

Subsection 8.10: Unix-specific installation issues

Subsection 8.11: Windows Cygwin installation

Subsubsection 8.11.1: Cygwin POSIX windows environment

Subsubsection 8.11.2: R installation:

Subsubsection 8.11.3: Mega2 Download instructions for Windows

Subsection 8.12: Windows Mingw installation

Subsubsection 8.12.1: Mingw POSIX windows environment

Subsection 8.13: Native Windows installation

Subsubsection 8.13.1: Native Windows environment

Section 9: Input file formats

Subsection 9.1: Mega2 input file formats

Subsubsection 9.1.1: Mega2 Names file

Subsubsection 9.1.2: Mega2 Pedigree file

Subsubsection 9.1.3: Mega2 Map file

Subsubsection 9.1.4: Omit file

Subsubsection 9.1.5: Frequency file

Subsubsection 9.1.6: Penetrance file

Subsection 9.2: PLINK input file formats

Subsubsection 9.2.1: PLINK PED input format

Subsubsection 9.2.2: PLINK binary PED input format

Subsection 9.3: LINKAGE input file formats

Subsubsection 9.3.1: LINKAGE locus file

Subsubsection 9.3.2: LINKAGE pedigree file

Subsubsection 9.3.3: Handling of loops inside pedigrees

Subsubsection 9.3.4: Simple Mega2 Map file

Subsubsection 9.3.5: Specifying sex-specific maps

Subsubsection 9.3.6: Omit file [Optional]

Subsubsection 9.3.7: Header-less Names file

Subsection 9.4: Variant Call Format (VCF, BCF, compressed VCF) input file formats

Subsubsection 9.4.1: Variant Call File

Subsubsection 9.4.2: PLINK family File

Subsubsection 9.4.3: Augmented PLINK Phenotype File

Subsubsection 9.4.4: Map File (Optional)

Subsubsection 9.4.5: Omit File (Optional)

Subsubsection 9.4.6: Frequency File (Optional)

Subsubsection 9.4.7: Penetrance File (Optional)

Section 10: Genetic Map Interpolator (GMI)

Section 11: Converting to Mega2’s format

Subsection 11.1: Pedigree file conversion

Subsection 11.2: Locus file conversion

Subsection 11.3: Map file conversion

Section 12: The input menu

Subsection 12.1: Input formats: Mega2 and Linkage

Subsection 12.2: Common PLINK menu items

Subsection 12.3: Input format: PLINK binary PED

Subsection 12.4: Input format: PLINK ped

Subsection 12.5: Common Variant Call Format menu items

Subsection 12.6: Input format: Variant Call Format files

Subsection 12.7: Input menu items: Omit, frequency and penetrance data files (optional):

Subsection 12.8: Input menu item: Output Directory:

Subsection 12.9: Input menu item: Simulating genotyping errors:

Subsection: Genotyping error simulation

Subsection: Parameter selection menu

Subsection: Error simulation output files

Subsection 12.10: Input menu item: Untyped pedigree exclusion option:

Subsection 12.11: Input menu item: Upper limit for squared deviation between input and observed allele frequencies

Subsection 12.12: Input menu item: Unknown allele and affection definition

Subsection 12.13: Input menu item: Maximum number of alleles per marker

Subsection 12.14: Errors in input data

Subsubsection 12.14.1: Problems with locus data

Subsubsection 12.14.2: Problems with pedigree data.

Subsubsection 12.14.3: Genotype reset menu

Section 13: The analysis menu

Subsection 13.1: Create SimWalk2 format files

Subsection 13.2: Convert to Vintage Mendel format

Subsection 13.3: Convert to ASPEX format

Subsection 13.4: Convert to GeneHunter-Plus format

Subsection 13.5: Convert to GeneHunter format

Subsection 13.6: Convert to APM format [DISABLED]

Subsection 13.7: Convert to APM MULT multiple locus format [DISABLED]

Subsection 13.8: Create nuclear families

Subsection 13.9: Convert to SLINK format

Subsection 13.10: Convert to SPLINK format

Subsection 13.11: Set up for homogeneity analyses

Subsection 13.12: Convert to SIMULATE format

Subsection 13.13: Create summary files

Subsection 13.14: Convert to Old SAGE format

Subsection 13.15: Set up for TDTMax analyses[DISABLED]

Subsection 13.16: Convert to SOLAR format

Subsection 13.17: Convert to Vitesse format

Subsection 13.18: Convert to Linkage format

Subsection 13.19: Test loci for Hardy-Weinberg Equilibrium

Subsection 13.20: Convert to Allegro format

Subsection 13.21: Convert to MLBQTL format

Subsection 13.22: Convert to SAGE format

Subsection 13.23: Convert to pre-makeped format

Subsection 13.24: Setup for Merlin-SimWalk2 combined analysis

Subsection 13.25: Convert to PREST format

Subsection 13.26: Convert to PAP format

Subsection 13.27: Convert to Merlin format

Subsection 13.28: Convert to LOKI format

Subsection 13.29: Convert to Mendel format

Subsection 13.30: Convert to SUP format

Subsection 13.31: Convert to PLINK format

Subsection 13.32: Convert to CRANEFOOT format

Subsection 13.33: Convert to Mega2 format

Subsection 13.34: Convert to IQLS/Idcoefs format

Subsection 13.35: Convert to FBAT format

Subsection 13.36: Convert to Morgan format

Subsection 13.37: Convert to Beagle format

Subsection 13.38: Convert to Eigenstrat format

Subsection 13.39: Convert to Structure format

Subsection 13.40: Convert to PSEQ (PLINK/SEQ) format

Section 14: The Missing Value Menu

Section 15: The allele frequency menu

Subsection 15.1: The recoding process

Subsection 15.2: The recode log file

Subsection 15.3: Penetrances for affection status loci

Section 16: The locus reordering menu

Subsection 16.1: Locus reordering option 1) Select all loci in map order on chromosome.

Subsection 16.2: Locus reordering option 2) Select by locus number.

Subsection 16.3: Locus reordering option 3) Select marker loci on multiple chromosomes.

Section 17: Map Selection Menus

Subsection 17.1: The Genetic Map Selection Menu

Subsection 17.2: The Physical Map Selection Menu

Section 18: The trait selection menu

Subsection 18.1: Select multiple trait loci to loop across.

Subsection 18.2: Use trait loci in specified order.

Subsection 18.3: List of selected trait loci

Subsection 18.4: Selection of covariates

Subsection 18.5: More details on trait selection.

Subsection 18.6: Affection status labels

Section 19: Creating plots using Mega2

Subsection 19.1: Statistics selection menu

Subsection 19.2: R-plot parameters menu

Subsection 19.3: General usage of nplplot and nplplot.multi

Section 20: Custom tracks for the UCSC Browser

Section 21: Additional Mega2 Output Files

Subsection 21.1: Summary file directory

Subsection 21.2: MEGA2.LOG

Subsection 21.3: MEGA2.ERR

Subsection 21.4: MEGA2.BATCH

Subsection 21.5: MEGA2.KEYS

Subsection 21.6: MEGA2.SIM

Subsection 21.7: MEGA2.RECODE

Subsection 21.8: MEGA2run.html and related files

Section 22: Mega2 command-line arguments

Subsection 22.1: Species options

Subsection 22.2

Subsection 22.3

Subsection 22.4

Subsection 22.5: Obsolete options

Subsection 22.6: *Other arguments*

Section 23: Running Mega2 in Batch mode

Subsection 23.1: Overview

Subsection 23.2: Using the *--*nosave option

Subsection 23.3: Batch file format

Subsection 23.4: Major classes of batch file options

Subsection 23.5: Details on batch file items

Section 24: Hints and troubleshooting

Subsection 24.1: Hints on loci reordering

Subsection 24.2: Problems commonly encountered with input files:

Subsection 24.3: Pedigree file reading problem

Subsection 24.4: Mega2 hangs while reading in input files

Subsection 24.5: DOS format file related errors

Subsubsection 24.5.1: Error messages

Subsection 24.6: Non-DOS related problems

Section 25: Detailed information on analysis options

Subsection 25.1: Create SimWalk2 format files

Subsection 25.2: Convert to MENDEL format

Subsection 25.3: Convert to ASPEX format

Subsection 25.4: Convert to GeneHunter-Plus format

Subsection 25.5: Convert to GeneHunter format

Subsection 25.6: Convert to APM format

Subsection 25.7: Convert to APM MULT multiple locus format

Subsection 25.8: Create nuclear families

Subsection 25.9: Convert to SLINK format

Subsection 25.10: Convert to SPLINK format

Subsection 25.11: Set up for homogeneity analyses

Subsection 25.12: Convert to SIMULATE format

Subsection 25.13: Create summary files

Subsection 25.14: Convert to SAGE format

Subsection 25.15: Set up for TDTMax analyses

Subsection 25.16: Convert to SOLAR format

Subsection 25.17: Convert to Vitesse format

Subsection 25.18: Convert to Linkage format

Subsection 25.19: Test loci for Hardy-Weinberg Equilibrium

Subsection 25.20: Convert to Allegro format

Subsection 25.21: Convert to MLBQTL format

Subsection 25.22: Convert to S.A.G.E. 4.0 format

Subsection 25.23: Convert to pre-makeped format

Subsection 25.24: Convert to Merlin-SimWalk2 format

Subsection 25.25: Convert to PREST format

Subsection 25.26: Convert to PAP format

Subsection 25.27: Convert to Merlin format

Subsection 25.28: Convert to Loki format

Subsection 25.29: Convert to Mendel format

Subsection 25.30: Convert to SUP format

Subsection 25.31: Convert to PLINK format

Subsection 25.32: Convert to Cranefoot format

Subsection 25.33: Convert to Mega2 format

Subsection 25.34: Convert to IQLS/Idcoefs format

Subsection 25.35: Convert to FBAT format

Subsection 25.36: Convert to PANGAEA MORGAN format

Subsection 25.37: Convert to Beagle format

Subsection 25.38: Convert to Eigenstrat format

Subsection 25.39: Convert to Structure format

Subsection 25.40: Convert to PSEQ (PLINK/SEQ) format

Section 26: Utilities included with Mega2

Subsection 26.1: Converting linkage format files to Mega2 format - l2a.py

Subsection 26.2: Map making utilities

Subsection 26.3: Creating a Mega2 omit file

Subsection 26.4: Scripts to generate formatted output for Hardy-Weinberg test:

Section 27: List of third-party applications used by Mega2

Subsection 27.1: R statistical package and its libraries

Subsubsection 27.1.1: R Installation

Subsection 27.2: Python

Subsubsection 27.2.1: Python Installation

Subsection 27.3: Perl

Subsubsection 27.3.1: Perl Installation

Subsection 27.4: Awk

Subsubsection 27.4.1: Awk Installation

Subsection 27.5: C-shell

Section 28: Changes made to Mega2

Subsection 28.1: Recent releases

Subsection 28.2: Changes from Version 4.6.2 to Version 4.7.1 (Released Oct 16, 2014)

Subsection 28.3: Changes from Version 4.6.2 to Version 4.7.0 (Released May 15, 2014)

Subsection 28.4: Changes from Version 4.6.1 to Version 4.6.2 (Released Feb 28, 2014)

Subsection 28.5: Changes from Version 4.6.0 to Version 4.6.1 (Released Oct 21st, 2013)

Subsection 28.6: Changes from Version 4.5.9 to Version 4.6.0 (Released Sept 6th, 2013)

Subsection 28.7: Changes from Version 4.5.8 to Version 4.5.9 (Released July 5th, 2013)

Subsection 28.8: Changes from Version 4.5.7 to Version 4.5.8 (Released June 6th, 2013)

Subsection 28.9: Changes from Version 4.5.6 to Version 4.5.7 (Released January 11th, 2013)

Subsection 28.10: Changes from Version 4.5.5 to Version 4.5.6 (Released July 6th, 2012)

Subsection 28.11: Changes from Version 4.5.4 to Version 4.5.5 (Released June 15th, 2012)

Subsection 28.12: Changes from Version 4.5.3 to Version 4.5.4 (Released July 26th, 2011)

Subsection 28.13: Changes from Version 4.0 R5.2 Beta - Version 4.5.3 (Released June 15th, 2011)

Subsection: Changes from Ver 4.0 R5.1 - Ver 4.0 R5.2 Beta

Subsection: Changes from Ver 4.0 R5.0 - Ver 4.0 R5.1 (Released June 15th, 2010)

Subsection: Changes from Ver 4.0 R4.0 - Ver 4.0 R5.0 (Released Dec 31st, 2009)

Subsection: Changes from Ver 4.0 R3.1 - Ver 4.0 R4.0 (Released October 4th, 2009)

Subsection: Changes from Ver 4.0 R3 - Ver 4.0 R3.1 (Released July 12, 2009)

Subsection: Changes from Ver 4.0 R2 - Ver 4.0 R3 (Released Jun 15, 2009)

Subsection: Changes from Ver 4.0 R1 - Ver 4.0 R2 (Released April 15, 2009)

Subsection: Changes from Ver 4.0 - Ver 4.0 R1 (Released Jun 13, 2008)

Subsection: Changes from Ver 4.0 Beta R1 - Ver 4.0 (Released March 31, 2008)

Subsection: Changes from Ver 4.0 Beta- Ver 4.0 Beta R1 (Released August 7, 2007)

Subsection: Changes from Ver 3.0 R11- Ver 3.0 R12 and Ver 4.0 Beta (Released June 14, 2007)

Subsection: Changes from Ver 3.0 R10- Ver 3.0 R11 (Released May 17, 2007)

Subsection: Changes from Ver 3.0 R9- Ver 3.0 R10 (Released February 1, 2007)

Subsection: Very old versions

Subsection: Changes from Ver 3.0 R8- Ver 3.0 R9 (Released July 14, 2006)

Subsection: Changes from Ver 3.0 R7- Ver 3.0 R8 (Released June 19, 2006)

Subsection: Changes from Ver 3.0 R5,R6 - Ver 3.0 R7 (Released June 15, 2006)

Subsection: Changes from Ver 3.0 R4 - Ver 3.0 R5 (Released Feb 2, 2006)

Subsection: Changes from Ver 3.0 R3 - Ver 3.0 R4 (Released June 10, 2005)

Subsection: Changes from Ver 3.0 R2 - Ver 3.0 R3 (Released November 29, 2004)

Subsection: Changes from Ver 3.0 R1 - Ver 3.0 R2 (Released September 30, 2004)

Subsection: Changes from Ver 3.0 - Ver 3.0 R1 (Released August 15, 2004)

Subsection: Changes from Ver 2.5 R4 - Ver 3.0 (Released June 15, 2004)

Subsection: Changes from Ver 2.5 R3 - Ver 2.5 R4 (Released April 22, 2004)

Subsection: Changes from Ver 2.5 R2 - Ver 2.5 R3 (Released April 15, 2004)

Subsection: Changes from Ver 2.5 R1 - Ver 2.5 R2 (Released August 8, 2003)

Subsection: Changes from Ver 2.5 - Ver 2.5 R1 (Released July 5th, 2003)

Subsection: Changes from Ver 2.3 R4 - Ver 2.5 (Released June 3, 2003)

Subsection: Changes from Ver 2.3 R3 - Ver 2.3 R4 (Released Feb 7, 2003)

Subsection: Changes from Ver 2.3 R2 - Ver 2.3 R3 (Released Dec 13, 2002)

Subsection: Changes from Ver 2.3 - Ver 2.3 R2 (Released July 20 2002)

Subsection: Changes from Ver 2.2 R3 - Ver 2.3 (Released June 14 2002)

Subsection: Changes from Ver 2.2 R2 - Ver 2.2 R3 (Released January 23rd, 2002)

Subsection: Changes from version 2.2 - version 2.2 R2 (Released 28th June 2001)

Subsection: Changes from version 2.1 beta R3 - version 2.2 (Released 15 Jun 2001)

Subsection: Changes from version 2.1 beta R2 - version 2.1 beta R3 (Released 30 Mar 2001)

Subsection: Changes from version 2.1 beta - version 2.1 beta R2 (Released 16 Mar 2001)

Subsection: Changes from version 2.05 to version 2.1 beta (Released 16 Feb 2001)

Section 29: List of fixed bugs

Subsection: Bugs in Mega2 4.0 R5.2 Beta

Subsection: Bugs in Mega2 4 R5.1

Subsection: Bugs in Mega2 4 R5.0

Subsection: Bugs in Mega2 4 R4.0

Subsection: Bugs in Mega2 4 R3.1

Subsection: Bugs in Mega2 4 R3

Subsection: Bugs in Mega2 4.0 R1

Subsection: Bugs in Mega2 4.0

Subsection: Bugs in Mega2 4.0 beta R1

Subsection: Bugs in Mega2 4.0 beta

Subsection: Bugs in Mega2 3.0 R11

Subsection: Bugs in Mega2 3.0 R10

Subsection: Bugs in Mega2 3.0 R9

Subsection: Bugs in Mega2 3.0 R8

Subsection: Bugs in Mega2 3.0 R7

Subsection: Bugs in Mega2 3.0 R5, R6

Subsection: Bugs in Mega2 3.0 R4

Subsection: Bugs in Mega2 3.0 R3

Subsection: Bugs in Mega2 3.0 R2

Subsection: Bugs in Mega2 3.0 R1

Subsection: Bugs in Mega2 3.0

Subsection: Bugs in Mega2 2.5 R4

Subsection: Bugs in Mega2 2.5 R3

Subsection: Bugs in Mega2 2.5 R2

Subsection: Bugs in Mega2 2.5 R1

Subsection: Bugs in Mega2 2.5

Subsection: Bugs in Mega2 2.3 R4

Subsection: Bugs in Mega2 2.3 R3

Subsection: Bugs in Mega2 2.3 R2

Subsection: Bugs in Mega2 2.3 and Mega2 2.3 R1

Subsection: Bugs in Mega2 2.2 R3

Subsection: Bugs in Mega2 2.2 R2

Subsection: Bugs in Mega2 2.2

Subsection: Bugs in Mega2 2.1 beta

Section 30: License agreements

Subsection 30.1: GNU General Public License Version 3 for Mega2

Subsection 30.2: MIT License for VCFtools

Subsection 30.3: License for ZLIB

Subsection 30.4: GNU Lesser General Public License Version 3 for VCFtools

Section 31: PDF documentation

Section 32: Grant Acknowledgments

Section 33: References

# 1 Introduction

During an association or linkage analysis project, one may need to analyze the data with several different programs. Unfortunately, it can often be quite difficult to get one’s data in the proper format desired by each different computer program. Not only must the data be converted to the proper format, but also the loci must be reordered into their proper order. Writing custom reformatting scripts can be error-prone and very time-consuming. To address these problems, we created Mega2, which can be obtained following the instructions in Section 8↓.

The Quick Start Mega2 tutorial in Section 4↓ will help you get started using Mega2.

Mega2 can read input data in several formats: LINKAGE format, PLINK format, and VCF format (as listed here: 1.2.1↓). Mega2 allows one to augment these input formats with additional information, if desired. For example, trait locus pentrance information can be specified.

Mega2 then takes the input files and, via a menu-driven interface, transforms them into various other file formats (listed here 1.2.2↓), thus greatly facilitating a variety of different analyses. In addition, for many of these options, it also sets up a shell script that then can automatically run these analyses (if you are using Mega2 in a Unix or Macintosh environment).

Mega2 is currently structured so that the user proceeds through a series of menus, making choices in each one (or accepting the default values), until the desired output files are created. After the desired output files are created, Mega2 exits. It can also be run in a “hands-free” mode, using a control file to specify these choices.

In addition to the ability to reformat data for a variety of analysis programs, other useful features of Mega2 include:

1. The ability to create publication-quality PDF plots of the results using our nplplot R library (See Section 19↓).
2. The ability to create custom tracks of results for visualization in the UCSC genome browser (See Section 20↓).
3. The ability to run in an automated way using batch files (See Section 23↓).
4. The availability of our Genetic Map Interpolator for aiding in constructing genetic maps of markers (See Section 10↓).
5. The ability to simulate genotype errors (See section 12.9↓).
6. Input and output support for Mega2 format files that contain informative header lines and are readable into R (See Section 9.1↓).
7. Input and output support for the widely-used PLINK format files (See Section 9.2↓).
8. Input support for Variant Call Format (VCF, BCF, compressed VCF) files, including flexible filtering on input (See Section 9.4↓).
9. The ability to automatically zero out selected genotypes for specific individuals in order to resolve Mendelian inconsistencies (See Section 9.1.4↓).
10. In most cases, in addition to generating appropriately re-formatted files, Mega2 also generates a shell script that will automatically run the desired program.
11. Creation of an HTML summary of the most recent run of Mega2, with links to input and output and log files (See Section 21.8↓).

## 1.1 Graphical overview of Mega2

\end

## 1.2 Supported formats

Mega2 can convert data from three primary input formats to a large set of target output formats.

### 1.2.1 Input formats

Mega2 supports the following input file formats:

1. Mega2 format (See 9.1↓)
2. LINKAGE format (See 9.3↓)
3. PLINK format (See 9.2↓)
4. Variant Call Format (VCF, BCF, compressed VCF) (See 9.4↓)

### 1.2.2 Output formats

Mega2 supports the following output file formats (For more detail, see Sections 13↓ and 25↓):

1. SimWalk2 format
2. Vintage Mendel format
3. ASPEX format
4. GeneHunter-Plus format
5. GeneHunter format
6. Create nuclear families
7. SLINK format
8. SPLINK format
9. Homogeneity analyses
10. SIMULATE format
11. Create summary files
12. Old SAGE format
13. SOLAR format
14. Vitesse format
15. Linkage format
16. Test loci for HWE
17. Allegro format
18. MLBQTL format
19. SAGE format
20. Pre-makeped format
21. Merlin/SimWalk2-NPL format
22. PREST format
23. PAP format
24. Merlin format
25. Loki format
26. Mendel format
27. SUP format
28. PLINK format
29. CRANEFOOT format
30. Mega2 format
31. IQLS/Idcoefs format
32. FBAT format
33. PANGAEA MORGAN format
34. Beagle format
35. Eigenstrat format
36. Structure format
37. PSEQ format

\end

# 2 Download Mega2

Mega2 can be obtained from https://watson.hgen.pitt.edu/register/ .

Detailed installation instructions can be found in Section 8↓.

# 3 Recent improvements and changes

## 3.1 Enhancements in Mega2 version 4.7.1

- Mega2 now gives you complete control to specify missing values for quantitative and affection phenotypes, both in the input data and for the data that Mega2 will output.
- Mega2’s performance is almost twice as fast as Version 4.7.0

## 3.2 Enhancements in Mega2 version 4.7.0

- Mega2 now passes non-numeric alleles through (without recoding them to numeric alleles) to analysis programs that will accept non-numeric alleles (section 15↓). This simplifies analyses, as it avoids relabelling alleles, making it easier to interpret the resulting output. Conversion to numeric alleles can still be requested if desired (section 22.4↓).
- Bug Fix: A number of problems with Beagle Analysis have been addressed.
- Bug Fix: Resolved an issue specifying PLINK mode interactively.
- Bug Fix: Problems discovered via the CLANG/LLVM compiler have been addressed.

## 3.3 Enhancements in Mega2 version 4.6.2

- Mega2 can now read VCF, BCF, and compressed VCF input files, while applying optional ’VCFtools’ filters (See section 9.4↓).
- Initial interactive menus have been modified to improve the specification of different input file types (See section 12↓).

## 3.4 Enhancements in Mega2 version 4.6.1

- Mega2 can now produce PLINK/SEQ format files and a script for loading these files into a PLINK/SEQ project (See section 25.40↓).
- Bug fix: Mega2 4.6.0 had broken support for Linkage format input when using a “Names file” (See section 9.3.7↓).
- Added binaries for native Microsoft Windows 8 and MinGW built on Microsoft Windows 8.

---

# 4 Quick start - get Mega2 running in minutes

## 4.1 The fast, easy way to set up your files for Mega2

Minimal input to Mega2 consists of three matched files: (1) a names file; (2) a pedigree file; and (3) a map file. We illustrate this here with a simple example pedigree:

This pedigree consists of the mother, father and their three offspring. The mother and two of the siblings are considered to be affected for a binary trait called ’Trait’ (as indicated by the shaded symbols), and all five pedigree members are also genotyped at a genetic marker ’Marker1’.

To analyze this pedigree, you need to create (1) a names file containing the trait and marker locus names, (2) a pedigree file with relationship, phenotype and genotype information, and (3) a map file indicating which chromosome Marker1 is on and its genetic map position.

1. Names file:

   Mega2 supports the use of the relatively simple QTDT format "names" file (used by Merlin), containing two columns of data, (i) locus type and (ii) locus name.   
   Call this file names.txt . For our example data, this is how it looks:

   ```
   A   Trait       
   M   Marker1
   ```
2. Pedigree file:   
   The *pre-makeped* pedigree file format is a relatively simple format with the first 5 columns containing pedigree information followed by genotypes and phenotypes. The first 5 columns are:   
   pedigree-id, individual-id, father, mother, gender.

   Genotypes and phenotypes have to match the order of the loci in the names file. Here, gender is coded as ’1’ = male, and ’2’ = female; the ’Trait’ phenotypes are coded as ’2’ = affected, and ’1’ = unaffected.   
   Call this file pedin.txt . Here is how it looks for our example data:

   ```
   1   1  0  0  2  2  1  2
   1   2  0  0  1  1  1  1 
   1   3  2  1  2  2  1  2
   1   4  2  1  2  1  1  1
   1   5  2  1  1  2  1  2
   ```

   The columns in this file are Pedigree, Person, Father, Mother, Gender, Trait phenotype, Marker1 allele 1, and Marker1 allele 2.

   Note: the *pre-makeped* pedigree file above is exactly the same layout as a PLINK PED file; but while the *pre-makeped* pedigree file may have any number of traits, the PED file may have **only one** trait.
3. Map file:   
   This is a simple tabular format with three columns of data: chromosome number, position in centiMorgans and marker name. The first line is a header line and should be something like "Chromosome Haldane Name" (case is not relevant).

   Call this file “map.txt”. Note that the positions will be read in as Haldane centiMorgans. Our map file looks like this:

   ```
   Chromosome   Haldane  Name
     1          1.0      Marker1
   ```

   The second column heading decides whether the map function is Haldane or Kosambi.

   Convert to Mega2 format files: Use the l2a.py utility to convert the three files you created as illustrated below:

   l2a.py -p pedin.txt -d names.txt -m map.txt -x 01

   \endverbatim

   Three new files are created in the Mega2 format, pedin.01, names.01 and map.01. These look like the three files above, except that they all have header lines with columns names, e.g. the pedigree file has these column names **"Pedigree"**, **"ID"**, **"Father"**, **"Mother"**, **"Sex"**, **"Trait.A"**, **"Marker1.M.1"**, **"Marker2.M.2"**.
4. Run mega2

From within a Unix (or Cygwin) shell, at the Unix prompt, type the command: mega2 \endverbatim

Assuming that the *Mega2* program has been installed according to the installation instructions, you should now see the first menu, like so:

```
==========================================================
              Mega2 4.7.1 input menu:
==========================================================
0) Done with this menu - please proceed
 1) Select input file format:                   Mega2 format with header
 2) Input file suffix:                          01
 3) Locus file:       (Mega2 names.) [required] _
 4) Pedigree file:    (Mega2 pedin.) [required] _
 5) Map file:         (Mega2 map.)   [required] _
 6) Omit file:        (Mega2 omit.)  [optional] _
 7) Frequency file:   (Mega2 freq.)  [optional] _
 8) Penetrance file:  (Mega2 pen.)   [optional] _
 9) Output Directory:                           [ Current directory ]
10) Simulate genotyping errors:                 [ no ]
11) Include all pedigrees whether typed or not 
12) Maximum number of alleles per marker:       2 alleles
Select from options 0-12 >
```

This input menu displays the names of these three input files in the appropriate items (e.g., items 3, 4, and 5). When you are done making all your choices within this menu, to proceed to the next menu, choose item 0 “Done with this menu - please proceed”. Simply follow the instructions provided by this and subsequent menus to continue with the Mega2 analysis.

Note that option 1 allows one to instruct Mega2 to read PLINK format input files, either in binary format or in PED format, or Variant Call Format (VCF) input files in either binary (.bcf), compressed (.vcf.gz), or text (.vcf) format.

\hypertargettips

## 4.2 Tips on more complex data

Specifying allele-frequencies:   
If you have allele frequencies available and wish to use these instead of letting Mega2 estimate them from the pedigree data, then you can set up a frequency file. The Mega2 format frequency file has three columns and looks like:

```
  Name      Allele  Frequency
  Marker1   1       0.2
  Marker1   2       0.8
```

It is not necessary for the trait locus to be included, but you may do so if you wish. If you name this file "frequency.01", then it is discovered automatically by Mega2 as a an input frequency file (and displayed in item 6 of the menu above). \hypertargetcaution

This frequency file, as well as the other optional Mega2 files: the omit file and the penetrance file, may also be provided for PLINK format input. The files follow the exact same format as is used for the Mega2 input format.

## 4.3 Word of caution regarding input file names

For Macintosh OS X users:   
Be careful when you select the common extension **.dat** for your input files. If your map file is named **map.dat** , and you create SimWalk2 files and then run SimWalk2, the **map.dat** file will be overwritten by the SimWalk2 formatted file **MAP.DAT**. The default Macintosh file system is case-insensitive, so it would treat **map.dat** and **MAP.DAT** as the same file.

---

# 5 Citing Mega2

If you use Mega2 as part of a published work, please remember to reference Mega2. You may reference it by citing the following:

> Mukhopadhyay N, Almasy L, Schroeder M, Mulvihill WP, Weeks DE (2005)  
> Mega2: data-handling for facilitating genetic linkage and association analyses.   
> Bioinformatics. 2005 May 15;21(10):2556-7.   
> PMID: 15746282

as well as citing the web site and the version that you used. For example, the web site citation for version 4.7.1 should be:

> Baron RV, Kollar CP, Mukhopadhyay N, Almasy L, Schroeder M, Mulvihill WP, Weeks DE (2013) Mega2 (Version 4.7.1). https://watson.hgen.pitt.edu

If you use Mega2 to convert VCF or BCF input files, then, as this makes use of a built-in copy of VCFtools, you should also cite the following paper:

> The Variant Call Format and VCFtools, Petr Danecek, Adam Auton, Goncalo Abecasis, Cornelis A. Albers, Eric Banks, Mark A. DePristo, Robert Handsaker, Gerton Lunter, Gabor Marth, Stephen T. Sherry, Gilean McVean, Richard Durbin and 1000 Genomes Project Analysis Group, Bioinformatics (2011) 27 (15): 2156-2158.

# 6 Support, bug reports, and feedback

## 6.1 Mega2 feedback

As we strive to improve Mega2, we would greatly appreciate your feedback. This page provides a link to our Mega2 feedback form:

https://watson.hgen.pitt.edu/docs/mega2\_html/Mega2\_feedback.html

Or feel free to send us an e-mail (contact information below).

## 6.2 Mega2 Google Group

Mega2 users are invited to participate in our Mega2 Google Group at https://groups.google.com/forum/#!forum/mega2-users. Support questions should be posted there.

## 6.3 Bug reports

If you encounter a bug in Mega2, please send us a detailed bug report, including the following information:

- Program Name: Mega2
- Program Version: [Mega2 prints out a version number when you first start it up.]
- Platform: [Windows, Macintosh, Linux, etc.]
- Description:
- Example files: [If you can, please send us example files that will allow us to try to re-create the bug on our own computers.]

Please send your bug reports and feedback to: Daniel E. Weeks at weeks@pitt.edu

# 7 Contact information

```
Daniel E. Weeks, Ph.D.        
Professor of Human Genetics and Biostatistics 
Department of Human Genetics
Graduate School of Public Health   
University of Pittsburgh        
Crabtree Hall, Room A303     
130 DeSoto Street             
Pittsburgh, PA 15261          
USA                         
                              
1 412 624-5388              
FAX: 1 412 624-3020         
E-mail: weeks@pitt.edu 
Web page: https://watson.hgen.pitt.edu
```

# 8 Installation

INSTALLATION INSTRUCTIONS FOR MEGA2 VERSION 4.7.1 AND HIGHER

## 8.1 Download Instructions

Mega2 can be downloaded from:

```
https://watson.hgen.pitt.edu/register
```

The latest version is 4.7.1

1. Download the Mega2 distribution package:

   ```
   mega2_v4.7.1_src.tar.gz
   ```
2. Uncompress the package using the "gunzip" Unix command. For example:

   ```
   gunzip mega2_v4.7.1_src.tar.gz
   ```
3. Untar the result using the "tar" Unix command:

   ```
   tar xvf mega2_v4.7.1_src.tar
   ```

   NOTE: You may combine the above two steps via a single command:

   tar xzvf mega2\_v4.7.1\_src.tar.gz

   The "z" instructs tar that it is a compressed archive. This is works for GNU-tar.
4. At this point, you should see a new folder named **mega2\_v4.7.1\_src**. This is the "distribution folder" which contains all the necessary components of Mega2.

### 8.1.1 Mega2 Bitbucket repository

You can obtain, using git, the latest development snapshot of the code from our Mega2 Bitbucket repository at https://bitbucket.org/dweeks/mega2

The development snapshot is not as thoroughly tested as our stable release version available above, but will contain the newest features and changes.

## 8.2 Prerequisites for Mega2

There are installation details specific to each particular platform that are covered in the platform specific sections below. But the common prerequisites will be described in this section.

Before you attempt to install and run Mega2, you should already have Perl, Python, R, awk or GNU-awk, bash and csh (or tcsh), and installed on your computer. All of the above except R are usually present on a typical Unix computer or within the Cygwin (www.cygwin.com) environment.

The R statistical package is used by Mega2’s Hardy-Weinberg equilibrium estimation options, as well as for plotting non-parametric linkage and variance-components LOD scores from the output of Merlin, LOD scores of Allegro and non-parametric linkage P-values from SimWalk2, as well as the Merlin/Simwalk2 combined analysis.

### 8.2.1 R

R can be downloaded from the R distribution site

```
http://cran.r-project.org/
```

For installation instructions, see the R-documentation at:

```
http://cran.r-project.org/doc/manuals/R-admin.html
```

### 8.2.2 R Libraries

Genetics package

To use the HWE options, you need to obtain and install separately the R "genetics" library which provides functions to perform population-genetics related analyses.

The genetics package requires these other packages:

```
combinat, gdata, gtools, MASS, mvtnorm
```

The nplplot R package

To use the plotting option, you need the "nplplot" R library (version 4.5 or greater), also distributed from CRAN. The Mega2 install.sh script now checks to see if you have the proper version installed.

To install these packages, open up R, go to the "Package manager" menu item, and use the "install from the web" option. To load in these libraries, you can again use the package manager menu.

Or alternatively at the command line, start up R, and then issue the R command:

```
install.packages(c("genetics", "nplplot"), dependencies=T)
```

You should be able to see the above libraries in the list of installed libraries.

## 8.3 Installing Mega2 from a Binary Package

### 8.3.1 Contents of a binary package

If you downloaded and unpacked the mega2\_v4.7.1\_src.tar.gz bundle following the download section (8.1↑), you should see a directory named mega2\_v4.7.1\_src containing the following directories (in bold) or files listed in alphabetical order:

> |  |  |
> | --- | --- |
> | Directory (bold) or file | Contents |
> | **developer\_documentation** | Directory containing documentation describing the technical details involved with adding a new output target to Mega2. |
> | **example** | Directory containing example data and batch files. The MEGA2.BATCH.post creates output files for the Cranefoot option and the other MEGA2.BATCH.\* files create output files for the Mendel option. The last four batch files below: MEGA2.BATCH\_pre, MEGA2.BATCH\_ped, MEGA2.BATCH\_bed, and MEGA2.BATCH\_preannotated, run on the equivalent pre-makeped input files and will produce equivalent results. |
> | **example\_output\_post** | Folder containing output files created by running mega2 on the \*.ex files using the MEGA2.BATCH.post batch file. |
> | **example\_output\_annotated** | Folder containing output files created by running with mega2 input files, using the batch file MEGA2.BATCH.annotated. |
> | **example\_output\_pre** | Folder containing output files created by running mega2 on chromosome 5 data using the MEGA2.BATCH.pre batch file from the example folder. |
> | **example\_output\_ped** | Folder containing output files created by running mega2 with PLINK PED input files, using the batch file MEGA2.BATCH.ped. |
> | **example\_output\_bed** | Folder containing output files created by running mega2 with PLINK binary input files, using the batch file MEGA2.BATCH.bed. |
> | **example\_output\_preannotated** | Folder containing output files created by running mega2 with mega2 input files, using the batch file MEGA2.BATCH.preannotated. |
> | install.sh | Bash script for installing Perl scripts and Mega2. |
> | LICENSE.txt | License agreement. |
> | l2a.py.src | Python script linkage\_to\_annotated.py to convert linkage-style format files to the new Mega2 format. |
> | make\_gen\_table.pl.src | Perl source code to reformat the output of the GEN program to a table. |
> | make\_hwe\_table.pl.src | Perl source code to reformat the output of the HWE program to a table. |
> | **map\_making\_utils** | Folder of awk scripts for creating the old Mega2 format map files. |
> | mega2log2html.pl.src | Perl source to create html-formatted Mega2 log and summary files. |
> | **mega2\_bin** | **Pre-compiled binaries for some platforms.** |
> | **mega2\_html** | PDF and html documentation pages for Mega2 |
> | merlin2sw2.pl.src | Perl source for feeding Merlin output into SimWalk2 for the Merlin-SimWalk2 combined option. |
> | Rallegro.pl.src Rmerlin.pl.src Rsimwalk2.pl.src | Perl source files for reformatting Allegro, Merlin and SimWalk2 linkage-analysis output for plotting by nplplot. |
> | **srcdir** | **Mega2 source code** |

### 8.3.2 Mega2 Binaries

In the **mega2\_bin** directory, there will be binary versions of Mega2 for several platforms. These binaries will have a Mega2 version number and a platform version number. Typically, the Mega2 binary references dynamic load libraries (.dll’s or .so’s) for a particular platform/version and will not necessarily work on other versions of the platform. There are separate native binaries for Microsoft Windows 7 vs Microsoft Windows 8. Also, the binaries for MinGW are labeled according to whether they were built on Windows 7 vs Windows 8. (MinGW needs two .dll’s if you wish to install just Mega2 without the whole MinGW. They are included and depend on Windows Version.)

The **install.sh** program (below) determines whether any compiled binaries will work on your platform. (Alternatively, **install.sh** will compile an appropriate binary from the source.

### 8.3.3 Installing Mega2

To install the mega2 executable, Perl scripts and nplplot, from within the Mega2 package folder (i.e. mega2\_v4.7.1\_src in above step), type the command:

```
./install.sh
```

It prompts the user to enter a directory to install the Mega2 components into. Enter the full path name of the desired directory e.g.

```
/usr/local/bin
```

(do not add a trailing backslash!)

The directory entered above must be in the user’s path and you will need write permission to copy files into this directory.

On some platforms, you may need to type ’rehash’ to refresh your search path (this is not necessary on Linux, and the ‘rehash’ command is not a standard Linux command).

Mega2 can now be run on a set of data by simply typing the command "mega2". It should be invoked from within the directory containing the input data files.

## 8.4 Compiling and Installing Mega2 from Source

The mega2\_v4.7.1\_src.tar.gz package also includes a source code folder "srcdir" containing C source files, a Makefile, and several OS-specific Makedefs files that define compilation flags that govern locations of standard C libraries, and other OS-specific behavior.

Please note that GNU-make is required to compile Mega2.

To compile Mega2 follow these steps:

1. From within the mega2\_v4.7.1\_src folder, type

   ```
   ./install.sh
   ```

   to find out whether your OS or OSTYPE variable is recognized. This script attempts to determine the platform using the *uname* command.
2. The script will print an error message if the platform is not included within a pre-defined list, and ask you to create a Makedefs file to describe compilation parameters for your specific platform.
3. Create this file by adapting one of the existing Makedefs files.
4. Run

   ```
   ./install.sh
   ```

   again, and “make all” will be run to compile Mega2. Finally, you will be prompted for the name of an installation path as before. Mega2 will then proceed to install the Mega2 binary and Perl programs correctly.

If you make changes to Mega2’s source code, we would greatly appreciate your informing us of what changes you made and why.

## 8.5 Running Mega2 on Your Data

Familiarize yourself by running Mega2 on the example data provided.

1. cd into the **example/** folder.
2. Check its contents by using the ls command.

   The **example**/ folder contains the following files:

   datain.05

   map.05

   MEGA2.BATCH.post

   datain.ex

   pedin.ex

   map.ex

   omit.ex

   pedin.pre.05

   MEGA2.BATCH.pre

   names.annotated

   frequency.annotated

   penetrance.annotated

   map.annotated

   pedin.annotated

   MEGA2.BATCH.annotated

   names.preannotated

   frequency.annotated

   penetrance.annotated

   map.preannotated

   pedin.preannotated

   MEGA2.BATCH.preannotated

   ped.ped

   ped.map

   ped.phe

   ped.frequency

   ped.penetrance

   MEGA2.BATCH.ped

   bed.fam

   bed.bed

   bed.map

   ped.phe

   ped.frequency

   ped.penetrance

   MEGA2.BATCH.bed

These constitute several sets of the preexisting Mega2 format data files, namely the ".05" data set and the ".ex" data set, as well as a data set in the Mega2 format. The "pedin.pre" file is analogous to the "pedin.05", except that it is in pre-makeped format. The \*.ex set also includes an omit file to illustrate the usage of the "omit" file. The pedin.pre.05 file is a pre-Makeped format file. It uses the datain.05 locus data file. The Mega2 data set was created by running the python script linkage\_to\_annotated.py on the ".05" data set.

1. Run mega2, by simply issuing the command mega2 without any arguments, or you may run it using one of the batch files:

   ```
   mega2 MEGA.BATCH.pre
   ```

   Your own data can be run exactly as above; simply change into your data folder, and run Mega2.
2. Example Output:

   The example\_output\_pre, example\_output\_post, example\_output\_annotated, example\_output\_ped, example\_output\_bed and example\_output\_preannotated directories contain output from Mega2 when run using the MEGA2.BATCH.pre, MEGA2.BATCH.post, MEGA2.BATCH.annotated, MEGA2.BATCH.ped, MEGA2.BATCH.bed and MEGA2.BATCH.preannotated batch files in the example/ directory.

## 8.6 Mega2 Documentation

The sub-folder called mega2\_html/ within the mega2\_v4.7.1\_src folder contains documentation for mega2. The Mega2 documentation is also available on-line at

```
https://watson.hgen.pitt.edu/docs/mega2_html/
```

## 8.7 License Agreement

Distribution of Mega2 is governed by the terms of GNU General Public License Version 3 (GPLv3) 30.1↓.

Mega2 uses two libraries developed by other external groups, the sources of which are included with the Mega2 sources. Minimal modifications have been made to these two libraries to facilitate their integration with Mega2. The VCFtools library handles reading of the various Variant Call Format (VCF) input files. ZLIB is needed by the VCFtools library and is also used to format Beagle output. The VCFtools library is released under the GNU Lesser General Public License Version 3 (LGPLv3) 30.4↓, and the MIT license 30.2↓. The license for ZLIB is shown in 30.3↓.

## 8.8 Feedback and Bug Reports

If any of these instructions are not clear, please let us know via our feedback form by following the "Feedback/Bug report" link on the following page:

```
https://watson.hgen.pitt.edu/register/
```

You may also use this feedback form to send us comments and bug reports on Mega2.

## 8.9 Macintosh-specific installation issues

### 8.9.1 Introduction to Command line programs for Mac users

You will need to familiarize yourself with using the Macintosh OS terminal, as installing and using Mega2 is mainly done within a Unix terminal.

Unix organization conventions

The "Users" folder contains sub-folders with user names matching those that have user-accounts on your computer. Each user has full access and modification permissions to his or her /home/user\_name folder.

All other folders can only be written or modified by the administrator. The /bin and /sbin folder contains system programs that are very basic to Unix (such as ls, cd, rm, mkdir, bash, tcsh etc.).

The /usr folder hierarchy is used to install programs that should be made available to all users. Widely-used standard Unix software such as compilers and language development tools (gcc, Perl, Python) are directly installed inside /usr. Thus to run Perl from inside a terminal, you would invoke /usr/bin/perl.

There is also a /usr/local folder hierarchy inside the /usr folder which is normally used to install lesser known or non-standard software, of which Mega2 would be an example. Thus, you may choose to install your genetic analysis software (such as Merlin, SimWalk2 etc.) inside the /usr/local/bin folder.

The Mac OS environment

When you open up a Mac terminal window, you can use Unix commands such as ls (list folder contents), cd (change directory) etc. The default folder associated with such a window (i.e. before you issue a a cd command), is your "home" folder, generally named */Users/your-user-name.* Your Desktop folder is called */Users/your-user-name/Desktop*.

Command line version of R

R is required to use Mega2’s Hardy-Weinberg equilibrium estimation options, as well as for plotting LOD scores from the output of Merlin, SimWalk2, and Allegro. Therefore, for full functionality, you need to obtain and install the R software. The command line version of R is identical to R as used on other Unix operating systems. Therefore general documentation for R applies to this version as well. On each release (and patched-release), binaries are distributed through CRAN. These binaries come with a common installer used by R.app so please read the related notes (see How to get R.app). To use R, you probably need to add a symbolic link on your system as the R binary is located inside the MAC “framework” hierarchy. Suppose you have the /usr/local/bin directory on your system (if you do not have one, you can use /usr/bin instead), you should just type in your Terminal (a root password is required)

```
sudo ln -s /Library/Frameworks/R.framework/Resources/R /usr/local/bin/R
```

Assuming that you have /usr/local/bin in your PATH environment variable, you will be able to launch R from any location on your system just by typing R. In this way, when you install a new version of the R.framework this link will point to the latest R binary.

### 8.9.2 Xcode compilation environment

Xcode is free software, and contains development tools including "make" and "gcc", both of which are required to compile and install Mega2. Xcode is not typically installed by default, but is available on one of the Mac OS system install DVDs that came with your computer. Xcode is also available as a free download from:

```
http://developer.apple.com/technologies/tools/xcode.html
```

You need to be a registered Apple user to download Xcode.

## 8.10 Unix-specific installation issues

This release of Mega2 has been tested on several versions of Ubuntu Linux. It should work on other Unixes as well. The supplied binaries are for Ubuntu 10.04.2 both 32 and 64 bit platforms as well as for Ubuntu 11.04 both 32 and 64 bit platforms. Be careful to run the appropriate binary on the matching platform.

If you discover errors, please report them so we can improve our port.

Note: The current version of mega2 has some C++ files. If you compile mega2 from source, make sure that your system includes a C++ compiler.

## 8.11 Windows Cygwin installation

### 8.11.1 Cygwin POSIX windows environment

Cygwin(www.cygwin.com) provides a rather complete Posix Unix environment running on top of a Windows platform. An interface layer lets traditional Unix programs be compiled and interface with Windows services. Thus all of the Unix programs can be made available.

If you are planning to use the Cygwin environment extensively to run Mega2 and other programs, you should familiarize yourself with the Unix system in general; see 3↑ (above).

Before you attempt to install and run Mega2, Cygwin should already have been installed on your Windows machine.

Installing Cygwin

Download and install Cygwin from http://cygwin.com, the Cygwin distribution site.

IMPORTANT: the cygwin version should be 1.7.9-1 or higher to be able to run Mega2.

Run

```
setup.exe
```

Click on either the "install" or "install/update" link and follow the instructions.

The cygwin user’s guide can be found at:

```
http://cygwin.com/cygwin-ug-net/cygwin-ug-net.html
```

If your Windows machine has multiple user-accounts, you should install cygwin when logged in as "administrator". Also, select the "All users" default option to make Cygwin available to all users.

You should do a custom install of Cygwin, and make sure that the following are selected/installed:

1. Perl (top level; click on ’Default’ to change it to ’Install’)
2. tcsh and bash (inside "Shells"; bash is already selected for installation)
3. GNU C libraries (inside "Devel"; libgcc1 is already selected for installation)
4. gcc compiler (inside “Devel”; choose the version 4 compiler gcc4-core)
5. gdb debugger (inside “Devel”; just in case)
6. make (inside "Devel", named "make: The GNU version of the ’make’ utility"’)
7. Python (top level).
8. An editor such as nano, or vim (inside "Editors")
9. The gawk interpreter (inside "Interpreters")
10. Finally, R (inside “Interpreters”) can be installed if the pre-compiled versions available are new enough for your purposes.

Click on the check box next to each of these items, and change the "default" to "install".

In the newest version of Cygwin, many of these are set to install by default, however you should verify this. If you do forget to install any of these components, you may update the existing installation rerunning the same setup script "setup.exe".

The Cygwin environment

Cygwin provides a Unix-like environment inside the Windows operating system. During the installation process it will prompt you for an installation path. Let us assume that you choose to install it on the Desktop. Then you will see a folder called Cygwin on the Desktop, which contains folders named "home", "bin", "etc", "tmp", "sbin", "usr" etc.

An icon is created on the Desktop for running the Cygwin program. When you click on this icon, Cygwin starts an interactive shell (resembling the Windows DOS shell), and puts you inside the /home/your\_user\_name folder.

Accessing data

Files within Cygwin folders from the Windows can be opened using the Windows file explorer. All cygwin files and folders are kept within the Cygwin installation folder (in our example: Desktop/Cygwin).

To access Windows files from inside of Cygwin, note that relative to Cygwin, all Windows drives can be accessed by using the ’/cygdrive/’ path prefix. For example, to list files and folders on your C drive, do this:

```
ls /cygdrive/c
```

to list the contents of the directory C:

(Inside Unix, you are always required to enclose names inside quotes if they have white-spaces inside them.)

Introduction to command line programs for Windows

The "home" folder contains sub-folders with user names matching those that have user-accounts on your Windows machine. Each user has full access and modification permissions to his or her /home/user\_name folder.

All other folders can only be written or modified by the administrator. The /bin and /sbin folder contains system programs that are very basic to Unix (such as ls, cd, rm, mkdir, bash, tcsh etc.).

The /usr folder hierarchy is used to install programs that should be made available to all users. Widely-used standard Unix software such as compilers and language development tools (gcc, Perl, Python) are directly installed inside /usr. Thus to run Perl from inside Cygwin, you would invoke /usr/bin/perl.

There is also a /usr/local folder hierarchy inside the /usr folder which is normally used to install lesser known or non-standard software, of which Mega2 would be an example. Thus, you may choose to install your genetic analysis software (such as Merlin, SimWalk2 etc.) inside the /usr/local/bin folder.

### 8.11.2 R installation:

R is required to use Mega2’s Hardy-Weinberg equilibrium estimation options, as well as for plotting LOD scores from the output of Merlin, SimWalk2, and Allegro. You can follow the earlier instructions and include R from the Cygwin repository.

Alternatively, the windows version of R can be downloaded from the R distribution site http://cran.r-project.org/. By default R will install into a folder named R inside the Program Files folder. Each version of R installs into a separate sub-folder inside R, e.g., if you installed version 2.13.0, you should have a folder

```
c:Program Files/R/R-2.13.0/
```

The R executable (.exe) file is named R.exe and resides inside R-2.13.0/bin.

To make sure that Cygwin knows where to find R, start up Cygwin as administrator, change directory into /usr/bin, and create an alias to this R-executable using the following command:

> ln -s /cygdrive/c/Program\ Files/R/R-2.13.0/bin/R.exe R

### 8.11.3 Mega2 Download instructions for Windows

Mega2 can be downloaded from:

```
https://watson.hgen.pitt.edu/register
```

You need to have registered your e-mail before you can download software at this site.

1. The Mega2 distribution package is named mega2\_v4.7.1\_src.tar.gz. Download this package into your Downloads folder.
2. Depending on your browser settings, Windows may automatically untar the file, in which case you will see this folder on your desktop instead:

   ```
   mega2_v4.7.1_src.tar
   ```
3. Now please follow the common installation instructions in section 8.1↑.
4. Note: In some cases, your file may be downloaded as:

   ```
   mega2_v4.7.1_src.tar.tar
   ```

   instead of as:

   ```
   mega2_v4.7.1_src.tar.gz
   ```

   If that happens, then rename the “tar.tar” file as follows:

   ```
   mv mega2_v4.7.1_src.tar.tar mega2_v4.7.1_src.tar.gz
   ```

   and then follow the common installation instructions in section 8.1↑.

## 8.12 Windows Mingw installation

### 8.12.1 Mingw POSIX windows environment

Mingw (www.mingw.org) provides a **min**imal **G**NU environment running on top of a **W**indows platform. The environment is just sufficient to support the traditional gcc C language compiler and other elements of the tool chain. Some additional programs have been ported as well such as bash. But all the target libraries and services used are supplied by Windows. Thus most Unix programs have to be modified to compile and link in this environment. Still, these programs will run faster than the equivalent programs available under Cygwin which has an interface library between the programs and the Windows services.

Before you attempt to install and run Mega2, Mingw should already have been installed on your Windows machine.

Installing Mingw

Browse the Mingw page

```
http://www.mingw.org/wiki/Getting_Started
```

on the Mingw distribution site. Click on the link

```
mingw_get_inst
```

to get the interactive loader. Select g++ and in addition select the development environment. After a few minutes of downloading, mingw and MSYS (the development environment programs) will be set up on you machine. To allow easy access to all of the programs, add

```
c:\mingw\bin
```

and

```
c:\mingw\msys\1.0\bin
```

to your path.

Building Mega2

You should follow the previous instructions for downloading Mega2 on Windows (8.11.3↑) and then just build Mega2. The instructions for compiling were given in section 8.4↑.

Using Mega2

Following the previous recipe, you will have a Mega2 binary program. But as discussed earlier and summarized in section27↓, there are several third party programs that are needed by Mega2. Mingw only provides Perl and Bash. You will have to download Python and R and csh if you need them.

## 8.13 Native Windows installation

### 8.13.1 Native Windows environment

All that is truly necessary to build Mega2 is a compiler and the GNU make program. Microsoft provides a free C/C++ compiler as part of its Visual Studio Express; visit

```
http://www.microsoft.com/visualstudio/en-us/products/2010-editions/express
```

and download the C++ compiler.

Note: You will not be using the IDE just the compiler and linker from visual studio. This means you need to put them on your path before doing the *make* (below). The easiest way to do this is to run the script **vcvars32.bat** in the binary directory of visual studio visual C++ compiler.

It is a bit harder to find a native version of GNU make that is not part of Cygwin or Mingw. The sourceforge project GNU Win32 has it at:

```
http://gnuwin32.sourceforge.net/packages/make.htm
```

You should download GNU make and install it to some place on your path.

Building Mega2

You should follow the previous instructions for downloading Mega2 on Windows (8.11.3↑) and then just build Mega2. Unfortunately, the instructions that were given in section 8.4↑ do not work because **install.sh** depends on the **bash** shell. The work around is to change to the **srcdir** directory and execute

```
make all
```

You will have to copy the Mega2 binary and all the Perl and Python scripts to a directory on your path. The script files end in ***.src***; you should remove this suffix so that Windows can find the right program to run the file.

Using Mega2

Following the previous recipe, you will have a Mega2 binary program. But as discussed earlier and summarized in section 27↓, there are several third party programs that are needed by Mega2. If you need these programs, you will have to load them onto Windows.

# 9 Input file formats

Mega2 supports the following input file formats:

1. Mega2 format (See 9.1↓)
2. Linkage format (See 9.3↓)
   1. Linkage format
   2. Linkage format with Mega2 names file (See 9.3.7↓)
3. PLINK format (See 9.2↓)
   1. PLINK binary format
   2. PLINK PED format
4. Variant Call Format (See 9.4↓)
   1. BCF format
   2. VCF compressed format
   3. VCF format

## 9.1  Mega2 input file formats

Mega2 can read input file sets in the Mega2 format ( referred to in previous documentation as the Annotated format), which consist of files are in table format with a header line defining the names of each data column. Columns are separated by one or more white-space characters (tabs or blanks), and rows are separated by newlines (or returns).

Some columns require special reserved names (these are listed as fixed names below under each file heading), and other column names are specified by the user following our naming convention. Every file has some required columns (e.g. pedigree file always has an ID column), and some files allow optional columns, including extra ones that are ignored by Mega2. A column is read in and ignored if its name contains “X.” as the first two characters. Note that the “#” character at the beginning of the column name also works, but the “X.” convention allows files to be compatible with R.

Data lines below the header column are read in accordance with the column names/types, therefore, there is no special order required for the columns themselves, as is the case for the linkage format files.

The Mega2 distribution package contains a conversion program for converting from the old LINKAGE-based format files into the Mega2 format files (See section 11↓).

Mega2 requires three matched files as its input. These are the locus, pedigree, and map data files. This trio of files can be supplemented by an omit file, for omitting specific data points from all reformatted output files. It is easiest if you give these files their **default names** with the same extension, as then Mega2 will automatically fill in the file names for you when you specify the chromosome number. So, for example, if your files contained information for just chromosome 4 markers, then it is easiest if you name them as follows:

```
  datain.04
  pedin.04
  map.04
  omit.04
```

Mega2 also supports two more optional files: an allele-frequency file, and a penetrance file for affection status traits. The default file names are (e.g. for chromosome 4):

```
  freq.04 
  pen.04
```

Note: *frequency* can be used in place of *freq* and *penetrance* in place of *pen*. In the current version, there is no provision for providing quantitative trait distribution parameters.

**WINDOWS/DOS USERS WARNING**

If you are creating your files on a Windows or DOS system and then transferring them to a Unix machine, please remember to convert the DOS end-of-line characters to Unix end-of-line characters. When run on a Unix machine, Mega2 will detect DOS end-of-line characters and terminate with an error. See the trouble-shooting section 24.5↓ for more details.

### 9.1.1 Mega2 Names file

The two required columns inside a names file are the locus name and the locus type. The required column names are Name and Type. Allowed marker types are the six locus types, as for the QTDT names file, namely autosomal numbered (M), X-linked numbered (X), Y-linked numbered (Y), binary trait with a single liability class (A), binary trait with multiple liability classes (L), quantitative trait (T), and quantitative covariate (C).

|  |  |
| --- | --- |
| **Type code** | **Description** |
| M | autosomal marker |
| X | X-linked marker |
| Y | Y-linked marker |
| T | Quantitative trait |
| C | Quantitative covariate |
| A | Affection status trait (single liability class) |
| L | Affection status trait (multiple liability classes) |

Here is an example names file:

```
Type  Name
A Trait
T Q1
M M1
M M2
M M3
```

### 9.1.2 Mega2 Pedigree file

There are 5 required columns and 9 optional columns with fixed column names, which primarily refer to the pedigree information. The required columns are ID, Pedigree, Father, Mother, and Sex. The optional columns allowed in the pedigree file are: PerID, PedID, MZTwin, DZTwin, Proband, Group, FirstOff, NextMatSib, NextPatSib .

Phenotype and genotype column headers are defined by the user using the following convention: numbered autosomal and x-linked loci genotype column names are named as

**marker-name.marker-type.[1/2]** ,

where marker-name one of the locus names within the names file, marker-type is the corresponding locus type, and the extension 1 or 2 denotes the allele-number. Currently, the allele-number is of no significance with respect to the haplotype, in future, we will consider extending Mega2 to process haplotypes.

Binary traits are given an extension A for the status column header, and L for the liability class header. Traits with a single liability class does not need this liability column. Quantitative traits and covariates have either a Q or a C as the extension.

For an affection trait, the headers should be as follows: If there is a single liability class, then you only need one phenotype column containing the affected status, and the header for this should have the single extension A .

If there are multiple liability classes, then the status column header should have 2 extensions L.1, and the liability class column should have the extensions L.2.

|  |  |
| --- | --- |
| **Extension** | **Description** |
| M | autosomal marker |
| X | X-linked marker |
| Y | Y-linked marker |
| T | Quantitative trait |
| C | Quantitative covariate |
| A | Affection status trait (single liability class) |
| L | Affection status trait (multiple liability classes) |

In version 4.5.7 and later, you can just use M for all the **markers** in the pedigree file header line and in the names file. Mega2 will read the map file to determine whether the marker is on a X-linked or Y-linked chromosome.

Using non-numeric allele names

Marker genotypes are coded as a pair of alleles, with at least one space or tab between them. Non-numeric allele names are allowed inside the pedigree file only with the use of a names file, and only for numbered marker loci. In releases of Mega2 starting with 4.7.1, non-numeric allele names when specified will be copied forward from input files to output files for analysis types that allow non-numeric alleles. Carrying the original allele labels through to the output files without recoding makes it easier to keep track of which allele is which. The analysis options which support non-numeric allele names are: Beagle, Cranefoot, IQLS/Idcoefs, Loki, Mega2, Mendel, Merlin, Merlin-SimWalk2, PLINK, SAGE, SAGE4.0, SOLAR, SPLINK, and SimWalk2. For the other analyses, the recoded output pedigrees will have their genotypes altered to have numeric alleles. Also, in the case where the input contains numeric alleles that are not contiguous (e.g., microsatellites) they will be treated as if they are allele names and copied. The Mega2 command line flag (*-\/-force\_numeric\_alleles*) can be been specified to force allele recoding, if it is not necessary, but preferred by the researcher. Allele names have to be strings, and may not contain white-space characters, since the pedigree file is read in assuming a white-space delimited column format.

Note: Some analyses, like Merlin, require the letter alleles to be A, C, G or T. The coding convention of using A and B as the allele designator is not accepted. Mega2 has not been programmed to make this check. So for the present, in this and similar analyses, you will have to manually set *-\/-force\_numeric\_alleles.*

Missing values

Missing values are “NA” by default in Mega2 format. In order to be compatible with linkage format data, we have provided a means to specify missing allele and affection codes. Missing quantitative values can be “NA” or a special number such as -99. In a future release, we will also allow non-numeric missing value indicators for quantitative phenotypes.

```
Pedigree ID Father Mother Sex TRAIT.A Q1.T M1.M.1 M1.M.2 M3.M.1 M3.M.2 M2.M.1 M2.M.2 PedID PerID  
 1   1   0   0  1  2    21.2   1  2   1  1   1  2   1  1
 1   2   0   0  2  0     1.3   1  2   2  2   1  1   1  2
 1   3   0   0  1  0     NA   NA NA  NA NA  NA NA   1  3
 1   4   1   2  2  2    19.1   1  2   2  1   1  2   1  4
 1   5   1   2  1  2    18.3   2  2   2  1   1  2   1  5
 1   6   0   0  2  0     0.7   2  2   1  1   1  2   1  6
 1   7   3   4  2  2    20.5   2  2   1  2   2  1   1  7
 1   8   3   4  2  2    22.1   2  2   1  1   2  2   1  8
 1   9   3   4  2  0    11.1   2  2   1  1   2  2   1  9
 1  10   5   6  1  2    19.5   2  2   1  1   2  2   1 10
 1  11   5   6  1  2    17.9   2  2   1  2   1  2   1 11
```

### 9.1.3 Mega2 Map file

The map file requires 3 mandatory columns, including at least one set of genetic map positions. Multiple versions of the genetic map can be provided using additional columns, as well as physical position on each chromosome in base-pairs. Mega2 allows users to choose between multiple maps. Each genetic map can include sex-specific maps. Details on specifying male and female distances are provided below.

See the Genetic Map Interpolator section 10↓ for help making map files.

The chromosome number column should be named ’Chromosome’ (case-sensitive) and the marker name column should have the heading ’Name’. Map position columns are identified by user-defined names of the format *map-name.map-type.sex* for genetic maps. Map-type can be either ’k’ or ’h’, denoting Kosambi or Haldane centiMorgans respectively. Sex can be ’a’, ’m’ or ’f’ denoting sex-averaged, male, and female maps, respectively.

Physical maps columns should be named as map-name.p, where the ’map-name’ is user-defined, and the ’p’ stands for the physical map type.

Below is an example Mega2 map file, with two maps, one genetic and one physical:

```
Chromosome    Map.k.a  Name   Map.k.m    Map.k.f  Buildxx.p
5             0.0       M1      0.0        0.0    144255
5             5.0       M3      2.0        7.0    144355
5             8.0       M2      4.0       12.0    144400
```

Shown below is a more complicated Mega2 map file containing four maps, two genetic (M and M4) and two physical (M2 and M3):

```
Chromosome M.h.a    Name   M.h.m   M2.p    M.h.f  M3.p   M4.k.a   M4.k.m
1          0.0     rs101   0.0     543     0.0    304    0.0       0.0 
1          5.0     rs102   2.0     678     7.0    821    5.0       2.0 
X          0.0     rs231   0.0     743     0.0    912    0.0       0.0 
X          4.0     rs232   1.0     862     6.0    954    4.0       0.0
```

For annotated map files, acceptable chromosome labels include:

|  |  |
| --- | --- |
| **Chromosome code** | **Description** |
| X | X chromosome |
| Y | Y chromosome |
| XY | Pseudo-autosomal |
| MT | Mitochondrial |
| U | Unknown chromosome |
| 1 to 22 | Autosomal chromosome |

Mapping used by l2a.py when converting chromosome numbers into more human-readable chromosome codes:

|  |  |
| --- | --- |
| **Chromosome number** | **Chromosome code** |
| 23 | X |
| 24 | Y |
| 25 | XY |
| 26 | MT |
| 999 | U |

**Important Note**: Before release 4.5.5, Mega2 used the mapping table below with number 25 coding for Y, and 24 coding for XY. But as of version 4.5.5, this coding has been changed to be compatible with PLINK.

Table of the previous coding system (version 4.5.4 and earlier):

|  |  |
| --- | --- |
| **Chromosome number** | **Chromosome code** |
| 23 | X |
| 24 | XY |
| 25 | Y |
| 26 | MT |
| 999 | U |

**Special chromosome names and numbers**

Note: We have not defined the **simple map file format** yet. It is used with LINKAGE style input files and it is not as flexible as the **map file** we have described here. It provides a single set of map information. See section 9.3.4↓.

**X chromosome**   
 **map file** - “X” denotes x-linked loci in the map file.   
**simple map file** - “23” should be used as the chromosome label to denote X-linked loci in the simple map file.   
  
Females can be heterozygous at X-linked markers, whereas males should be assigned homozygous genotypes. Males’ genotypes are then analyzed as being hemizygous by Mega2 and the other genetic analysis software that can handle X-linked loci.

Mega2 asks the user if loci on chromosome 23 (in simple map input files) should be considered as X-linked or autosomal.

**Pseudo-X region of X and Y chromosomes**   
 **map file** - Some genes are common to both X and Y chromosomes in humans. These genes are said to reside in the pseudo-autosomal region. The chromosome labels should be set to “XY”.   
  
 **simple map file** - In simple map files, such markers should be labeled with chromosome number “25”.   
Loci labeled as pseudo-autosomal are treated as autosomal loci. (Before Mega2 4.5.5 this used to be “24”.)

**Y chromosome**   
 **map file** - “Y” should be used as the chromosome label for markers that are to be treated as Y-linked.   
 **simple map file** - “24” should be the chromosome number inside the simple map file. (Before Mega2 4.5.5 this used to be “25”.)  
  
Note that most analysis software listed inside our output options do not handle Y-linked loci. We have included this facility so that the user can check and clean data for Y-linked loci as well as generate allele-frequency and genotyping summaries.

Females’ genotypes for Y-linked loci are ignored for now, and males are expected to be homozygous at these loci. In future, we will provide counts on “putative” female genotypes at Y-linked loci, in order to flag data errors.

**Mitochondrial chromosome**   
 **map file** - “MT” should be used as the chromosome label for markers that are to be treated as linked to the mitochondrial chromosomes.   
 **simple map file** - “26” should be the chromosome number for mitochondrial markers.   
  
Although an individual is expected to be homozygous at mitochondrial markers, and also have inherited the mother’s genotype exclusively, there have been known to be deviations from this inheritance pattern. Therefore, for the present, Mega2 only reports genotypes which are heterozygous, or different from the maternal genotype, it does not flag these as Mendelian inheritance errors, nor are these reset along with other Mendelian inconsistencies.

**Unknown chromosome**   
If the input data contains unmapped markers, some limited analysis is still possible on such markers, which do not need map information. These include the allele-frequency estimation and data-checking/cleaning steps of Mega2, as well as marker-related summaries. Some output options do not need map information. These are: SUMMARY options, PLINK (using a map file with dummy positions), the HWE options, which do not need a genetic map, LINKAGE, NUCLEAR PEDIGREES, PREMAKEPED-FORMAT, and PREST.   
  
 **map file** - Use the label “U” inside the map file to label unmapped markers.   
 **simple map file** - Use “999” to label unmapped markers inside the simple map file.

**Output file labels**   
If input files were in Mega2 format with chromosomes labeled with X,Y etc. the output files also have character extensions instead of chromosome numbers (e.g. Lpedin.X, Ldatain.X etc.). If input files were in simple map format, numeric extensions are used, 23-25 and 999 for the unknown chromosome.

**New reordering menu items**  
For analysis menu options that can handle unmapped loci, you will be able to select the unknown chromosome as well as the usual numbered chromosomes if your map file contains such loci. The menu option which allows selection of multiple chromosomes for output now offers 3 choices:  
1) All known chromosomes.   
2) Specific known chromosomes.   
3) All mapped and unmapped loci.

### 9.1.4 Omit file

The omit file contains the three required columns. Again, names must be maintained, ordering of columns doesn’t matter, and neither does the case in the header line.

```
Pedigree  Individual  Marker
1 All All
2 10  M2
2 All M1
```

The optional omit file permits one to easily delete certain marker genotypes from all Mega2-generated output files. This is useful if certain marker genotypes are Mendelianly-inconsistent, yet one wants to preserve the original marker data in the input file. Marker genotypes can be omitted for a whole family at once or for one specific individual.

The omit file should be in the following format:

Each line should have two integers and a string, separated by white space. The first number is the pedigree number and must match that used in your input LINKAGE format file. The second number is the person number and must match that used in your input LINKAGE format file. The string should be either All or the name of the locus. If All is used, the person or pedigree indicated will be untyped at all marker loci. Trait phenotypes will not be set to unknown. Specific omit file entries containing the name of the trait locus should be included to do the latter. If the person number is zero or all, then all marker genotypes will be set to unknown for the entire pedigree. Otherwise only the indicated person will be untyped.

A summary of the omit results will be found in the file omit.log. This file is rewritten the next time Mega2 is run with an omit file specified. If Mega2 can not find a person or pedigree as specified in the input omit file, it will halt with an error message.

Example omit file, ’omit.05’:

```
Pedigree  Individual  Marker
1 0 All
2 10 M2
2 0  M1
```

This file generates the following log file:

```
Marker untyped everyone in pedigree 1
Marker untyped pedigree 2 person 10 at locus M2
Marker untyped everyone in pedigree 2 at locus M1
```

The omit file can now be used to set trait phenotypes to unknown. Here is such an example:

```
Pedigree  Individual  Marker
1 11 AFF2
1 11 QUANT1
```

These two lines direct Mega2 to untype person 11 of pedigree 1 at the affection locus AFF2 and at the QTL trait QUANT1. The affection status will be set to unknown (0), and the quantitative phenotype will be set to the appropriate missing value in the output. These actions are logged as well.

Please note that when the marker column contains the keyword “All”, it still refers to only marker loci, trait loci are left untouched.

Hint: See the section 26.3↓ on creating omit files based on errors found by running the pedigree checking program Pedcheck.

### 9.1.5 Frequency file

The frequency file contains one line for each marker and allele. All alleles present in the data have to be listed. The allele labels should match those in the pedigree file (raw or coded). The column headings should be as listed below (letter case and order of columns don’t matter).

```
Name Allele Frequency
TRAIT 1 0.990000
TRAIT 2 0.010000
Q1 1 0.990000
Q1 2 0.010000
M1 1 0.500000
M1 2 0.500000
M3 1 0.250000
M3 2 0.250000
M3 3 0.250000
M3 4 0.250000
M2 1 0.500000
M2 2 0.500000
```

It is not necessary to have all your loci listed within the frequency file, however, if a locus is included, it is necessary to list ALL its alleles. If this is not so, then alleles encountered in the genotype data, and not specified inside the frequency file will be flagged as errors. Locus names listed inside the frequency file, but missing from the names file will cause Mega2 to terminate with error messages.

For marker loci whose allele frequencies are not listed, Mega2 estimates them, as well as recoding them to consecutive integer values. For trait loci, Mega2 accepts only 2 alleles. If trait allele frequencies are not provided, then Mega2 uses default values of 0.5 for both alleles.

### 9.1.6 Penetrance file

Affection status traits are listed in the penetrance file. This file should contain 5 columns listing the name class number and penetrance values for the three genotypes in each class. As in the names file and frequency file a header is required; the column headers should be as specified below (case is ignored, and there is no special ordering).

```
Name   Class  Pen.11    Pen.12    Pen.22
TRAIT 1 0.0000 1.0000 1.0000
```

With the above header, the penetrances are assumed to be autosomal. An additional column, “Type”, may be added to the header with row value of *autosomal* for autosomal chromosomes or with the values, *female*, or *male* to indicate the penetrance values for the particular population on the X chromosome.

```
Name   Class  Pen.11    Pen.12    Pen.22 Type
TRAIT 1 0.1000 1.0000 1.0000 autosomal
TRAIT 1 0.1500 1.0000 1.0000 female
TRAIT 1 0.0500 1.0000 1.0000 male
```

NOTE: Every row must have 6 column, even though there is no meaningful value for Pen.12 for males. Whatever value is put in the Pen.12 column is ignored.

## 9.2  PLINK input file formats

The PLINK program (http://pngu.mgh.harvard.edu/~purcell/plink/) written by Shaun Purcell and colleagues has become a *de facto* standard for whole genome association analysis. It uses input files that contain the same kind of information as the Mega2 format. It also allows for several different formats of the input data and supports conversion between these formats. Perhaps the most popular (but unreadable) format is its compressed binary PED format. Mega2 supports the binary format and one of the text formats, PED. We suggest you visit the PLINK documentation web site (http://pngu.mgh.harvard.edu/~purcell/plink/) to understand these formats.

Our desire is to have Mega2 accept the PLINK files unmodified. One “feature” of the PLINK file format is that some of the columns in the files listed below may be omitted by using a corresponding flag when starting PLINK. Mega2 will accept most of those flags and interpret them as the PLINK program would. The flags are explained below. How to enter these flags is explained in section 39↓.

### 9.2.1 PLINK PED input format

This format is typically coded using two primary files: the pedigree file and the map file. The default naming scheme is to use the same stem for both, and a ’.ped’ extension for the pedigree file, and a ’.map’ extension for the map file.

Pedigree file (\*.ped)

Below is a small example PED file (corresponding to the map file just below):

```
1  1  0  0  1  21.2  1  2  1  1  1  2  
1  2  0  0  2   1.3  1  2  2  2  1  1  
1  3  0  0  1   0.9  2  2  2  1  1  2  
1  4  1  2  2  19.1  1  2  2  1  1  2  
1  5  1  2  1  18.3  2  2  2  1  1  2  
1  6  0  0  2   0.7  2  2  1  1  1  2  
1  7  3  4  2  20.5  2  2  1  2  2  1  
1  8  3  4  2  22.1  2  2  1  1  2  2  
1  9  3  4  2  11.1  2  2  1  1  2  2  
1 10  5  6  1  19.5  2  2  1  1  2  2  
1 11  5  6  1  17.9  2  2  1  2  1  2  
2  1  0  0  1   1.2  1  2  2  2  2  2  
2  2  0  0  2  19.1  1  1  2  1  1  1  
2  3  0  0  1   0.8  1  1  1  2  1  2  
2  4  1  2  2  21.1  1  1  2  1  2  1  
2  5  1  2  2  20.3  1  1  2  1  2  1  
2  6  0  0  1   0.7  2  2  1  2  1  2  
2  7  3  4  2  18.6  1  1  2  1  2  1  
2  8  6  5  1  17.6  2  1  2  1  2  1  
2  9  8  7  2  20.2  1  1  2  1  2  1  
2 10  8  7  2  22.3  1  1  2  1  2  1
```

To read the example PED file correctly while naming the quantitative trait “Q1”, we use the Mega2 PLINK flags:

```
--trait Q1 --quantitative
```

This file is very similar to a *pre-makeped* style Mega2 pedigree file. It has the standard five initial columns: pedigree, person, father, mother and sex. This is followed by a single trait and then the markers, coded as pairs of alleles. There is no header for this file, so it includes no information regarding the type of trait in the trait column; this information is supplied via the use of the Mega2-PLINK options -\/-affectionstatus or -\/-quantitative. In Mega2, it is sometimes necessary to have a name for the trait. This name is supplied as part of the Mega2 file selection menu, using the -\/-trait Mega2 PLINK option. (The name is necessary to correlate this trait with the information in the Mega2 penetrance and frequency files.) The Mega2 PLINK flags are:

> |  |  |
> | --- | --- |
> | **flag** | **meaning** |
> | *-\/-*no-fid | The two columns pedigree & person are replaced by one column, specifying a unique identifier |
> | *-\/-*no-parents | The father and mother column are not present; Mega2 will assign the missing value zero ’0’ to the entries in these two columns. |
> | *-\/-*no-pheno | The trait column is not present. |
> | *-\/-*trait | specify a name for the pedigree file trait column. |
> | -\/-affectionstatus | indicate that trait is a dichotomous affection status trait locus (default). |
> | -\/-quantitative | indicate that trait is a quantitative trait locus. |

Map file (\*.map)

Below is a small example map file:

```
5    M1        0.0	1
5    M2        8.0	3
5    M3        5.0	2
```

To read the example map file, under the assumption the genetic map positions are specified in Kosambi centiMorgans, we use the Mega2-PLINK flags:

```
--kosambi --cM
```

This file allows the alleles in the **Pedigree file** to be assigned to a particular marker. Successive lines of the **Map file** identify the marker for each allele pair. Each row typically includes four fields: the chromosome (1-22, X, Y or 0), the marker name, the genetic position in Kosambi Morgans, and finally the base-pair position. The override flags are:

> |  |  |
> | --- | --- |
> | **flag** | **meaning** |
> | *-\/-*map3 | The genetic position column is not present. |
> | *-\/-*cM | The genetic position is specified in centiMorgans. |
> | *-\/-*kosambi | genetic distance in map file is in Kosambi units (default) |
> | *-\/-*haldane | genetic distance in map file is in Haldane units |

There is one final PLINK convention involving the base-pair column. If any marker has a negative base-pair position, then the corresponding data from the **Pedigree file** is not used.

Alternated Phenotype file (header line required)

Below is a small example phe file:

```
FID  IID  TRAIT
1    1    2
1    2   NA
1    3   NA
1    4    2
1    5    2
1    6   NA
1    7    2
1    8    2
1    9    0
1   10    2
1   11    2
2    1   NA
2    2    2
2    3   NA
2    4    2
2    5    2
2    6   NA
2    7    2
2    8    2
2    9    2
2   10    2
```

The PLINK standard allows for only one phenotype in the **Pedigree file**, but more phenotypes can be specified in a separate file. The alternate **Phenotype file** has three or more columns; the first two columns contain the pedigree and person IDs and the remaining columns are phenotypes. The PLINK standard allows there to be a header line for this file. The first two columns of the header **MUST** contain **FID** and **IID** the remaining header columns name the phenotypes. ***Mega2 requires that this header line be present.*** There are flags:

> |  |  |
> | --- | --- |
> | **flag** | **meaning** |
> | *-\/-*missing-phenotype | Specifies the missing value for quantitative traits (default -9). |
> | *-\/-*1 | Affection status is coded 0 (unaffected) / 1 (affected) / -9 (missing,) vs. 0 (missing) / 1 (unaffected) / 2 (affected) / -9 (missing). |

Miscellaneous files

The **omit file**, **frequency file**, and **penetrance file** are part of the Mega2 data schema, and they may be supplied with the PLINK input files.

### 9.2.2 PLINK binary PED input format

**Pedigree files** for GWAS studies can become rather large and slow to process. The PLINK binary PED file makes these data more accessible.

This format is typically coded using three primary files: the pedigree file, the family file, and the map file. The default naming scheme is to use the same stem for all three, and a ’.ped’ extension for the pedigree file, a ’.fam’ extension for the family file, and a ’.map’ extension for the map file.

Binary PED file (\*.bed)

This file is described in detail in the PLINK manual. It tightly encodes the allele data in two bits per SNP. The file is not ASCII and not human readable.

Family file (\*.fam)

Below is a small example fam file:

```
1  1  0  0  1  21.2
1  2  0  0  2   1.3
1  3  0  0  1   0.9
1  4  1  2  2  19.1
1  5  1  2  1  18.3
1  6  0  0  2   0.7
1  7  3  4  2  20.5
1  8  3  4  2  22.1
1  9  3  4  2  11.1
1 10  5  6  1  19.5
1 11  5  6  1  17.9
2  1  0  0  1   1.2
2  2  0  0  2  19.1
2  3  0  0  1   0.8
2  4  1  2  2  21.1
2  5  1  2  2  20.3
2  6  0  0  1   0.7
2  7  3  4  2  18.6
2  8  6  5  1  17.6
2  9  8  7  2  20.2
2 10  8  7  2  22.3
```

To read the example fam file correctly while naming the quantitative trait “Q1”, we use the Mega2 PLINK flags:

```
--trait Q1 --quantitative
```

The “**Family**” **file** only contains the pedigree information; in other words it is the first six columns of the PED **Pedigree file** described above: pedigree, person, father, mother, sex, and trait. The flags shown earlier and repeated below can be used if the **Family file** has fewer than the default six columns. The flags are:

> |  |  |
> | --- | --- |
> | **flag** | **meaning** |
> | *-\/-*no-fid | The two columns pedigree & person are replaced by one column, specifying a unique identifier |
> | *-\/-*no-parents | The father and mother column are not present; 0 is used as the value. |
> | *-\/-*no-pheno | The trait column is not present. |

Binary Map file (\*.bim)

Below is a small example bim file:

```
5	M1	0	1	1	2
5	M3	5	2	2	1
5	M2	8	3	1	2
```

The “**BI**nary” **M**ap file (**BIM file)** is similar to the **Map file** described above. There is no space available in the **Binary PED file** to represent the allele values; so these are added to the end of each line of the **Map file**. So each row typically includes six fields: the chromosome (1-22, X, Y or 0), the SNP name, the genetic position in Morgans, the base-pair position, and the two allele names. The override flags are as before:

> |  |  |
> | --- | --- |
> | **flag** | **meaning** |
> | *-\/-*map3 | The genetic position column is not present. |
> | *-\/-*cM | The genetic position is specified in centiMorgans. |

Note: negative base-pair distances are **NOT allowed**.

Alternate Phenotype file (header line required)

This file is exactly the same as that used for the non binary input. The PLINK standard allows for only one phenotype in the **Pedigree file** but more phenotypes can be specified in a separate file. The **Alternate Phenotype file** has three or more columns; the first two columns contain the pedigree and person and the remaining columns are phenotypes. The PLINK standard allows there to be a header for this file. The first two columns of the header **MUST** contain **FID** and **IID** the remaining header columns name the phenotypes. ***Mega2 requires that this header be present.*** Of course there are flags:

> |  |  |
> | --- | --- |
> | flag | meaning |
> | *-\/-*missing-phenotype | Specifies the missing value for quantitative traits (default -9) |
> | *-\/-*1 | Affection status is coded 0 (unaffected) / 1 (affected) / -9 (missing), vs. 0 (missing) / 1 (unaffected) / 2 (affected) / -9 (missing) |

Miscellaneous files

These files are exactly the same as that used for the non binary input. The **omit file**, **frequency file**, and **penetrance file** are part of the Mega2 data schema, and they may be supplied with the PLINK input files.

## 9.3  LINKAGE input file formats

The LINKAGE format is the one used by the LINKAGE programs (Lathrop and Lalouel 1984; Lathrop et al. 1986; Lathrop and Lalouel 1988); these formats are described in detail in the LINKAGE User’s Guide at

https://linkage.rockefeller.edu/soft/linkage/

and also in the LINKAGE Handbook by Terwilliger and Ott (1994).

LINKAGE format files typically come in pairs: the ’datain.dat’ contains the locus information (disease model, allele frequencies, numbers of alleles, etc.), while the ’pedin.dat’ contains the pedigree structure information and phenotypes. However, the original LINKAGE format made no provisions for locus names nor for marker map information. So Mega2 uses as input a trio of files which remedy these omissions:

1. the LINKAGE locus file modified to contain locus name information;
2. the LINKAGE pedigree file; and
3. the Simple Mega2 map file.

### 9.3.1 LINKAGE locus file

*Default name:* datain.##, where ## is the number of the chromosome (01, 02,..., 23) or datain.ex, where ex is the input file extension. For example, if the chromosome number chosen is 2, then Mega2 looks for the file datain.02 in the current directory.   
The locus data file is in standard LINKAGE format with the addition of locus names, which must be specified. The standard (but not well-known) LINKAGE format for including loci names is to, right after the number of alleles, put a # sign followed by the marker name. For example:

```
5 0 0 5  << NO. OF LOCI, RISK LOCUS, SEXLINKED (IF 1) PROGRAM
0 0.0 0.0 0  << MUT LOCUS, MUT RATE, HAPLOTYPE FREQUENCIES (IF 1)
1       2 3 4 5
1   2   # TRAIT
 0.990000 0.010000   << GENE FREQUENCIES
1 << NO. OF LIABILITY CLASSES
0.0000 1.0000 1.0000 << PENETRANCES
0       2  # Q1
 0.990000 0.010000   << GENE FREQUENCIES
1 << NO. OF TRAITS
1.000   10.000 20.000 << GENOTYPE MEANS
1.000   << VARIANCE - COVARIANCE MATRIX
1.000   << MULTIPLIER FOR VARIANCE IN HETEROZYGOTES
3   2   # M1
 0.500000 0.500000   << GENE FREQUENCIES
3   2   # M2
 0.500000 0.500000   << GENE FREQUENCIES
3   2   # M3
 0.500000 0.500000   << GENE FREQUENCIES
0 0  << SEX DIFFERENCE, INTERFERENCE (IF 1 OR 2)
0.1     0.1 0.1 0.1  << RECOMBINATION VALUES
1 0.10000 0.45000 << REC VARIED, INCREMENT, FINISHING VALUE
```

This setup would give the name TRAIT to the first locus, the name Q1 to the second locus, and the name M1 to the third locus, etc. (You may put no space between the # sign and the locus name, if desired).

Each co-dominant marker name must have an exact match in the corresponding map file; if a locus name in the locus data file is not found in the map file, then the user is warned about this. If the user still chooses to proceed, any marker that was not found in the map file will not appear in any of the output files (as Mega2 would not know which map position to put it in). HINT: This feature can be used to easily exclude a marker from all files produced by Mega2 - simply alter the name of the marker in the map file so that it no longer matches.

### 9.3.2 LINKAGE pedigree file

*Default name:* pedin.##, where ## is the number of the chromosome (01, 02, ..., 23) or pedin.ex, where ex is the input file extension. So if the chromosome number chosen is 2, then Mega2 looks for the file pedin.02 in the current directory.   
The pedigree data file should have either of the following formats,

a) pre-Makeped linkage format

b) the standard (post-Makeped) LINKAGE format without loops.

Example of a pre-Makeped file with an inbred pedigree [no. 2] (to match the example locus datafile above):

```
   1   1   0   0   1  2  21.2 1  2  1  2  1  1
   1   2   0   0   2  0   1.3 1  2  1  1  2  2
   1   3   0   0   1  0   0.9 2  2  1  2  2  1
   1   4   1   2   2  2  19.1 1  2  1  2  2  1
   1   5   1   2   1  2  18.3 2  2  1  2  2  1
   1   6   0   0   2  0   0.7 2  2  1  2  1  1
   1   7   3   4   2  2  20.5 2  2  2  1  1  2
   1   8   3   4   2  2  22.1 2  2  2  2  1  1
   1   9   3   4   2  0  11.1 2  2  2  2  1  1
   1  10   5   6   1  2  19.5 2  2  2  2  1  1
   1  11   5   6   1  2  17.9 2  2  1  2  1  2
   2   1   0   0   1  0   1.2 1  2  2  2  2  2
   2   2   0   0   2  2  19.1 1  1  1  1  2  1
   2   3   0   0   1  0   0.8 1  1  1  2  1  2
   2   4   1   2   2  2  21.1 1  1  2  1  2  1
   2   5   1   2   2  2  20.3 1  1  2  1  2  1
   2   6   0   0   1  0   0.7 2  2  1  2  1  2
   2   7   3   4   2  2  18.6 1  1  2  1  2  1
   2   8   6   5   1  2  17.6 2  1  2  1  2  1
   2   9   8   7   2  2  20.2 1  1  2  1  2  1
   2  10   8   7   2  2  22.3 1  1  2  1  2  1
```

The LINKAGE format is essentially the de facto standard for coding pedigree information in a machine-readable form. For a complete description of this format, please see the Handbook of Human Genetic Linkage (Terwilliger and Ott 1994) and the LINKAGE Users Guide (at http://linkage.rockefeller.edu/soft/linkage/) .   
pre-Makeped LINKAGE pedigree file consist of columns of integer data. The pre-Makeped columns are:

Pedigree Person Father Mother Gender Phenotype1 Phenotype2 Phenotype3 ... \endverbatim

where missing parents are entered as 0 (zero), and, for the gender column, a 1 = Male and a 2 = Female (This is easy to remember if you think of the number of X chromosomes). Makeped inserts some additional columns of pointers (which would be difficult to enter by hand) and breaks loops, which is required by the LINKAGE programs.The columns should be separated by spaces or tabs (any number of these is allowed).  
While the order of the phenotypes is arbitrary, it is common to put the affection status phenotype first, followed by the marker phenotypes (which, for co-dominant markers, are the same as the genotypes).   
**Phenotype coding:**   
1) **Trait locus:** To code a simple affection status locus, use these codes:

```
0 = unknown
1 = normal
2 = affected
```

2) **Marker locus:** To code a codominant marker locus phenotype, simply list the two numbered alleles with at least one space or tab between the alleles. The unknown genotype is coded as 0 0.

Using non-numeric allele names:

Non-numeric allele names are allowed inside the pedigree file only with the use of a names file, and only for numbered marker loci. The recoded output pedigrees will have their genotypes altered to numeric alleles if the target output format requires this. Allele names have to be strings, and may not contain white-space characters, since the pedigree file is read in as a white-space separated column format. See the names file section above for details on recoding.

Note: Everyone must have either two parents or no parents in the data set. Thus, to connect relatives one may need to include people in a pedigree for whom there is at present no data. Example of post-Makeped file corresponding to the pre-Makeped file (above):

```
   1   1   0   0   4   0   0 1  1  2  21.2 1  2  1  2  1  1
   1   2   0   0   4   0   0 2  0  0   1.3 1  2  1  1  2  2
   1   3   0   0   7   0   0 1  0  0   0.9 2  2  1  2  2  1
   1   4   1   2   7   5   5 2  0  2  19.1 1  2  1  2  2  1
   1   5   1   2  10   0   0 1  0  2  18.3 2  2  1  2  2  1
   1   6   0   0  10   0   0 2  0  0   0.7 2  2  1  2  1  1
   1   7   3   4   0   8   8 2  0  2  20.5 2  2  2  1  1  2
   1   8   3   4   0   9   9 2  0  2  22.1 2  2  2  2  1  1
   1   9   3   4   0   0   0 2  0  0  11.1 2  2  2  2  1  1
   1  10   5   6   0  11  11 1  0  2  19.5 2  2  2  2  1  1
   1  11   5   6   0   0   0 1  0  2  17.9 2  2  1  2  1  2
   2   1   0   0   4   0   0 1  1  0   1.2 1  2  2  2  2  2
   2   2   0   0   4   0   0 2  0  2  19.1 1  1  1  1  2  1
   2   3   0   0   7   0   0 1  0  0   0.8 1  1  1  2  1  2
   2   4   1   2   7   5   5 2  0  2  21.1 1  1  2  1  2  1
   2   5   1   2   8   0   0 2  0  2  20.3 1  1  2  1  2  1
   2   6   0   0   8   0   0 1  0  0   0.7 2  2  1  2  1  2
   2   7   3   4   9   0   0 2  0  2  18.6 1  1  2  1  2  1
   2   8   6   5   0   0   0 1  2  2  17.6 2  1  2  1  2  1
   2   9  11   7   0  10  10 2  0  2  20.2 1  1  2  1  2  1
   2  10  11   7   0   0   0 2  0  2  22.3 1  1  2  1  2  1
   2  11   0   0   9   0   0 1  2  2  17.6 2  1  2  1  2  1
```

Defining missing quantitative phenotype values:

The missing value is user-defined: if there are one or more quantitative trait loci in your input files, then Mega2 will ask you what the missing value is. However, you have to use the same missing value for all of the quantitative traits in your file, and (unfortunately) it has to be a real-valued missing value (but need not be zero!).

Using Ped, Per and ID identifiers in the pedigree file:

Unique IDs for each person can be specified in the pedigree file using the tag “Id:”. **NOTE**: Mega2 checks if your unique IDs are unique over the entire set of individuals inside the pedigree file.

If not, Mega2 gives you the option to generate unique IDs by prepending the pedigree name to the ID. Uniqueness is generally not required except for the Cranefoot pedigree drawing option, so Mega2 will automatically create new unique IDs if needed for this option. For the PAP option, Mega2 numbers individual sequentially from 1 through the total number of individuals, thus it neither checks, nor generates unique IDs.   
Non-numeric pedigree and person identifiers can be indicated using the “Ped:” and “Person:” tags. These three tags are case insensitive e.g. the ID tag can be any of “ID:”, “Id:”, “iD:”, or “id:”. Unique IDs are allowed within the pre-makeped format pedigree file, but the “Ped:” and “Per:” tags are not recognized. If these are provided, Mega2 simply ignores these fields as long as they are placed after the phenotype/genotype columns.   
There is no restriction on the ordering of Ped:, Per: and ID: field pairs except that each label has to be followed by the appropriate value. Thus these are correct

... ID: 10001 Ped: 101 Per: 10001

... Ped: 101 Per: 10001 ID: 10001

\endverbatim whereas this is not a correct order:

... Per: Ped: 10001 101 ID: 10001

\endverbatim

For output formats which can handle arbitrary pedigree and person names, Mega2 allows the user to select, which pedigree and person id should be used in the output file. Mendel is one such option. The output pedigree and person IDs can be selected via the “Output file names” menu which looks like:

```
==========================================================
  MENDEL file name menu
==========================================================
0) Done with this menu - please proceed
 1) Locus file name:                   locus.05         [new]
 2) Pedigree filename:                 pedm.05          [new]
 3) M13 batch file name:               m13bat.05        [new]
 4) Batch file name:                   batch.05         [new]
 5) M13 batch file name:               m13bat.05        [new]
 6) Person id in output pedigree file:        Individual id
 7) Pedigree identifier in output
                     pedigree file:           Pedigree number
Select options 0-7 to enter new file names/options >
==========================================================
```

Selecting option 6 will display the individual id selection menu:

```
==========================================================
Output person id selection menu:
0) Done with this menu - please proceed.
*1) Individual id
 2) Create unique id e.g 1_2, 1=ped, 2=ind
 3) Renumber consecutively within pedigree
Select from options 0 - 3 >
==========================================================
```

The current choice is indicated with an asterisk. Option 2 can be either selecting unique IDs, if these are present as the “:ID” field in the input pedigree file, or creation of unique IDs by Mega2 if they are absent.   
Selecting option 7 will display the pedigree id selection menu:

```
==========================================================
Output pedigree id selection menu:
0) Done with this menu - please proceed
*1) Pedigree number
 2) Renumbered consecutively
Select from options 0 - 2 >
==========================================================
```

This functionality is available for the following options:   
Mendel   
SimWalk2   
Aspex   
Genotyping Summary   
SOLAR   
Pre-makeped   
Merlin-SimWalk2-NPL   
(Selection allowed only once, and used in both sets of files   
Merlin   
Loki

### 9.3.3 Handling of loops inside pedigrees

Loop breaking:

Mega2 will automatically select an optimal set of loop-breakers if there are loops inside a pre-Makeped pedigree file, and if the output analysis type requires loop-less pedigrees. For example, VITESSE and SLINK options require loops to be broken in the pedigree.

Mega2’s loop-breaking capabilities have been successfully tested on several pedigrees, including large ones, and complex inter-breeding structures. Multiple marriages are also handled by the Mega2 loop-breaking procedure, although, we currently limit the number of marriages at 10 per person. If your pedigree contains more than 10 marriages per individual, then you are advised to use Makeped in order to break the loops.

Loop-breaker selection menu:

This allows the user to specify whether selecting a loop-breaker should be limited only to the non-founders in a family. The selected list of loop-breakers is displayed as well recorded in the MEGA2.LOG file.

```
==========================================================
Loop-breaker selection menu:
0) Done with this menu - please proceed.
 1) Select only non-founders as loop-breakers [n].
Enter 1 to toggle, 0 to exit >
```

Loop reconnection:

Mega2 will automatically reconnect the loops of a linkage pedigree file when necessary. For example, this is done when generating output files in MENDEL, SAGE, and SOLAR formats. The reconnection will, unfortunately, result in a renumbering of person IDs.

If the input pedigree was in pre-Makeped format, then the pedigrees remain intact for these options.

Mega2 displays each pair of pedigree records that were re-connected, as well as logging them in the MEGA2.KEYS file.

### 9.3.4 Simple Mega2 Map file

*Default name:* map.##, where ## is the number of the chromosome (01, 02, ..., 23) or map.ex, where ex is the input file extension. So if the chromosome number chosen is 2, then Mega2 looks for the file map.02 in the current directory.

The map file gives the (relative) map position of each marker in centiMorgans (cM). If two markers fall at exactly same position, then Mega2 will assume that the marker listed first should come first, and will automatically add a small increment (of 0.0001 cM) to the position of the second marker.

Note that Mega2 can now make the distinction between Haldane and Kosambi map distances by looking at the first line of the map file. If the second column heading contains “Kosambi”, then the distances are read in as Kosambi centimorgans.

An additional 4th column can be added to the map file specifying mistyping probabilities for each marker. These values are utilized within the Genotyping error simulation option. This column should have the heading “error” (case-insensitive).

Some of the analysis options like SLINK, Vitesse etc. use recombination fractions. In this case, the appropriate mapping function is used to convert the inter-marker distances into recombination fractions. In Aspex, e.g., if the user chooses to output Kosambi map distances, using Haldane distances in the map file, the map will first be converted to thetas which will then be converted to Kosambi map distances.

Example map file, ’map.05’:

```
CHROMOSOME    KOSAMBI   NAME
5             0.0       M1
5             5.0       M3
5             8.0       M2
```

NOTE: Any marker that is in the locus file must be given a map position in the map file. Thus, the marker names used in the map file must match exactly the names used in the locus file. If a codominant marker locus in the locus file is not found in the map file, then Mega2 will warn you about this. If you ignore the warning, then this locus will not appear in the output files created by Mega2. You may have more loci in the map file than appear in the locus file. While you will be warned about this, it does not pose any difficulties.

NOTE: The Simple Mega2 Map file is only available for linkage input.

Hint: See the Genetic Map Interpolator section 10↓ for help on creating map files.

### 9.3.5 Specifying sex-specific maps

The simple map file now allows two extra columns for specifying male and female map distances. These columns should appear after the “Name” column. They should be labeled “male” and “female” respectively, and Mega2 is case-insensitive to these headers. The map function for the male and female maps is assumed to be the same as the sex-average map (the second column). Here is an example of a Mega2 map file, ’map.05’, containing male and female maps:

```
CHROMOSOME    KOSAMBI   NAME    MALE       FEMALE
5             0.0       M1      0.0        0.0
5             5.0       M3      2.0        7.0
5             8.0       M2      4.0       12.0
```

### 9.3.6 Omit file [Optional]

The optional omit file permits one to easily delete certain genotypes/phenotypes from all Mega2-generated output files. This is useful if certain genotypes are Mendelianly-inconsistent, yet one wants to preserve the original marker data in the input file, or one wishes to exclude specific phenotypes from the output.

The omit file should be in the following format:

Each line should contain three items separated by white space. These items represent pedigree, person, and loci. For a LINKAGE format file these must be ordered pedigree, person, and loci respectively. For a Mega2 format file the columns can be in any order but must be named Pedigree, Individual, and Marker. The pedigree should be either All, or an integer that must match a pedigree number used in your input file. If All, then the locus(loci) with associated person(s) will be untyped for all pedigrees. Otherwise only loci with the specific pedigree number will be affected. The person should be either All, a zero (synonym for All), or an integer that must match a person number used in your input file. If a zero, or All, then the locus(loci) with associated pedigree(s) will be untyped for all persons. Otherwise only loci with the specific person number will be affected. The loci (third item) should be either All, or the name of a locus used in your input file. If All, then the person(s), and pedigree(s) indicated will be untyped for *all marker* *loci only; trait loci will not be affected*. Otherwise only the specific loci (marker or trait) will be affected.

A summary of the omit results will be found in the file omit.log. This file is rewritten the next time Mega2 is run with an omit file specified.

The following describes possible errors and warnings associated with the reading of an omit file. If Mega2 can not find a specific pedigree, person, or loci (from the omit file) in the input data, it will halt with an error message. If All is used for a loci in the omit file, and the input data contains a loci named All, it will halt with an error message. If the pedigree is specified as All, and a person number given, a warning will be issued if that person cannot be found within one of the available pedigrees. If pedigree, person, and loci are all specified as All, a warning will be issued stating that all marker loci will be untyped.

Example LINKAGE format omit file, ’omit.05’:

```
1 0   All
2 10  M2
2 All M1
```

This file generates the following log file:

```
Untyped all individuals in pedigree 1 at all marker loci.
Untyped individual 10 in pedigree 2 at locus M2.
Untyped all individuals in pedigree 2 at locus M1.
```

The omit file can also be used to set trait phenotypes to unknown. Here is such an example:

```
1 11 AFF2
1 11 QUANT1
```

These two lines direct Mega2 to “untype” person 11 of pedigree 1 at the affection status locus AFF2 and the QTL locus QUANT1. The affection status will be set to unknown (0), and the quantitative phenotype will be set to the appropriate missing value in the output (see 101↓). These actions are logged as well.

Please note that when the loci column contains the keyword “All”, it still refers to only marker loci; trait loci are left untouched. You may also use a locus named “All” in your input data so long as you do not use “All” as a loci in the omit file.

*Default name:* omit.##, where ## is the number of the chromosome (01, 02, ..., 23) or omit.ex, where ex is the input file extension. So if the chromosome number chosen is 2, then Mega2 looks for the file omit.02 in the current directory. NOTE: Since a person who breaks a loop is indicated twice in a post-Makeped pedigree file, if you want to untype a loop breaker, you must explicitly untype both occurrences of this loop breaker person. Mega2 checks to see if loop-breakers have the same genotypes, and will flag an error otherwise.

Hint: See the section 26.3↓ on creating omit files based on errors found by running the pedigree-checking program Pedcheck.

### 9.3.7 Header-less Names file

The names file is an alternate way of specifying locus data. It consists of only locus names and types. If a names file is used, the pedigree file can contain **uncoded genotypes** which could be base-pair sizes or non-numeric allele names. Mega2 will then read in the pedigree file, and, if needed, recode the pedigree data marker genotypes as numbered alleles.

Here is a names file, ’names.05’, which corresponds to the locus datafile in the preceding section.

```
A TRAIT
T Q1
M M1
M M2
M M3
```

Six locus types are recognized, autosomal numbered(M), x-linked numbered(X), binary trait with a single liability class(A), binary trait with multiple liability classes(L), quantitative traits(T) and covariates(C). The pedigree data file is then processed as follows:

An autosomal or X-linked numbered locus is read in as a pair of character strings, a binary trait is read in as a single number which has to be 0, 1 or 2, a trait locus with multiple liability classes is read in as a  < status, liability-class >  pair, where status is 0, 1, or 2, and liability-class is any number greater than 0.

Names file and Mega2’s RECODE facility

Mega2’s **recoding facility** eliminates the need for conversion of raw genotype data into consecutively numbered alleles which is required by the linkage format specification. This facility also eliminates the need to have allele frequencies for chromosomal markers, instead estimating these from the pedigree data itself.

In the Mega2 file format, providing a frequency file will cause the allele-frequency estimation to be skipped, only allele labels will be recoded.

## 9.4  Variant Call Format (VCF, BCF, compressed VCF) input file formats

Variant Call Format (VCF) was designed to store information about genetic variants (See: Wikipedia). Mega2 currently uses source code from the popular VCFtools program (See: VCFtools) as its underlying engine for parsing VCF files. VCFtools (and so Mega2) currently supports VCF format v4.0 and VCF format v4.1 (See: VCF Specification), and BCF format v2.1 (See: BCF (Binary VCF) version 2). Mega2 supports only the ’filtering’ options associated with the binary executable ’vcftools’ file (See: VCFtools Options). In particular, the options supported are: the Site Filtering Options, the Individual Filters, and the Genotype Filters. These options are specified to Mega2 as they would be to the ’vcftools’ program in the form of a command line option string. In Mega2 interactive mode (See 12.5↓) Mega2 allows the user to specify the filtering options via the input menu, and in batch file mode the VCF\_Args batch file item is used (See: 80↓).

VCF input format uses a subset of the PLINK parameters (See 48↓) to allow the user to specify phenotype information. With these the user can: specify a name for the pedigree file trait column (See 9.4.2↓), indicate the trait to be affection status or quantitative, specify the missing value for quantitative traits, and specify how the affection status is coded.

Mega2 and the ’vcftools’ program will accept variant call input files in one of three formats: text only, gzipped compressed text only, or binary file. The variant call file format is specified using the appropriate menu in Mega2 (See 12.5↓) or using the batch file item Input\_Format\_Type (See: 69↓) set to the values of: 5 for a .bcf (binary) file, 6 for a .vcf.gz (compressed text) file, and 7 for a .vcf (text) file.

In addition to variant information, Mega2 needs additional information (i.e., about family structure, genetic map positions, etc.) depending on the output analysis chosen (See 13↓). The VCF file format allows for the possibility of user-defined fields, which could in theory specify this additional information within the VCF file. However, in many instances there is no consistent method for representing this information, and so Mega2 instead takes the approach of specifying additional information in separate files, which is consistent with other input file formats. As the VCF file standard evolves, we will revisit this decision. For example, the map position information in the variant file does not include genetic position information. Genetic position information can be easily input by providing a Mega2-format map file.

When using VCF-format input files, one needs to provide several files, which are described below. At a minimum, two files are required: 1) the VCF file containing the marker genotypes, and 2) the PLINK-format family file containing the family structure information. Optional files include a phenotype file, a map file, an omit file, an allele frequency file, and a penetrance file. The phenotype file may include a SAMPLEID column specifying the key mapping individuals in the family file to the samples in the VCF file.

### 9.4.1 Variant Call File

The Variant Call File (See: VCF Specification) is divided into three sections. The first section contains the meta information (lines from the beginning of the file that begin with the double hash mark ’##’). The second section contains only one line which is the header line (this line follows the last meta information line and begins with a single hash mark ’#’). The third section follows the header line, and contains all of the variant call information. The header line contains a number of required fields, followed by sample identifiers (See 9.4.3↓ for rules on mapping individual identifiers to sample identifiers). In interactive mode the VCF file is specified through the “Variant file” menu item. In batch file input the VCF file is specified using the Input\_Aux\_File batch file item (See 78↓).

A simple example VCF file is as follows:

```
##fileformat=VCFv4.0
##fileDate=20131205
##FORMAT=<ID=GT,Number=1,Type=String,Description="Genotype">
##FILTER=<ID=PASS,Description="Passed variant FILTERs">
#CHROM  POS     ID      REF     ALT     QUAL    FILTER  INFO    FORMAT  1_3     1_4     1_5     1_6     1_7
5       2       M1      A       T       .       PASS    .       GT      1/1     0/1     1/1     1/1     1/1
5       4       M3      G       C       .       PASS    .       GT      0/1     0/1     0/1     0/1     0/1
5       6       M2      T       A       .       PASS    .       GT      0/1     0/1     0/1     0/0     0/1
```

The file continues off of right of the page with the following header and marker data.

```
1_8     1_9     1_10    1_11    2_3     2_4     2_5     2_6     2_7     2_8     2_9     2_10
1/1     1/1     1/1     1/1     0/0     0/0     0/0     1/1     0/0     0/1     0/0     0/0
0/0     0/0     0/0     0/1     0/1     0/1     0/1     0/1     0/1     0/1     0/1     0/1
0/0     0/0     0/0     0/1     0/1     0/1     0/1     0/1     0/1     0/1     0/1     0/1
```

This simple example VCF file specifies three biallelic markers (M1, M3, and M2). It should be noted that Mega2 is not restricted to biallelic markers. The markers and their information is as follows:

M1 chromosome: 5; physical position: 2; reference allele: 1; alternate allele: 2

M3 chromosome: 5; physical position: 4; reference allele: 2; alternate allele: 1

M2 chromosome: 5; physical position: 6; reference allele: 1; alternate allele: 2

This VCF file contains seventeen (17) sample IDs (1\_3, 1\_4, ..., 2\_10). If the sample IDs do not correspond to the person IDs in the family file, they can be mapped to individual IDs using a SAMPLEID column in the phenotype file (See 9.4.3↓).

The map information from the VCF File is parsed and made available by Mega2. In interactive mode the map is selectable as ’VCF.p’. In batch file mode this map is specified by setting the value of Value\_Base\_Pair\_Position\_Index (See 94↓) equal to 0 if no Mega2 Map File is specified (See: 9.1.3↑), or to one greater than the number of maps available in the Mega2 Map File. The map file (VCF.p) that is derived from the VCF file will contain all of the markers that have passed the vcftools filtering criteria. The Mega2 Map File may contain the same number of markers or less in which case it will provide additional filtering. That is, markers will be dropped if they do not appear in a map that is selected from a Mega2 Map File.

In many instances a Mega2 analysis option may require a genetic map. When using a VCF file, a genetic map can be specified using a optional Mega2 Map File (See 9.1.3↑). However, if an analysis option requires a genetic map and a Mega2 Map File is not specified or if a map is selected that contains no genetic maps, then a dummy Sex Average genetic map (whose values are all 0) will be used for the required genetic map. For example, this would allow one to convert from VCF input format to PLINK output format (with a dummy genetic map) when only physical map positions are known.

The marker associated with a line in the VCF file can be specified as the string associated with the ’ID’ field of the VCF file (as in the above example), or as the value associated with a key in the ’INFO’ field. This key can be specified in interactive mode, or in batch mode through the batch file identifier VCF\_Marker\_Alternative\_INFO\_Key. For a full description of how this is done please see 81↓.

**Converting from a VCF to a BCF file**

It is possible to use the standalone VCFtools program to convert from a VCF file to a BCF file using the following command:

```
$ vcftools --vcf my_study.vcf --recode-bcf-to-stream > my_study.bcf
```

For other uses of VCFtools and its options please see vcftools: Usage and Options2.

### 9.4.2 PLINK family File

A PLINK family file is used to specify pedigree information. See 23↑ for a PLINK family file associated with the simple example given here. In a family base study, it is possible (if not likely) that some of the individuals found in the family file will not have been genotyped, and therefore will have no corresponding samples mapped to them in the VCF file. In this case, Mega2 will assign these individuals with the missing genotype, and included them in the list of individuals available for processing. If on the other hand, an individual in the VCF file is excluded from the family file, then they are not considered for processing by Mega2. In this manner the family file drives individual selection.

### 9.4.3 Augmented PLINK Phenotype File

The PLINK Phenotype file (See 20↑) lists the family identifier (FID), the individual identifier (IID), and any traits that can be considered. In addition, the Phenotype file is used to map the sample identifiers from the header line in a VCF file (See: 9.4.1↑) to the family and individual identifiers. It is possible for these sample identifiers to correspond to individuals, but this is generally not the case. The sample identifiers correspond to individuals if they have the same string values associated with individuals. For family based data analysis a sample identifier must be associated with an individual and family information. To effect this, the approach that was chosen was to allow an additional column (SAMPLEID) to be specified in the PLINK format Input\_Phenotype\_File (See 20↑) that would allow mapping of sample identifier to the individual (IID) and family (FID).

An example of a phenotype file corresponding to the simple example VCF file (See 9.4.1↑) could look like this:

```
FID  IID  TRAIT SAMPLEID
1    1    2     NA
1    2   NA     NA
1    3   NA     1_3
1    4    2     1_4
1    5    2     1_5
1    6   NA     1_6
1    7    2     1_7
1    8    2     1_8
1    9    0     1_9
1   10    2     1_10
1   11    2     1_11
2    1   NA     NA
2    2    2     NA
2    3   NA     2_3
2    4    2     2_4
2    5    2     2_5
2    6   NA     2_6
2    7    2     2_7
2    8    2     2_8
2    9    2     2_9
2   10    2     2_10
```

Note that four individuals (FID IID 1 1; 1 2; 2 1; 2 2) do not have sample information in the VCF file in the example above.

### 9.4.4 Map File (Optional)

A Mega2 Map File (See 9.1.3↑) is not required, but may be specified. The maps in the map file may be used in addition to the map found in the VCF file (VCF.p). When used with a VCF file, the Mega2 Map file may not contain a map with the name VCF.p.

### 9.4.5 Omit File (Optional)

An omit file may be specified. Please see 74↓.

### 9.4.6 Frequency File (Optional)

A Mega2 frequency file may be specified. Please see 9.1.5↑. The following file could be used with the example VCF file shown in 9.4.1↑. Note that corresponding REF and ALT alleles are used.

```
Name Allele Frequency
A1 1 0.990000
A1 2 0.010000
M1 A 0.500000
M1 T 0.500000
M2 A 0.500000
M2 T 0.500000
M3 G 0.500000
M3 C 0.500000
```

### 9.4.7 Penetrance File (Optional)

A Mega2 penetrance file may be specified. Please see 9.1.6↑.

# 10 Genetic Map Interpolator (GMI)

Given a list of markers, one may easily create a map file containing both genetic and physical positions for these markers using our Genetic Map Interpolator (GMI) program, which is available from https://watson.hgen.pitt.edu/register/.

The Genetic Map Interpolator (GMI) program is designed to create interpolated genetic maps of single nucleotide polymorphism (SNP) markers. Starting from a list of single nucleotide polymorphism (SNP) **rs** numbers, GMI fetches the most up-to-date SNP and microsatellite physical positions from Ensembl, and then combines these with the Rutgers combined genetic and physical map (Matise et al., 2007; Kong et al., 2004; Kong and Matise, 2004) to estimate the corresponding Kosambi genetic positions by linear interpolation for these SNPs. The resulting information is then output in map files formatted to be read in by Mega2 (Mukhopadhyay et al., 2005; https://watson.hgen.pitt.edu/register/) .

GMI has a number of useful features:

- GMI automatically looks up and uses the physical positions for each marker from the *most recent* Ensembl build.
- For each SNP that is not initially found in Ensembl, GMI automatically checks to see if that SNP has been assigned a new rs number.
- GMI automatically figures out which chromosome each marker is on, so knowledge of a marker’s chromosome is not required to run GMI.

GMI is available from https://watson.hgen.pitt.edu/register/ and documentation for GMI is available at https://watson.hgen.pitt.edu/register/docs/gmi.html.

# 11 Converting to Mega2’s format

We have provided a Python script, l2a.py, to convert linkage format files to Mega2’s format. This is included within the Mega2 distribution package.

This format is strongly encouraged as it is less prone to data errors, although it is a more complicated format.

Usage:   
 **l2a.py** without any arguments for help with input options and arguments

**l2a.py -p pedfile -d locusfile -m mapfile -o omitfile -x output-extension**

Output files are named pedin.conv, names.conv, penetrance.conv, frequency.conv, map.conv and omit.conv, if no output extension is specified via the -x option. Otherwise, the user-specified extension is used.

**Inputs to l2a.py:**  
The user is prompted twice for missing value indicators:

```
Missing quantitative phenotype value
```

- ```
     To enter a string value, enclose within "",
     otherwise entered value will be interpreted numerically.
  Enter missing value indicator >
  ```

Entering a value without quotes (e.g. -99) will allow you to interpret all phenotypes numerically evaluating to -99 (-99.00000, -99.0 etc.) to be considered missing.

Affection and unknown allele values are always interpreted as strings:

```
 Unknown allele value string > 0
```

- ```
  ----Unknown allele 0----
  ```

## 11.1 Pedigree file conversion

The converter can detect pre- or post-makeped pedigree formats, by checking if the 5th column contains 0s; for a pre-makeped format file, this should contain only 1s and 2s, since it is the sex indicator.

If your data contains unique individual identifiers as specified by the “ID:” field, then these will be used for individual IDs. **NOTE**: Mega2 checks if your unique IDs are unique over the entire set of individuals inside the pedigree file. If not, Mega2 gives you the option to generate unique IDs by prepending the pedigree name to the ID. Uniqueness is generally not required except for the Cranefoot pedigree drawing option, so Mega2 will automatically create new unique IDs if needed for this option. For the PAP option, Mega2 numbers individuals sequentially from 1 through the total number of individuals, thus it neither checks, nor generates unique IDs.

If your pedigree file contains “Ped:” and “Per:” fields, these are filled in as “PedID” and “PerID” columns.

The linkage pedigree and person IDs (1st and second columns respectively) are stored as “LinkPedID” and “LinkPerID” respectively, and these can be used as output individual and pedigree IDs if you select menu items 1 from the output identifier selection menus described below.

**Missing value indicators in pedigree file:** Usually pedigree data contains unknown genotypes and phenotypes. The l2a.py script prompts the user to enter two missing value indicators, one for marker alleles and affection status, and another one for missing quantitative. If you started off from a linkage format file, unknown alleles and affection status is usually coded as 0 whereas missing quantitative traits are coded as some unlikely number such as -99. All missing genotype and phenotype values are coded as NA in the converted Mega2 file.

**Missing fields inside pedigree records:** The converter decides the number of fields required inside each line by looking at the pedigree-file format (post- or pre- makeped), the number of loci defined inside the names file, and whether extra fields such as Ped:, Per: and ID: are present. It flags an errors and terminates if there are not enough fields on one or more lines. If there are extra columns, it warns the users and proceeds. However, such variations could be indicative of problems with the input data, therefore, even if the conversion continued, the resulting output file could have wrong values.

## 11.2 Locus file conversion

If you are using a names-format file, then only a names file will be created. If you convert a linkage format locus data file with frequencies and trait penetrances, then Mega2 format frequency and penetrance files are also written.

## 11.3 Map file conversion

The converter checks the map function type and male or female-specific maps from the map file headers, and creates the appropriate Mega2 map file.

# 12 The input menu

When you first started up Mega2, it will present a menu for entering input file names in Mega2 native format:

```
==========================================================
              Mega2 4.7.1 input menu:
==========================================================
0) Done with this menu - please proceed
 1) Select input file format:                   Mega2 format with header
 2) Input file suffix:                          01
 3) Locus file:       (Mega2 datain.)[required] _
 4) Pedigree file:    (Mega2 pedin.) [required] _
 5) Map file:         (Mega2 map.)   [required] _
 6) Omit file:        (Mega2 omit.)  [optional] _
 7) Frequency file:   (Mega2 freq.)  [optional] _
 8) Penetrance file:  (Mega2 pen.)   [optional] _
 9) Output Directory:                           [ Current directory ]
10) Simulate genotyping errors:                 [ no ]
11) Include all pedigrees whether typed or not 
12) Maximum number of alleles per marker:       2 alleles
Select from options 0-12 >
```

Each line that selects an input file indicates the type of file, the format of the file (here Mega2), the prefix (when prepended to the suffix) [ or the suffix (when appended to a stem)] and whether the file is required or optional. Whether the file type is embedded in the suffix or prefix depends on the convention used for the particular input class. Additional menu lines are used to set operational parameters.

“Line 1” (Select input file format) introduces a special menu that lets you select a specific input type from Linkage, Mega2, PLINK (choosing among various subtypes) and Variant Call Format (VCF) (choosing among various subtypes).

```
==========================================================
Mega2 4.7.1 input file type menu:
==========================================================
0) Done with this menu - please proceed
*1) Mega2 format with header
 2) Linkage format
 3) Linkage with Mega2 names file
 4) PLINK binary PED format (bed)
 5) PLINK PED format (ped)
 6) BCF format (bcf)
 7) VCF compressed format (vcf.gz)
 8) VCF format (vcf) 
Select from options 0-8 >
```

For example, selecting “Line 6” (BCF format (bcf)) replaces the old input menu with a new menu:

```
==========================================================
              Mega2 4.7.1 input menu:
==========================================================
0) Done with this menu - please proceed
 1) Select input file format:                 BCF format (bcf)
 2) Enter VCF parameters:                     --remove-indels
 3) Enter PLINK parameters:                    --missing-phenotype -9 --trait default
 4) Input file stem:                          study
 5) Variant file:     (binary .bcf)[required] _
 6) Pedigree file:    (PLINK .fam) [required] _
 7) Phenotype file:   (PLINK .phe) [optional] _
 8) Map file:         (Mega2 .map) [optional] _
 9) Omit file:        (Mega2 .omit)[optional] _
10) Frequency file:   (Mega2 .freq)[optional] _
11) Penetrance file:  (Mega2 .pen) [optional] _
12) Output Directory:                         [ Current directory ]
13) Simulate genotyping errors:               [ no ]
14) Include all pedigrees whether typed or not
15) Maximum number of alleles per marker:     2 alleles
Select from options 0-15 >
```

Here you see that some (actually one) of the files are intrinsic Variant Call Format files (.bcf), but other files are in PLINK format, and other files are in Mega2 native format. The reason for this varied assortment of file formats will be explained later. Below, we will discuss the input-specific files first, then the other menu items.

Note: Mega2, by default, starts with the “Mega2 format with header” input type. It is possible using the flags in the table below to have Mega2 start with a different input type.

```
|  |  |
| --- | --- |
| Mega2 Argument | Initial Input File Type |
| --mega2 | Mega2 format with header |
| --linkage | Linkage format |
| --extend_linkage | Linkage with Mega2 names file |
| --bed | PLINK binary PED format (bed) |
| --ped | PLINK PED format (ped) |
| --bcf | BCF format (vcf) |
| --vcf.gz | VCF compressed format (vcf.gz) |
| --vcf | VCF format (vcf) |
```

Note: Batch files created with Mega2 version 4.6.1 or earlier will not have an input type. In this case, Mega2 will look at the inputs and try to decide what the input type was choosing from the Mega2, Linkage, and PLINK types.

## 12.1 Input formats: Mega2 and Linkage

Input menu item 1: Input file extension:

The first item is a file extension. When the input file extension (ext) is specified, Mega2 will search through the current directory and retrieve files named datain.ext (or names.ext), pedin.ext, map.ext, omit.ext, frequency.ext and penetrance.ext, if they exist. So it is a good idea to use the default file naming schemes, as then many items of the input menu are automatically populated once the correct extension has been chosen.

**Locus, Pedigree and Map datafile:**

The locus, pedigree, and map file are required files. In future versions, for the Mega2 format, the names file will be made optional, since locus names and types are already specified in the first line of the pedigree file.

Input menu item: Locus datafile:

This specifies the locus file name.

Input menu item: Pedigree datafile:

This specifies the pedigree file name.

Input menu item: Map datafile:

This specifies the name of map file. The locus datafile, pedigree datafile and the map datafile are now mandatory for all Mega2 options. If the input pedigree and locus data do not contain mapped loci, then a dummy map file should be created with a single marker, which need not be present in the pedigree and locus files. See the locus reordering section for more details.

## 12.2 Common PLINK menu items

Menu item: PLINK parameters

This menu item is displayed mainly for PLINK PED and PLINK binary PED input. As discussed previously in section 9.2↑, the ***plink*** program will accept input files with omitted columns if an appropriate flag is specified. Mega2 also respects these flags and allows them to be set. Selecting this option displays the available feature flags:

```
PLINK options: --no-fid --no-parents --no-pheno --1 --map3 ...
    --missing-phenotype <value> --cM --kosambi/--haldane ...
    --trait <value> --affectionstatus/--quantitative
```

Most of these flags are familiar from the previous PLINK file format discussion in Section 9.2↑. The new flags will be explained below:

|  |  |
| --- | --- |
| **flag** | **meaning** |
| *-\/-*kosambi | genetic distance in map file is in Kosambi units (default) |
| *-\/-*haldane | genetic distance in map file is in Haldane units |
| *-\/-*trait | specify a trait name for the pedigree file trait column (the default trait name is ’default’) |
| -\/-affectionstatus | indicate that the trait is a dichotomous affection status trait locus (default) |
| -\/-quantitative | indicate that the trait is a quantitative trait locus |

For reference, we will repeat the PLINK related flags from section 9.2↑

> |  |  |
> | --- | --- |
> | **flag** | **meaning** |
> | *-\/-*no-fid | The two columns pedigree & person are replaced by one column, specifying a unique identifier |
> | *-\/-*no-parents | The father and mother column are not present; Mega2 will assign the missing value zero ’0’ to the entries in these two columns. |
> | *-\/-*no-sex | The sex column is not present; 0 is used as the value. |
> | *-\/-*no-pheno | The trait column is not present. |
> | *-\/-*map3 | The genetic position column is not present. |
> | *-\/-*cM | The genetic position is specified in centiMorgans. |
> | *-\/-*missing-phenotype | Specifies the missing value for quantitative traits (default -9) |
> | *-\/-*1 | Affection status is coded 0 (unaffected) / 1 (affected) / -9 (missing,) vs. 0 (missing) / 1 (unaffected) / 2 (affected) / -9 (missing) |

You type the desired flags for the PLINK parameters menu item line and hit enter to have the flags (validated and) recorded.

Menu item: Input file stem

This field becomes the file stem. If the stem is set to ’plink’, then Mega2 attempts to fill in the menu items that require file names by searching for plink.ped, plink.bed, plink.bam, plink.fam, plink.map, and plink.phe and additionally plink.omit, plink.frequency, and plink.penetrance.

## 12.3 Input format: PLINK binary PED

Pedigree, Map and Binary datafile:

The pedigree (.fam), map (.bim) and binary (.bed) files are required for PLINK binary PED input. The phenotype file as well as the omit, frequency and penetrance files are optional.

Input menu item: Pedigree datafile:

This specifies the pedigree file name (six column “fam” file).

Input menu item: Map datafile:

This specifies the extended map file with allele data (six column “bim” file).

Input menu item: Binary PED datafile:

This specifies the binary allele data.

## 12.4 Input format: PLINK ped

Pedigree and Map datafile:

The pedigree (.ped), and map (.map) files are required for PLINK PED input. The phenotype file as well as the omit, frequency and penetrance files are optional.

Input menu item: Pedigree datafile:

This specifies the pedigree file name (.ped file).

Input menu item: Map datafile:

This specifies the four column map file (.map file).Common PLINK menu items

## 12.5 Common Variant Call Format menu items

The Variant Call Format file (See 9.4↑) can be presented as text (.vcf), compressed text (.vcf.gz) or binary format (.bcf). Conceptually, this file is a combination of the PLINK .bed/.ped file (for genotype information) and the PLINK .map file (for chromosome and physical position). The variant file is missing the pedigree information (.fam) and phenotype information (.phe) that we customarily need for linkage analysis. Hence, we allow the user to supply these two additional files in PLINK format. Then we need to allow a few of the PLINK parameters introduced above for these PLINK files.

Menu item: PLINK parameters

Restricted list of available PLINK parameters. Selecting this option displays the available feature flags:

```
PLINK options: --missing-phenotype <value> --trait <value> ...
```

- ```
  --affectionstatus/--quantitative
  ```

The flags are a subset of the flags allowed for PLINK input:

|  |  |
| --- | --- |
| **flag** | **meaning** |
| *-\/-*trait | specify a name for the pedigree file trait column |
| -\/-affectionstatus | indicate that trait is a binary affection status |
| -\/-quantitative | indicate that trait is an arbitrary number |

Again, we will repeat the supplementary PLINK related flags from section 9.2↑

> |  |  |
> | --- | --- |
> | **flag** | **meaning** |
> | *-\/-*missing-phenotype | Specifies the missing value for quantitative traits (default -9) |
> | *-\/-*1 | Affection status is coded 0 (unaffected) / 1 (affected) / -9 (missing,) vs. 0 (missing) / 1 (unaffected) / 2 (affected) / -9 (missing) |

Menu item: Variant Call Format parameters

Mega2 supports only the ’filtering’ options associated with the binary executable ’vcftools’ file (See: VCFtools Options). In particular, the options supported are the: Site Filtering Options, the Individual Filters, and the Genotype Filters.

Note that filtering must be done using position information and sample IDs from the input VCF file.

## 12.6 Input format: Variant Call Format files

Pedigree and Map datafile

The variant file in the selected format and the pedigree (.fam) are required for Variant input files. The phenotype file as well as the Mega2 map, omit, frequency and penetrance files are optional.

Input menu item: Variant Call Format datafile:

This specifies the vcf file name as .bcf, .vcf, or .vcf.gz as appropriate to the input type chosen.

Input menu item: Pedigree datafile:

This specifies the pedigree file name (.fam file).

Input menu item: Map datafile:

This specifies a Mega2 map file to provide genetic position information for analysis modes.

## 12.7 Input menu items: Omit, frequency and penetrance data files (optional):

This specifies the names of the omit file, allele-frequency file and trait penetrance file respectively. The omit, frequency and penetrance input data files are all optional files. The frequency and penetrance files are read in only for the Mega2 file format. These files were created to support the Mega2 native file format, but we allow these files to be specified for PLINK and VCF file formats.

## 12.8 Input menu item: Output Directory:

This specifies the name of the directory where Mega2 creates the output files. By default, this is set to the directory from which Mega2 is invoked. The output directory can be specified either as an absolute path (e.g., /home/user/mega2\_results/example) or a relative path. Mega2 checks to see if the specified directory exists, if not, the user is asked if this directory should be created before proceeding.

## 12.9 Input menu item: Simulating genotyping errors:

This option can be toggled between *yes* and *no* by simply selecting option 8 of the input menu. The default value is no. Setting this option to “yes” will cause Mega2 to execute a genotyping error simulation step after selection and reordering of marker loci. In this step, genotypes of individuals are changed at user-selected marker loci, according to a user-specified error-probability model.

## Genotyping error simulation

Overview

This option was created in order to evaluate Genotyping error detection software. This allows the user to introduce errors at a certain percentage of genotypes within a marker, by changing the input genotype subject to certain requirements (such as allele frequencies, whether to select a homozygous or heterozygous genotype, etc.). This option can be turned on and off using in the Mega2 input menu.

## Parameter selection menu

The mistyping simulation step requires selection of loci at which errors should be introduced, the probability model for introducing errors, and names of the output files which contain lists of changed genotypes, and the percentage of genotypes changed.

The error simulation menu is as follows:

```
Error model and loci selection menu 
0) Done with this menu - please proceed. 
1) Apply error model to selected loci. 
2) Apply error to all except selected loci. 
3) Select error model Uniform 
4) Change error probability [0.050] 
5) Mistyping genotypes file name error_genos.06 [new] 
6) Mistyping summary file name error_sum.06 [new]
```

**Marker selection**   
Loci can be selected by two methods: (a) by specifying loci that should have errors, or, (b) specifying loci that should NOT have errors.

**Error model selection**   
Currently input error probabilities can follow any one of three models:

- One uniform error rate for all selected markers

  A uniform rate means that alternate genotypes are selected with a uniform probability). The value of this probability is set to 0.05 by default, and can be changed via the menu.
- Separate uniform error rates for each selected marker:

  These error rates have to be specified inside the map file as a 4th column under the heading “error”. These rates cannot be changed once Mega2 has started running.
- SimWalk2’s error model:

  This involves the specification of 5 separate error probabilities, which can be changed using the menu. For an explanation of these probability values, refer to the paper:   
  **Detection and Integration of Genotyping Errors in Statistical Genetics**   
  by Sobel et al. in AJHG, Vol 70: pages 496-508.

## Error simulation output files

Two output files and a log file is created for each run of Mega2 with the mistyping simulation option. The log file is named **MEGA2.ERR** and behaves like the other log files. It contains details on the options selected by the user via the menu, and a log of each genotype changed in the process.

Two other output files are created which are in table formatted for easy reading, a genotypes file, and a summary file. Here is a part of a genotypes file created with the SimWalk2 error model:

```
Locus   Pedigree Person Orig1 Orig2  Mis1  Mis2    Error type
D06G025        1     10     6     6      1     1   E3 Homozygote
D06G025        1    460     5     6      6     6              E1
D06G025        1    461     5     6      3     3 E5 Heterozygote
D06G025        1    685     5     5      1     3   E5 Homozygote
D06G025        2     18     4     6      4     4              E1
D06G025        2     25     6     6      5     6              E4
D06G025        2    469     4     6      2     5 E3 Heterozygote
D06G025        3     52     4     6      6     6              E1
```

And here is the corresponding summary file:

```
Locus    Genotypes  Errors  Overall_rate Obs_E1 Obs_E2 Obs_E3 Obs_E4 Obs_E5
D06G025       1497      59         0.039  0.020  0.034  0.004  0.059  0.004
```

The last 5 columns refer to the percentage of errors actually introduced in each error category.

## 12.10 Input menu item: Untyped pedigree exclusion option:

This specifies the minimum number of typed members a pedigree should have in order to be included in the output. Selecting this option displays the following sub-menu:

```
==========================================================
Untyped pedigree exclusion options:
0) Done with this menu - please proceed.
 1) Omit any completely untyped pedigrees
*2) Include all pedigrees whether typed or not
 3) Exclude pedigrees not fully typed at one or more markers
 4) Exclude any pedigree with 1 or less marker-typed people
```

## 12.11 Input menu item: Upper limit for squared deviation between input and observed allele frequencies

This is a floating-point value that defines when to flag discrepancies between observed and input allele-frequencies. The smaller this value, the more sensitive it is to such differences. Since the squared sum of differences between input and observed frequencies is expected to be less than 1.0, any value 1 and above would automatically disable flagging. By default flagging is turned off. **Note:** This menu item is not displayed unless a frequency file is specified in the input menu.

See also Mega2’s error checking procedure for allele-frequencies.

## 12.12 Input menu item: Unknown allele and affection definition

This is read in as a string value and compared against affection phenotypes and marker genotypes for an exact match. The unknown allele and affection status indicator is used only for Mega2 files.

## 12.13 Input menu item: Maximum number of alleles per marker

```
==========================================================
              Mega2 4.7.1 Maximum alleles per marker menu:
==========================================================
0) Done with this menu - please proceed 
*1) 2 alleles (2 bits/marker) 
 2) 255 alleles (2 bytes/marker)  
 3) 256 or more alleles (16 bytes/marker) 
Select from options 0-3 >
```

In the first case, each genotype is represented internally with two bits; this is the same as the PLINK compressed binary “bed” representation. This technique does not provide enough space to represent microsatellite markers or half-typed genotypes (where one allele is unknown). For these data sets, the second compression option should be adequate. It provides for up to 255 alleles. The final compression option is left for historical comparisons - it should never be necessary.

Note that in the 2 byte mode, if the input data are in Linkage format, all allele labels are integers used as is. This means that an allele with the label ’256’ would not be allowed. However, if the input data are in Mega2 format, then allele labels are treated as strings, and will be recoded to sequential integers; so ’256’ might be allowed if the labeling is sparse.

## 12.14 Errors in input data

### 12.14.1 Problems with locus data

1. Incomplete locus records and invalid entries:

   Mega2 checks the locus file for invalid and incomplete locus data, following the linkage format specifications. If a names file is used, it checks the marker codes, and that there are at least two entries on each line.   
   Incomplete records are considered to be fatal errors, i.e. Mega2 will terminate upon encountering such errors.
2. Sum of input allele frequencies for each marker

   Mega2 checks the total allele-frequency for each marker to check that the sum is 1.0 (or a tiny fraction of 1.0).
3. Total squared deviation between observed and input allele frequencies

   Mega2 scans the observed genotype data and computes observed allele frequencies over full-typed as well as half-typed individuals to see if these match input frequencies within a certain tolerance. The tolerance value is decided by item 10 of the input menu.   
   This tolerance value can be set to a large value to avoid warnings.

   Both these errors are considered to be non-fatal, i.e., mega2 will stop and warn the user, with the option to continue.
4. Markers without locus positions

   Marker loci in the map file are matched against those in the locus file for missing names from either file. A warning is issued in this case. This is also a non-fatal error. Since many options require map positions, recombination fractions, and some even require loci to be ordered from the closest to the furthest from the p-ter, Mega2 will notify users if marker loci do not have non-zero map locations.  
   Depending on the analysis, this can cause mega2 to terminate, but such errors are usually considered to be non-fatal.

### 12.14.2 Problems with pedigree data.

Mega2 reports problems such as incomplete pedigree records, invalid sex and proband fields, and missing parent records. In most cases, Mega2 will read in the entire pedigree file, and attempt to identify as many errors as it can. Here are a list of errors flagged by Mega2.

1. Father is not male or mother is not female

   Linkage format pedigree file format assigns the first parent to be the father, and the second parent to be the mother. Thus, Mega2 will check for the necessary gender according to this rule. This is flagged as a fatal error because in most cases, subsequent analysis with the output data may not run.
2. One or both parents are missing

   Most pedigree analysis programs require that individual records for both parents are included within the pedigree data, therefore, so does Mega2. Missing parental records are fatal errors.
3. Unknown sex

   1 and 2 are the only values allowed in the sex field, any other value is flagged as a fatal error.
4. Corrupt or incomplete records

   Depending on the number and type of loci given in the locus data file, Mega2 expects each line of the pedigree file to have a certain number of entries. It does allow extra entries at the end, which are skipped. Thus, if The record does not have all the necessary fields, Mega2 will generate error messages like:

   Pedigree 100: entry record 3 is corrupt or incomplete

   \endverbatim
5. Mendelian inconsistencies

   Mega2 performs a set of simple checks to identify inconsistent genotypes such as more than 4 unique allele labels within a sibship (3 unique labels for a sex-linked marker), and obvious Mendelian inheritance rule violations, e.g. offspring’s alleles do not match the parent’s alleles, or a male is a heterozygote at a sex-linked marker.  
   These checks do not guarantee that the pedigree data is free of genotype inconsistencies, specially if one or more parents are untyped, however, these ensure that the output files are acceptable to many of the target analysis programs. The user is encouraged to use a more rigorous testing program such as Pedcheck or Mendel once Mega2 has tested for the simpler errors.  
   The user is offered the choice of setting inconsistent genotypes to unknowns.
6. Half-typed individuals

   Individuals with only one known allele are detected, and the user has the choice of setting these individuals’ genotypes to unknown.

   Genotypes on mitochondrial markers: Although an individual is expected to be homozygous at mitochondrial markers, and also have inherited the mother’s genotype exclusively, there have been known to be deviations from this inheritance pattern. Therefore, for the present, Mega2 only reports genotypes which are heterozygous, or different from the maternal genotype, it does not flag these as Mendelian inheritance errors, nor are these reset along with other Mendelian inconsistencies.
7. Unconnected pedigrees with the same pedigree ID

   Mega2 checks that the entire pedigree is one connected structure, i.e. each individual has either a parent or an offspring in that pedigree. Unconnected pedigrees are considered non-fatal errors when processing a post-makeped format pedigree file, and a fatal error if the file is a pre-makeped format file, and the selected output option requires Mega2 to break loops.

### 12.14.3 Genotype reset menu

This menu is displayed for Mega2 to look for problems with the pedigree data. It will display all four options or a subset depending on which problems were detected.

```
==========================================================
Specify whether to reset poorly typed individuals and families: ==========================================================
0) Done with this menu - please proceed
 1) Set half-typed genotypes to unknown [no ].
    If "no" is indicated, half-typed genotypes
    will not be looked for.
 2) Set all genotypes to unknown within entire pedigrees
    at each Mendelianly-inconsistent locus? [no ]
    If "no" is indicated, Mendelianly-inconsistent loci
    will not be looked for.
 3) Set out-of-bound genotypes to unknown? [no ]
    If "no" is indicated, out-of-bound genotypes
    will not be looked for. 
 4) EXIT Mega2.
```

Item 3) only appears for linkage format inputs. In addition item 1) is not possible for PLINK binary inputs that do not allow half-typed genotypes to be represented. If you have already run your data through a program that checks for these consistency issues and not there are none, it will be faster to not request that Mega2 also look for these issues.

# 13 The analysis menu

After all the input files and the chromosome number have been specified, then one proceeds to the Analysis menu, which presents a variety of different conversion options:

```
===========================================================
                 ANALYSIS MENU  
========================================================== 
 1 SimWalk2 format                 21 MLBQTL format
 2 Vintage Mendel format           22 SAGE format
 3 ASPEX format                    23 Pre-makeped format
 4 GeneHunter-Plus format          24 Merlin/SimWalk2-NPL format
 5 GeneHunter format               25 PREST format
 6 APM format [DISABLED]           26 PAP format
 7 APM-MULT format [DISABLED]      27 Merlin format
 8 Create nuclear families         28 Loki format
 9 SLINK format                    29 Mendel format
10 SPLINK format                   30 SUP format
11 Homogeneity analyses            31 PLINK format
12 SIMULATE format                 32 CRANEFOOT format
13 Create summary files            33 Mega2  format
14 Old SAGE format                 34 IQLS/Idcoefs format
15 TDTMax analyses [DISABLED]      35 FBAT format
16 SOLAR format                    36 PANGAEA MORGAN format
17 Vitesse format                  37 Beagle format
18 Linkage format                  38 Eigenstrat format
19 Test loci for HWE               39 Structure format
20 Allegro format                  40 PSEQ format
                                 
Select an option between 1-40 >
```

**Overview:**  
Note that we can not provide in depth documentation for each of the programs for which Mega2 can create files; in fact, we assume that the user will read the documentation of the appropriate program. Also, since many of these external programs are written by others, it is possible (and perhaps likely) that Mega2 will be out of sync with a newly updated version of one of these programs. If you notice this, please notify us as soon as possible.

In brief, the various options in the Analysis menu are listed below. Note that in most cases, in addition to generating appropriately re-formatted files, Mega2 also generates a C-shell script that will automatically run the desired program. However, these C-shell scripts will only function correctly if the needed programs (with the appropriate names) are in your path and installed properly on your workstation.

## 13.1 Create SimWalk2 format files

This Analysis Menu option creates the files needed for haplotyping, parametric analysis, non-parametric analysis, or IBD estimation via the SIMWALK2 program.   
Name: SIMWALK2 2.82  
Web: https://watson.hgen.pitt.edu/register  
Primary reference(s): (Sobel and Lange 1996)

## 13.2 Convert to Vintage Mendel format

This Analysis Menu option creates output files in the old Vintage Mendel format.  
NOTE: This option will also place the chromosome and map position information into the locus data file, as required by the RELPAIR program by Michael Boehnke and William L. Duren (Boehnke and Cox 1997).   
Name: Vintage Mendel  
Web: http://www.genetics.ucla.edu/software/  
Primary reference(s): (Lange et al. 1988)

## 13.3 Convert to ASPEX format

This Analysis Menu option creates files in the appropriate format for analysis via the many modules (sib\_ibd, sib\_tdt, sib\_phase, sib\_map) of the ASPEX program. There is an option to convert larger pedigrees to their component nuclear families if needed.  
Name: ASPEX  
Web: http://aspex.sourceforge.net/  
Primary reference(s): (Hinds and Risch 1996)

## 13.4 Convert to GeneHunter-Plus format

This Analysis Menu option creates the files needed for analysis via the GeneHunter-Plus program.  
Name: GeneHunter-Plus  
Web: http://galton.uchicago.edu/genehunterplus/  
Primary reference(s): (Kong and Cox 1997)

## 13.5 Convert to GeneHunter format

This Analysis Menu option creates the files needed for analysis via the GeneHunter program.  
Name: GeneHunter  
Web: http://www.broadinstitute.org/ftp/distribution/software/genehunter/  
Primary reference(s): (Kruglyak et al. 1996)

## 13.6 Convert to APM format [DISABLED]

This Analysis Menu option creates the files needed for analysis via the Affected Pedigree Member method (single marker analyses).  
Name: APM and APM Mult  
Web: https://watson.hgen.pitt.edu/register  
Primary reference(s): (Weeks and Lange 1988)

## 13.7 Convert to APM MULT multiple locus format [DISABLED]

This Analysis Menu option creates the files needed for analysis via the multilocus version of the Affected Pedigree member method.

## 13.8 Create nuclear families

This Analysis Menu option breaks down larger pedigrees into their component nuclear families (parents plus children), and output the resulting locus and pedigree files in LINKAGE format.

## 13.9 Convert to SLINK format

This Analysis Menu option creates the files needed for input into the simulation program SLINK.  
Name: SLINK, FASTSLINK (Use FASTSLINK instead of SLINK)  
Web: https://watson.hgen.pitt.edu/register  
Primary reference(s): (Ott 1989; Weeks et al. 1990)

## 13.10 Convert to SPLINK format

This Analysis Menu option creates the files needed for linkage analyses using affected sib-pairs as implemented in the SPLINK program by David Clayton.  
Name: SPLINK  
Web: http://www-gene.cimr.cam.ac.uk/clayton/software/  
Primary reference(s): (Holmans 1993)

## 13.11 Set up for homogeneity analyses

This Analysis Menu option creates the files needed to compute lod scores under heterogeneity, using component programs from the ANALYZE package.  
Name: ANALYZE package  
Web: http://www.helsinki.fi/~tsjuntun/linkage/analyze/  
Primary reference(s): (Terwilliger 1996)

## 13.12 Convert to SIMULATE format

This Analysis Menu option creates the files needed for input into the simulation program SIMULATE.  
Name: SIMULATE  
Web: ftp://linkage.rockefeller.edu/  
Primary reference(s): (Terwilliger et al. 1993)

## 13.13 Create summary files

This Analysis Menu option creates summary files providing information about the segregation of the trait, counts of affected relative pairs and affected sibling pairs, marker allele frequencies (including observed and expected heterozygosity), genotyping success rate, and phenotype counts summaries. There is also an option to create counts of alleles and genotypes within categories (affection status categories or liability class categories).  
  
The different summary options can be selected using the Summary file option sub-menu:

```
Selection Menu: Summary file options
1) Create segregation and relative count summary files.
2) Create allele frequency summary table.
3) Count alleles and genotypes within groups.
4) Create genotyping success rate summary.
5) Create quantitative phenotype summary.
Enter selection: 1 - 5 >
```

## 13.14 Convert to Old SAGE format

This Analysis Menu option creates the files needed for analysis via the SAGE package. This supports the old FORTRAN-like SAGE format (rather than the new more elegant format now available in beta-version).   
Name: SAGE  
Web: http://darwin.cwru.edu/sage/index.php  
Primary reference(s):

## 13.15 Set up for TDTMax analyses[DISABLED]

This Analysis Menu option creates the files needed for analysis via the TDTMax program.  
Name: TDTMax  
Web: Current location unknown   
used to be http://www.rdg.ac.uk/AcaDepts/sn/wsn1/dept/APM.html  
Primary reference(s): (Morris et al. 1997)

## 13.16 Convert to SOLAR format

This Analysis Menu option creates the files needed for analysis via the SOLAR package.  
Name: SOLAR  
Web: http://txbiomed.org/departments/genetics\_detail.aspx?p=37  
Primary reference(s): (Blangero and Almasy 1997; Almasy and Blangero 1998)

## 13.17 Convert to Vitesse format

This Analysis Menu option creates files in LINKAGE format for analysis with Vitesse. Note it also sets up shell-scripts that automate MLINK-like and LINKMAP-like analyses.  
  
Name: Vitesse  
Web: https://watson.hgen.pitt.edu/register  
Primary reference(s): (O’Connell and Weeks 1995)

## 13.18 Convert to Linkage format

This Analysis Menu option creates files in LINKAGE format. This is useful for reordering loci or selecting subsets of loci from large files.  
  
Name: LINKAGE  
Web: ftp://linkage.rockefeller.edu/  
Primary reference(s): (Lathrop and Lalouel 1984; Lathrop et al. 1986; Lathrop and Lalouel 1988)

## 13.19 Test loci for Hardy-Weinberg Equilibrium

This Analysis Menu option permits one to test markers for Hardy-Weinberg equilibrium using either the “hwe” program by Guo and Thompson (1992) or the “Gen” program by Lazzeroni and Lange (1997).  
  
Name: hwe  
Web:  
Authors: Guo and Thompson  
  
Name: Gen  
Web:  
Authors: Lazzeroni and Lange  
  
Name: Exact test for bialleleic markers from the R genetics library  
Web: http://cran.r-project.org/web/packages/genetics/index.html  
Authors: Gregory Warnes and Friedrich Leisch   
  
Name: Chi-sq test for bi- and multi-allelic markers from the R genetics library  
Web: http://cran.r-project.org/web/packages/genetics/index.html  
Authors: Gregory Warnes and Friedrich Leisch   
  
Name: Hardy-Weinberg equilibrium test from Mendel   
Web: http://www.genetics.ucla.edu/software/  
Authors: Lange et al.

## 13.20 Convert to Allegro format

This Analysis Menu option creates the files needed for analysis via the Allegro program. Further information is available at the URL below.   
Name: Allegro  
Web: http://www.decode.com/software/   
Primary reference(s): Allegro, a new computer program for multipoint linkage analysis; (May 2000, Gudbjartsson DF, Jonasson K, Frigge ML, Kong A)

## 13.21 Convert to MLBQTL format

This Analysis Menu option creates the files needed for the analysis of quantitative traits as well as affection status loci via the Maximum-Likelihood-Binomial extension of the GENEHUNTER program. Please make sure that quantitative data are normalized, as required by the MLBQTL program.   
Name: MLBQTL  
Web: http://www.hgid.net/hgid/site/site.php?rubr=9   
Primary reference(s): (Alcais A, Abel L. 1999)

## 13.22 Convert to SAGE format

This Analysis Menu option creates the files needed for using SAGE 4.0 and up version. Currently, the user has to manually add sections required for running various SAGE programs to the bottom of the parameter file. The pedinfo program can be run as is.   
Name: S.A.G.E.  
Web: http://darwin.cwru.edu/   
Primary reference(s):

## 13.23 Convert to pre-makeped format

This Analysis Menu option will output pedigree and locus files where the pedigrees are in pre-makeped format. If the input pedigree file was in a post-makeped format, this option will also connect together loops in the output.

## 13.24 Setup for Merlin-SimWalk2 combined analysis

This Analysis Menu option sets up one set of output data files which can be run through Merlin’s NPL analysis option, and a second set which can be run through SimWalk’s NPL option. It also sets up the requisite scripts that run these two programs in tandem, feeding in NPL results from Merlin to SimWalk2. To use this option, the latest versions of Merlin (0.10.2 or higher) and Simwalk2 (2.89 or higher) are required.   
Name: Merlin and SimWalk2  
Web: http://www.sph.umich.edu/csg/abecasis/Merlin and https://watson.hgen.pitt.edu/register

## 13.25 Convert to PREST format

This Analysis Menu option sets up pedigree, chromosome, control and script files for analyzing with PREST (Pedigree RElationship Statistical Test) version 3.0.   
Name: PREST  
Web: http://galton.uchicago.edu/~mcpeek/software/prest/

## 13.26 Convert to PAP format

This Analysis Menu option sets up the files necessary for running PAP (Pedigree Analysis Package) including the triplets file (trip.dat), the phenotypes file (phen.dat), the control file (header.dat), and the locus file (popln.dat).   
Name: PAP  
Web: http://hasstedt.genetics.utah.edu/

## 13.27 Convert to Merlin format

This Analysis Menu option sets up the files necessary for running Merlin including the pedigree, locus and map files.   
Name: Merlin  
Web: http://www.sph.umich.edu/csg/abecasis/Merlin

Please note that Merlin requires a sex-averaged map as well when the user sets up for sex-specific analysis (Value\_Genetic\_Distance\_SexTypeMap = 193↓).

## 13.28 Convert to LOKI format

This Analysis Menu option sets up the files necessary for running LOKI including the pedigree file, control files, and link files.   
Name: Loki   
Web: http://www.stat.washington.edu/thompson/Genepi/Loki.shtml

## 13.29 Convert to Mendel format

This Analysis Menu option sets up the files necessary for running Mendel (version 7 and later) including per-chromosome pedigree file, locus, map, penetrance, variable and control files.   
Name: Mendel   
Web: http://www.genetics.ucla.edu/software/  
Primary reference(s): (Lange et al. 1988)

## 13.30 Convert to SUP format

This Analysis Menu option sets up files for simulation of marker data with the SUP simulation program. It sets up a SLINK format pedigree and locus file which act as templates to simulate inheritance patterns, and a SUP formatted locus file which contain the actual marker loci to be simulated. An C-shell script is created to run the analysis.   
If the pedigree data contains either loops (premakeped format) or loop-breakers (post-makeped format), then the SLINK pedigree file will have its loops broken. However, SUP requires loops to be reconnected so that the simulated genotypes reflect the fact that some individuals are in fact copies of other individuals. Therefore a set of Mega2 formatted files are created to reconnect loops inside the pedigree output file “pedfile.dat” that is created by SLINK, and output a new pedigree file for SUP. The analysis script contains commands to invoke Mega2 to reconnect loops.

**Caution:** Files created for SUP should not have loops reconnected, but the loops do need to be reconnected after running SLINK and before running SUP.

If the loops are broken, then ’slink’ is happy, but SUP itself does not do the correct thing, because the ’sup.\*.sh’ script now needs another step, which is to use Mega2 to reconnect the loops *after* running SLINK and *before* running SUP, as the SUP documentation states:

"LOOPS:

If there are inbreeding loops in your pedigrees, you have to select loop-breakers prior to creating the simped.dat file that SLINK needs (by use of makeped if you have a list of loop breakers, or maybe by use of Mega2, which can select them for you). In SLINK’s output traitfile.dat, the loops are still broken, and prior to using SUP, you need to reconnect them. Following the suggestion of a reviewer, by far the easiest way is to use Mega2. The file traitfile.dat can be used as one of Mega2’s required input file, and by choosing to create, say, a "Pre-makeped" format file in the analysis menu, Mega2 takes care of reconnecting the pedigrees. You can then use the output created by Mega2 along with the -pre <pedfile> flag."

Name: SUP  
Web: http://mlemire.freeshell.org/software.html   
Primary reference(s): Lemire M (2006) SUP: An extension to SLINK to allow a larger number of marker loci to be simulated in pedigrees conditional on trait values. BMC Genet. 7:40   
SLINK and FASTSLINK references

## 13.31 Convert to PLINK format

This Analysis Menu options sets up pedigree, genotype, phenotype and map files in PLINK format. If output consists of a single trait, then this would be included inside the pedigree file, and phenotype file is created. For multiple traits, the pedigree file contains a dummy trait column, and phenotypes are written out into a separate file. When the ’lgen’ PLINK output file option is chosen, the genotype file is in **long format** (lgen) with a single genotype in every line. There are two binary output file options, which produce files that are much more compact than the lgen format. PLINK requires a physical map, and only our Mega2 format allows physical maps, therefore this option can only be used with Mega2 format files.   
Name: PLINK  
Web: http://pngu.mgh.harvard.edu/~purcell/plink/   
Primary reference: Purcell S, Neale B, Todd-Brown K, Thomas L, Ferreira MAR, Bender D, Maller J, Sklar P, de Bakker PIW, Daly MJ & Sham PC (2007) PLINK: a toolset for whole-genome association and population-based linkage analysis. American Journal of Human Genetics, 81.

## 13.32 Convert to CRANEFOOT format

This Analysis Menu option sets up pedigree and control files for each chromosome in Cranefoot format for drawing pedigree diagrams using the Cranefoot program. The user can select affection status or quantitative traits as well as markers in order to display phenotypes and genotypes along with each individual. Each individual can also be shaded according to her affected status based on their primary affection trait (as selected by the user via a menu). In addition, you can choose to shade the individuals according to their genotyped status (black = genotyped, unshaded = not genotyped). A shell script is also created to run Cranefoot on all output files, or you can choose to have separate shell scripts created for each chromosome.   
Name: Cranefoot  
Web: http://www.finndiane.fi/software/cranefoot/   
Primary reference: Mäkinen et al. (2005) High-throughput pedigree drawing. Eur J Hum Gen 13:987-989.

## 13.33 Convert to Mega2 format

This Analysis Menu option creates pedigree, names, map, frequency, and penetrance files in Mega2 format. If the input pedigree file is pre-makeped, then so is the output file, and vice-versa. This can be used instead of l2a.py, provided that your pedigree and person identifiers are all integers, since Mega2 makes this assumption when reading in Linkage format files. It possesses a few advantages over the Python converter, namely, you can make use of Mega2’s allele-frequency and recoding functionalities in order to produce frequency and penetrance files, there is no omit file, since the output was already produced using one, and the corresponding genotypes reset. Additionally, You can use Mega2’s chromosome, locus and trait selection functions to slice and dice the data, to create chromosome and trait-specific output files instead of creating a single set of files.

## 13.34 Convert to IQLS/Idcoefs format

This Analysis Menu option creates pedigree, marker, and parameter files for the IQLS program, as well as pedigree and study files for the Idcoefs program. In addition, it creates an executable shell file that will first run the Idcoefs program, and then run the IQLS program. Note that IQLS only supports two-allele markers and needs to know the physical positions of the markers. Thus, this option should only be run on input data in Mega2 format that contains two-allele markers, at least one affection status trait, and where a physical map has been specified. The output IQLS marker file has to contain only character allele names, so if your input marker has numbered alleles 1 and 2, these will be remapped to character alleles A and G respectively.

If a marker was originally input with text allele labels in the input Mega2 pedigree file, then those original allele labels are used on output into the IQLS marker file, and the strand orientation is set to ’+’.

If a marker was originally input with numeric allele labels, then it is output using the dummy alleles ’A’ and ’G’, and the strand orientation is set to ’-’.

As far as I can tell, it appears that the orientation information is not used by the IQLS program.

IQLS requires that for each run, all the markers should be on a single chromosome. So to analyze more than one chromosome, a separate run must be performed for each chromosome. Accordingly, when multiple chromosomes are selected, Mega2 will set up chromosome-specific sets of files.

Name: IQLS Web: http://galton.uchicago.edu/~mcpeek/software/IQLS/index.html   
Primary references: Wang, Z., and McPeek, M.S. (2009). An incomplete-data quasi-likelihood approach to haplotype-based genetic association studies on related individuals. J. Am. Stat. Assoc. 104, 1251-1260; Wang, Z., and McPeek, M.S. (2009). ATRIUM: Testing untyped SNPs in case-control association studies with related individuals. Am. J. Hum. Genet. 85, 667-678.  
Name: Idcoefs Web: http://home.uchicago.edu/~abney/abney\_web/Software.html   
Primary reference: Abney M, McPeek MS, Ober C (2000) Estimation of variance components of quantitative traits in inbred populations. Am J Hum Genet 66:629–650.

## 13.35 Convert to FBAT format

This Analysis Menu option creates files for analysis with FBAT. By default, Mega2 chooses to use FBAT with an explicit “map” file. The additional marker position information in the “map” file is needed for the plots that will be produced. Note: Mega2 also sets up shell-scripts that automate running various analyses available under the FBAT program. Several scripts may be produced: one to perform the analysis commands (See the FBAT manual for more details.) and others to load into FBAT the files required for a particular (or several) chromosome(s), traits and phenotypes. The user is expected to revise the primary script with the analysis commands for his/her particular needs, but should not need to change any of the other scripts. There is also a script that will run all the other scripts.  
  
Name: FBAT  
Web: http://www.biostat.harvard.edu/fbat/default.html  
Primary reference(s): Laird, N., Horvath, S. and Xu, X. (2000) "Implementing a unified approach to family based tests of association." Genet Epidemiol 19(Suppl 1): S36-S42  
Documentation reference(s): Nan M. Laird (laird@hsph.harvard.edu), Family-Based Association Tests and the FBAT-toolkit, Horvbath\_FBAT\_Manual\_2009.pdf

## 13.36 Convert to Morgan format

“MORGAN (Monte Carlo Genetic Analysis) is a collection of programs and libraries developed at the University of Washington under the PANGAEA (Pedigree Analysis for Genetics and Epidemiological Attributes) umbrella. This software implements a number of methods for the analysis of data observed on members of a pedigree, with the main programs implementing Markov Chain Monte Carlo (MCMC) methods.” (Taken from the tutorial introduction.) More details are provided in section 25.36↓,

The suboption to the Morgan analysis request specifies which analysis of those listed below is desired. Mega2 produces the “ped”, “marker” and a template “par” file to perform the selected analysis. The user is expected to improve/change the “par” file to his/her specific needs. Shell scripts are created to run the analysis on the “par” file for all the traits and chromosomes selected.

Name: Morgan: pedcheck, kin, translink, lm\_linkage, lm\_bayes, lm\_ibdtests, ...

Web: http://www.stat.washington.edu/thompson/Genepi/MORGAN/Morgan.shtml

Primary references:

E. A. Thompson (1995) Monte Carlo in Genetic Analysis. Technical report No. 294, Department of Statistics, University of Washington.

E. A. Thompson (2000) Statistical Inferences from Genetic Data on Pedigrees NSF-CBMS Regional Conference Series in Probability and Statistics. Volume 6. IMS, Beachwood, OH.

Thompson, E. A. (2005) MCMC in the Analysis of Genetic Data on Pedigrees. In Markov Chain Monte Carlo: Innovations and Applications. Pp. 183--216. F. Liang, J-S Wang, and W. Kendall (eds). Lecture Note Series of the IMS, National University of Singapore. World Scientific Co Pte Ltd, Singapore.

Documentation:

HTML: http://www.stat.washington.edu/thompson/Genepi/MORGAN/morgan311-tut-html/morgan-tut.html

PDF: http://faculty.washington.edu/eathomp/Anonftp/PANGAEA/MORGAN/morgan-tut/morgan311-tut.pdf

## 13.37 Convert to Beagle format

This option creates files for analysis with Beagle. For further information please see the section concerning detailed information on Beagle Analysis 25.37↓.

**Important:** Note that to get correct results when converting to Beagle, your input data must contain the appropriate data. If you wish to set up data in the “unphased unrelated” Beagle format, then your input data must consist of unrelated case/control individuals. If you wish to set up data in the “unphased trio” Beagle format, then your input data must consist of parent-child trio families with unaffected parents and affected children.

Name: Beagle

Web: http://faculty.washington.edu/browning/beagle/beagle.html

Primary references:

If you use BEAGLE in a published analysis, please report the BEAGLE version used and cite the appropriate publication or publications listed below. Please check http://faculty.washington.edu/browning/beagle/beagle.html for the most up-to-date citations.

BEAGLE’s fastIBD method is described in

B L Browning and S R Browning (2011) A fast, powerful method for detecting identity by descent. The American Journal of Human Genetics 88:173-182.

BEAGLE’s methods for detecting homozygosity-by-descent and identity-by-descent are described in

S R Browning and B L Browning (2010) High-resolution detection of identity by descent in unrelated individuals. The American Journal of Human Genetics 86:526-539.

BEAGLE’s methods for calling genotypes from genotype likelihood data are described in

B L Browning and Z Yu (2009) Simultaneous genotype calling and haplotype phase inference improves genotype accuracy and reduces false positive associations for genome-wide association studies. The American Journal of Human Genetics 85:847-861.

BEAGLE’s methods for imputing ungenotyped markers and phasing parent-offspring trios are described in

B L Browning and S R Browning (2009) A unified approach to genotype imputation and haplotype phase inference for large data sets of trios and unrelated individuals. Am J Hum Genet 84:210-223.

BEAGLE’s methods for inferring haplotype phase or sporadic missing data in unrelated individuals are described in

S R Browning and B L Browning (2007) Rapid and accurate haplotype phasing and missing data inference for whole genome association studies using localized haplotype clustering. Am J Hum Genet 81:1084-1097.

BEAGLE’s methods for association testing are described in

B L Browning and S R Browning (2007) Efficient multilocus association mapping for whole genome association studies using localized haplotype clustering. Genet Epidemiol 31:365-375.

BEAGLE’s haplotype frequency model was first described in:

S R Browning (2006) Multilocus association mapping using variable-length Markov chains. Am J Hum Genet 78:903-13.

Documentation: See http://faculty.washington.edu/browning/beagle/beagle.html#download

## 13.38 Convert to Eigenstrat format

This Analysis Menu option creates files for analysis with Eigenstrat. For further information please see the section concerning detailed information on Eigenstrat Analysis 25.38↓.

Name: Eigenstrat, part of the Eigensoft package

Web: http://www.hsph.harvard.edu/alkes-price/software/

Primary references:

Price AL, Patterson NJ, Plenge RM, Weinblatt ME, Shadick NA, Reich D. Principal components analysis corrects for stratification in genome-wide association studies. Nat Genet. 2006 Aug;38(8):904-9. Epub 2006 Jul 23. PubMed PMID: 16862161.

Patterson N, Price AL, Reich D (2006) Population Structure and Eigenanalysis. PLoS Genet 2(12): e190. doi:10.1371/journal.pgen.0020190

Documentation: http://www.hsph.harvard.edu/alkes-price/software/

## 13.39 Convert to Structure format

This Analysis Menu option creates files for analysis with Structure. For further information please see the section concerning detailed information on Structure Analysis 25.39↓.

Name: Structure

Web: http://pritch.bsd.uchicago.edu/structure.html

Primary references:

Pritchard JK, Stephens M, Donnelly P. Inference of population structure using multilocus genotype data. Genetics. 2000 Jun;155(2):945-59. PubMed PMID: 10835412; PubMed Central PMCID: PMC1461096.

Falush D, Stephens M, Pritchard JK. Inference of population structure using multilocus genotype data: linked loci and correlated allele frequencies. Genetics. 2003 Aug;164(4):1567-87. PubMed PMID: 12930761; PubMed Central PMCID: PMC1462648.

Falush D, Stephens M, Pritchard JK. Inference of population structure using multilocus genotype data: dominant markers and null alleles. Mol Ecol Notes. 2007 Jul 1;7(4):574-578. PubMed PMID: 18784791; PubMed Central PMCID: PMC1974779.

Hubisz MJ, Falush D, Stephens M, Pritchard JK. Inferring weak population structure with the assistance of sample group information. Mol Ecol Resour. 2009 Sep;9(5):1322-32. doi: 10.1111/j.1755-0998.2009.02591.x. Epub 2009 Apr 1. PubMed PMID: 21564903; PubMed Central PMCID: PMC3518025.

Documentation: http://pritch.bsd.uchicago.edu/structure.html

## 13.40 Convert to PSEQ (PLINK/SEQ) format

This Analysis Menu option creates files for analysis with PLINK/SEQ. For further information please see the section concerning detailed information on PLINK-SEQ Analysis 25.40↓.

Name: PSEQ

Web: http://atgu.mgh.harvard.edu/plinkseq/

Primary references:

TBD

Documentation: http://atgu.mgh.harvard.edu/plinkseq/pseq.shtml

# 14 The Missing Value Menu

This menu is used to indicate what values in the input data represents a missing value. Quantitative traits may have a different missing value for this purpose than Affection Status traits. In addition, when Mega2 produces outputs for analysis programs, it allows you to specify what missing value code should be used. Again a different value can be used for Quantitative traits vs. Affection Status traits. Typically, the input dictates the default missing values. Below is what one would see if the input files are in LINKAGE format. Mega2 input format would use “NA” for both input missing values, and PLINK input format uses -9 by default. Output missing values can present problems. Some analysis programs dictate the value to be used to represent a missing value. In this case, the options “3)” and “4)” in the Missing Value menu would be written “#)” and “#)” respectively indicating that you can not change these output missing values. All four values are copied to the batch file that is generated.

```
==========================================================
              Mega2 4.7.1 Missing Value menu:
==========================================================
If it is necessary, specify a different value to indicate that a trait is missing 
both for input to Mega2 and/or output from Mega2.
Note: Output entries that are marked with a "#" can not be changed.
0) Done with this menu - please proceed
 1) Specify input missing value for Quantitative traits:     -999
 2) Specify input missing value for Affection status:           0
 3) Specify output missing value for Quantitative traits:       x
 4) Specify output missing value for Affection status:          x
Select from options 0-4 > 0
```

You may edit these values as you desire in keeping with what is allowed by the analysis program. Mega2 will verify if the value is constrained to be numeric.

After “0)” is typed to exit the menu, a summary is written to the logs as shown below (This case indicates no changes from the default):

```
Quantitative  Input Missing Value  -999
Affection     Input Missing Value "0"
Quantitative Output Missing Value "x"
Affection    Output Missing Value "x"
```

# 15 The allele frequency menu

Allele frequencies for marker loci are estimated from a subset of genotyped individuals in the pedigree file. The user can select which individuals will be selected from the following menu:

```
0) Done with this menu, please proceed.
 1) Genotyped founders only.
*2) Genotyped founders + a randomly chosen genotyped person
    from pedigrees without genotyped founders.
 3) Genotyped founders + genotyped individuals with unique alleles.
 4) All genotyped individuals.
 5) Count half-typed individuals’ alleles. [no]
```

Option 2 is the default choice for individual selection. The selection option takes effect after pedigrees have been excluded as per the “Untyped pedigree selection option” as well as after genotypes have been set to unknown as per the omit file, if one is provided. Note that this was not the case before, recoding took place before these two steps.

For X-linked loci, males have to be represented as homozygotes, as only the first allele is counted.

The meanings of options 2 and 3 are explained below:

**Option 2:** Genotyped founders + randomly chosen genotyped person: If a pedigree does not contain at any genotyped founder, then a random person is selected from the list of genotyped individuals. If a pedigree has only one genotyped person, that person is selected.

Please note that the behavior of option 3 has changed as described below:

**Option 3:** Count all genotyped founders + genotyped individuals with unique alleles: In the presence of untyped founders and rare alleles, non-founders may possess alleles which are not counted by option 2. In this case, the output locus file contains alleles with zero frequencies, which may cause some of the analysis programs to reject the output locus file. Vitesse is one such program. Thus, menu option 3 first goes through all genotyped founders, then randomly selects a genotyped person from each pedigree without genotyped founders, then identifies alleles with 0 counts for each marker. Previously, it did not go through the random-selection process. For each allele with 0 counts, it counts the first person with that allele encountered in each pedigree. The other allele of that person is also counted if that person if the marker is autosomal, or if the person is a female.

**Option 5** is a toggle item, and has to do with whether half-typed individuals should be included in the allele-frequency computation. Half-typed individuals are excluded , i.e. menu item 5 is set to “no” by default.

## 15.1 The recoding process

Affection status and quantitative trait phenotypes are not recoded. For now, it is assumed that these are numeric following the linkage format convention.  
Marker genotypes can be non-numeric. However, many of the analysis options handle only consecutively numbered alleles, therefore, the original genotypes are converted to alleles with numeric labels. The analysis-specific output pedigree and locus files contain recoded allele numbers. Non-numeric allele names are lexically sorted by their value and assigned allele numbers. If, for a marker, the original allele names are numbers from 1 though N , and Mega2 encounters all N alleles within the genotype data, the resulting alleles are NOT recoded, i.e., the original allele numbers are maintained in the output. However, if any allele is missing from the data, renumbering is done to create consecutively numbered alleles.

## 15.2 The recode log file

A summary of the recoding process is stored in the MEGA2.RECODE log file. The option selected from the individual-selection menu is logged. For each marker, the number of individuals counted is recorded, as well as the frequency and number of each allele within the selected subset of individuals. The recode summary file also records the number of half-typed individuals for each marker if this is set to “yes”.

## 15.3 Penetrances for affection status loci

Starting from version 2.5.3, Mega2 also allows the user to specify one disease allele frequency and 3 autosomal penetrance values or 6 sex-specific penetrance values for an affection status locus with a single liability class. The frequency is taken to refer to the second allele, which is considered to be the disease allele. The first three penetrances are assumed to be the female penetrances for genotypes 1/1, 1/2 and 2/2, and the following three are assigned to the male penetrance values. If male penetrances are not specified, female penetrances are used in their place. If frequency and penetrances are not provided, equal allele frequencies (i.e. 0.5 for both alleles), and an incomplete dominant disease model are used (1/1=0.05, 1/2=0.90, 2/2=0.90).   
Affection status loci with multiple penetrance classes: Trait loci denoted type “L” are considered to have more than one liability classes and Mega2 assigns default penetrance values in each class. If the user wishes to apply her own penetrance values in this case, she would first need to create a linkage format locus file via the Pre-makeped format option (analysis option 23) or the Linkage format option (analysis option 18), change the penetrance values within the resulting linkage format locus file (Pdatain.\* or Ldatain.\*), then run Mega2 again with the edited locus data file in order to create the desired output format.

# 16 The locus reordering menu

The locus reordering menu allows the user to reorder the loci into the map order as specified in the map file, or into a user-chosen order. The user can also select subsets of loci from a list of loci.

The reordering menu in version 4.0 allows the user to select and change selections as many times as necessary before she decides to proceed. It has only three ways to select loci. The 4th item allows the user top choose which map to use, if the input map file contains more than one map.

```
       Locus Reordering Menu
0) Done with this menu - please proceed.
*1) Select all loci in map order on chromosome: 5
 2) Select by locus number.
 3) Select loci on multiple chromosomes[]
```

## 16.1 Locus reordering option 1) Select all loci in map order on chromosome.

Option 1 is used to select a single chromosome. By default, it is set to the first chromosome encountered in the input data. If the user selects this item, then the user is prompted to enter a new chromosome number.

If the user hits 0 at this point to proceed, then loci present on this chromosome will be included in the output file, arranged as per their map positions as specified by the input map file.

## 16.2 Locus reordering option 2) Select by locus number.

Option 2 permits the user to select a set of loci from a numbered list by entering their numbers in the desired order. The user may at any time during the selection process type in the character ‘v’ to review the original loci list or ‘o’ to view all the loci he/she has selected. To finalize the selection process the user will include an ‘e’ at the end of the string or on the next input line (e.g. ‘1 3 8-11 e’ is a typical input string and will select the loci at the 1, 3, 8 through 11th positions in the original locus order).

In previous versions of Mega2, during the display of loci via the ’v’ input, the user had to type ’q’ to end the display, then enter locus numbers. In the new version of Mega2, instead of typing ’q’, locus numbers can be entered followed by the carriage-return. This will save the selections from the current screen, then continue onto the display of the next screen-full of loci. This is useful if selections have to be made from different regions on the chromosome, out of many loci, as is often the case with SNP data.  
HINT:   
Several of the output options will complain if markers are not ordered according to their map positions, so this has to be kept track of while re-ordering the loci manually.

## 16.3 Locus reordering option 3) Select marker loci on multiple chromosomes.

Option 3 is used to set up multiple sets of files, one for each chromosome. When Option 4 is chosen, this sub menu appears, if the input map file includes sex-linked markers, allowing users to select markers on human autosomal chromosomes 1-22, all autosomal and sex-linked chromosomes, or specific chromosomes:

```
1) Select all autosomes. 
2) Select all chromosomes. 
3) Select markers on specific chromosomes.
```

Otherwise, only options 2 and 3 are shown:

```
 1) Select all marker loci on all chromosomes. 
 2) Select marker loci on specific chromosomes.
```

As before, the second option lists the available chromosome numbers and allows the user to select specific ones from this list.

The asterisk indicates the current selection. Once you enter a number indicating which map should be used in the output, you will be taken back to the locus reordering menu. The map names are read in from the header of the Mega2 map file.

# 17 Map Selection Menus

When a map file (see section 9.1.3↑) contains multiple genetic or physical maps, the user needs to be able to specify which map is to be used for processing. When running with a batch file, this is done through the use of batch file parameters (see section 92↓). When running in interactive mode, the user will be presented with menus that will allow them to select an input map. If only one genetic map is specified in the map file, then this map will be automatically used and no menu will be presented to the user (as there is no choice to be made).

## 17.1 The Genetic Map Selection Menu

When using a map file, there are three possible types of genetic maps: sex-averaged, sex-specific (male and female), or female (see section 9.1.3↑; Map file).

If sex-averaged, male, and female maps for a map name exist in the input map file, the user will be given the option to use the sex-averaged, sex-specific, or female map. If only the sex-averaged map for a map name exists in the input map file, the user will be given the option to select a sex-averaged map. If the male and female map for a map name exists in the input map file, the user will be given the option to select a sex-specific or female map. If only the female map for a map name exists in the input map file, the user will be given the option to select a female map. For the physical map only one map can be specified per map name.

As an example, if the input map file that is discussed in section 9.1.3↑ is used,

```
Chromosome M.h.a    Name   M.h.m   M2.p    M.h.f  M3.p   M4.k.a   M4.k.m
1          0.0     rs101   0.0     543     0.0    304    0.0       0.0 
1          5.0     rs102   2.0     678     7.0    821    5.0       2.0 
X          0.0     rs231   0.0     743     0.0    912    0.0       0.0 
X          4.0     rs232   1.0     862     6.0    954    4.0       0.0
```

then the following genetic map selection menu would be presented to the user:

```
Genetic map selection menu: 
0) Done with this menu - please proceed 
*1) M: sex-averaged Haldane 
 2) M: sex-specific Haldane
 3) M: female Haldane
 4) M4: sex-averaged Kosambi
With the current option, any X-chromosome processing will 
                         be done with a sex-averaged genetic map 

Select from options 0 - 4 >
```

The input map file has three columns for map ‘M’ (sex-averaged, female, and male). Because of this it is possible for the user to select among three different map types.

The input map file has two columns for map ‘M4’ (sex-averaged, and male). Here, it is only possible for the user to select one map type (sex-averaged), since the male map cannot be used by itself.

There are processing implications for selecting a specific genetic map. A sex-averaged map can be used in processing only the autosomes and the X chromosome. In this case the sex-averaged map positions will be used on the X chromosome as if they were female map positions.

If a sex-specific map is selected, then the male and female positions are used for all autosomal markers, and the female positions are used for all markers on the X chromosome.

If only a female map is selected, then that female map can only be used in processing only markers on the X chromosome.

## 17.2 The Physical Map Selection Menu

As an example, if the input map file that is discussed in 9.1.3↑ is used,

```
Chromosome M.h.a    Name   M.h.m   M2.p    M.h.f  M3.p   M4.k.a   M4.k.m
1          0.0     rs101   0.0     543     0.0    304    0.0       0.0 
1          5.0     rs102   2.0     678     7.0    821    5.0       2.0 
X          0.0     rs231   0.0     743     0.0    912    0.0       0.0 
X          4.0     rs232   1.0     862     6.0    954    4.0       0.0
```

then the following physical map selection menu would be presented to the user:

```
Physical map selection menu: 
0) Done with this menu - please proceed 
 1) M2 
 2) M3 
*3) None
Select from options 0 - 3 >
```

If the analysis mode does not require a physical map, the user will be presented with the ‘None’ option.

# 18 The trait selection menu

The trait selection menu allows the user to select one or more traits and place them before or after the marker loci inside the output files. Some analysis options allow only a single traits locus at a time, in which case the third option is not available. In other cases, only affection status loci are allowed, and the trait selection process will not terminate unless a valid set of traits have been chosen. (See More details on trait selection for specific analysis options.)

Here is what the menu would look like when entered for the first time for the Mendel option which allows more than one trait to be output together.

```
==========================================================
Trait and covariate selection menu:
0) Done with this menu - please proceed.
 1) Create trait-specific files or combine: [Order as specified in item 2]
 2) Trait loci selected: [aff [MARKERS]]
Enter option 0 - 2 >
```

## 18.1 Select multiple trait loci to loop across.

If Option 1 is set to “one trait at a time”, the analysis is carried out with one trait and all marker loci at a time. The output for each trait is stored in a sub-directory (see Output directory selection menu). Trait loci are selected via option 1, and the order in which they are selected does not affect the output, except the order in which each trait is analyzed. to the traits.

## 18.2 Use trait loci in specified order.

If Option 2 is set to “Order specified in option 2”, all traits are combined into a single set of output files. Trait loci appear in the output files in the order they are selected in, with the marker loci inserted at the “[MARKERS]” position . Thus, this option controls the positioning of trait loci with respect to the marker loci.

## 18.3 List of selected trait loci

Option 2 allows to user to specify zero or more trait loci to be used in the analysis. If there is only a single trait locus, and the analysis option requires at least one trait locus, then this menu is skipped, and the trait locus automatically selected. For these options, if no trait loci are present, or the trait locus is not of the correct type (e.g. SimWalk2-NPL requires at least one affection locus, and the data has only QTLs), then Mega2 will stop with an error message.

If option 2 is set to “one locus at a time”, a list of trait loci are displayed, and the user enters 0 or more corresponding numbers. If option 2 is set to “combine”, then the trait list also includes a [MARKER] item. In this case the order of the loci decides the order in which they appear in the output files.

## 18.4 Selection of covariates

The trait selection menu includes a covariate selection item if the data includes QTLs for GeneHunter, Merlin and SOLAR. Here is what the trait menu looks like with the covariates item:

```
Trait and covariate selection menu:
0) Done with this menu - please proceed.
 1) Trait loci selected: [Q1]
 2) Covariates selected: []
 3) Loop over traits or combine traits: [Loop   ]
Enter option 0 - 3 >
```

Traits appearing in the list in option 1 is automatically excluded from the list of covariates.

## 18.5 More details on trait selection.

The following options require at least one affection status trait locus:

Simwalk2: Parametric (Location scores) and Non-parametric linkage   
Aspex  
GeneHunter, GeneHunter-Plus, Allegro, and MLBQTL  
APM and APM-Mult  
SPLINK  
Segregation and liability group counts  
TDTMax  
Vitesse  
FBAT

Thus, if there is only a single trait locus in the input data, this trait locus is automatically selected and the trait selection menu skipped.

The following do not allow quantitative trait loci:

Simwalk2 options  
Aspex  
GeneHunter-Plus and Allegro  
APM and APM-Mult  
SIMULATE  
Segregation and liability group counts  
TDTMax

Therefore, selecting a quantitative locus for output generates an error message, and the user asked to re-select traits.

The following options can handle quantitative trait loci but require at least one affection status locus. For these options if the user doesn’t specify an affection status locus, a dummy locus is inserted in the output files.

GeneHunter  
MLBQTL

## 18.6 Affection status labels

If one or more affection trait locus has multiple liability classes, then a separate menu lets you choose which status-class combinations to label as “affected”. This is so that options, which do not recognize multiple liability classes can analyze such affection status loci. The list of such options includes SimWalk2, Aspex, Segregation summary, Slink, Splink, PAP, Mendel7+, S.A.G.E. 3.0 and S.A.G.E. 4.0, combined Merlin/SimWalk2 non-parametric linkage, Merlin, and PLINK.

Here is what the menu looks like, for two traits with multiple liability classes:

```
Affection label menu:
     0) Done with this menu - please proceed
     1) TRAIT [2-*]
     2) trt2 [2-*]
     Enter 0 or a trait item between 1-2 >
```

Selecting a trait item allows the user to specify one or more status-class combinations for that trait locus. The default which implies that any person with a status 2 is to be treated as affected, irrespective of the class value.

For example, if the user enters 1, this prompt appears:

```
==========================================================
Found these status-class pairs:
                0-1  (7 entries)
                2-1  (4 entries)
                2-2  (6 entries)
                2-3  (4 entries)
Now enter the "trait status"-"liability class" pairs 
you wish to be considered as "affected" by Mendel7+ format (e.g. 2-1).
You may enter a * in either field as a wild-card 
e.g., 2-* means status 2 and all classes). 
Separate pairs with commas (e.g. 2-1,2-3) >
```

Please also consult the documentation on the corresponding batch item Value\_Affecteds to specify affected labels inside the batch file.

```

```

# 19 Creating plots using Mega2

Our R graphing package nplplot has been designed to generate PDF or postscript files containing statistical analysis results for markers along a chromosome. Thus, R is also required for this to function. Although Mega2 uses this package to plots of results for the four options listed in the Supported options section, it can conceivably be used to plot any type of scores along a set of positions along a chromosome.

The package consists of three functions which are related to the plotting itself, and three other functions that also create output files for adding custom tracks and Genome Graph plots within the UCSC genome browser interface. The three plot functions are nplplot, nplplot.multi and nplplot.old. The other three are functions for creating formatted files for the UCSC genome browser, namely bedplot, genomeplot and prepareplot. These are described in in the Custom tracks section of the documentation.

As mentioned in the installation instructions, the package nplplot should be installed along with the R libraries. Documentation on installing libraries in general is maintained at the CRAN web-site.

For users already familiar with nplplot, note that the nplplot.old function is the old nplplot function, provided only for backward compatibility, to support the old-style plot data file format. The function nplplot.multi replaces nplplot.old , and provides greater flexibility to customize your plots. The new input files are of two types, a **Scores** file that is strictly meant to contain a table of marker names, positions and scores, and a **Header** file, which is an R-language file to set various plot parameters. Examples of these files are provided in the general usage section below.

Installing nplplot from CRAN

The nplplot package is distributed from within CRAN (The Comprehensive R Archive Network). Like any other R add-on package, nplplot can be installed from within R. It does not depend on any other package.

In order to generate the plots from within Mega2, it is not necessary to invoke R. The scripts generated by Mega2 run R in batch mode to create the appropriate postscript or PDF files. Currently PDF files are the default; you need to go into the R plot menu and change output format to postscript, if you wish to generate postscript files.

Supported analysis options

The following Mega2 options can currently create R plots:

- SimWalk2 - Non-parametric analysis
- Allegro
- Merlin-SimWalk2 option
- Merlin’s npl, pairs, and vc analyses
- FBAT

## 19.1 Statistics selection menu

SimWalk2 statistic selection menu

SimWalk2 computes 5 statistics for the NPL LOD score computation (see the SimWalk2 output file STATS-\*.ALL for a description). This is also true of the Merlin-SimWalk2 option. Allegro computes LOD scores separately for 12 different non-parametric statistics. Each is stored in a separate output file named appropriately (e.g. allegro\_linpairs\_spt.01). Therefore, for each analysis option there is a specific statistic selection menu where the user can specify the ones to be included in the plots.

Please note that running SimWalk2 or Allegro will still compute all of their statistics, however, only those selected via this menu will be plotted. Further instructions on how to subsequently modify the selections are provided below.

SimWalk2 statistic selection menu:

```
========================================================== 
R plot statistic selection menu: 
NPL statistics will be automatically plotted into a 
PDF file using R after Simwalk2-NPL 
has been run. 
Please select the statistics to be included in this R plot. 
This list can be later modified in the shell script Rsimwalk2.sh before 
running this script. 
========================================================== 
1) BLOCKS 
2) MAX-TREE 
3) ENTROPY 
4) NPL_PAIRS 
5) NPL_ALL 
========================================================== 
Enter string of statistic numbers (’e’ to terminate) > 1 2 3 e 
Selected statistics: BLOCKS MAX_TREE ENTROPY 
==========================================================
```

In the above example, the user has selected statistic BLOCKS, MAX-TREE, and ENTROPY for plotting. For the Merlin-SimWalk2 option, only statistics NPL\_PAIRS and NPL\_ALL, which correspond to Merlin’s Pairs and All statistics respectively, can be selected.

Merlin statistics selection menu

For Merlin, the statistics list is :

```
========================================================== 
1) ALL 
2) Pairs 
3) VC 
==========================================================
```

Plotting is still restricted to the “one trait at a time” mode of analysis.

Allegro statistics selection menu

The Allegro statistics selection menu is:

```
========================================================== 
R plot statistic selection menu: 
NPL statistics will be automatically plotted into a 
PDF file using R after Allegro 
has been run. 
Please select the statistics to be included in this R plot. 
This list can be later modified in the shell script Rallegro.sh before 
running this script. 
========================================================== 
1) exppairs_mpt Exponential multi-point pairs 
2) exppairs_spt Exponential single-point pairs 
3) expall_mpt Exponential multi-point all 
4) expall_spt Exponential single-point all 
5) linpairs_mpt Linear multi-point pairs 
6) linpairs_spt Linear single-point pairs 
7) linall_mpt Linear multi-point all 
8) linall_spt Linear single-point all 
9) par_mpt:LOD Parametric multi-point LOD scores 
10) par_spt:LOD Parametric single-point LOD scores 
11) par_mpt:HLOD Parametric multi-point heterogeneity LOD scores 
12) par_spt:HLOD Parametric single-point heterogeneity LOD scores 
========================================================== 
Enter string of statistic numbers (’e’ to terminate) >
```

## 19.2 R-plot parameters menu

This menu allows to user to define what to plot, where and how. Here is an example of what the menu looks like:

```
========================================================== 
Customization of graphical output:

** Y-axis min and max values can be enforced by setting "enforce" to yes
   otherwise, these will be set according to the plot data.
** Plot orientation can be toggled between 
   portrait or landscape.
** Format of output file (ps/pdf) is decided by file-name extension (.ps/.pdf).
   If no extension is provided, a pdf file will be created.

==========================================================
R plot parameter selection menu:
0) Done with this menu - please proceed
 1) PDF output file name          RMerlin.06.pdf 	[new]
 2) Minimum Y-axis value                    0.00
 3) Maximum Y-axis value                    3.00
 4) Horizontal cut-off line at              2.00
 5) Plot orientation             [landscape]
 6) Enforce Y-axis bounds even if data does not fit [no ]
Select from options 0-6 (5,6 to toggle, 2,3,4 to change values) >
```

In this example, data containing marker loci on chromosomes 1, 2, and 3 and traits AFF1 and AFF2 is analyzed with the Merlin *-\/-*npl and *-\/-*pairs option. Here, the user has chosen to combine the LOD score curves for the two traits AFF1 and AFF2 on a single plot, and plot the three chromosomes in separate output files.

If you choose to combine the chromosomes into one file, by typing 3 at the prompt, the resulting menu would have some additional choices:

```
========================================================== 
R plot parameter selection menu: 
0) Done with this menu - please proceed 
1) Plot output file name stem RMerlin 
2) Plot traits together in same graph [yes ] 
3) Combine chromosomes into one file [yes] 
4) Minimum Y-axis value 0.00 
5) Maximum Y-axis value 3.00 
6) Horizontal cut-off line at 2.00 
7) Plots per page: number of rows 1 
8) Plots per page: number of columns 1 
9) Plot orientation [portrait] 
10) Enforce Y-axis bounds even if data does not fit [no ] 
Select from options 0-10 (2,3,9,10 to toggle, 4,5,6,8,7 to change values) > 
Options 7 and 8 control the number of columns and rows on a page. 
 Creating plots
```

For each of the three analysis options listed above, the output file name selection menu now includes a switch that can turn the plot creation on or off. If is it turned on, these additional files are created :

- Rsimwalk2.pl or Rallegro.pl (perl file to format SimWalk’s or Allegro’s output appropriately for nplplot.
- Rsimwalk2.sh or Rallegro.sh (C-shell file to run R and create the plots).
- Rsimwalk2.R or Rallegro.R (R-script run by the shell script to generate the plots).

Thus, first the user has to execute either the *npl.\*.sh* or *al\_script.\*.sh* files (these could reside in separate trait directories, if multiple traits are analyzed), then run *Rsimwalk2.sh* or *Rallegro.sh* respectively.

Output

The SimWalk2 output file is called SW2NPL.##.ps where ## stands for the chromosome number or SW2NPL.all.ps if multiple chromosomes are plotted into one file.

The Allegro output file is called Allegro.##.ps. These files can be viewed using a PDF viewing utility such as ghostscript, or printed on a printer directly.

Re-using old Merlin analysis results with the new Rmerlin.pl

You can manually change the Rmerlin.\*.sh scripts by replacing the arguments to the Rmerlin.pl command. The arguments following the -c switch need to be changed as follows:   
-c 2,1 or -c 1,2 need to be changed to -c All,Pairs or -cPairs,All respectively.   
-c1 needs to be replaced by -cPairs or -cAll, depending on what the analysis was.

- Run Mega2 to re-generate the scripts. Then, being careful not to run the merlin.\*.sh scripts simply re-run the Rmerlin.\*.sh scripts.

## 19.3 General usage of nplplot and nplplot.multi

Nplplot functions can also be used from within R. Start R, load in the library, using the command library(nplplot), and you are ready to create your own plots.

Example data files and code is included with the nplplot and nplplot.multi functions. These can be run using the example command available in R. Use these examples to create your own data files for plotting or use the example files given below.

Scores file

```
marker location score1 score2 score3 
d1s228 0.00 0.546 0.345 0.142 
d1s429 1.00 0.346 0.335 0.252 
d1s347 2.00 0.446 0.245 0.342
```

Header file for nplplot.multi

```
# reference line, y-axis minimum and y-axis maximum 
yline <- 2.00 
ymin <- -1.0 
ymax <- 3.00 
# Enforce y-axis bounds? 
yfix <- FALSE 
# Plot subtitle 
title <- "Chromosome 2" 
# Score units 
ylabel <- "LOD Score" 
# Font scaling for legend 
cex.legend <- 0.7000 
# Font scaling for axis 
cex.axis <- 0.9000 
# Tick length for marker labels at the top of plots 
tcl <- -0.3000 
# Use colors 
bw <- FALSE 
# Remove NAs before plotting each curve 
na.rm <- TRUE 
lgndtxt <- c("Trait1", "Trait2", "Trait3") 
ltypes <- c(1,1,1) 
my.colors <- c("black", "red", "green") 
ptypes <- c(0, 0, 0)
```

If you wish to use nplplot only, then these parameters can be passed in as arguments to nplplot. The table of scores can be supplied as a file as well as an R data-frame to nplplot.

# 20 Custom tracks for the UCSC Browser

Overview

The nplplot package now has functions to create custom track files that then can be uploaded into the UCSC Genome Browser for further visualization. From within Mega2, the creation of custom track files is done automatically under the following conditions: 1) an option has been chosen where the creation of R plots is supported; 2) the input files are in Mega2 format; and 3) the map file contains a physical map giving the physical positions of the markers. When these conditions hold, the running of the C shell script to do the analyses and to create the R plots will also automatically create a set of custom track files for the UCSC Genome Browser.

Custom track output files

Mega2 will create four types of output files:

1. BED.01 - this track indicates the names and positions of the markers.
2. BedGraph.01 - this track creates a bar graph of the results.
3. GG.markers.all - this is a Genome Graph file that can be loaded in via the ’Genome Graphs’ interface. This contains results for all chromosomes and uses the marker name and so only lists results at each named marker.
4. GG.positions.all - this is a Genome Graph file that can be loaded in via the ’Genome Graphs’ interface. This contains results for all chromosomes and uses the ’chromosome base’ (e.g. chr1 130000) format style, and so lists results at every position for which a statistic was calculated.

**Notes:**

The BED.\* and BedGraph.\* files are named using extensions indicating the chromosome (e.g., .01, .02, .03, etc). They come in pairs, one pair per chromosome.

The GG.positions.all file will upload much faster into the UCSC Genome Browser than the GG.markers.all file. Presumably this is because the UCSC Genome Browser has to look up positions for each of the markers named in the GG.markers.all file.

When creating your input physical map, it is important to use physical positions from the exact same build that you will be using within the UCSC Genome Browser to display your results. This is particularly important and vital when uploading results from the BED.\*, BedGraph.\*, and GG.positions.all files, as all of these files use and rely on the physical positions as originally provided in the input map file. In contrast, the GG.markers.all file does not contain any physical positions and so is not vulnerable to mismatches between your input physical map positions and the positions used in the UCSC Genome Browser. However, the UCSC Genome Browser will only display results from the GG.markers.all for which it was able to look up the marker name. If it fails to find a marker name for a record, that record will be dropped and not displayed.

Supported analysis options

The following options can currently create R-plots, and, if run on Mega2 input files with a map file containing a physical map, will also create UCSC Custom Track files:

- SimWalk2 - Non-parametric linkage analysis
- Allegro
- Merlin-SimWalk2 option
- Merlin’s npl, pairs and vc analyses

Creation details

The creation of the custom track files is done using R functions that are part of the nplplot package. For further details, please see the R help pages for these functions.

1. bedplot - BED and BedGraph plotting
2. genomeplot - Genome Graph plotting
3. prepareplot - Prepare input data files for plotting

General usage

To use the three track-related functions independently from within R, first run prepareplot. This function can be used to process multiple nplplot format scores datafile, by specifying a common prefix, a list of chromosomes, and a single Mega2-annotated format map file with physical positions for all the required markers and chromosomes. You can then run bedplot or genomeplot on the resulting files to further reformat the data for uploading into the UCSC genome browser.

# 21 Additional Mega2 Output Files

Mega2 produces these additional output files, containing further details of the current run. These files are either stored in a special directory described below, or tagged with the date and time of the run, if Mega2 terminated abnormally in the very early stages of its execution, e.g. if you ran Mega2 at 2:30 pm on June 15, 2004, then you may end up with a files named MEGA2.LOG.2004-6-15-14-30, MEGA2.ERR.2004-6-15-14-30 etc.

## 21.1 Summary file directory

The summary file directory (also referred to as the run-directory), contains the summary files created by Mega2, along with html-versions of these files. This directory is created within the output folder specified in the input menu, and identified by the date and time of the run. For example, in the previous example, if Mega2 ran without problems, you should see a directory named 2004-6-15-14-30.

To turn off saving summary files in a separate directory run mega2 with the *-\/-*nosave option, i.e.

mega2 *-\/-*nosave

mega2 -x

\endverbatim

If saving is turned off, then the MEGA2.LOG, MEGA2.ERROR, MEGA2.KEYS and MEGA2.RECODE files are left within the folder from which Mega2 was run. If you run Mega2 again from within this folder with the *-\/-*nosave option, these files will be overwritten.

This may seem undesirable for normal use, but is a useful space-saving feature when you are running many consecutive runs of Mega2 from within the same folder, as would be the case when running replicates for a simulation study.

## 21.2 MEGA2.LOG

In addition to the sate and time of the last run and input file names, this file is a copy of Mega2’s screen output including summary statistics on the input pedigrees and locus data, the analysis option name, what loci were selected for reordering, pedigree statistics after locus selection, and finally, what output files were produced.

It may also contain other important messages such as families and individuals excluded from analysis, breaking or re-connection of loops in inbred pedigrees, individuals whose genotypes were zeroed out, etc. If there were any warnings or error messages, they are written into this file in addition to the MEGA2.ERROR file.

Note that the backup copy MEGA2.LOG.old is no longer created.

## 21.3 MEGA2.ERR

This file contains all the error and warning messages produced during a single run of Mega2. These messages include problems encountered with the input files, such as inconsistent or invalid phenotype/genotype data (Mendelian inconsistencies, markers named in the locus file but missing from the map file and vice-versa etc.), and if there are problems with creating and writing new files, directories during the execution.

For a detailed list of errors and warnings, see the trouble shooting section . It is a good idea to examine this file carefully and consult the Trouble-shooting documentation, if Mega2 terminates abnormally. We have made all efforts to make the error and warning messages as informative as possible, in order to help the user debug her data. We always appreciate feedback and suggestions for improvement in this area.

## 21.4 MEGA2.BATCH

This is a default batch file created every time Mega2 is run in the interactive mode. See the section 23↓ on Running Mega2 in Batch Mode for details on the format and contents.

## 21.5 MEGA2.KEYS

This file contains a list of input and output pedigree and person identifiers. It relates each input pedigree record to its corresponding output pedigree record. Since, several of the output formats within Mega2 require reordering individuals, or re-assigning pedigree and person IDs, it is difficult for the user to match up the output against the input. Therefore, it was felt necessary to create a mapping between input and output pedigree data. From the key file, the user can identify loops that were broken up or re-connected, or which nuclear pedigrees were created from an extended pedigree. In the case of PAP, for instance, where individuals are assigned completely new IDs, the key file should help greatly.   
Here is an example MEGA2.KEYS file.

```
---------------------
Fri Oct 11 13:15:05 2002
Input file names
locus file:    datain.ex
pedigree file: pedin.ex1
map file:      map.ex
omit file:     omit.ex
Untyped pedigree option: Include all pedigrees whether typed or not
---------------------
 INPUT                                                OUTPUT
 Pedigree    Person      Ped:       Per:    Loop id   Pedigree    Person
         1          1        1          1                  1          1
         1          2        1          2                  1          2
         1          3        1          3                  1          3
         1          4        1          4                  1          4
         1          5        1          5                  1          5
         1          6        1          6                  1          6
         1          7        1          7                  1          7
         1          8        1          8                  1          8
         1          9        1          9                  1          9
         1         10        1         10                  1         10
         1         11        1         11                  1         11
         2          1        2          1                  2          1
         2          2        2          2                  2          2
         2          3        2          3                  2          3
         2          4        2          4                  2          4
         2          5        2          5                  2          5
         2          6        2          6                  2          6
         2          7        2          7                  2          7
         2          8        2          8     2            2          8
         2         11        2          8     2            2          8
         2          9        2          9                  2          9
         2         10        2         10                  2         10
```

## 21.6 MEGA2.SIM

This file is created if the user selects random-genotyping error generation in the input menu. Details on this file can be found in the Mistyping section .

## 21.7 MEGA2.RECODE

This file contains a list of recoded marker loci with the original allele names, recoded identifiers, allele frequencies, the type of individuals counted for the purpose of estimating allele frequencies, and total number of alleles counted for each allele and each marker. Given below is an excerpt for a recode summary file.

```
----------------------------------------------
        Mega2 version 3.0 R5
Run date:                  2006-1-20-13-53
This file created on       Fri Jan 20 13:53:20 2006
Input file names
  Locus file:              names.01
  Pedigree file:           pedin.01
  Map file:                map.01
  Untyped pedigree option: Include all pedigrees whether typed or not
Mendelianly-inconsistent genotypes included in output.
Half-typed individuals’ genotypes included in output.
Allele frequency computed from:
     Genotyped founders or a randomly chosen individual
     excluding half-typed individuals.
---------------------
Recoded loci:
---------------------------------------------
Frequencies and counts:
   1=Founders       2=1+Random       3=2+Unique     4=Everyone
Marker 2: marker2 has 6 alleles; frequencies computed using 11 individuals:
6 Founders and 5 randomly chosen individuals
Allele  Code      Freq1     Count1  Freq2     Count2  Freq3     Count3  Freq4     Count4
     1 10         0.25000        3  0.31818        7  0.31818        7  0.35714       10
     2 11         0.25000        3  0.13636        3  0.13636        3  0.10714        3
     3 12         0.25000        3  0.13636        3  0.13636        3  0.10714        3
     4 13         0.25000        3  0.31818        7  0.31818        7  0.35714       10
     5 14         0.00000        0  0.04545        1  0.04545        1  0.03571        1
     6 17         0.00000        0  0.04545        1  0.04545        1  0.03571        1
TOTAL                           12                22                22                28
```

Please note that Mega2 now computes and logs allele-frequencies for all 4 subsets of individuals, but uses only the one specified by the user in the re-formatted output data files. \hrule

At the start of the execution, existing error, log, key, and recode summary files are moved into MEGA2.ERR.old, MEGA2.LOG.old, MEGA2.KEYS.old and MEGA2.RECODE.old respectively.

The MEGA2.LOG and MEGA2.KEYS files are created each time Mega2 is executed.

The batch file MEGA2.BATCH is created only if Mega2 is run in the interactive mode. In this case the existing batch file is backed up into MEGA2.BATCH.old.

MEGA2.ERR is created only if there are warnings and messages for the current run.

MEGA2.SIM is created only in the error simulation mode. The existing MEGA2.SIM file is renamed into MEGA2.SIM.old.

MEGA2.RECODE is created only if a names file is used instead of a linkage format locus data file.

## 21.8 MEGA2run.html and related files

This file along with three other html files (MEGA2links.html, MEGA2outputfiles.html, and MEGA2file.html) contains links to the relevant summary files, input and output files for easy reference. Please note this uses frames. In addition, the input and output files are all text files, but may not be recognized as such by the browser, unless its settings are modified. The run summary files point to links which are HTML-versions of the text format summaries, and should display correctly.

If you open the MEGA2run.html in a web-browser, it will provide you with a nicely organized view of the results of the most recent run of Mega2, with links to log files, input files, and output files.

# 22 Mega2 command-line arguments

**Usage:** *mega2* [options] [batch-file-name]

## 22.1 Species options

Mega2 has modest support for different species. Its model is that all chromosomes up to some number are autosomes. The last autosome is then followed by sex chromosome, “X” and “Y”, which have the next two numeric values. The pseudo XY chromosome number may be specified as well as the mitochondrial “chromosome” (“MT”) number. For all chromosomes after the “last autosome”, letter designations should be used in Mega2 files for clarity. Note: Mega2 reserves the letter designation “U” for an unknown chromosome.

```
|  |  |  |  |  |
| --- | --- | --- | --- | --- |
| flag | species | last autosome | pseudo XY | mitochondrial |
| -\/-horse | horse | 31 | - | - |
| -\/-sheep | sheep | 26 | - | - |
| -\/-dog | dog | 38 | 41 | - |
| -\/-mouse | mouse | 19 | - | - |
| -\/-cow | cow | 29 | - | - |
| -\/-human | human | 22 | 25 | 26 |
```

In addition, several flags allow the non-autosomal chromosomes to be specified:

```
|  |  |  |
| --- | --- | --- |
| flag | value | interpretation |
| -\/-autosome | chromosome | last autosomal chromosome number. The next two values are for “X” and “Y”, respectively |
| -\/-pseudo | chromosome | chromosome number for pseudo XY region |
| -\/-mito | chromosome | chromosome number for mitochondrial DNA. |
```

## 22.2 **Input format options**

- *-\/*-mega2  
    
   Mega2 format with header
- *-\/*-linkage  
    
   Linkage format
- *-\/*-extend\_linkage  
    
   Linkage with Mega2 names file
- *-\/*-bed  
    
   PLINK binary PED format (bed)
- *-\/*-ped  
    
   PLINK PED format (ped)
- *-\/*-bcf  
    
   BCF format (vcf)-
- *-\/*-vcf.gz  
    
   VCF compressed format (vcf.gz)
- *-\/*-vcf  
    
   VCF format (vcf)

## 22.3 **Missing Value options**

Several flags allow an override value to be supplied to specify the missing value code read as input or generated into output for the analysis program. Mega2 has rules that constrain the values; using these flags, allows you to break the built-in rules and enter any values you like.

```
|  |  |  |
| --- | --- | --- |
| flag | value | interpretation |
| -\/-quant_in | decimal number | override for batch item Value_Missing_Quant_On_Input and default for interactive menu for same item 23.4↓ |
| -\/-quant_out | decimal number | override for batch item Value_Missing_Quant_On_Output and default for interactive menu for same item 23.4↓ |
| -\/-affect_in | integer number | override for batch item Value_Missing_Affect_On_Input and default for interactive menu for same item 23.4↓ |
| -\/-affect_out | integer number | override for batch item Value_Missing_Affect_On_Output and default for interactive menu for same item 23.4↓ |
```

## 22.4 **General options**

- *-\/-force\_numeric\_alleles*    
    
  As of Mega2 version 4.7.1, if non-numeric alleles are found in the input, they will be passed through to the output and not recoded as numeric alleles. This is done for all input formats that allow letter alleles (i.e., all input formats except LINKAGE) and for the analysis options that support non-numeric alleles. To force the recoding of alleles to numeric alleles to take place, set this flag *-\/-force\_numeric\_alleles.*
- *-x, -\/-nosave*   
    
  Do not create a new run-folder. By default, Mega2 stores run-related summaries in a separate folder tagged with the date and time of the run (for details, see description of summary file directory ). The -x or *-\/-*nosave option turns off this feature, and saves all run-related files with their default names in the folder from which Mega2 is run.
- *-w, -\/-noweb*   
    
  Do not check for latest on-line version. From version 4.0 revision 1, we implemented an automatic check inside Mega2, which queries our distribution site to figure out whether the version being run is older than the released version. This requires that the computer is able to connect to the internet. This check may be turned off using the *-\/-*noweb option.
- *-h, -\/-help*   
    
  Prints the list of available command line options with short descriptions.
- *-v, -\/-version*   
    
  Prints the mega2 version number.

## 22.5 Obsolete options

- *-nosave:*   
    
  Note that the -nosave option is not valid any more. Mega2 will terminate with a message, if it matches any of its arguments to “-nosave”.

## 22.6 *Other arguments*

The only other option accepted by Mega2 besides the switches listed above, is the batch file name. It checks to see that there is at most one such file name. If you mistakenly supplied one of the other valid options, Mega2 proceeds as normal.

# 23 Running Mega2 in Batch mode

## 23.1 Overview

Mega2 can be run in a batch-mode by invoking it with a single argument which is a batch file name:

> mega2 MEGA2.BATCH

\endverbatim

This runs Mega2 in a non-interactive mode through the following steps:

- Input Menu
- Analysis Menu
- Locus Reordering and Selection menu
- Trait Selection Menu
- Genotyping error simulation setup
- Output file names menu
- Input of default parameters

In the previous version, Mega2 started running in the interactive mode after the genotyping error simulation setup, and prompted the user to input analysis-specific parameters, such as output file names etc. New batch file items have been implemented which makes user-interaction unnecessary for the bulk of the execution. Subsequent versions will be made fully automatic except for input data errors that cannot be ignored.

Each time you run Mega2 in interactive mode, a new batch file is created. This batch file is named MEGA2.BATCH, and the existing MEGA2.BATCH is moved into MEGA2.BATCH.old.

## 23.2 Using the *-\/-*nosave option

The purpose of having a batch-execution mode is to enable Mega2 to be embedded within scripts, so as to be run automatically. A typical application of this feature would be running thousands of replicates of simulated data. In this case, mega2 should be run with the *-\/-*nosave (short option -x) option, so that summary files for each run are not saved in a separate directory as below:

```
         mega2 --nosave batch_file
         mega2 -x batch_file
```

The online check for the latest release is automatically disabled in the batch mode.

## 23.3 Batch file format

The batch file is a text file which has a specific format. Each line is either a comment or a definition. Blank lines are ignored. Comment lines begin with a **#** in the first column. A definition line has two parts, a name and a value. Each definition line is of the form:   
  
*Name=Value*

Here is an example batch file

```
Input_Pedigree_File=pedin.06
Input_Locus_File=datain.06
Input_Map_File=map.06
Input_Untyped_Ped_Option=2
Input_Do_Error_Sim=0
Analysis_Option=Solar
Chromosome_Single=6
Traits_Combine=8 9 10 11 12
Value_Missing_Quant=-9.000000
Output_Path=mega2_results
```

## 23.4 Major classes of batch file options

The batch file options are currently classified into 7 different classes which mirror the different menus of Mega2:

- Input menu items
  1. Input\_Format\_Type
  2. Input\_Pedigree\_File
  3. Input\_Locus\_File
  4. Input\_Map\_File
  5. Input\_Omit\_File
  6. Input\_Untyped\_Ped\_Option
  7. Input\_Do\_Error\_Sim
  8. Output\_Path
  9. Input\_Frequency\_File
  10. Input\_Penetrance\_File
  11. Input\_Binary\_File
  12. Input\_Phenotype\_File
  13. Input\_Aux\_File
  14. PLINK
  15. VCF\_Args
  16. VCF\_Marker\_Alternative\_INFO\_Key
- Analysis menu items
  1. Analysis\_Option
  2. Analysis\_Sub\_option
- Chromosome and marker selection items
  1. Chromosome\_Single
  2. Chromosomes\_Multiple\_Num
  3. Chromosomes\_Multiple
  4. Loop\_Over\_Chromosomes
  5. Loci\_Selected\_Num is no longer necessary
  6. Loci\_Selected
  7. Output map number
  8. Value\_Genetic\_Distance\_Index
  9. Value\_Base\_Pair\_Position\_Index
  10. Value\_Genetic\_Distance\_SexTypeMap
- Trait-related items
  1. Trait\_Single
  2. Traits\_Loop\_Over
  3. Traits\_Combine
  4. Trait\_Subdirs
- Special phenotype and genotype indicators, i.e. missing quantitative phenotype, definition of affecteds (used only for multiple liability classes), and unknown allele and affection status indicators  
  1. Value\_Marker\_Compression
  2. Value\_Missing\_Quant\_On\_Input (formerly Value\_Missing\_Quant)
  3. Value\_Missing\_Quant\_On\_Output
  4. Value\_Missing\_Affect\_On\_Input
  5. Value\_Missing\_Affect\_On\_Output
  6. Value\_Affecteds
  7. Value\_Missing\_Allele\_Aff
- Mistyping simulation related items
  1. Error\_Loci
  2. Error\_Except\_Loci
  3. Error\_Loci\_Num
  4. Error\_Model
  5. Error\_Probabilities
- Default behavior related options
  1. Default\_Outfile\_Names
  2. Default\_Reset\_Invalid
  3. Default\_Other\_Values
  4. Default\_Ignore\_Nonfatal
  5. Default\_Rplot\_Options
- Covariate selection items (for GeneHunter, SOLAR, Merlin)
  1. Covariates\_Num
  2. Covariates\_Selected
- More options
  1. Xlinked\_Analysis\_Mode
  2. Count\_Genotypes
  3. Rplot\_Statistics
  4. Count\_Halftyped
  5. AlleleFreq\_SquaredDev
  6. Count\_HWE\_genotypes
  7. Value\_Missing\_Allele\_Aff
  8. Structure.PopDataPheno

Special considerations

The order in which these options appear in the batch file does not affect Mega2’s behavior. Some options are dependent on the definition of others. **Each class is regarded as complete only if an adequate number of definitions in that class are provided.** For example, in the Input menu items class, one has to have at least the three file names (pedigree, locus and map), the untyped-ped option and the error-simulation setup flag. If one or more of these are missing, Mega2 will stop and require the user to go through the input menu before proceeding through the other sections. These and other requirements are described below, and also in the batch file generated by Mega2.   
  
Since the batch file is required to have a very specific format to run correctly, the user is advised to create several test runs in order to become familiar with different batch files. An existing batch file can then easily be adapted to create a new analysis, or a new set of markers, etc.

## 23.5 Details on batch file items

Input\_Format\_Type

This item specifies the input file format type as follows:

1. Mega2 format with header
2. Linkage format
3. Linkage with Mega2 names file
4. PLINK binary PED format (bed)
5. PLINK PED format (ped)
6. BCF format (bcf)
7. VCF compressed format (vcf.gz)
8. VCF format (vcf)
9. Traditional (4.6.1) input file format

Input\_Pedigree\_File

This item specifies the input pedigree file name. This is a required item. The value should be a string without any white-space characters. It will store the name of the pedigree file in *linkage, Mega2 and PLINK PED* input formats and the name of the “.fam” file for *PLINK Binary PED input*.

Input\_Map\_File

This item specifies the name of the input map file. This is a required item. The value should be a string without white-space characters. It will store the map file in *linkage, Mega2 and PLINK PED* input formats and the “.bim” file for *PLINK Binary PED input*.

Input\_Locus\_File

This item specifies the name of the input locus data file. This is a required item for *linkage and Mega2* input format, but it is not used for either PLINK input format. The value should be a string without white-space characters.

Input\_Binary\_File

This item specifies the name of the PLINK binary PED format file. The value should be a string without white-space characters.

*The first two file types above are always necessary and for PLINK PED format these two are sufficient. For Mega2 or linkage formats, the **Input\_Locus\_File** must also be provided. While for PLINK binary PED files, the **Input\_Binary\_File** must be present. Otherwise Mega2 will stop for user-input of the incomplete items at the Input menu.*

Input\_Omit\_File

This item specifies the input omit data file. This is an optional item. The value should be a string without white-space characters.

Input\_Frequency\_File

This item specifies the input allele frequency file name. This is an optional item for Mega2 and either PLINK format input files are used. The value should be a string without white-space characters.

Input\_Penetrance\_File

This item specifies the input penetrance parameter file name. This is also an optional item, for Mega2 and either PLINK format input files are used. The value should be a string without white-space characters.

Input\_Phenotype\_File

This item specifies the input phenotype file name. This is an optional item available for either PLINK format input files. The value should be a string without white-space characters.

Input\_Aux\_File

This item specifies the input auxiliary file name. This is an optional item available for either VCF format input files. The value should be a string without white-space characters.

PLINK

This parameter is for either PLINK input formats. It has an odd format as white space separated strings. The strings consist of flags and values as illustrated in section 39↑.

VCF\_Args

This parameter is for any VCF file format. It is used to specify a subset of the command line options supported by the ’vcftools’ program. These are command line options are (See VCFtools Options) the: Site Filtering Options, the Individual Filters, and the Genotype Filters.

VCF\_Marker\_Alternative\_INFO\_Key

The marker can be specified as the string associated with the ’ID’ field of the VCF file, or as the value associated with a key in the ’INFO’ field. If the user specifies a key (not null) then only the INFO field will be used, otherwise only the ’ID’ field will be used. One or the other is used, never switching between them.

If from this the string value is a ’.’ (unknown) then the marker will be constructed from a prefix (’chr’) appending it to the CHROM and POS VCF file field values in the following way (chrCHROM\_POS). This is not guaranteed to be unique since the VCF file specification allows multiple records to have the same POS. In the construction of this marker name ’X’, ’Y’, ’XY’, and ’MT’ will be used rather than the associated numbers for the chromosomes. This is done in accordance to the species option (See 22.1↑) that Mega2 is told to use.

Input\_Untyped\_Ped\_Option

This item specifies the handling of marker-untyped pedigrees. This is also a necessary item. The value should be an integer greater than or equal to 1. Values 1-3 select one of the first 3 options of the Untyped pedigree option menu . Values 4 and onwards refer to the minimum number of the typed persons in a pedigree in order for it to be included in the analysis.   
4 corresponds to at least 1 typed person   
5 corresponds to at least 2 typed persons   
and so on, i.e. included pedigrees must have at least *< value >  - 3* typed persons.

Input\_Do\_Error\_Sim

This option is decides whether Mega2 should execute the random genotyping error simulation step. Valid values are *“yes/no”* .

Output\_Path

This option decides which directory to create the output files in. The value is a string which should be a valid path-name (absolute or relative path) or “.” which is the current directory. This is the default value. White space is not allowed within the path-name.

Analysis\_Option

Valid strings identifying an analysis option are listed below. The analysis option string is case insensitive.

1. SimWalk2
2. Mendel
3. Aspex
4. GeneHunter-Plus
5. GeneHunter
6. APM is disabled.
7. APM-Mult is disabled.
8. Nuclear families
9. Slink
10. Splink
11. Homogeneity
12. Simulate
13. Summary
14. S.A.G.E.3.0
15. TDT-max is disabled.
16. SOLAR
17. Vitesse
18. Linkage
19. Hardy-Weinberg
20. Allegro
21. MLBQTL
22. S.A.G.E.4.0
23. Premakeped
24. Merlin/SimWalk2
25. Prest
26. PAP
27. Merlin
28. Loki
29. Mendel7+
30. SUP
31. PLINK
32. CRANEFOOT
33. Mega2
34. IQLS/Idcoefs
35. FBAT
36. PANGAEA MORGAN
37. Beagle
38. Eigenstrat
39. Structure
40. PSEQ

Analysis\_Sub\_option

This option allows the user to choose between a file output format or sub-program for a particular analysis option. For example, for SimWalk2 analysis the user must select from among 5 sub-options, Haplotyping, Parametric linkage, Nonparametric linkage, IBD estimation, and Mistyping detection. The analysis options are listed below along with their valid Analysis\_Sub\_options.

> |  |  |
> | --- | --- |
> | **Analysis** | **Analysis\_Sub\_option** |
> | SimWalk2 | Haplotype (1) |
> |  | Parametric linkage (2) |
> |  | Nonparametric linkage (3) |
> |  | IBD estimation (4) |
> |  | Mistyping detection (5) |
> | Aspex | Sib-ibd (1) |
> |  | Sib-tdt(2) |
> |  | Sib-phase (3) |
> |  | Sib-map (4) |
> | Summary | Segregation count (1) |
> |  | Allele frequency (2) |
> |  | Liability (3) |
> |  | Genotyping success (4) |
> |  | Quantitative phenotypes (5) |
> | Vitesse | LINKMAP (1) |
> |  | MLINK (2) |
> | Test for Hardy-Weinberg | Gen (1) |
> |  | HWE (2) |
> |  | Chi-sq (3) |
> |  | Exact (4) |
> |  | Mendel (5) |
> | PLINK | lgen (1) |
> |  | SNP major binary (2) |
> |  | Individual major binary (3) |
> |  | ped (4) |
> | Beagle | Unphased\_Unrelated (1) |
> |  | Unphased\_Trio (2) |
> |  | Unphased\_Pair (2) |
> | Eigenstrat | PACKEDPED |
> |  | PED |
> | PANGAEA MORGAN | pedcheck(1) |
> |  | kinship(2) |
> |  | translink(3) |
> |  | lm\_linkage(4) |
> |  | lm\_bayes(5) |
> |  | lm\_ibdtests(6) |
> |  | lm\_ibdtests\_lr(7) |

Chromosome\_Single

This option refers to the locus reordering menu option 1. It refers to the chromosome selected for analysis. If item **12** below is specified as well, then the marker numbers specified by **12** refer to markers on the chromosome specified by this option. The value should be a single integer. The validity of the chromosome is decided after the locus data file and map file are read in, and the chromosome numbers present within the input data are known.  
If there are no chromosomal markers, then this item is ignored, and the user is required to provide loci numbers using the Loci\_Selected keyword.

Chromosomes\_Multiple\_Num

This option is a single integer which decides how many chromosome numbers should be read in option 11. Valid values are positive integers. This option is necessary for option Chromosomes\_Multiple89↓ to work, otherwise Mega2 will report an error in the batch-file and terminate.

Chromosomes\_Multiple

This option specifies which chromosomes should be selected for output. It should be a list of integers, each being a chromosome number present in the input data (as decided by the map and locus data files). In addition to integers the values ’X’, ’Y’, ’XY’, and ’MT’ are also valid. This option also requires that the batch file option Chromosomes\_Multiple\_Num88↑ be specified. There must be at least as many chromosome numbers in this list as specified in option Chromosomes\_Multiple\_Num.

For the three options 8-10, if option 8 is specified, the others are ignored.

Loop\_Over\_Chromosomes

This option specifies whether data from multiple chromosomes are written to the same file or not. If true (y), each chromosome gets its own file. If false (n) only one file is produced which contains data for all chromosomes.

Loci\_Selected

Important: From version 3.0 onwards, the keyword Markers\_Selected\_Num has been decommissioned, and the keyword Markers\_Selected is now Loci\_Selected. If the old keywords appear within the batch file, they will be ignored.   
The Loci\_Selected option specifies the marker numbers to be selected for analysis. As mentioned in option 9, these markers refer to the ones present on the chromosome number specified in option 9. Whether these markers are valid can only be decided after marker data has been read in.  
The list of markers have to be terminated with “e”.

Value\_Genetic\_Distance\_Index

This item specifies which genetic map to use for analysis. The index is zero based. Maps are numbered in the order that they are first discovered in the map file, reading from left to right.

As an example, if the input map file is that discussed in 9.1.3↑, then map ‘M’ would be represented by an index value of 0, map ‘M2’ by an index value of 1, map ‘M3’ by an index value of 2, and map ‘M4’ by an index value of 3. Since only map ‘M’ and ‘M4’ are genetic maps, valid values for the ’Value\_Genetic\_Distance\_Index’ are 0 and 3.

```
Chromosome M.h.a    Name   M.h.m   M2.p    M.h.f  M3.p   M4.k.a   M4.k.m
1          0.0     rs101   0.0     543     0.0    304    0.0       0.0 
1          5.0     rs102   2.0     678     7.0    821    5.0       2.0 
X          0.0     rs231   0.0     743     0.0    912    0.0       0.0 
X          4.0     rs232   1.0     862     6.0    954    4.0       0.0
```

Value\_Genetic\_Distance\_SexTypeMap

This item specifies the map type to be used from the selected genetic map. For a sex-averaged map the value is 0, for a sex\_specific map the value is 1, for a female map the value is 2.

Value\_Base\_Pair\_Position\_Index

This item specifies the physical map to use for analysis. The index is zero based. If no physical map is to be used for analysis, an index value of -2 is used. Maps are numbered in the order that they are first discovered in the map file, reading left to right. As an example, if the input map file is that discussed in 9.1.3↑, then map ‘M’ would be represented by an index value of 0, map ‘M2’ by an index value of 1, map ‘M3’ by an index value of 2, and map ‘M4’ by an index value of 3. Since only map ‘M2’ and ‘M3’ are physical maps valid values for ’Value\_Base\_Pair\_Position\_Index’ would include 1, 2, and also -2 (none).

```
Chromosome M.h.a    Name   M.h.m   M2.p    M.h.f  M3.p   M4.k.a   M4.k.m
1          0.0     rs101   0.0     543     0.0    304    0.0       0.0 
1          5.0     rs102   2.0     678     7.0    821    5.0       2.0 
X          0.0     rs231   0.0     743     0.0    912    0.0       0.0 
X          4.0     rs232   1.0     862     6.0    954    4.0       0.0
```

Trait\_Single

This option corresponds to the menu option 1 of the Trait reordering menu. It should be a positive integer, referring to the list of trait loci present in the input data. Trait loci are numbered 1 - N (the total number of trait loci) in order of their appearance in the locus data file.  
For example, if the locus data file lists loci 10-12 as “trait1” through “trait3”, specifying a “1” in this option selects “trait1” and so on.

Traits\_Loop\_Over

This option corresponds to the menu item 2 of the trait reordering menu where traits are selected to be analyzed one at a time. Trait numbers should correspond to the order in which they are listed in the locus file, starting from 1 (as in option 14).

Traits\_Combine

This option corresponds to the menu item 3 of the trait reordering menu where trait loci are selected to be combined in the same output. The actual trait numbers should correspond to the order in which traits appear in the locus file, and these numbers can be permuted for the purpose of reordering them in the output. There should also be an item numbered *N + 1* where N is the number of traits. This item refers to the [MARKERS] item displayed in the trait selection menu-option 3.   
For options 13, 15 and 16, option 13 takes precedence over the other items, and option 15 takes precedence over option 16 i.e. if both “Trait\_Single” and “Traits\_Loop\_Over” are defined then the latter is ignored.

Trait\_Subdirs

These are trait-specific directories in which output files are created if the Traits\_Loop\_Over option is defined. The value is a list of strings separated by white-space in a single line. The number of directory names read in depend on option 14.

Value\_Marker\_Compression

This is an integer value: 1, 2, or 3. The default value is 1. In this case, each pair of alleles is represented internally with two bits, the same as the PLINK compressed binary ’bed’ representation. This technique does not provide enough space to represent microsatellite markers or half-typed genotypes. For these data sets, the compression value of 2 should be adequate. It provides for up to 256 alleles. The compression value of 3 is left for historical comparisons - it should never be necessary.

Value\_Missing\_Quant\_On\_Input (Value\_Missing\_Quant is deprecated)

If the list of trait loci selected include one or more quantitative traits or covariates, then this value is interpreted as the missing value code in the input data. The value should be a real number or the string ’NA’. In previous versions of Mega2 this item was Value\_Missing\_Quant, which still can be used but is not guaranteed to be supported in future versions of Mega2.

Value\_Missing\_Quant\_On\_Output

If the list of trait loci that will be output include one or more quantitative traits or covariates, then this value is used as the missing quantitative phenotype value in the output data for those analysis modes where missing values are not predefined (e.g., Merlin uses ’x’ for its output missing value).

Value\_Missing\_Affect\_On\_Input

This value is interpreted as the missing value code for affection status traits in the input data. The value may be string ’NA’. PLINK uses -9 as the default missing value indicator and also allows you to change it via a flag (missing-phenotype) which Mega2 also honors. If it is not specified, the value for Value\_Missing\_Quant\_On\_Input is used.

Value\_Missing\_Affect\_On\_Output

This value is used as the missing phenotype value for affection status in the output data for those analysis modes where missing values are not predefined (e.g., Merlin uses ’x’). If it is not specified, the value for Value\_Missing\_Quant\_On\_Output is used.

Value\_Affecteds

If any of the affection loci selected have more than one liability class, then this value refers to the list of  < status > - < class >  pairs which should be considered affected.   
The value should contain one entry per multiple-liability trait, with white-space separating each entry. Each entry specifies the trait name followed by the list of affection labels, and looks like the following:

ValueAffecteds=TRAIT:2-1,2-3 trt2:2-\*

\endverbatim

The “\*” denotes a wild-card e.g., 2-\* means status 2 and all classes. Similarly “\*-1” would mean all known phenotypes in liability class 1 should be treated as being affected. Please also consult the affection label definition menu.

Error\_Loci

These and the following options are used by the genotyping error simulation module of Mega2. This option defines a set of locus numbers at which random genotyping errors will be introduced. These should be a subset of the marker loci chosen in the locus reordering step. The number of loci read in depends on option 23. The locus numbers are selected from all the loci selected in the reordering step.

Error\_Except\_Loci

If this option is defined instead of option 21, this list of loci is excepted from errors. The number of loci read in also depends on option 23 and locus numbers refer to the full list of loci selected in the locus-reordering step.

Error\_Loci\_Num

The value of this option is a single integer denoting how many markers to read in from options 21 or 22. It should be a positive integer.

Error\_Model

This option specifies the error model to use in error simulation. This should be a single character, *“u” or “U”* , *“m” or “M”* , *“s” or “S”* . They refer to the Uniform error probability model, Marker-specific uniform error probability model, and the SimWalk2 error model respectively.

Error\_Probabilities

This option specifies the prior genotyping error probability or probabilities for error simulation. It should be a single real value, if the model specified in option 24 is “Uniform”, or a list of 5 real values, if the “SimWalk2” error model is specified. Marker-specific error probabilities can only be specified in the map file.

Default\_Outfile\_Names

If set to *yes* , the output file-names menu will be skipped for all options. In addition to file names, some of these menus also contain options for selecting pedigree and person identifiers in the output files, these will be set to default values as well.

Default\_Reset\_Invalid

This option defines how invalid genotypes should be handled without pausing for user-input via the invalid-genotypes menu (which is skipped). If set to *yes* the genotypes will be reset to unknowns, and if set to *no* invalid genotypes will not be reset.

Default\_Other\_Values

If set to *yes* Mega2’s default value settings will be used for miscellaneous parameters within various options (such as random seed for simulate), and Mega2 will skip the input menus meant for those parameters. This has not been completely implemented yet, so Mega2 may still halt and ask for input.

Default\_Ignore\_Nonfatal

If set to *yes* Mega2 will not halt for user-input on whether to continue execution if non-critical inconsistencies are found in the input data such as extra locus names in map file or locus file. Otherwise, it will halt for user-input upon encountering such inconsistencies.

Default\_Rplot\_Options

If set to *yes* , Mega2 will skip the Rplot parameters menu and use its default values instead for options that set up R plots.

Xlinked\_Analysis\_Mode

This value can be 0, 1, or 2 depending on how the data should be analyzed: 0 indicates that all data should be treated as autosomal, 1 indicates that all data should be considered sex-linked, and 2 implies that the data is a mixture of autosomal and x-linked loci. Markers on the SEX-CHROMOSOME, set to 23 by default in Mega2 should be treated as X-linked, whereas loci on all other chromosomes will be considered autosomal.

Covariates\_Selected

The trait loci defined in this keyword are output as covariates for the GeneHunter format option. In future releases, we plan to include more options that make use of this keyword.

Count\_Genotypes

This item is used in Mega2’s Recode facility, and refers to which individuals are counted for the purpose of estimating allele frequencies. The values correspond to the choices in the Individuals selection menu.

1. Genotyped founders only
2. Genotyped founders + a randomly chosen genotyped person from pedigrees without genotyped founders.
3. Genotyped founders + genotyped individuals with unique alleles
4. All genotyped individuals (default)

Rplot\_Statistics

This is a list of numbers that refer to the list of statistics that are to be plotted within SimWalk2-NPL, Merlin-SimWalk2, Merlin and Allegro. Please refer to the individual analysis options for a list of statistics that are supported. The numbers match with the statistics menu displayed in the interactive mode.

Count\_Halftyped

Starting with the R5 revision of Mega2 version 3, the inclusion of half-typed individuals within the recoding process is optional. Setting this to yes means that individuals who are typed only at one allele are considered to be “genotyped”, and counted in the allele frequency computation if they otherwise fall within one of the individual selection categories given in the “Individual Selections” menu.  
Please note that setting this to “yes” does not imply that they will also be included in the output files. This latter choice is made in the “Invalid genotypes” menu after error-checking of the data . The corresponding batch item is Default\_Reset\_Invalid111↑.

AlleleFreq\_SquaredDev

This is a real value which is used to decide whether input allele-frequencies are different from that observed in the data for any marker locus. If the sum of squared deviation for all alleles of a marker exceeds the specified value, the user is notified and prompted whether Mega2 should be terminated. Setting this value to a very large number, or setting Default\_Reset\_Invalid should prevent Mega2 from halting for user-input in the batch mode.

Count\_HWE\_genotypes

This is similar to the “Count\_Genotypes117↑” item, however, only considered within the HWE estimation option of Mega2. Refer to the HWE option details for what the value should be.

Value\_Missing\_Allele\_Aff

The Mega2 map file allows the user to also specify the indicator for unknown allele and affection status values. This is again an optional item, and used only if Mega2 format input files are used.

Structure.PopDataPheno

Structure allows the user to specify a user-defined population for each individual; when this option is used, Structure expects each individual to be assigned an integer code representing their population. In Mega2, this information can be stored in a quantitative trait column in the pedigree file. This batch file item Structure.PopDataPheno specifies the name of the quantitative trait containing the population assignments for each individual. Only the integer portion will be used as the population group IDs, and an error will be generated if any of the values are negative.

**OBSOLETE OPTIONS** [Updated]  
The following batch file keywords are obsolete. If these are present in the batch file, they will be ignored and Mega2 will warn the user of their presence.

- Markers\_Selected, use Loci\_Selected instead
- Loci\_Selected\_Num, Markers\_Selected\_Num, Covariates\_Selected\_Num
- Default\_Ignore\_Xlinked
- Traits\_Num
- Output\_Map\_Number
- Value\_Missing\_Quant, use Value\_Missing\_Quant\_On\_Input instead

# 24 Hints and troubleshooting

## 24.1 Hints on loci reordering

1. If you wish to create a set of LINKAGE format files with all the markers re-arranged into their map order, one chromosome after the other, choose Option ‘18) Convert to Linkage format’ from the Analysis menu and then choose the sub-option 1 ‘Select all markers in order on all chromosomes from Option 4 of the Locus Reordering Menu. This is useful, for example, if you want to run the Analyze package and you want all the markers in chromosomal order.
2. If you have a single affection status locus in your input files, and then choose sub-option 1 from Option 4 of the Locus Reordering Menu, then Mega2 will create multiple sets of chromosome-specific files for many of the analysis options, including ASPEX, GeneHunter-Plus, and GeneHunter. A ‘global’ C-shell script will be created that will analyze one chromosome after another, all at once. This greatly eases analyses of genome-wide data sets.
3. Option 4 lets the user select some or all chromosomes. Typically the output files are chromosome-specific, each set of output files containing genotype data from all marker loci on one chromosome along with trait loci.   
   Many linkage analysis programs do not care about the ordering of trait loci with respect to the marker. So, by default Mega2 would place the trait loci at the beginning of the marker order. If this order is important to you, then there are two ways to specify an alternate ordering:
   1. Reorder menu option 2: This works for markers on a single chromosome.
   2. Trait selection menu’s Combine option   
      If menu item number 2 is not set to the “Combine” mode, this should be the first step BEFORE selecting the trait loci. Enter 2 at the prompt to switch modes, then press 1 to obtain a list of traits along with a [MARKER] item, which is a place-holder for marker loci.Specify the desired order using the item numbers. The resulting output file will have the markers inserted in the right place with respect to the trait loci. For chromosome-specific output files, markers for each chromosome will be substituted in turn, otherwise, if chromosomes are being combined into one set of files, then all chromosomal markers selected appear in this position.

## 24.2 Problems commonly encountered with input files:

Fatal errors cause Mega2 to terminate without creating output files. We try to identify as many problems with the data files as we can before termination of a Mega2 run, for the user’s benefit. However, several iterations may be necessary with wrong or corrupted data.

## 24.3 Pedigree file reading problem

Reading in a pre-makeped file as a post-makeped file has been reported frequently in the past. Mega2 judges whether a file is in pre-makeped format or post-makeped format by looking at the 5th column, which should be the *sex* column in a pre-makeped file format. If this column contains anything other than 1s and 2s, the pedigree file is read in as a post-makeped file, so either the sex column has to be filled in, or the pedigrees need to be converted to post-makeped outside of Mega2.

## 24.4 Mega2 hangs while reading in input files

A common cause is that one of the files do not have a newline at the end of the last line. An easy way to check this on a Unix system is to count the lines using the command **“wc -l”** . If the last line does not contain a newline, then this will print a number which is one short of the expected number of lines.

## 24.5 DOS format file related errors

The most common problem users have faced so far has had to do with DOS format map files. DOS text files contain the “^M” carriage-return character in addition to the “^J” line-feed character used by the Unix C library routines. This caused the map file to be read in incorrectly by earlier versions up to version 2.1 beta R3. This resulted in warning messages such as:

```
WARNING: Locus GENE1 is not in map file.
```

although GENE1 is actually present in the marker file. In extreme cases, Mega2 cannot find a single marker defined in the Map file and terminates abnormally.

This problem is only encountered if DOS-files are being run though a Unix copy of Mega2. Therefore, each input file is first checked for “^M” characters, if these are present, Mega2 exits with an error message.

Dos formatted files should be converted to Unix formatted files by removing the “^M” characters. This can be done manually, or through freely available programs. If you have Perl installed, here is a one-line Perl utility:

```
perl -pi -e ’s/\r\n/\n/g’ pedin.01
```

Solaris usually comes with a “dos2unix” utility installed. Linux RPMs are available for a variety of platforms (try to locate one suitable for you.)

Files created by Mac-specific applications may contain the “^M” character, but not the “^J”. The “dos2unix” and “mac2unix” utilities may be used to convert these files, alternatively, you can use Perl:

```
perl -pi -e ’s/\r/\n/g’ pedin.01
```

For more information, see http://en.wikipedia.org/wiki/Newline#Conversion\_utilities

### 24.5.1 Error messages

- “Pedigree file appears to be a DOS format file.” This means that Mega2 encountered the “^M^J” sequence.
  - “Pedigree file appears to be a Mac-format file.”   
    This would appear if a “^M” character was encountered without the “^J”.

## 24.6 Non-DOS related problems

Macintosh users - Note that on your system file-names may be insensitive to letter case. So, if you create a file map.dat, this is equivalent to having a file MAP.DAT (or any combination of uppercase and lower case letters therein). Thus, if you try to modify a file named **MAP.DAT**, you will notice that you are changing **map.dat** instead.   
  
This may cause problems if you use a Mega2 input map file map.dat, then create files for SimWalk2 inside the same folder, and run the SimWalk2 shell script. The SimWalk2 map file name is hard-coded as MAP.DAT, therefore the shell script creates a copy of the Mega2-created SimWalk2 map file with this name prior to each SimWalk2 run.   
  
Mega2 warns the user if they have used an input map file with the name **map.dat**, and selected the SimWalk2 option.

# 25 Detailed information on analysis options

## 25.1  Create SimWalk2 format files

Choosing this option will lead you to this sub-menu of SimWalk2 specific options:

```
SimWalk2 program options:
1) Haplotype analysis
2) Parametric linkage analysis
3) Non-parametric linkage analysis
4) IBD estimation
5) Mistyping analysis
```

Suppose the chromosome number is 5. Then Mega2 will, by default, generate the following files (the [\*] represents different names for each sub-option).

```
   Locus file:           sw2_locus.05   
   Pedigree file:        sw2_pedigree.05
   Map file:             sw2_map.05     
   Penetrance file:      sw2_pen.05     
   Simwalk2 batch file:  sw2_batch.05   
   C-Shell script:       sw2_haplo.05.sh
```

It multiple traits and/or multiple chromosomes are selected for analysis, the a combined shell script is created which executes the chromosome-specific shell scripts in the individual trait directories. This shell script is called **[\*].all.sh**

If user selected to set up R-graphics for the non-parametric analysis option, then this script will also execute the Rsimwalk2.\*.sh script that sets up commands for creating postscript files of LOD score plots. This ensures that all requires analyses are run before the graphics are generated.

Given below is an example of this combined shell script for two traits on a single chromosome:

```
#----------------------------------------------
#   Mega2 version 4.2
#   Run date:                2009-4-15-09-49
#   This script created on   Wed Apr 15 09:49:08 2009
#   Input file names:
#       Pedigree file:              pedin.01
#          Locus file:              names.01
#            Map file:              map.01
#    Untyped pedigree option  Include all pedigrees whether typed or not
#----------------------------------------------
echo SimWalk2 analysis for chromosome 1 ,trait 1 ...
echo This may take a while.
cd Trait-1
./sw2_npl.05.sh
cd ..
echo SimWalk2 analysis for chromosome 1 ,trait 2 ...
echo This may take a while.
cd Trait-2
./sw2_npl.05.sh
cd ..
echo Executing R shell script Rsimwalk2.all.sh
./Rsimwalk2.all.sh
```

Note that, when generating the Mendel format pedigree file, Mega2 automatically reconnects the loops (if the input file is in post-Makeped LINKAGE format with broken loops), which is very important for running the haplotyping programs. Unfortunately, reconnection of loops will result in a renumbering of the person IDs, which can be confusing (One can run Makeped and tell it that there are no loops, this results in a post-Makeped LINKAGE format file with loops intact and thus Mega2 will not need to reconnect any loops).

The batch file for SIMWALK2 will contain the appropriate recombination fractions, as inferred from the information provided in the map file. We use the Haldane map function to convert cM to recombination fractions (that reside in the batch file).

Simwalk2 does not currently handle QTLs. Moreover, at most one trait locus is allowed at the beginning of the locus order. The trait locus can be omitted for Haplotyping, IBD estimation and Mistyping analysis, a trait is required for the non-parametric and parametric linkage analysis options. Note that the shell script will overwrite the files LOCUS.DAT, PEDIGREE.DAT, PEN.DAT, MAP.DAT BATCH2.DAT, and ERROR-\*.TXT. This is more of an issue with some versions of the Macintosh OS, where files names are case-insensitive. So, if you have a Mega2 format input file named **map.dat** , this will be overwritten, unless you select a different output folder using the input menu option 8 .   
  
The haplotyping shell script is called **haplo.05.sh**   
The name of the Location scores C-Shell script file is **loc.05.sh** .   
The Non-parametric linkage C-Shell script is called **npl.05.sh** .   
The IBD estimation C-Shell script is called **ibd.05.sh.**   
The Mistyping analysis C-Shell script is called **mis.05.sh.**

## 25.2  Convert to MENDEL format

Suppose the chromosome number is 5. Then Mega2 will, by default, generate the following files:

```
                 Mendel pedigree file:   pedm.05
                 Mendel locus file:      locus.05
                 Mendel penetrance file: pen.05
```

Note that the penetrance file will only be created if there are one or more affection status loci in the set of selected loci.  
Detailed explanation of the penetrance file:  
Our penetrance file is designed to permit the user to duplicate precisely any analysis done using the LINKAGE package (http://linkage.rockefeller.edu/soft/linkage/) which relies on liability class coding of the penetrance vectors at the disease locus. In this approach, the disease locus is assumed to have two alleles, and then a person’s penetrance is defined in terms of a vector whose entries are the penetrances for the 1/1, 1/2, and 2/2 disease genotypes, in that order. For example, an autosomal recessive trait with full penetrance would have the penetrance vector [0, 0, 1] for the affecteds and the vector [1, 1, 0] for the normals (if the disease allele is the second allele).   
To use the penetrance file, you need a set of three matched input files:  
1) locus.dat  < - The usual MENDEL locus file.  
2) pedm.dat  < - The usual MENDEL pedigree file with one quantitative variable representing the phenotype at the trait locus.  
3) pen.dat  < - A penetrance table (created by Mega2).   
  
NOTE: The alleles must be listed in the ‘natural’ order in the ‘locus.dat’ file, so allele ‘1’ is listed first, and allele ‘2’ is listed second. Mega2 will do this automatically.  
For each person in the pedigree file, SIMWALK2 (or the USERM15 MENDEL module) looks up the appropriate penetrance vector based on the person’s value at the quantitative variable (which must be in one-to-one correspondence with the disease phenotypes). Mega2 does this in the following manner: It takes the affection status code (‘1’ = normal, ‘2’ = affected) and multiplies it by 100 and adds to the liability class. So someone who was coded in the original linkage file as ‘2 4’ (i.e., affected in class 4) is assigned the quantitative variable ‘204’ and so we can look up the correct penetrance for them in the ‘pen.dat’ file (see example below).  
Example:   
If we input this LINKAGE-style datafile into Mega2, then it will generate the ‘pen.dat’ file seen below:   
LINKAGE-style datafile.dat:

```
2 0 0 5  << NO. OF LOCI, RISK LOCUS, SEXLINKED (IF 1) PROGRAM 
0 0.0 0.0 0  << MUT LOCUS, MUT RATE, HAPLOTYPE FREQUENCIES (IF 1)
1	2
1	2  # trait 
0.999800 0.000200   << GENE FREQUENCIES
4 << NO. OF LIABILITY CLASSES
0.0000 0.0500 0.0500
0.0000 0.2000 0.2000
0.0000 0.6000 0.6000
0.0000 0.8000 0.8000 << PENETRANCES
3	3  # M1
0.200000 0.166670 0.633330   << GENE FREQUENCIES
0 0  << SEX DIFFERENCE, INTERFERENCE (IF 1 OR 2)
0.10000 << RECOMBINATION VALUES
1 0.02500 0.50000 << REC VARIED, INCREMENT, FINISHING VALUE
```

Resulting ‘pen.dat’ file:

```
1	<= Number of affection status loci trait
4     <= Locus name, Number of liability classes
101 1.00000 0.95000 0.95000 <= Penetrances 1  1   
102 1.00000 0.80000 0.80000 <= Penetrances 1  2   
103 1.00000 0.40000 0.40000 <= Penetrances 1  3   
104 1.00000 0.20000 0.20000 <= Penetrances 1  4   
201 0.00000 0.05000 0.05000 <= Penetrances 2  1   
202 0.00000 0.20000 0.20000 <= Penetrances 2  2   
203 0.00000 0.60000 0.60000 <= Penetrances 2  3   
204 0.00000 0.80000 0.80000 <= Penetrances 2  4
```

Restrictions:  
USERM15 expects only one affection status locus which should be first in the locus order.  
Mega2 supports no more than 99 liability classes.  
If your input pedigree file has been created by Makeped, then, usually, Makeped will have re-numbered your pedigrees, making it very difficult to interpret the resulting data without going through the hassle of figuring out the correspondence between the new person IDs and the original person IDs. Thus, since Mendel supports text IDs, Mega2 will now use original IDs if they exist by default. These original IDs are assumed to appear at the end of each record in the pedigree file in the format:   
  
... Ped: 1 Per: 100101   
  
For Mendel, this option may be toggled on or off within the ’MENDEL file name menu’:

```
==========================================================
  MENDEL file name menu
==========================================================
0) Done with this menu - please proceed
 1) Locus file name:           locus.06         [overwrite]
 2) Pedigree filename:         pedm.06          [overwrite]
 3) Penetrance file name:      pen.06           [overwrite]
 4) Batch file name:           batch.06         [overwrite]
 5) M13 batch file name:       m13bat.06        [overwrite]
 6) Use original ids if given:  yes
Select options 0-5 to enter new file names, 6 to toggle >
```

## 25.3  Convert to ASPEX format

The Aspex option has 4 sub-options

```
1) sib_ibd
2) sib_tdt
3) sib_phase
4) sib_map
```

This option produces one set of ASPEX input files for each chromosome selected. The files are (e.g. for chromosome 6 and the sib\_ibd option):

```
        Aspex locus file:           asp_in.06 
        Aspex pedigree file:        asp_dat.06 
        C-shell script file:        sib_ibd.06.sh
```

User has the option of creating a combined c-shell script for multiple chromosomes. Mega2 allows the user to define a list of parameters to be specified in the Aspex locus file, which will control subsequent Aspex runs. The parameters and their default settings are as follows:

```
        1) discard_partial:        [true]
        2) linear_model:           [true]
        3) most_likely:            [true]
        4) fixed_step:             [false]
        5) no_Dv:                  [true]
        6) fixed_freq:             [false]
        7) truncate_sharing:       [true]
        8) count_once:             [false]
        9) first_pair:             [false]
        10) count_unaffected:      [false]
        11) count_discordant:      [false]
        12) sex_split:             [false]
        13) show_pairs:            [false]
        14) mapping:               [haldane]
        15) max_step:              [ 1.0000 cM]
        16) error_freq             [0.0000]
        17) exclusion_level        [0.0000]
        18) Distance to left end
            of chromosome          [10.0000 cM]
        19) Distance to right end
            of chromosome          [10.0000 cM]
```

The user can also elect to convert the families to Nuclear families, and define risk-ratios.   
If your input pedigree file has been created by Makeped, then, usually, Makeped will have re-numbered your pedigrees, making it very difficult to interpret the resulting data without going through the hassle of figuring out the correspondence between the new person IDs and the original person IDs. Thus, since ASPEX supports text IDs, Mega2 will now use original IDs if they exist and if the user chooses not to use the “conversion to nuclear families” option. These original IDs are assumed to appear at the end of each record in the pedigree file in the format:   
  
... Ped: 1 Per: 100101   
  
This option may be toggled on or off within the ’ASPEX file name menu’:

```
==========================================================
ASPEX file name menu:
==========================================================
0) Done with this menu - please proceed.
 1) Locus file name:           asp_in.06        [overwrite]
 2) Pedigree datafile name:    asp_dat.06       [overwrite]
 3) C-shell script file name:  sib_ibd.06.sh    [overwrite]
 4) Output original ids if given:   yes
Select options 0-3 to enter new file names, 4 to toggle >
```

## 25.4  Convert to GeneHunter-Plus format

For each chromosome Mega2 creates the following set of GeneHunter-Plus format files (for e.g. chromosome 6):

```
      GeneHunter pedigree file:       gh_ped.06 
      GeneHunter locus file:          gh_dat.06
      GeneHunter command file:        gh_in.06  
      C-shell script file:            gh_script.06.sh
```

The Locus file is identical to the linkage format locus data file. The pedigree file is in the Pre-makeped linkage format. The command file contains GeneHunter-Plus commands to load in the data, and perform non-parametric linkage analysis on the data.  
Example command file:

```
photo ghp.06.out 
load ghpdat.06
use 
score 
scan ghpped.06 
ps on 
total het 
nplall.06.ps 
lod.06.ps 
info.06.ps 
quit
```

In order to run GeneHunter-Plus on these data files, one needs only to execute the C-shell script.

## 25.5  Convert to GeneHunter format

Files created for GeneHunter:   
A set of files similar to those for GeneHunter-Plus is created for this option as well. As before, the pedigree file is in pre-Makeped format, and the locus file is in the standard linkage locus file format.

```
      GeneHunter pedigree file:       gh_ped.06 
      GeneHunter locus file:          gh_dat.06
      GeneHunter command file:        gh_in.06  
      C-shell script file:            gh_script.06.sh
```

Selection of trait Loci:   
GeneHunter supports quantitative as well as affection status loci. One or more of these quantitative loci can be labeled as covariates. However, there are restrictions on how the loci and genotypes/phenotypes may be ordered. The required order is one affection status locus, followed by the marker loci, followed by quantitative traits, then finally the covariates. This is also reflected in the pedigree record.   
A typical locus file would be as follows:

```
10 0 0 5
0 0.000 0.000 0
1 2 3 4 5 6 7 8 9 10
1 2 # gaw12
 0.999900 0.000100
1
 0.0100 0.8000 0.8000
3 6 # D06G025
 0.104180 0.045500 0.024650 0.196970 0.283060 0.345640
3 7 # D06G028
 0.080650 0.084430 0.186500 0.150680 0.148510 0.234920 0.114310
3 8 # D06G034
 0.373830 0.084400 0.092330 0.070510 0.016640 0.096660 0.144270 0.121360
3 4 # D06G035
 0.354540 0.330390 0.071380 0.243690
3 14 # D06G041
 0.001670 0.005050 0.050980 0.034930 0.000060 0.101470 0.187700 0.030160 0.062930 0.011720 0.276800 0.012440 0.088740 0.135350
3 8 # D06G043
 0.098180 0.118750 0.207360 0.228480 0.049630 0.045060 0.235700 0.016840
0 2  # Q1
 
 
 
 
 
0 2  # Q2
 
 
 
 
 
4 0 # Q3
0 0
 0.50000 0.04431 0.03816 0.01400 0.05307 0.02095 0.49000 0.49000 0.49000 Haldane
1 0.10000 0.45000
```

Specification of means, standard deviations, etc. are not necessary for the quantitative variables, so these lines are required to be blank in order to match the linkage format. Note also, the definition for trait “Q3”. The “4 0” denotes a co-variate, and this locus record contains only a single line.   
Mega2 allows omission of the affection status locus during locus re-ordering for GeneHunter. In this case, it inserts a “dummy” locus at the proper position. The user is informed of the Insertion of a dummy locus on the screen and inside the log file.

## 25.6  Convert to APM format

This option creates the files needed for analysis via the Affected Pedigree Member method (single marker analyses). A single file is created for all the markers irrespective of whether they are on the same chromosome or not:

kinml.06

\endverbatim

for only chromosome 6 and

kinml.all

\endverbatim

for combined data from multiple chromosomes.

## 25.7  Convert to APM MULT multiple locus format

This option creates the files needed for analysis via the multilocus version of the Affected Pedigree member method. For chromosome 6, 4 marker loci, and for an interval size of 3 markers, the following files are created:

```
    Data file: kin_mult1_3.06
    Data file: kin_mult2_4.06
    Script file apmmult.01.sh
```

The user can select the length of the marker interval via a menu.

## 25.8  Create nuclear families

This option breaks down larger pedigrees into their component nuclear families (parents plus children), and output the resulting locus and pedigree files in LINKAGE format. It automatically renumbers the pedigrees by multiplying the original pedigree id by 100 and incrementing it by the number of nuclear families found so far. Individuals are numbered consecutively starting from 1 inside each family. The files are named:   
1) For each specific chromosome (# 1 in this case):

```
     Locus file nuke_data.01.
     Pedigree file nuke_ped.01.
```

2) For combined output from multiple chromosomes:

```
     Locus file nuke_data.all.
     Pedigree file nuke_ped.all
```

The user has a choice of combined or separate output files per chromosome. Markers can be reordered freely.

## 25.9  Convert to SLINK format

This option creates the files needed for input into the simulation programs SLINK and FASTSLINK. User can choose from a list of options that control the output files, such as including families without affecteds, changing recombination fractions between markers, proportion of unlinked families etc.   
The following files are created for this option (all chromosome-specific):

```
    SLINK-format pedigree file : simped.01
    SLINK-format locus file    : simdata.01
    SLINK-format parameter file: slinkin.01
    SLINK-format C-shell file  : slink.01.sh
```

## 25.10  Convert to SPLINK format

When one chooses to create files in SPLINK format, the following menu will appear:

```
SPLINK file conversion options:
1) Single pedigree file for all markers.
2) One pedigree file for each marker.
```

If you are seeking to use SPLINK to compute maximum LOD scores for each marker, you should choose option 2. This is because if there is more than one marker in an input SPLINK file, then SPLINK will treat these as forming a haplotype. So, in most cases, Option 2 is the one to use.  
  
The ’splink.sh’ script will append the results to the file ’splink.lst’. However, this C-shell script will only work with the latest version of SPLINK (version 1.08 or higher), as it uses the “-S+” command line option (which replaces the “-S” option found in earlier versions).

## 25.11  Set up for homogeneity analyses

This option creates the files needed to compute LOD scores under heterogeneity, using component programs from the ANALYZE package. Output from multiple chromosomes can be combined into a single set of output file. The following files are created:

```
     Pedigree file : lod2ped.01  
     Locus file    : lod2data.01 
     C-shell file  : lod2.01.sh
```

This option can handle only a single trait (affection status or quantitative) per analysis. If multiple traits are selected, then separate output files will be created for each trait.

## 25.12  Convert to SIMULATE format

This option creates the files needed for input into the simulation program SIMULATE. The following files are created (one set per chromosome):

```
    Pedigree file : simped.06
    Locus file    : simdata.06
    Parameter file: problem.06
```

SIMULATE will also allow only a single trait which has to be a binary trait.

## 25.13  Create summary files

Selection Menu: Summary file options 1) Create segregation and relative count summary files. 2) Create allele frequency summary table. 3) Count alleles and genotypes within groups. 4) Create genotyping success rate summary. 5) Create quantitative phenotype summary.

\endverbatim

```
Selection Menu: Summary file options
1) Create segregation and relative count summary files.
2) Create allele frequency summary table.
3) Count alleles and genotypes within groups.
4) Create genotyping success rate summary.
5) Create quantitative phenotype summary.
```

*Option 1) Create segregation and relative count summary files.*   
If your selected loci have an affection status trait locus (with only one liability class) first, then Option 1 will create a segregation table similar to this example one:

```
15 affected members with status 2.
0 unaffected members with status 1.
 Segregation Mating Table Counts:
Mo Fa              0           1           2 Seg Ratio Aff/(Unaff + Aff)
------           —         —         —  -------- --------
0 x 0 ->           0           0           0                 
0 x 1 ->           0           0           0                 
0 x 2 ->           0           0           4   100.000  100.000
1 x 0 ->           0           0           0                 
1 x 1 ->           0           0           0                 
1 x 2 ->           0           0           0                 
2 x 0 ->           1           0           6    85.714  100.000
2 x 1 ->           0           0           0                 
2 x 2 ->           0           0           2   100.000  100.000
```

At an affection status locus, a code of ’2’ means ’affected’, a code of ’1’ means ’normal’, and a code of ’0’ means ’unknown’. So this table provides a summary of the different mating types and what type of offspring they created. This provides a very simple overview of the data, and is not meant as a substitute for more sophisticated segregation analyses.  
  
If your selected loci have an affection status trait locus (with only one liability class) first, then Option 1 will also create a relative count summary file similar to this example file:

```
Summary Counts
There are 2 pedigrees, of which 1 are typed at 
one or more of 2 markers on chromosome 5
            All members  |  Affecteds   |      Sibships  Sib | Affected pairs with
                 Typed   |    Typed     |         Naff  pairs| kinship k/32
Pedigree      #   >1  All|   #   >1  All|   #  Min  Max      |   2    4    6    8 
   Ped 1:    11    0    0|   7    0    0|   3    2    2     3|   4    8    0    9 
   Ped 2:    10   10    9|   7    7    6|   4    1    2     1|   1    6    4    5 
  TOTAL      21   10    9|  14    7    6|                   4|   5   14    4   14 
Type of affected pair      Count    Kinship
 sib pairs                     4      8/32
 parent-child pairs           10      8/32
 halfsib pairs                 0      4/32
 avuncular pairs               6      4/32
```

This provides a quick count of the types of affected relative pairs that are contained in the pedigrees. However, please be aware that it counts an affected relative pair whether or not both members are genotyped. If you need a count of *genotyped* affected relative pairs, then this can be generated via the SAGE option of Mega2.  
  
*Option 2) Create allele frequency summary table.*   
For each co-dominant marker, this option will produce an allele frequency table. Here is an example one:

```
Chromosome 5 : locus M2 with 2 alleles
There are 2 pedigrees containing 22 individuals,
of whom 10 are genotyped.
Heterozygosity Frequency   Count
     Observed  0.8000          8
     Expected  0.5000          5
Allele Frequency Table:
Allele#   Input    Observed  Count
     1    0.50000  0.50000      10
     2    0.50000  0.50000      10
Total Alleles (observed)   =    20
Here is the GENOTYPE DISTRIBUTION of all known genotypes:
OBSERVED count 
EXPECTED count
allele #
1) 1
   2
  
2) 8 1
   5 2
  
   1 2 <- allele #
```

For each marker, this table provides information on how many alleles were seen, how many individuals were genotyped, what the observed and expected heterozygosity frequencies are, and the observed and input marker allele frequencies (where the marker allele frequencies are estimated simply by counting without regard to relationship). There is also a table giving the observed and expected genotype counts. In the example table above, there were 8 ’1/2’ genotypes observed in the data, but under Hardy-Weinberg equilibrium we would expect to see only 5 such heterozygotes, given the observed allele frequencies.  
  
Option 3) *Count alleles and genotypes within groups.*   
If the first locus in the selected locus order is an affected status trait locus, then this option will produce, for each affection status category (or affection status-liability class category) two tables per marker, the first giving the counts of alleles within each category, and the second giving the counts of genotypes within each category. There are some options as to how the summary file should appear:

```
Summary file output options :
0) Done with this menu - please proceed
 1) Display percentages in addition to counts [yes]
 2) Include rows with all zero counts [no]
 3) Include columns with all zero counts [no]
 4) Output tab-text file [no]
```

Sub-option 4 here, when toggled on, will result in the production of tab-delimited text files, one for each sub-table, that can easily then be read into most any statistical package for further analyses.  
  
Here is an example summary file produced by this option:

```
Trait TRAIT
      Allele counts for marker  M2
M2     Unknown       Affected    Total    Total Freq
    1        2   0.3333     8   0.5714    10     0.5000 
    2        4   0.6667     6   0.4286    10     0.5000 
TOTAL        6             14             20
      Genotype counts for marker  M2
M2     Unknown       Affected    Total     Total Freq
 1/ 1        0   0.0000    1   0.1429     1      0.1000
 1/ 2        2   0.6667    6   0.8571     8      0.8000
 2/ 2        1   0.3333    0   0.0000     1      0.1000
TOTAL        3             7             10
```

HINT: If one has case-control data, one can use Mega2 to generate the appropriate case-control counts by setting up an affection status locus and coding “cases” as “affected” and “controls” as “unknowns” (or “normals”). Likewise, if one has multiple categories (*e.g.,* high, medium, low BMI), one can generate the proper tables with this option of Mega2 by simply setting up a dummy affection status locus with multiple liability classes, one for each category.

*Option 4) Genotyping success rate summary .*   
This creates a summary of the genotyping success rate within each individual for each marker locus, and within each marker for the number of individuals genotyped at that marker. The output file is called *genotyping\_rate.##* . Here is an excerpt form a typical output file:

```
---------------------
Thu Jun  6 14:18:47 2002
Input file names
locus file:    datain.01
pedigree file: pedin.01
map file:      map.01
Untyped pedigree option: Include all pedigrees whether typed or not
---------------------
 Genotyping success rate summary for chromosome 1
---------------------------------------------------
Per person genotyping rate :
Number of markers: 4
Maximum number of markers typed 4
Minimum number of markers typed 0
---------------------------------------------------
Ped      Person   #Markers typed  Success Rate
---------------------------------------------------
3         1                   4         1.00
3         2                   4         1.00
3         3                   4         1.00
3         4                   4         1.00
13        1                   4         1.00
13        2                   4         1.00
13        3                   4         1.00
13        4                   4         1.00
7         1                   4         1.00
:	  :		      :		:
Per marker genotyping rate:
Total number of individuals: 384
Maximum number of individuals typed at a marker 364
Minimum number of individuals typed at a marker 356
---------------------------------------------------
Marker      Alleles   #Individuals   #Percentage
                       Typed          Typed
---------------------------------------------------
d1s196            4            356          0.93
d1s238            4            364          0.95
d1s229            4            364          0.95
d1s103            4            364          0.95
---------------------------------------------------
```

*Option 5) Quantitative phenotype summary .*   
This creates a summary of phenotype values for selected quantitative loci present in the pedigree file. The user can create separate file for each quantitative trait locus or a combined file for all loci. The output file is called *phenotyping\_rate.##* . Here is an excerpt form a typical output file:

```
---------------------
Mon Mar  8 15:04:59 2004
Input file names
locus file:    datain.06
pedigree file: pedin.06
map file:      map.06
omit file:     omit.06
Untyped pedigree option: Include all pedigrees whether typed or not
---------------------
Phenotype Summary for Q1
---------------------------------------
                        All                             Founders
Pedigree        Members Phenotyped       %Phenotyped    Total   Phenotyped      %
1               52              37              0.71    17      8               0.47
2               86              69              0.80    24      13              0.54
3               100             66              0.66    30      10              0.33
4               90              57              0.63    27      12              0.44
5               63              34              0.54    20      4               0.20
6               52              34              0.65    16      5               0.31
7               74              48              0.65    21      6               0.29
8               68              43              0.63    21      7               0.33
9               41              27              0.66    14      5               0.36
10              60              37              0.62    19      7               0.37
```

\hrule

## 25.14  Convert to SAGE format

Suppose the chromosome number is 5. Then this option will generate the following files:

```
        Pedigree file:         sage_ped.05
        Locus file:            sage_loc.05
        FSP parameter file:    sage_par.05
        SIBPAL parameter file: sage_sibpal.05
        SIBPAL C-shell file:   sage.05.sh
        Genotype count file:   sage_cnt.05
        FCOR parameter file:   sage_cntpar.05
        FCOR C-shell file:     sage_cnt.05.sh
```

Reminder: This supports the old FORTRAN-like SAGE format (rather than the new more elegant format now available in beta-version which is supported by option 22).  
Provided you have selected only one trait locus (affection status or quantitative), the C-shell script file ’sage.05.sh’ will carry out Haseman-Elston linkage analyses of the trait versus each of the markers using the SAGE programs fsp and sibpal. This uses the files sage\_ped.05, sage\_par.05, sage\_loc.05, and sage\_sibpal.05.  
The second C-shell script file ’sage\_cnt.05.sh’ is designed to provide a count of all genotyped relative pairs (where an individual is considered genotyped if they are genotyped at one or more markers in the input file). The ‘sage\_cnt.05’ file is a SAGE pedigree file which will contain, for each affected, a ‘trait’ which is the total number of genotypes in the input file (*before* any locus re-ordering) for each affected individual. The script ’sage\_cnt.05.sh’ first runs fsp on sage\_par.05 and sage\_cnt.05, and then runs fcor using sage\_cnt.05 and fsp-generated files.   
NOTE: The affected relative pair counts generated by SAGE may differ from those generated with Mega2, because Mega2 will only count a relative pair as “full sibs” if in fact they are full sibs and their parents are not inbred.   
You may use other SAGE modules as desired (See the SAGE documentation). For example, if you wanted to compute familial correlations, you would first run fsp and then run fcor, as follows:  
To run fsp,

```
cp sage_par.## fort.1
cp sage_ped.## fort.11
fsp
```

To then run fcor, create the appropriate ‘fcor.par’ parameter file and then:

```
cp fcor.par fort.1
cp sage_ped.## fort.11
cp fort.22 fort.12
fcor
```

If you make an affection status locus (with no liability classes) your first locus, then the ‘Pairs Used’ in the FCOR fort.22 output file will contain counts of affected relative pairs in your pedigrees (whether or not they are genotyped).

\hrule

## 25.15  Set up for TDTMax analyses

This option permits easy setup of files for the ‘tdtmax’ program from this paper:   
Ann Hum Genet 1997 Jan;61( Pt 1):49-60   
Randomization tests of disease-marker associations.  
Morris AP, Curnow RN, Whittaker JC  
  
This approach is very nice in that it uses randomization approach to correct for the problem of multiple testing (across alleles) inherent to the TDT approach for testing for association. However, Dr. Morris seems not to be distributing his program anymore, and so now the ASPEX program may be used instead, as the ASPEX program now uses randomization in a similar way to compute appropriately corrected empirical p-values.   
The shell scripts generated assume that you have the ‘convert’ and ‘tdtmax’ programs installed and in your path.   
The TDTMAX option will create a plethora of files, one per marker, with the default name “tdtmax\_data.#.locus\_name”, where “#” is the chromosome number and “locus\_name” is the name of the marker.   
It will also create a C-shell script with the default name “tdtmax#.sh”, where “#” is the chromosome number.  
You also have to choose between these two analysis options.  
1) all affected offspring in families  
2) only one affected sib per family  
  
Please see the ‘tdtmax’ documentation for a discussion of these options. However, suffice it to say here that the “all affected offspring in families” uses transmitted and non-transmitted alleles from all affected offspring. The authors of tdtmax advise against this option, and instead recommend that one uses only one affected sib per family (NOTE: the tdtmax documentation does not seem to indicate how this one sib is selected.).   
You also need to enter the number of permutations desired. Obviously, one should use as many permutations as is computationally feasible.   
  
Output from the ‘tdtmax#.sh’ C-shell script file:  
The C-shell script file will, if all goes well, create (or append to) two output files:  
a) tdtmax.lst - This file contains the complete output generated by the ‘tdtmax’ program.   
b) tdtmax.sum - This file contains summary output, one line per marker, giving the marker name, the position on the chromosome, the chromosome number, and the empirical tdtmax p-value.

\hrule

## 25.16  Convert to SOLAR format

Mega2 creates one set of files per chromosome in the SOLAR input format, along with a combined Tcl script for all chromosomes, e.g.:

```
        Pedigree file:         solar_ped.06
        Allele frequency file: solar_freq.06
        Phenotype file:        solar_phen.06
        Genotype file:         solar_marker.06
        Map file:              solar_map.06   
        Pedigree file:         solar_ped.07
        Allele frequency file: solar_freq.07
        Phenotype file:        solar_phen.07
        Genotype file:         solar_marker.07
        Map file:              solar_map.07  
	    Tcl script:            solar_load.all.tcl
```

SOLAR allows any ordering of traits versus markers, since trait phenotypes are written in a separate file from marker genotypes. It allows QTLs as well as affection status loci. If no trait is selected the phenotype file is not created.   
The Tcl script is loaded automatically when SOLAR is invoked within the directory containing the SOLAR format files. This script provides Tcl functions to load in the pedigree, map, phenotype and genotype data for each chromosome separately. For instance, if the user created files for chromosomes 6 and 7, then the Tcl script contains the functions load6() and load7().

\hrule

## 25.17  Convert to Vitesse format

The Vitesse option offers a choice of two kinds of output, MLINK and LINKMAP. In the case of MLINK, a trait locus is analyzed against every marker, so we obtain one set of pedigree and locus files per marker locus. A C shell script is also created that runs Vitesse.

```
         Pedigree file       vpedin.M1
         Locus file          vdatain.M1
==========================================================
         Pedigree file       vpedin.M2
         Locus file          vdatain.M2
         Cshell file          vitesse.06.sh
```

These files were created for a set of two markers and a trait.  
  
In the LINKMAP mode, the user can select a specific set of linked markers, in order to compute a LOD score for the trait at each such marker. Otherwise, the user can elect to analyze moving windows of specified length along each chromosome for the same purpose. Mega2 produces multiple sets of pedigree and locus files for each interval and for each position of the trait in that interval. A C shell script is also written out to run Vitesse on all this data.

```
         Pedigree file       vpedin.56_3.06
         Locus file          vdatain.56_3.06
         Cshell file         vitesse.06.sh
```

This set, for example, refers to an interval consisting of marker 5, and 6, and the trait placed to the right of 6th marker.   
QTLs are allowed as well as affection status loci, and the trait has to occur at the beginning of the order.

\hrule

## 25.18  Convert to Linkage format

This option creates files in LINKAGE format. This is useful for reordering loci or selecting subsets of loci from large files. Files can be created one set per chromosome or as combined output files. They consist of:

```
        Pedigree file:        Lpedin.01
        Locus file:           Ldatain.01
```

As might be expected, there are no restrictions on trait and marker selection and reordering. However, the recombination fractions at the bottom of the locus file corresponds to the input map file (not the original input locus file). \hrule

## 25.19  Test loci for Hardy-Weinberg Equilibrium

This option permits one to test markers for Hardy-Weinberg equilibrium. The user can select one out of five HWE testing programs,   
1) HWE by Guo and Thompson   
2) GEN program by Lazzeroni and Lange   
3) Chi-sq test from the R “genetics” library by Warnes and Leisch  
4) Exact test for bi-alleleic markers from the R “genetics” library   
5) Mendel 5 by Lange et al.  
  
Use of options 3 and 4 require R to be installed along with the “genetics” library as well its supporting libraries: combinat, gregmisc and MASS. The user also has a choice of individuals selected estimation of allele and genotype frequencies:

```
Individual selection menu for counting alleles
==========================================================
0) Done with this menu, please proceed.
 1) Genotyped founders only
*2) Genotyped founders + a randomly chosen genotyped person
    from pedigrees without genotyped founders.
 3) All genotyped individuals
Select from options 0 - 3 >
```

The “HWE” program has been incorporated into the Mega2 program by the authors’ permission, however, the user has to obtain the other programs separately.  
For “HWE” and “GEN”, two files are created, one is a long format output containing the combined output from either program for each marker, and the other file is a summary table produced by Mega2. They are called:   
HWE files:

hweresults.01 hwetable.01

\endverbatim

```
    hwe_results.01
    hwe_table.01
```

GEN files:

```
    gen_results.01
    gen_table.01
```

The Perl scripts make\_hwe\_table.pl and make\_gen\_table.pl create the summary tables from the long format output file. here is an example table format output file:

```
These results are produced by the HWE program by
Sun-Wei Guo and Elizabeth Thompson.  If you publish
any of these results,please cite:
 
Guo, S.-W., Thompson, E. T. (1992)
Performing the Exact Test of Hardy-Weinberg Proportion
for Multiple Alleles. Biometrics 48:361-372
---------------------
Mon Feb 12 14:27:44 2001
Input file names
locus file:    datain.01
pedigree file: pedin.01
map file:      map.01
Counted individuals consist of:
    Genotyped founders or a randomly chosen genotyped person from each pedigree
---------------------
 
 
Marker       Alleles   Expected      Observed       P-value    Std Error   #Genotypes
                       Homozygosity  Homozygosity
 
d1s228             4       0.3331       0.2472        0.5669      0.0050       89
d1s234             4       0.2842       0.1379        0.0025      0.0005       87
d1s255             4       0.3591       0.3297        0.7339      0.0045       91
```

Options 3 and 4 set up data files that can be read into R, as well as an R script for invoking the appropriate function to test for HWE. There is a separate table format data file for each marker, named hwe\_genos.[marker\_name] containing the genotypes of each individual selected for HWE estimation. Mega2 also runs the R script automatically, and creates summary tables, hwe\_chisq\_table.[chromosome] and hwe\_exact\_table.[chromosome] respectively. If more than one chromosome is present, then the extension is “.all”, instead of a chromosome number.   
Here is an excerpt from the table produced by the chi-sq test:

```
             max(exp-obs) max2(exp-obs) Chi-sq P-value
d21s258      5.948276      5.689655     13.815 0.139
d21s265      8.555743      4.623311     12.165 0.243
d21s219      9.924051      8.799051     37.573 0.001
d21s65       8.454545      7.501420     18.647 0.031
d21s167      4.734722      4.391667     32.483 0.001
```

The two largest differences between expected and observed genotype frequencies are output in this table along with the chi-sq test statistic and p-value.

For option 5, Mega2 sets up Mendel format pedigree, locus and map files containing all markers selected for testing. There is also a Mendel5 format control file to run the analysis. Mega2 does not automatically run Mendel. The Mendel format output files are called:

```
    Pedigree file:      hwe_mendel5_ped.all
    Locus file:         hwe_mendel5_loc.all
    Map file:           hwe_mendel5_map.all
    Control file:       hwe_mendel5_control.all
```

\hrule

## 25.20  Convert to Allegro format

This option creates the files needed for analysis via the Allegro program. The pedigree and locus files are similar to the GeneHunter format files. Allegro supports only one trait locus per analysis, and only binary traits. The following files are created per chromosome in this option:

```
        Command file       : al_in.01
        Locus file         : al_dat.01
        Pedigree file      : al_ped.01
        C-shell script     : al_script.01.sh
```

The command file contains parameters that specify the Allegro input files and the analyses that must be performed on these files, e.g.:

```
PREFILE al_ped.01
DATFILE al_dat.01
MODEL mpt par het allegro_par_mpt.01
MODEL spt par het allegro_par_spt.01
MODEL mpt exp pairs equal allegro_exppairs_mpt.01
MODEL spt exp pairs equal allegro_exppairs_spt.01
MODEL mpt exp all equal allegro_expall_mpt.01
MODEL spt exp all equal allegro_expall_spt.01
MODEL mpt lin pairs equal allegro_linpairs_mpt.01
MODEL spt lin pairs equal allegro_linpairs_spt.01
MODEL mpt lin all equal allegro_linall_mpt.01
MODEL spt lin all equal allegro_linall_spt.01
```

This is the standard file produced by Mega2. \hrule

## 25.21  Convert to MLBQTL format

This creates files for analysis by MLBQTL. The pedigree and locus files are identical to the GeneHunter format. However, extended pedigrees are first converted to nuclear families. Each chromosome-specific set of files includes:

```
        Command file       : mlb_in.06
        Locus file         : mlb_dat.06
        Pedigree file      : mlb_ped.06
        C-shell script     : mlb_script.06.sh
```

\hrule

## 25.22  Convert to S.A.G.E. 4.0 format

S.A.G.E 4.0 files created by Mega2:

```
        Pedigree file:         sage4_ped.06
        Locus file:            sage4_dat.06
        Parameter file:        sage4_par.06
        Map file:              sage4_map.06
```

The pedigree file is identical to that produced by the older version of S.A.G.E., except that it does not contain the analysis name at the start of each record. The locus file formats are the same. The map file is a new addition. It lists marker loci and the distances between them:

```
genome, map=Haldane
{
  region=chr6
  {
    marker = D06G025
    theta = 0.04431213
    marker = D06G028
    theta = 0.0381648
    marker = D06G034
    theta = 0.01400026
    marker = D06G035
    theta = 0.05306726
    marker = D06G041
    theta = 0.02094853
    marker = D06G043
  }
}
```

The parameter file contains information about the pedigree file records. It should also contain an “analysis” description section at the bottom which is not currently generated by Mega2. The user should make sure to insert his/her own sections inside the parameter file.   
The pedinfo program can by run on the Mega2-generated files straightaway, without any modifications.   
S.A.G.E. 4.0 flags the pedigree file currently produced for X-linked data as inconsistent. This is seen in male offspring which are written down as homozygotes by Mega2. When we are better aware of how S.A.G.E. 4.0 handles X-linked marker data, Mega2 will also be updated with the correct format. Meanwhile, the user should use this option only for autosomal and pseudo-autosomal data.

## 25.23  Convert to pre-makeped format

Pre-makeped files created by Mega2:

```
        Pedigree file:         Ppedin.06
        Locus file:            Pdatain.06
```

This is analogous to the Linkage format option which generates post-makeped format files. If the input pedigree file was in post-makeped file with broken loops, this option will reconnect the loops.

## 25.24  Convert to Merlin-SimWalk2 format

Output files created by Mega2 for Merlin:  
Note that the Merlin format file have new names.

```
        Pedigree file:        sw2merlin_ped.06
        Locus file:           sw2merlin_data.06
        Map file:             sw2merlin_map.06
	    Frequency file:       sw2merlin_freq.06
        Order file:           sw2merlin_order.06
        Perl script file:     sw2merlin2sw2.pl
```

Output files created by Mega2 for SimWalk2-NPL:

```
        Locus file:           LOCUS.06
        Pedigree file:        PEDIGREE.06
        Penetrance file:      PEN.06
        Simwalk2 batch file:  BATCH2.06
        C-Shell script:       npl.06.sh
```

In addition files are created which will generate R-graphics plots after the SimWalk2 analysis has been run. These are:

```
        Perl script file:     Rsimwalk2.pl
        R-script file:        Rsimwalk2.R
        Shell file to run R:  Rsimwalk2.sh
```

The C-shell script npl.06.sh runs Merlin on the Merlin format files to create exact npl scores for pedigrees which are amenable to exact computations i.e. do not require a great deal of computation. Merlin can be instructed to limit the amount of time spent on each pedigree by means of the *--minutes* switch. Currently, this value is set to **1 minute** inside the Pedigrees that cannot be handled by Merlin are then analyzed by SimWalk2’s NPL option, and Merlin’s exact scores incorporated into the final STATS.06.ALL file which is the output file created by SimWalk2.   
At this point the Rsimwalk2.sh script should be invoked to generate postscript plots using R from the SimWalk2 output.

## 25.25  Convert to PREST format

Output files created by Mega2 for PREST:

```
        Pedigree file : prest_ped.06
        Chrom file    : prest_chrom.06
        Shell file    : prest_script.06.sh
        Locus file    : prest_loc.06
        Genotype file : prest_geno.06
```

If there are multiple chromosomes, the following files are created::

```
        Pedigree file : prest_ped.all
        Chrom file    : prest_chrom.all
        Shell file    : prest_script.all.sh
        Locus file    : prest_loc.02
        Genotype file : prest_geno.02
==========================================================
        Locus file    : prest_loc.03
        Genotype file : prest_geno.03
==========================================================
        Locus file    : prest_loc.04
        Genotype file : prest_geno.04
==========================================================
        Locus file    : prest_loc.05
        Genotype file : prest_geno.05
==========================================================
```

The shell script prest\_script.all.sh runs PREST over the marker data on the selected chromosomes to produce three output files:   
prest\_out1, prest\_out2 and prest\_out3  
and writes errors (if any) into prest\_error.

## 25.26  Convert to PAP format

Output files created by Mega2 for PAP:

```
        Pedigree file :   trip.06
        Header file :     header.06
        Phenotype file :  phen.06
        Population file : popln.06
        C-shell file :    pap_script.06.sh
```

The C-shell script simply copies the rest of the files into files with the proper names as required by PAP, e.g. trip.06 into trip.dat, header.06 into header.dat and so on. This option creates separate sets of files for each chromosome.

## 25.27  Convert to Merlin format

The Merlin format locus file used to be in Linkage format. It was brought to our attention that for a linkage format locus file, Merlin ignores the frequency and map files, even if they are specified via the -f and -m options. It uses allele frequencies and recombination fractions from the locus file instead. Therefore, we have now modified Mega2 to create a QTDT format file instead, which is identical to the “names” file used by Mega2, so that Merlin is forced to use the frequency and map files created by Mega2.

Output files created by Mega2 for Merlin:

```
        Pedigree file :   merlin_ped.06
        Locus file :      merlin_dat.06
	    Map file:         merlin_map.06
	    Frequency file:   merlin_freq.06
	    C-shell script:   merlin.06.sh
```

Merlin also sets up the following files for creating LOD score curves:

```
        R-script file:        Rmerlin.01.R
        Shell file to run R:  Rmerlin.01.sh
```

Merlin files are created separately for each chromosome except for the shell-script, which is created only once. This option also allows the user to choose which pedigree and person field to select. See the section on “using Ped, Per and ID identifiers in the pedigree file” .   
Merlin now allows the user to enter a string containing valid Merlin analysis options selected from a list displayed at the beginning of this menu. (these can also be seen at the beginning of each run of Merlin). Some of these options such as --steps, --grid etc. should be followed by a numeric argument, and Mega2 checks for this as well. Merlin graphics are only set up in --markerNames option is not selected, and if either or both of the linkage options are selected (--npl and --pairs).

Please note that Merlin requires a sex-averaged map as well when the user sets up for sex-specific analysis (Value\_Genetic\_Distance\_SexTypeMap = 1 93↑).

## 25.28  Convert to Loki format

Output files created by Mega2 for Loki:

```
     Pedigree file:               Loki_ped.01
     Frequency file:              Loki_freq.01
     Map file:                    Loki_map.01
     Marker control file:         Loki_locus.01
     Link control file:           Loki_link.01
     Overall Control file:        Loki_control.01
     Overall Parameter file:      Loki_param.01
     C-shell script:              Loki.01.sh
```

If multiple chromosomes are selected, Loki sets up chromosome-specific analyses as well as a combined analysis. In addition to the files above it will set up the following:

```
     Combined Control file for all chromosomes:   Loki_control.all
     Combined Parameter file for all chromosomes: Loki_param.all
```

Combined analysis also requires the chromosome-specific files described above.This option also allows the user to choose which pedigree and person field to select. See the section on “using Ped, Per and ID identifiers in the pedigree file” .

## 25.29  Convert to Mendel format

Starting with version 4.2, Mega2 creates only in CSV format (comma-separated values).

Output files created by Mega2 for Mendel7:

```
     Pedigree file:               mendel_ped.01
     Map file:                    mendel_map.01
     Definition file:             mendel_locus.01
     Control file:                mendel_control.01
     Penetrance file:             mendel_pen.01
```

Note that the locus and variable files are now combined into a single definition file . In addition, the penetrance file is not created if there are no affection status loci.   
 **Handling trait penetrances** If all affection traits selected for output have a single liability class, the penetrance file is not written. Rather a simple penetrance model is defined inside the Mendel control file. However, if one or more affection status loci have multiple liability classes, these are written separately into a penetrance file with three pentrances per person, whose affection status in known. If the affection status is missing, then this person is assigned a penetrance of 1.0 for all three possible underlying genotypes.   
This is in keeping with the latest Mendel documentation, and you should fully understand how Mendel treats affection data before you set up files for Mendel using Mega2.

This option also allows the user to choose which pedigree and person field to select. See the section on “using Ped, Per and ID identifiers in the pedigree file” .

## 25.30  Convert to SUP format

Output files created by Mega2 for SUP:

```
        Pedigree file:        sup_simped.01
        SLINK locus file:     sup_simdata.01
        SUP locus file:       sup_locus.all
        SLINK control file:   slinkin.01
        SUP shell file:       sup.01.sh
```

If the pedigree data contains either loops (pre-makeped format) or loop-breakers (post-makeped format), then three additional files are created:

```
        Mega2 locus file:     sup_mega2_locus.01
        Mega2 map file:       sup_mega2_map.01
        Mega2 batch file:     sup_mega2_batch.01
```

## 25.31  Convert to PLINK format

Output files created by Mega2 for PLINK:  
To make running PLINK easier, we have adopted the PLINK convention for file naming rather than the Mega2 convention. Thus, the “fam”, “map”, and “lgen” file have a common prefix, as below:

```
        Pedigree file:    plink.05.fam
        Map file:         plink.05.map
        Genotype file:    plink.05.lgen
```

If there is a single trait, the phenotypes are written into the “fam” file. If multiple traits are combined into one output, these are written into a separate phenotype file named:

Phenotype file: plinkpheno.05

\endverbatim

The shell script for running PLINK is named:

Shell file: plinkshell.05

\endverbatim

Since Family and phenotype information do not depend on marker and genotypes, we have saved on space requirements by creating an overall family and phenotype file where output includes multiple chromosomes. Then the shell script automatically creates symbolic links to these files with the appropriate names in order to run chromosome-by-chromosome analysis.

This option allows the user to choose which pedigree and person field to select. See the section on “using Ped, Per and ID identifiers in the pedigree file” . \hrule

## 25.32  Convert to Cranefoot format

Output files created by Mega2 for Cranefoot 3.2.3:  
The following chromosome specific files are created for Cranefoot:

```
        Pedigree file:    crnft_ped.05
	    Control file:     crnft_control.05
```

If there are multiple chromosomes in the data, the combined shell script is named

C-shell file: crnftshell.all.sh

\endverbatim

Chromosome-specific shell scripts are named:

C-Shell file: crnftshell.05.sh

\endverbatim

This option allows the user to choose which pedigree and person field to select. See the section on “using Ped, Per and ID identifiers in the pedigree file” . \hrule

## 25.33  Convert to Mega2 format

The following chromosome specific files are created for Mega2 format:

```
     Pedigree file:               pedin.01.mega2
     Names file:                  names.01.mega2
     Map file:                    map.01.mega2
     Frequency file:              frequency.01.mega2
     Penetrance file:             penetrance.01.mega2
```

Note that the default output file names have been chosen to match the Mega2 input file naming convention. Thus, within a subsequent run, Mega2 will easily detect these as valid input files if you specify the extension “01.mega2” in the input menu. If multiple chromosomes are combined within a single set of output files, then the extension “all” will be used instead of chromosome numbers. This option allows covariates, and also supports the selection of different individual and pedigree ID fields.

## 25.34  Convert to IQLS/Idcoefs format

The following chromosome specific files are created for IQLS/Idcoefs:

```
     IQLS pedigree file:        IQLS_pedigree.05
     IQLS marker file:          IQLS_marker.05
     IQLS parameter file:       IQLS_parameter.05
     IQLS shell script file:    IQLS.05.sh
     Idcoefs pedigree file:     Idcoefs_pedigree.05
     Idcoefs study file:        Idcoefs_study.05
```

## 25.35  Convert to FBAT format

The following chromosome specific files are created for FBAT:

```
     FBAT phenotype file:   fbat.phe
     FBAT R code file:      fbat.R
     FBAT shell file:       fbat.all.sh
     FBAT commands file:    fbat.cmd.txt

     FBAT pedigree file:    fbat.05.ped
     FBAT shell file:       fbat.05.sh
     FBAT R hdr file:       Rfbat.05.hdr
     FBAT map file:         fbat.05.map
```

The first file will be created only if there is more than one trait. The next file is an R program that parses the default FBAT output and produces graphs of the significance. The *fbat.all.sh* script will run the others scripts produced for all the chromosomes and traits. The *fbat.cmd.txt* script issues the commands for FBAT analyses. It is expected that you will change this file to suit your needs.

The next set of 4 files get repeated for each chromosome (if requested) and for each trait (if requested). The *ped* and *map* files satisfy requirements of FBAT. The *sh* file runs FBAT with the *ped* and *map* input performing the *fbat.cmd.txt* tasks and then runs the **fbat.R** program to collect and plot the results. Finally, the *hdr* file is used by **nplplot**to provide arguments to the plot.

Note: The FBAT program requires that an affection trait be specified. Mega2 will choose the first affection phenotype from the phenotype list for FBAT’s affection. If all the phenotypes are quantitative, an error will be given. Further, the trait selection menu can be used to select any affection trait and make it the first trait.

## 25.36  Convert to PANGAEA MORGAN format

“MORGAN (Monte Carlo Genetic Analysis) is a collection of programs and libraries developed at the University of Washington under the PANGAEA (Pedigree Analysis for Genetics and Epidemiological Attributes) umbrella. This software implements a number of methods for the analysis of data observed on members of a pedigree, with the main programs implementing Markov Chain Monte Carlo (MCMC) methods.” (Taken from the tutorial introduction.) The “Morgan tutorial” is available at http://www.stat.washington.edu/thompson/Genepi/MORGAN/morgan311-tut-html/morgan-tut.html; there are also example data files that are provided to match the tutorial.

The software download is available via http://www.stat.washington.edu/thompson/Genepi/MORGAN/Morgan.shtml We have used version 3.1.1 in our Mega2 development work. You should “*untar*” the release file, **morgan311\_release.tgz,** (in our case) into a convenient directory and “*make*” the software. The are several *README* files that explain more of the details of **MORGAN**.

One very useful thing to note is that in each subdirectory that contains one or more executable programs (as opposed to libraries), there is a “*Gold*” subdirectory that contains tests for the programs. By necessity, these “*Gold*” directories also contain sample parameter files for these programs. In addition, the directory that contains the “Gold” subdirectory also contains a “README\_userdoc” file that explains the programs and shows all the possible parameters for the programs. There are many dozens of possible options that can be present in a parameter file.

All of the **MORGAN** programs accept the same format data files: a pedigree file, a marker file, and a parameter file; Mega2 will produce these files for you. We have chosen several representative **MORGAN** programs to produce data and parameter files for rather than for all the **MORGAN** programs: we support: *pedcheck*, *kin*, *translink*, *lm\_linkage*, *lm\_bayes*, and two versions of *lm\_ibdtests* one using likelihood-ratios and the other not. Our design is intended to make it easy for a user to take one of these existing examples and just replace the program to be run to get different **MORGAN** functionality. We also have split the parameter files into two subfiles. One that refers to the pedigree and marker data and the other has the parameters for the desired program to be run. We do not expect to have specified all the parameters that someone might want in all cases and expect that the user will change and customize the parameters as needed. We have chosen default parameter values based on the examples provided in the “Gold” directories of the **MORGAN** distribution - the user should carefully tune the parameter values to their particular data set and desired analysis.

We provide shell scripts to run **MORGAN** programs for all the traits and chromosome selected by Mega2. These scripts must find the appropriate program. We require that the environment variable **MORGAN\_RELEASE** be set to the directory that **MORGAN** release was *untared* into. Please see the section on **Beagle** (below) for a discussion of several strategies for setting an environment variable.

One final note: In principle, **MORGAN** allows a single marker file to have data from different chromosomes. But not all the **MORGAN** programs support this extension. We have chosen not to implement the feature. So all output generated by Mega2 will have a separate marker file for each chromosome.

## 25.37  Convert to Beagle format

The following chromosome specific files are created for Beagle:

```
BEAGLE shell top file:      beagle.all.sh

BEAGLE bp marker file:      beagle.01.pmrk.gz
or
BEAGLE gd marker file:      beagle.01.gmrk.gz

BEAGLE genotype file:       beagle.01.bgl.gz
BEAGLE shell file:          beagle.01.sh
```

Beagle is used for imputing genotypes, inferring haplotype phase, and performing genetic association analysis. Since Beagle can read gzipped files, the marker and genotype files are output in gzip format for those platforms that support it.

**Important:** Note that to get correct results when converting to Beagle, your input data must contain the appropriate data. If you wish to set up data in the “unphased unrelated” Beagle format, then your input data must consist of unrelated case/control individuals. If you wish to set up data in the “unphased trio” Beagle format, then your input data must consist of parent-child trio families with unaffected parents and affected children.

Mega2 supports three of the valid Beagle genotype files (unphased unrelated, unphased trio, and unphased pair) through the sub-analysis options for Beagle (See 86↑). Beagle marker files can contain either physical or genetic map information. Both genetic (.gmrk.gz) and physical (.pmrk.gz) map files will be output if the maps are available on input. However, if both physical and genetic maps exist, the genetic map will be used by the shell file produced by Mega2 . You may change this by editing the shell file.

Beagle is written in Java and is distributed in the form of a Java ARchive (.jar) file as ’beagle.jar’. The shell script produced by Mega2 assumes that the Beagle .jar file is referenced by the shell environment variable BEAGLE\_JAR (which defaults to a file named ’beagle.jar’ in the current directory). The shell script will generate an error if the .jar file is not found.

Here are some examples of how to set the BEAGLE\_JAR environment variable so that the shell script may locate the beagle .jar file in a different directory other than the current directory. These examples assume that you have installed the Beagle distribution at the location ’/usr/local/src/beagle’.

If you are using a Bash or Korn shell, then you would use:

export BEAGLE\_JAR=/usr/local/src/beagle/beagle.jar

If you are using a C shell (csh), then you would use:

setenv BEAGLE\_JAR /usr/local/src/beagle/beagle.jar

If you are using using Windows, then the setting of the environment variable is implementation dependent. It is generally done through the “User variables” section of the “Environment Variables” button located on the “Advanced” tab of the “System Properties” of the “Control Panel” item. Once it is set it is retained when the user logs out and in again.

In practice the environment variable should be placed in a file to be defined once on user login. This file is dependent on the shell that you are using. For the Bash shell it is the user’s .bash\_profie file; for the C shell it is the user’s .login file; for the Korn shell it is the user’s .profile file.

If you encounter a “Java heap space” exception while running Beagle, please consult the “Memory Management” section of the Beagle documentation for further assistance.

Please note that Unphased pair and trio data is not permitted to have any Mendelian inconsistencies. Genotypes causing Mendelian inconsistencies for a trio must be replaced with missing genotypes.

Please also note that Loop\_Over\_Chromosomes = y is assumed when converting to Beagle format files (See 90↑).

The genetic map output for Beagle will be in Haldane cM.

## 25.38  Convert to Eigenstrat format

The following chromosome-specific files are created for Eigenstrat:

```
EIGENSTRAT shell top file:  eigenstrat.all.sh

PLINK map file:             eigenstrat.01.pedsnp
EIGENSTRAT binary file snp: eigenstrat.01.bed
EIGENSTRAT pedigree file:   eigenstrat.all.pedind
or
PLINK map file:             eigenstrat.01.map
EIGENSTRAT pedigree file:   eigenstrat.01.ped

EIGENSTRAT shell file:      eigenstrat.01.sh
```

Eigenstrat will take as input several different file formats. The formats that Mega2 creates for Eigenstrat are either PLINK binary format or PLINK ped format. The PLINK binary format is more efficient. The analysis sub-option for this analysis mode is used to the type of output produced (See 86↑).

In order to successfully run Eigenstrat from the shell scripts produced by Mega2 the Eigenstrat bin directory must be put in the PATH. Here are some examples of how to set the PATH so that smartpca.perl and smarteigenstrat.perl may be found as suggested by the EIG4.2/EIGENSTRAT/README documentation file. These examples assume that you have installed the Eigenstrat distribution at the location ’/usr/local/src/EIG4.2’.

If you are using the Bash or Korn shell, then you would use:

export PATH=/usr/local/src/EIG4.2/bin:$PATH

If you are using the C shell (csh), then you would use:

setenv PATH /usr/local/src/EIG4.2/bin:$PATH

If you are using Windows, then the setting of the environment variable is implementation dependent. It is generally done through the “System variables” section of the “Environment Variables” button located on the “Advanced” tab of the “System Properties” of the “Control Panel” item. Once it is set it is retained when the user logs out and in again.

Eigenstrat was designed for the correction of trait values based on ancestry (population stratification). This implies that you need to specify a trait to produce a pedigree file. If no trait is provided, then Mega2 will exit with an error message stating that a trait must be provided.

Please note that if you wish to run the ’ploteig’ program, you will need to install ’gnuplot’ (home page is http://www.gnuplot.info).

## 25.39  Convert to Structure format

The following chromosome specific files are created for Structure:

```
STRUCTURE mainparams:       structure.01.mainparams
STRUCTURE data:             structure.01.data
STRUCTURE shell file:       structure.01.sh
```

Structure is used for inferring population structure using genotype data. Mega2 will produce files that are used by the "computational part" (sometimes referred to the structure kernel or the console) of the program “structure” that is written in C. On the “Download Structure 2.3.4” web page, to obtain the kernel version of ’structure’ you should use the links allowing you to “Download package without front end.” (See http://pritch.bsd.uchicago.edu/structure\_software/release\_versions/v2.3.4/html/structure.html).

The structure kernel reads two control files and one data file. Examples of both of these control files and a data file are included as a part of the structure download. One of the control files "extraparams" contains parameters that describes algorithm variable settings (e.g., how the program runs) and so is not of concern for our purposes in data file conversion. Mega2 will create an empty “extraparams” file if one does not already exist, but you may need to customize this file for your specific needs. The "mainparams" control file contains parameters that describe the data file used as input to structure, and so is of concern here. Mega2 will produce a data file which stores data for each individual on one line (rather than the default two lines per individual). Mega2 allows you to choose to associate a quantitative phenotype to a population identifier through the use of the batch file parameter Structure.PopDataPheno (See 123↑).

## 25.40  Convert to PSEQ (PLINK/SEQ) format

The following files are created for PSEQ:

```
PSEQ phenotype file:        pseq.phe
PLINK map file:             pseq.all.bim
PLINK pedigree file:        pseq.all.fam
PLINK binary file snp:      pseq.all.bed
PSEQ shell file:            pseq.all.sh
```

PLINK/SEQ is a program and library for working with human genetic variation data. It is independent of, but complementary to the PLINK package.

A shell script is produced that, when run, creates a new PLINK/SEQ project and loads the data files into the project-specific PLINK/SEQ database. The data are converted to PLINK SNP Major Binary format (e.g., .bed, .bim, .fam files) by Mega2 and loaded with the appropriate PLINK/SEQ commands. If additional phenotypes are present in the input data (since the .fam file can only hold one phenotype), a phenotype file is created, and also processed by the shell script that loads the data.

The PLINK/SEQ shell script takes two optional arguments. The first optional argument is the name of the PLINK/SEQ project that you are creating (the default value is ’pseq.01’). The project will be created in the directory in which the shell script is run. The second optional argument should be the path to the PLINK/SEQ resource directory (the default value is the project name appended with the string ’\_res’). For a more complete description of these concepts, please see the PLINK/SEQ documentation at http://atgu.mgh.harvard.edu/plinkseq/.

When the PLINK/SEQ shell script is run, after creating a new project, it copies the trio of PLINK files into the project “\_out” folder before importing them into the PLINK/SEQ project. As PLINK/SEQ does not make a copy of the PLINK data, the trio of PLINK files must remain in the project folder - do not move them or delete them.

# 26 Utilities included with Mega2

## 26.1 Converting linkage format files to Mega2 format - l2a.py

The Mega2 format consists of input files which contain white-space delimited tabular data with mandatory column headers for each column of the data. We encourage you to use this format, as it is much more robust to data errors than the linkage format-based input files, and also allows more types of data to be specified. Therefore, we have also provided a Python script to convert your existing linkage format data files to the Mega2 format. The l2a.py utility script is described in detail inside the Mega2 input files section.

## 26.2 Map making utilities

These map making utilities are very old, and we have not used these in a long time (Instead we currently use the Genetic Map Interpolator (GMI) available from https://watson.hgen.pitt.edu/register/). These utilities consist of awk scripts for creating map files.

|  |  |
| --- | --- |
| mapmarsh.awk | Script to convert a frameworkMap file created by the ’Build your own map’ option of the Marshfield Center for Medical Genetics web page ( http://www.marshmed.org/genetics/ ) to a Mega2 format map file. However, note that this will create a map file in Kosambi cM, whereas Mega2 expects a map file in Haldane cM. |
| mapout.awk | Script to convert from the Mega2 format map file into a format that is easier to edit. This format gives the distances between each pair of adjacent markers, so we will call it the ’intermarker format’. |
| kos2hal.awk | Script to convert from an intermarker format map file in Kosambi cM to a Mega2 format file in Haldane cM. |

**Example:**

Here I illustrate the commands I used to convert some framework map files downloaded from the Marshfield site (which are in Kosambi cM) into Mega2 format map files in Haldane cM.

Comments are in ’[ ]’ brackets.

**NOTES:**

1) Make sure you save the files from Marshfield as text-only. The ’mapmarsh.awk’ script will not work properly otherwise. 2) You may have to edit the files from Marshfield, as sometime some markers are missing or alternative marker names are used.

```
mapmarsh.awk frameworkMap.20 20 > mapk.20
mapmarsh.awk frameworkMap.21 21 > mapk.21
mapmarsh.awk frameworkMap.22 22 > mapk.22
```

[So now we have three chromosome specific map files in Kosambi cM. We will place these in one file using the ’cat’ command: ]

cat mapk.\* > map.kos

\endverbatim [Now we must edit the ’map.kos’ file to remove the extra internal title lines.]

vi map.kos

\endverbatim [ Convert this file into intermarker format:]

mapout.awk map.kos > mapin.kos

\endverbatim [ Convert the intermarker format file into a Mega2 format file in Haldane cM: ]

kos2hal.awk mapin.kos > map.hal

\endverbatim [ And here it is: ]

```
    Chr      Haldane cM  Name        Haldane  Theta  Kosambi
    20           0.000   D20S103      10.981 0.09859   9.990
    20          10.981   GATA149E11   14.146 0.12321  12.580
    20          25.128   D20S851       8.916 0.08166   8.240
    20          34.043   D20S604       6.707 0.06277   6.310
    20          40.751   D20S470      17.011 0.14419  14.840
    20          57.761   D20S478       8.904 0.08156   8.230
    20          66.666   D20S481      20.622 0.16899  17.590
    20          87.288   D20S480      18.243 0.15285  15.790
    20         105.531   D20S171     
    21           0.000   D21S1432     11.065 0.09926  10.060
    21          11.065   D21S1437     13.032 0.11472  11.680
    21          24.097   D21S1442     13.476 0.11813  12.040
    21          37.573   D21S1440      3.858 0.03713   3.720
    21          41.431   D21S2055     20.208 0.16623  17.280
    21          61.640   D21S1446    
    22           0.000   D22S420      17.553 0.14803  15.260
    22          17.553   D22S1174     10.101 0.09146   9.250
    22          27.654   D22S689       3.966 0.03813   3.820
    22          31.620   D22S685       3.977 0.03823   3.830
    22          35.597   D22S683      10.516 0.09484   9.600
    22          46.113   D22S445
```

**Other scripts:**

|  |  |
| --- | --- |
| mapin.awk | Script to convert an intermarker format map file (without the chromosome numbers indicated) into a Mega2 format map file. Handles only files with markers from a single chromosome. |
| mapin2.awk | Script to convert an intermarker format map file into a Mega2 format map file. Handles files with markers from multiple chromosomes. |
| mapmen.awk | Script to convert an intermarker format map file into a Mendel 4 format map file. |
| mapsage.awk | Script to convert a Mega2 format map file into a map file in the new SAGE format. |
| mapsum.awk | Script to rearrange a Mega2 format map file so that the locus name is in the first column. |

NOTE:

1. These script were developed quickly for my personal use, and so may not be very robust to unexpected input file formats. Please check the files that are made by these scripts carefully.
2. Under Linux, you will need to change the word ’nawk’ to the word ’gawk’ at the top of each file in order for these scripts to run.
3. The scripts must be executable. Use ’chmod +x script.awk’ to make the ’script.awk’ file executable.

## 26.3 Creating a Mega2 omit file

The script omit.awk can generate a mega2 format omit file from the error file produced by running Pedcheck. This is strongly recommended in order to identify and remove inconsistent genotypes from the pedigree data.

To use it, first run pedcheck to create a ’pedcheck.err’ error file. Then use omit.awk to parse pedcheck.err and reformat the information into Mega2 omit file format:

```
omit.awk pedcheck.err > omit.01
```

The resulting omit file will contain instructions to zero out all genotypes within a given pedigree for each marker that is Mendelianly-inconsistent. This approach is conservative, and works well when one has large numbers of small pedigrees. It does not work well if one has a few extremely large pedigrees, as then this approach ends up zeroing out an entire large pedigree at each Mendelianly-inconsistent marker.

## 26.4 Scripts to generate formatted output for Hardy-Weinberg test:

The distribution provides two Perl scripts “make\_hew\_table.pl” and “make\_gen\_table.pl”. These are used from within Mega2 to format the raw output produced by Guo & Thompson’s HWE program and Lazzeroni & Lange’s “GEN” program respectively. They should be placed in inside a directory that is in the user’s execution path, otherwise Mega2 will fail to create the appropriate tables.

# 27 List of third-party applications used by Mega2

1. R statistical package and R-libraries
2. Python
3. Perl
4. Awk
5. C-shell

For the most part Mega2 does not require external programs, except a few instances.

Below is a list of third-party tools, and the analysis options that require these applications.

For instructions on obtaining these programs, please consult the installation instructions.

## 27.1 R statistical package and its libraries

R can be obtained from the CRAN web-site.

R is required for creating LOD score plots for SimWalk2’s npl option, Allegro, Merlin-SimWalk2 option, and Merlin’s NPL and variance components options. This requires the **nplplot** library in addition to the base R libraries.

Two of the Hardy-Weinberg tests use R routines included in the R library **Genetics**. Please note that the HWE options require the following libraries also to be installed:

> |  |  |
> | --- | --- |
> | combinat | combinatorics utilities |
> | gdata | Various R programming tools for data manipulation |
> | genetics | Population Genetics |
> | gregmisc | Backward compatibility package for gregmisc bundle |
> | gtools | Various R programming tools |
> | mvtnorm | Multivariate Normal and T Distribution |

### 27.1.1 R Installation

The web page that explains the R installation procedure and contains the binaries is at:

```
http://cran.r-project.org/bin/windows/base/
```

## 27.2 Python

Mega2 includes a Python script to convert some Merlin analyses output into nplplot-readable files. These consist of Merlin’s NPL, parametric and VC options.

### 27.2.1 Python Installation

The web page that explains the Python installation procedure and contains the binaries is at:

```
http://www.python.org/getit/
```

There are several different binaries to choose from. It would probably be wise to use Python 2.7.x version in either 32 or 64 bit formats.

## 27.3 Perl

Perl scripts are used by several of the options to format intermediate output files for further processing. Instructions for downloading and installing these scripts can be found in the download and install section. Perl scripts are used in:

Allegro, SimWalk2-NPL  
If the user opts to create graphical plots for the LOD scores, then output from these programs are formatted into the *nplplot* input format using perl scripts Rallegro.pl, Rmerlin.pl and Rsimwalk2.pl.

Formatting Merlin’s output for use by SimWalk2 in the Merlin-SimWalk2 interface option is done by merlin2simwalk2.pl

Formatting output from the HWE and GEN programs for the Hardy-Weinberg testing is carried out by make\_hwe\_table.pl and make\_gen\_table.pl respectively.

### 27.3.1 Perl Installation

The web page that explains the Perl installation procedure and contains the binaries is at:

```
http://www.perl.org/get.html
```

## 27.4 Awk

The Unix text-formatting utility awk or its platform-specific implementations gawk (GNU awk) and nawk (new awk on solaris machines) is used by the script produced by the Genehunter option. Currently, the compilation platform decides which awk utility to use: gawk is used on the Linux platform, nawk is used on Solaris, and awk is used by Darwin (Mac OSX).

### 27.4.1 Awk Installation

The web page that explains the Gawk installation procedure and contains the binaries is at:

```
http://gnuwin32.sourceforge.net/packages/gawk.htm
```

## 27.5 C-shell

C-shell scripts are produced for the many of the analysis options. These C-shell scripts will carry out a default analysis with the target analysis program, and, in some cases, will also create plots of the results and generate custom track files for plotting within the UCSC browser.

# 28 Changes made to Mega2

## 28.1 Recent releases

|  |  |  |  |
| --- | --- | --- | --- |
| **Type of release** | **Version** | **Release date** | **Valid until** |
| Patched version | 4.7.1 | Oct 17, 2014 | June 15, 2015 |
| Patched version | 4.7.0 | May 15, 2014 | June 15, 2015 |
| Patched version | 4.6.2 | Feb 28, 2014 | June 15, 2014 |
| Patched version | 4.6.1 | Oct 21, 2013 | June 15, 2014 |
| Patched version | 4.6.0 | Sep 6, 2013 | June 15, 2014 |
| Patched version | 4.5.9 | July 5, 2013 | June 15, 2014 |
| Patched version | 4.5.8 | June 6, 2013 | June 15, 2014 |
| Patched version | 4.5.7 | January 11, 2013 | June 15, 2013 |
| Patched version | 4.5.6 | July 6, 2012 | June 15, 2013 |
| Patched version | 4.5.5 | June 15, 2012 | June 15, 2013 |
| Patched version | 4.5.4 | July 26, 2011 | June 15, 2012 |
| Patched version | 4.5.3 | June 15, 2011 |  |
| Patched version: | 4.0 R5.1 | June 15, 2010 |  |
| Patched version: | 4.0 R5.0 | December 31, 2009 |  |
| Patched version: | 4.0 R4.0 | October 4, 2009 |  |
| Patched version: | 4.0 R3.1 | July 12, 2009 |  |
| Patched version: | 4.0 R3 | June 15, 2009 |  |
| Patched version: | 4.0 R2 | April 15, 2009 |  |
| Patched version: | 4.0 R1 | June 13, 2008 |  |
| Release version: | 4.0 | March 31, 2008 |  |

## 28.2 Changes from Version 4.6.2 to Version 4.7.1 (Released Oct 16, 2014)

- Mega2 now gives you complete control to specify missing values for quantitative and affection phenotypes, both in the input data and for the data that Mega2 will output.
- Mega2’s performance is almost twice as fast as Version 4.7.0
- Bug Fix: Allow 0 markers or 0 traits w/o generating voluminous warnings.
- Bug Fix: Omit file now (again) allows 0 for person to represent ALL — the entire pedigree.
- Bug Fix: Detect missing suboption when it is required.

## 28.3 Changes from Version 4.6.2 to Version 4.7.0 (Released May 15, 2014)

- Mega2 now passes non-numeric alleles to analysis programs that will accept this format (section 15↑). Conversion to numeric alleles can still be requested if desired (section 22.4↑).
- Bug Fix: A number of problems with Beagle Analysis have been addressed.
- Bug Fix: Resolved an issue in specifying PLINK mode interactively.
- Bug Fix: Problems discovered via the CLANG/LLVM compiler have been addressed.

## 28.4 Changes from Version 4.6.1 to Version 4.6.2 (Released Feb 28, 2014)

- Mega2 can now read VCF files (See section 9.4↑).
- Initial interactive interface screens have been modified to improve the specification of different input file types (See section 12↑).
- Bug Fix: Binary file support (mainly .bed files) was broken on Windows and Mingw platforms.

## 28.5 Changes from Version 4.6.0 to Version 4.6.1 (Released Oct 21st, 2013)

- Enhancement: Mega2 can now produce PLINK/SEQ format files and a script for loading these these files into a PLINK/SEQ project (See section 25.40↑).
- Bug fix: Mega2 4.6.0 had broken support for Linkage format input when using a “Names file” (section 9.3.7↑).
- Enhancement: Added binaries for native Microsoft Windows 8 and MinGW built on Microsoft Windows 8.

## 28.6 Changes from Version 4.5.9 to Version 4.6.0 (Released Sept 6th, 2013)

- Enhancement: Mega2 compresses allele data to handle much larger datasets.

## 28.7 Changes from Version 4.5.8 to Version 4.5.9 (Released July 5th, 2013)

- Bug fix: Mega2 will now compile properly on Red Hat (RHEL), CentOS, and Solaris systems.

## 28.8 Changes from Version 4.5.7 to Version 4.5.8 (Released June 6th, 2013)

- Enhancement: Mega2 can now convert to PANGAEA MORGAN format (See section 13.36↑)
- Enhancement: Mega2 can now convert to Beagle format (See section 13.37↑)
- Enhancement: Mega2 can now convert to Eigenstrat format (See section 13.38↑)
- Enhancement: Mega2 can now convert to Structure format (See section 13.39↑)
- Bug fix: Liability classes now work according to the manual.
- Bug fix: You can now specific a value to represent the missing value code for a quantitative trait.

## 28.9 Changes from Version 4.5.6 to Version 4.5.7 (Released January 11th, 2013)

- Enhancement: Mega2 PLINK Analysis can now generate binary format PLINK PED files.
- Enhancement: Mega2 can now generate files for FBAT analysis.
- Enhancement: Mega2 can now support non-human species.
- Enhancement: Mega2 allows a value to be specified for output to replace a missing quantitative value.
- Enhancement: Some of the internals of Mega2 have been rewritten to use classes/objects.
- Enhancement: Mega2 code files are now c++ files and require a c++ compiler.
- Bug-fix: Improve Mac Xcode support.
- Bug-fix: The program to generate html from the text files (mega2log2html.pl) was not finding files.
- Bug-fix: Allow blank lines and comment lines in any of the files of the Mega2 format.
- Bug-fix: Allow menus to input and output X, Y, XY, MT and U as chromosomes without referencing explicit numeric values.

## 28.10 Changes from Version 4.5.5 to Version 4.5.6 (Released July 6th, 2012)

- Bug-fix: PLINK Ped file input in non-batch mode was broken.
- Bug-fix: Fix Mingw compilation issues.
- Bug-fix: Generate better error diagnostics.

## 28.11 Changes from Version 4.5.4 to Version 4.5.5 (Released June 15th, 2012)

- Enhancement: Mega2 now accepts PLINK binary PED input files.
- Enhancement: Mega2 now accepts PLINK PED input files.
- Enhancement: Mega2 now handles multiple genetic and/or physical maps in the MAP file.
- Enhancement: Mega2 now generates PLINK output files which may contain both a genetic and physical map.
- Enhancement: Selecting maps and outputting maps for analysis is more general and robust.
- Enhancement: Mega2 now uses much less storage to represent alleles and no longer copies allele sets.
- Bug-fix: Frequency calculations were incorrect for microsatellite alleles with more than 9 allele values.
- Bug-fix: Negative genetic distances between out of order markers are now replaced with the absolute value instead of 0.
- Bug-fix: The \*.keys mapping file has been reformatted. And IDs have been made consistent between linkage input and Mega2 input.
- Bug-fix: In general, code in critical loops or using large memory allocations has been recoded for efficiency.

## 28.12 Changes from Version 4.5.3 to Version 4.5.4 (Released July 26th, 2011)

- Bug-fix: Attempting to convert to SimWalk2 - Mistyping format with an input file that contained no trait loci resulted in a segmentation fault.
- Bug-fix: Attempting to convert to Mendel output (Option 29) resulted in a segmentation fault.
- Bug-fix: The install.sh installation script incorrectly failed to install a binary version when available; it always compiled from source.
- Bug-fix: The mega2log2html.pl Perl program in some cases tried to read from a closed file handle.

## 28.13 Changes from Version 4.0 R5.2 Beta - >  Version 4.5.3 (Released June 15th, 2011)

- Enhancement: Mega2 now supports conversion to IQLS/Idcoefs format.
- Enhancement: Changes were made towards making Mega2 64-bit compatible.
- Enhancement: The memory allocation routines were enhanced to better report out-of-memory errors.
- Enhancement: Removed the Python code from the Mega2 source code, replacing it with C++ code instead. This should avoid installation problems that some users were encountering in the Cygwin environment because their Python libraries/headers were out of sync with the ones used to compile Mega2.
- Bug-fix: The use of unique Ped\_Per IDs like 1\_21 was improperly turned on in PAP, so the resulting trip.dat was no longer in proper PAP format.
- Bug-fix: When doing quantitative summaries output to a sub-directory, the phenotyping table got written into the subdirectory, but the header got written into the root directory.
- Bug-fix: When converting Mega2 files to Merlin format, the MEGA2.ERR file had two strange extraneous lines at the end.
- Bug-fix: When converting to Merlin while zeroing out half-typed people, the “Half-typed genotypes” in MEGA2.RESET was incomplete.
- Bug-fix: When default batch file options are set to ’yes’, Mega2 no longer stops at the ’SPLINK option selection’ menu when in batch mode.
- Bug-fix: Under certain conditions, an array storing trait loci indicies was too short in the locus reordering function ReOrderLociByPositionNumber.
- Bug-fix: When only markers were selected, the SUP routine read off the end of the line and created incorrect files. SUP requires at least one trait locus, so this restriction has been implemented.
- Bug-fix: Conversion to SLINK format did not work correctly if the trait locus was not at the beginning of the locus list.
- Bug-fix: Corrected behavior of the ’Disease locus selection menu’ for the SLINK option. It was giving the wrong prompt and not correctly indicating the currently selected option.
- Bug-fix: Under certain conditions, one of the theta values was being written into the wrong position of the ’theta’ array when converting to SLINK format.
- Bug-fix: When setting up for a Quantitative Trait Summary in batch mode, the loci were reordered twice when they only needed to be reordered once.
- Bug-fix: The create\_nuked\_fids() function, when trying to rename the pedigree ID, wrote a string into itself, generating a ’source and destination overlap in memcpy’ error.
- Bug-fix: Failed to detect when the input batch file contained a trait number out of bounds. Added this check in.
- Bug-fix: In copy\_annotated\_to\_premake(), there was an error in the logic which led to reading a value from beyond the end of the persons[] array.
- Bug-fix: There was a failure to initialize the missing\_quant value correctly when none of the trait loci were quantitative and only the covariates were quantitative.
- Bug-fix: When manually-reordering using the GeneHunter option, the code contained an error where it tried to access the ’-1’ element of the global\_trait\_entries[] array.
- Bug-fix: check\_map\_positions() was not printing error messages properly.
- Bug-fix: The ’Locus Reordering Menu’ in the Quantitative Summary option was not working correctly.
- Bug-fix: When reading an annotated pedigree file that contains unmapped markers (chromosome ’U’), the cumulative counts of markers per chromosomes in the chromo\_loci\_counts array was missing an entry for the ’unknown’ chromosome.
- Bug-fix: The ’Select by locus number’ option of the ’Locus Reordering Menu’ was not working correctly when Mega2 was run interactively (It worked correctly when Mega2 was run in batch mode).
- Bug-fix: When attempting to break two loops in a pedigree, Mega2 incorrectly assigned proband/loop IDs 1 2 2 2, but this should have been (2,2) (3,3) or (1,2) (3,3). The person assigned proband ID 1 on input ended up being a member of the second loop. Since the user can avoid such an error by changing their choice of proband or by changing their loop breaker selection criteria, a partial solution was implemented so that Mega2 exits whenever it is trying to assign a loop breaker who is the proband to a loop other than loop number 2.
- Bug-fix: When the user chose to put the output files in a new output folder, the resulting MEGA2run.html did not work properly because the time-dated summary folder was not being copied into the new output folder.

## Changes from Ver 4.0 R5.1 - >  Ver 4.0 R5.2 Beta

- Bug-fix: Merlin plots contained an extra straight line when the steps option is selected.
- Bug-fix: For X-linked and Y-linked data, homozygous genotypes were mistakenly flagged as being heterozygous and vice versa, and always labeled as X-chromosome errors.
- Bug-fix: The allele-frequency summary option gave wrong counts and frequencies on Y-linked markers.

## Changes from Ver 4.0 R5.0 - >  Ver 4.0 R5.1 (Released June 15th, 2010)

- Bug-fix: The l2a.py converter script now checks the input pedigree file to make sure that each line contains the required number of headers, since otherwise the converted output file may be invalid. It terminates if there are too few, continues with a warning, if too many. See details on l2a.py for examples.
- Enhancement: Merlin option now includes creation of PDF plots using *nplplot* for the parametric option.
- Enhancement: Mega2 now optionally creates a Merlin model file for use by the parametric analysis option *--model* . This contains trait penetrances and frequencies specified in the input data (or default values if data was recoded).
- Enhancement: Mega2 now recognized markers on the mitochondrial chromosomes. The chromosome label should be 26 in non-annotated format file and MT in annotated format. For more details, see the MT chromosome section on how Mega2 recognizes and handles genotypes on this chromosome.
- Enhancement: Mendel’s Hardy-Weinberg testing option now allows bi-allelic markers.
- Bug-fix: The allele summary option did not include half-typed individuals’ genotypes in the counts, even if their genotypes are passed through to the output.
- Bug-fix: Mega2 did not allow setting unknown phenotype value for covariates.
- Bug-fix: The linkage option was missing the Ped: value field at the end, if the input pedigree file did not contain a Ped: field.

## Changes from Ver 4.0 R4.0 - >  Ver 4.0 R5.0 (Released Dec 31st, 2009)

- Bug-fix: Mendel8 format control file contained multiple affection definitions if the user opted for combined output of multiple affection traits, causing Mendel to fail. Now, only the first affection trait is listed in the control file.
- Bug-fix: Mega2 aborted right after starting up on Mac OS X Snow Leopard machines. This has been fixed.
- Enhancement: Mega2’s support for Merlin has been improved: the Merlin non-parametric LOD score script now turns on the --tabulate option and stores the resulting tables in appropriately named files.
- Enhancement: Handling of YLINK markers has been improved.
- Enhancement: This version uses Python dictionaries to speed up reading in of the input files. You may notice a marked improvement on large data sets (100K markers or more).

## Changes from Ver 4.0 R3.1 - >  Ver 4.0 R4.0 (Released October 4th, 2009)

- Enhancement: New supported option CRANEFOOT: Mega2 now creates output files in CRANEFOOT format. CRANEFOOT is a publicly available program to draw pedigrees.
- Enhancement: New option to output Mega2 annotated format files. This option can be used to create annotated format files starting with linkage format files.
- Bug-fix: Mendel7+ and X-linked markers: if the input map file contains only sex-averaged map positions, the Mendel map file positions would be set to 0 for annotated format input file. This has been fixed; average positions are taken to be the female positions, and male positions are set to 0.

## Changes from Ver 4.0 R3 - >  Ver 4.0 R3.1 (Released July 12, 2009)

- Bug-fix: Mendel 9.0 did not accept, nor use any special missing quantitative phenotype value when analyzing quantitative traits, instead, it used the default value of 0.0.
- Bug-fix: Linkage format options (LINKAGE, SLINK, SPLINK, SIMULATE, VITESSE etc.) erroneously set the first paternal and maternal sibling fields to 0, even when such siblings were present inside the pedigree.

## Changes from Ver 4.0 R2 - >  Ver 4.0 R3 (Released Jun 15, 2009)

- Enhancement:The nplplot package has been re-vamped and extended to include the generation of formatted custom-track files for viewing within the UCSC genome browser.
- Enhancement:The nplplot() function itself has changed; for more detail please consult the documentation.
- Modification: We have added the “strsep” function as part of our source code, to be conditionally compiled for solaris platforms (use the flag -DSOLARIS in your Makedefs\_solaris file to include our strsep). This was necessary since certain Solaris installations are missing this particular function from their standard libraries.

## Changes from Ver 4.0 R1 - >  Ver 4.0 R2 (Released April 15, 2009)

**Version 4.0 R2 has several improvements and bug-fixes, so the list has been divided up into these sections.**

- PLINK bugs and improvements
- Mendel bugs and improvements
- Annotated file bugs and improvements
- Handling X,Y,XY, and unmapped loci  
  For more details, consult this page on handling special chromosomes.
- Menu related changes
- Other bug-fixes and modifications

The bug fixes listed on this page are also separately listed in the section Updated bug list .

**PLINK bugs and improvements**

- Bug-fix: PLINK long format output files were not named correctly, as per PLINK specifications.
  - Bug-fix: PLINK shell script had problems and failed to run. This has been fixed.
  - Bug-fix: Affection status inside the fam file was set to 0 for everyone.
  - Enhancement: PLINK map files are now created with genetic distances, by using the --cm analysis option.
  - Enhancement: Allows the creation of output files that are separated by chromosomes or combined into one set of files.

  **Mendel bugs and improvements**

  - Modification: Old format Mendel files are no longer supported within the new Mendel option (option 29). The old Mendel option (option 2) still continues to produce old Mendel format files.
  - Bug-fix: Newest version of Mendel does not accept the variable file, which lists quantitative variables, instead quantitative loci are now added to the definition file (which is named mendel\_locus.\* by default).
  - Modification: Since Mendel expects quantitative variables to be listed at the end of the definition file, the user-specified locus order may not be honored.
  - Modification: There was some confusion regarding whether Mendel map file should include traits, and these were excluded in the last revision. However, it seems that trait loci also need to be in the map file, so they have been re-introduced into the map file.
  - Enhancement: Mendel’s newest version now supports longer locus names, up-to 16 characters long.

  **Annotated file bugs and improvements**

  Enhancement: Mega2 does not require all loci from the names file to be inside allele-frequency and penetrance files. However, if you do enter a locus in one of these files, it has to be defined inside the names file, and it needs to be completely specified, i.e., with the required number of frequencies and penetrances.   
  If some loci are missing from the frequency file, Mega2 will estimate their allele frequencies from the data.

  - Modification:Missing value indicators to the conversion script l2a.py no longer need to be enclosed within “”s.
  - Bug-fix: Mega2 crashed if frequency file was not specified. This has been fixed.
  - Enhancement: The l2a.py conversion script now allows the missing quantitative missing value to be specified as a number if no quotes are used, or a string, by enclosing your values within quotes. Affection and quantitative missing values are interpreted as strings.
  - Enhancement: NAs are now acceptable as unknown value indicator for affection status loci as well.

  **Handling X,Y,XY, and unmapped loci**

  - Enhancement: Mega2 now allows inclusion of marker loci with unknown positions for a limited number of options that do not require a map. Unmapped loci can be specified using a special label inside the map file, and are output in order of their appearance inside the names file.
  - Enhancement: Mega2 now implements special handling of Y-linked markers. You can create output files for most options using Y-linked markers, but note that very few analysis programs do linkage analysis on these.

  **Menu related changes**

  - Bug-fix: The menu option for changing the unknown status and allele designator in the first menu was not working.
  - Enhancement: Mega2 now allows the user to specify affection labels (lists of status-class pairs) separately for each affection trait that has multiple liability classes. Previously, only a single set of labels was allowed over all traits.
  - Enhancement: Analysis options and sub-options can now be denoted by their names inside the batch file instead of numbers. For a list of these names, please consult the documentation.

  **Other bug-fixes and modifications**

  - Modification: Simwalk2 option output files for haplotype, parametric, non-parametric, mistyping-estimation, and IBD estimation are now named sw2\_pedigree, sw2\_locus, sw2\_map etc.
  - Bug-fix: Conversion from pre-makeped format to post-makeped format output options (such as SLINK, Vitesse etc.) created wrong first-sibling links to be created for pedigrees that had half-siblings. This has been fixed.
  - Bug-fix: Allegro, Gene Hunter, Gene Hunter-Plus and MLB-QTL output locus files had default trait penetrances set to 0.0 rather than the usual 0.5.
  - Enhancement: Mega2 automatically limits the display of warnings and error messages during the error-checking steps, as these can run into large numbers.

**Version 4.0 beta, 4.0 beta R1, 4.0** introduces a new input file format called the annotated format. observed under somewhat rare circumstances. Version 12 also implements a new output option for the simulation program SUP. Please check the SUP option details in section VI .

## Changes from Ver 4.0 - >  Ver 4.0 R1 (Released Jun 13, 2008)

- Enhancement: User-defined missing codes for affection status and marker alleles in menu1. Default value is NA.
- Bug-fix: Output file names were garbled when output folder name had a “.” inside it. This has been fixed.
- Bug-fix: SLINK crashed when multiple chromosomes were selected. This has been fixed.
- Bug-fix: Rsimwalk2.pl did not read in SimWalk2 scores correctly.
- Enhancement: The linkage2annotated.py is now named l2a.py. Also removed the requirement that input missing codes need to be enclosed inside “”.
- Enhancement: Mega2 now compares the version number on watson web-site against itself, to figure out if it needs to be updated. This can be turned off using the -noweb option.
- Modification: Trait loci are no longer included inside the Mendel CSV map file.
- Bug-fix: Quantitative summary gave wrong standard deviation values. This has been fixed.
- Bug-fix: Random simulation of genotyping errors did not work correctly from 4.0 beta onwards. This has been fixed.

## Changes from Ver 4.0 Beta R1 - >  Ver 4.0 (Released March 31, 2008)

- Bug-fix: Annotated files caused Mega2 to crash if a frequency file was not provided. This has been fixed, and Mega2 will now calculate observed allele frequencies from the pedigree data as usual.
- Bug-fix: Merlin format output files were missing covariates, if these were included in the selected traits.
- Bug-fix: For these options, Mega2 falsely detected Mendelian inheritance errors for male X-linked genotypes: PREST, HWE (all options), SUMMARIES (all options).
- Bug-fix: Several bugs in handling of X-linked data have been fixed for options that allow X-linked analysis on data that contains both autosomal and X-linked loci. These include setting special X-linked analysis flags for the X-chromosome output.
- Enhancement: Annotated format now allows male, female, and sex-averaged penetrances inside the penetrance file to be defined for each binary trait locus.
- Enhancement: SUP option now supports simulation of X-linked data.
- Enhancement: Trait selection menu now allows ranges inside input trait numbers e.g 1-10 etc. This makes it easier to handle data with a large number of phenotypes.
- Enhancement: In keeping with the above enhancement, the Trait\_Subdirs keyword now allows a special value “Use trait names”, which tells Mega2 to use the trait names are trait-specific output sub-directory names.
- Modification: The batch file keywords Traits\_Selected\_Num and Covariates\_Num are no longer required. If present, they will be ignored.
- Bug-fix: R plot menu behavior was incorrect.
- Enhancement: R plot now allows Merlin’s trait-combination mode. Also allows traits to be separated out even when Merlin output has these combined.
- Modification: The nplplot function now allows two extra arguments, lgndx, and lgndy to specify positions for legends inside the graph. The default values are NULL.
- Modification: The lgnd argument now takes three values, TRUE, FALSE, “page”, and a list of plot numbers. TRUE or FALSE causes legends to be plotted for all plots or none respectively. The “page” value causes a legend to be inserted into the first plot of every page. The plots numbers specify which plots to insert legends into. Default value is “page”.
- Enhancement: Using the new nplplot arguments, Mega2 now inserts a legend into the first plot for every trait if the user chooses to combine traits for several traits into the same file.
- Enhancement: A y-axis label has been added to the LOD score and P-value plots.

## Changes from Ver 4.0 Beta- >  Ver 4.0 Beta R1 (Released August 7, 2007)

- Bug-fix: The implementations for better memory utilization were not implemented correctly for the nuclear pedigree option, aspex option, and PAP option, which segfaulted. These options are working now.
- Bug-fix: Sage 3.0 sibpal parameter file was wrong
- Bug-fix: R plot menu behavior has been improved: wasn’t working for a single trait in LoopOverTrait mode.
- Bug-fix: Affected sib-pair summaries had wrong parental affection status. All parents were set to unknown. This has been corrected
- Bug-fix: Trait names in the Mendel formatted locus files (mendel\_locus, locus and LOCUS), are now shortened to 8 characters as for the 3.0 version and revisions.
- Modification: Merlin command line options were out-of-date. This revision of Mega2 has now been brought up-to-date.

**Version 3.0 Revision 10,11,12** fixes a few more bugs that were observed under somewhat rare circumstances. Version 12 also implements a new output option for the simulation program SUP. Please check the SUP option details in section VI .

## Changes from Ver 3.0 R11- >  Ver 3.0 R12 and Ver 4.0 Beta (Released June 14, 2007)

- Bug-fix: Nuclear pedigree files had redundant chromosome number extensions when multiple chromosomes were selected.
- Enhancement: There is new option to create files for the genotype simulation program SUP, which is based on SLINK, but can handle large numbers of loci unlike SLINK. For more details, consult the section on SUP .
- Modification: The APM, APM-Mult and TDTMAX options have been disabled, and will no longer be supported. The menu still contains these options, but with DISABLED labels.

## Changes from Ver 3.0 R10- >  Ver 3.0 R11 (Released May 17, 2007)

- Bug-fix: In some cases, large pedigrees in pre-makeped format files caused Mega2 to crash, if the number of individuals within a pedigree exceeded the number of pedigrees. For data with smaller pedigrees, the KEYS file contained wrong information, however, output files would not have been affected.

## Changes from Ver 3.0 R9- >  Ver 3.0 R10 (Released February 1, 2007)

- Bug-fix: Several options produced wrong pedigree file with missing pedigree IDs when using a pre-makeped pedigree file as input. These involve options which skip pre-makeped to post-makeped conversion including Mendel format options, Merlin, Prest, and Solar.
- Bug-fix: Reordering of traits contained a bug, where the trait locus was set to the wrong locus, if user changed the ordering from the input order.
- Bug-fix: Old SAGE format files wrote spaces for missing trait values instead of a missing value.
- Bug-fix: HLOD scores were not read in from Allegro’s output for plotting. These scores are produced by Allegro’s parametric linkage analysis.
- Bug-fix: HWE files for Mendel’s Hardy-Weinberg estimation contained completely ungenotyped individuals.
- Bug-fix: Loop-breaking in premakeped format pedigrees produced false error messages about failure to break loops for pedigrees that did not contain loops.
- Enhancement: The R-plotting now handles LOD scores produced by Merlin’s --grid option in non-parametric linkage analysis, where marker names are not output.
- Enhancement: The R-plotting process has been made more robust to errors in the plot data produced by Allegro, Merlin and SimWalk2. If these analyses failed to run and produced no LOD scores, then Mega2 would produce empty plot files. These errors are detected now, and the user informed accordingly. Plot files are not produced.
- Modification: For data containing multiple plots, such as genome-scan data, the user can now selectively ignore y-axis bounds set in the menu for plots containing data that exceed these bounds. Previously, bounds would be used for all or none of the plots.

## Very old versions

**Version 3.0 Revision 8, 9** are patched versions of Mega2 3.0. They contains some important bug-fixes that was introduced in the 3.0 R7 version.   
  
**Version 3.0 Revision 7** is a patched version of Mega2 3.0. It contains a couple of bug-fixes, and the expiry date has been changed to June 15, 2007.   
  
**Version 3.0 Revisions 1, 2,3, 4 and 5** are patched versions of Mega2 3.0. They contain some important bug-fixes which are listed below.   
  
**Version 3.0** is the fifth major release of Mega2. This version contains some important changes in Mega2’s behavior. Please consult the documentation for a detailed explanation of these changes.  
  
**Version 2.5** is the fourth major release of Mega2. This version contains many bug-fixes and new features. The user should consult this documentation for new features and bug-fixes prior to using version 2.5.   
  
**Version 2.3** is the third major release of Mega2. This version and its revisions will be no longer supported, and their licenses expire on June 15, 2003.   
  
**Version 2.3** had three revisions **R2** , **R3** , and **R4** containing several bug-fixes. These bugs and the options they might affect are listed below.

## Changes from Ver 3.0 R8- >  Ver 3.0 R9 (Released July 14, 2006)

- Bug-fix: The old Mendel format marker file was incorrectly read in by the RELPAIR conversion Perl script because there was no space between the chromosome number and marker position.
- Bug-fix: The Rallegro.pl, Rmerlin.pl and Rsimwalk2.pl Perl scripts failed to distinguish between marker loci that had identical map positions on a chromosome (very dense markers would have the same problem, if the output did not have enough precision), producing incorrectly formatted plot-output files.

## Changes from Ver 3.0 R7- >  Ver 3.0 R8 (Released June 19, 2006)

- Bug-fix: Premakeped format pedigrees with affection status loci caused Mega2 to crash. This has been fixed in 3.0R8.
- Bug-fix: For pre-makeped format files, output files did not contain pedigree numbers, if user selected premakeped pedigree IDs as the output pedigree id.
- Bug-fix: Mega2 failed to reset Mendelianly inconsistent genotypes even if the menu item for this action in the “Incorrect genotypes reset menu” was set to yes.

## Changes from Ver 3.0 R5,R6 - >  Ver 3.0 R7 (Released June 15, 2006)

- Modification:In the omit file, keyword “All” in the marker column reset trait phenotypes as well as marker genotypes. This has been changed so that only marker genotypes are set to unknown.
- Bug-fix:Unconnected individuals in premakeped format pedigrees went undetected, if the pedigrees consisted only of disconnected individuals. This has been fixed.
- Bug-fix:Mendel option did not label X-linked loci as such when the combined output option was selected.

## Changes from Ver 3.0 R4 - >  Ver 3.0 R5 (Released Feb 2, 2006)

- Bug-fix: Merlin format pedigree files created by Mega2 were not read in correctly by Merlin if affection status traits included a liability column, since there is no way to specify such loci using the QTDT format names file. Now, when Mega2 is converting to Merlin format and it encounters an affection status trait with a liability column, the user is prompted to select status-class labels to be designated as affected, and only the status is output in the pedigree file.
- Bug-fix: When a names file is used so that alleles are recoded, the random selection of individuals was not working correctly. Hence, allele frequencies computed via options 2 and 3 of the individual-selection menu may have been incorrect under certain conditions. Option 2 selects typed founders or a random member if a pedigree has no typed founders, and option 3 includes individuals selected via option 2 as well as one non-founder per pedigree with an allele not found by option 2. For more details, see the section on recode bug.
- Enhancement: When a names file is used so that alleles are recoded, Mega2 now computes and reports allele-frequencies in four different ways (see Recode section for details) and reports a table of these different allele frequency estimates in the MEGA2.RECODE summary file. Subsequent output, however, uses only frequencies computed from the subset (founders, founders+random etc.) specified by the user.
- Modification: The omit file is read in prior to recoding now. Also, untyped or incompletely-typed pedigrees are omitted as specified via menu 1 prior to recoding and allele-frequency computation.
- Enhancement: The individual selection menu now has an option to include half-typed individuals (where only one allele is known and the other is unknown) in the recoding process. For the subsequent output analysis options, half-typed individuals are included or excluded from the output files depending on the user’s choices in the Invalid genotypes exclusion menu.
- Modification: The Rallegro.pl Perl script has changed in keeping with the new Allegro output in which the absolute value of the NPL LOD score and the delta are reported in two separate columns. Results from older versions of Allegro used a single column with the actual value preceded by the sign. Users are advised not to use the new script to read Allegro output produced by Allegro versions older than 1.2c. For the purposes of graphing Allegro results, we follow the convention of Merlin and multiply the NPL LOD score by the sign if the delta before plotting it.
- Enhancement: Total squared deviation between observed and input allele frequencies Mega2 scans the observed genotype data and computes observed allele frequencies over full-typed as well as half-typed individuals to see if these match input frequencies within a certain tolerance. Exceeding this threshold is a non-fatal error, i.e., mega2 will stop and warn the user, with the option to continue. The tolerance value is decided by item 10 of the input menu. Tolerance can be set to a large value (e.g. set threshold to N or greater, if where N is the maximum number of alleles) to avoid warnings.
- Bug-fix: When recoding took place, Option 3 of of individual selection menu did not count all alleles present in data - it still gave 0 allele counts.
- Enhancement: In the recoding step, Option 3 has been changed now to include other individuals only after it goes through the selection process specified by option 2. Then it looks at alleles with 0 counts, and selects the first person from each pedigree with that allele, counting both alleles of that person.
- Bug-fix: Incorrect displaying of omitted pedigree and person numbers has been fixed.
- Enhancement: The default output format for NPL LOD score plots (various options) is now PDF instead of post-script. The nplplot() function decides this format based on the output file name’s extension, [.ps or .pdf].
- Enhancement: If the data contain both X-linked and autosomal markers, the user can choose whether to (a) treat all markers as sex-linked, (b) all markers as autosomal, or (c) only markers on chromosome 23 as sex-linked and the rest as autosomal.
- Enhancement: When creating plots with nplplot, yaxis bounds can now be enforced strictly by setting a flag in the plot-parameters menu.
- Enhancement: Merlin LOD scores can now be graphed when either the --grid or the --steps options is used. The --markerNames option is included by default when executing Merlin within the Merlin C-shell script.
- Bug-fix: The Allegro script for running and plotting results of analysis on multiple traits did not work. This has been fixed.
- Bug-fix: The R shell script contained mal-formed file-names when a single trait was selected for Merlin and SimWalk2 such as (null)/merlin.01.out. This has been fixed.

## Changes from Ver 3.0 R3 - >  Ver 3.0 R4 (Released June 10, 2005)

- Bug-fix: In recoding alleles, wrong frequencies were being assigned to the alleles if and only if the ORIGINAL alleles were numbered 1 to N (N  > = 10), making renaming unnecessary.
- Bug-fix: Due to floating-point precision error, the missing quantitative phenotype value was not read in correctly. The precision has been increased now.
- Bug-fix: When recoding alleles, total allele count was wrong in MEGA2.RECODE file.
- Important change: The usage of Rmerlin.pl script has been changed: \*Names\* of statistics are used instead of numbers, therefore older Mega2 generated Rmerlin.\*.sh scripts will no longer work with the new Rmerlin.pl Perl script. See the R-graphics section for details on how to regenerate plots on older analysis results.
- Enhancement: R-plots can be generated when Merlin’s --vc — --(variance components) is selected for analysis, see R-plots section for details.
- Enhancement: Output R-plots can be saved in either postscript or pdf format, see R-plots section for details.
- Enhancement: In option 2 of locus-reordering menu, we can select numbers during the display. Consult the locus reordering section for details.
- Enhancement: Added selection of covariates to the Merlin analysis option, premakeped format option and SOLAR.
- Enhancement: Mega2 checks entire pedigree file for pedigree errors before terminating Mega2 (instead of at the first error encountered).
- Enhancement: Mega2 checks entire map file for errors before terminating due to fatal errors. The reading is is also more robust to invalid values in the columns, (e.g. non-numeric strings in chromosome or position columns).

## Changes from Ver 3.0 R2 - >  Ver 3.0 R3 (Released November 29, 2004)

- Bug-fix:The R options for Hardy-Weinberg equilibrium testing were generating spurious error messages on skipping markers. This has been fixed.
- Bug-fix: Wrong column names were being written in the output table created by the HWE R exact option. This has been corrected.
- Bug-fix:Unique IDs were not carried over during conversion from Pre-makeped format to post-makeped format.

## Changes from Ver 3.0 R1 - >  Ver 3.0 R2 (Released September 30, 2004)

- Bug-fix:The ID: field not read in when multiple chromosomes were selected for analysis.
- Bug-fix: HWE-R EXACT option: R-script was wrong, this has been fixed.
- Bug-fix: Mendel5 pedigree files contained quantitative variable for affection loci at the end of each record, which does not work with the new version of Mendel.
- Bug-fix: Mendel5 requires a variable file for defining QTLs. This has been added to the list of output files created for Mendel.
- Bug-fix: Selection of individuals for allele-frequency estimation using the founders + all unique alleles did not work. It still produced zero allele frequencies which should not be the case.
- Bug-fix: HWE R options: Spurious error message even when table was created correctly.
- Bug-fix: Linkage option - Only pedigrees with non-numeric names assigned numbers causing possible conflicts with pedigrees whose original numeric IDs were maintained in the output.
- Bug-fix: Merlin gave a segmentation-fault, if the user selected only one statistic for graphical output.
- Bug-fix: Vitesse shell script was missing its header and hence could not be run.
- Enhancement: New keyword Count\_Genotypes to indicate which option of the select-individual menus to use.

## Changes from Ver 3.0 - >  Ver 3.0 R1 (Released August 15, 2004)

- Bug-fix: Hardy-Weinberg - HWE option (Guo and Thompson) had a bug, it either crashed or produced wrong genotype counts.
- Bug-fix: SimWalk2-npl option crashed while setting R-plots
- Bug-fix: Merlin’s R-plot shell script had the wrong file names and didn’t work for a single trait.
- Enhancement: Saving run-specific files can be turned off by invoking:  >  >  mega2 --nosave [optional batch\_file\_name]
- Important change: Merlin output locus file format is now QTDT format instead of linkage format. See section on Merlin option for details.
- Bug-fix: In rare cases, Mega2 crashed while breaking loops.
- Bug-fix: In the Combine-traits mode, Mega2 would not allow selection of all the markers and traits.
- Bug-fix: HWE-R based options produced incorrect MEGA2.KEYS file.
- Bug-fix: Corrected handling of missing R formatted data files for R-graphics - Allegro continued through the reading even when files were missing
- Bug-fix: HWE-Mendel file names menu had two options numbered 3.
- Enhancement: Mega2 now stores a copy of the batch file along with the run-specific files.

## Changes from Ver 2.5 R4 - >  Ver 3.0 (Released June 15, 2004)

- Bug-Fix: SAGE4 parameter file was wrong. The field-widths were not set correctly.
- Enhancement: Mega2 now allows to the user to specify an alternate location for the output files, the default is the current directory.
- Enhancement: An html format page is created with links to input files, run summary files and output files, for easy access.
- Important Change: A new Perl script is now included with the others, mega2log2html.pl for the creation of the html page.
- Enhancement: New Hardy-Weinberg options, two from the R “genetics” library, and Mendel version 5.0.
- Enhancement: We preserve run summaries for each run, by creating a unique directory identified by the run date and time, and storing the summaries within this directory.
- Bug-Fix: X-linked analysis was not working, caused Mega2 to crash.
- Bug-Fix: Alleles are not recoded if they are already numbered from 1 through N (N= number of alleles), and all N alleles are present in the data.
- Enhancement: New selection choice for counting genotypes for recoding alleles with the names file option.
- Enhancement: SOLAR option now also generates a TCL script that provides a function within the SOLAR environment to load in the pedigree, locus, map and phenotype files.
- Important Change: The trait-selection menu has changed. It DOES NOT include the markers by default in the “Combine” mode. See the trait menu section for more details.
- Important Change: Batch file keywords have changed: Markers\_Selected is now Loci\_Selected. Markers\_Selected\_Num is no longer valid. MEGA2 will produce a warning message and ignore this keyword.
- Enhancement: Untyped persons are not included in the per-person typing rate table in genotyping-rate summary. Instead the persons are listed at the end of the summary file.
- Bug-Fix: Since bash is not available on DEC-Alpha, the install script now uses “sh”.
- Bug-Fix: In the BATCH mode, Mega2 appended new declarations for Value\_MissingQuant to any existing MEGA2.BATCH file. This has been fixed.
- Bug-Fix: SAGE 4.0 parameter file now includes a default 0 as missing genotype value to prevent error messages in the GENIBD output about unrecognized genotype value.
- Bug-Fix: Mendel5 option created incorrect keys file.
- Bug-Fix: Duplicate names in the names files caused Mega2 to run incorrectly. These are detected and causes Mega2 to exit.

## Changes from Ver 2.5 R3 - >  Ver 2.5 R4 (Released April 22, 2004)

- Bug-Fix: SOLAR bug fixed: In the “loop-over traits” mode person IDs in the phenotype file did not match person IDs in the pedigree file.

## Changes from Ver 2.5 R2 - >  Ver 2.5 R3 (Released April 15, 2004)

- Important Change: Names file locus codes have been changed to match Merlin’s locus file format codes. Consult the section on Names file for details.
- Bug-Fix: SOLAR bug fixed: Mega2 crashed while looping over quantitative trait loci, one at a time.
- Bug-Fix: RECODE bug fixed: Mega2 crashed while recoding loci from *names file* , if there were no mapped loci.
- Bug-Fix: MERLIN bug fixed: Merlin analysis option menu was displayed twice.
- Bug-Fix: GENOTYPING ERROR simulation mode bug fixed: Locus selection was not working properly.
- Bug-Fix: SIMWALK2 bug fixed: Location scores option produced an incorrect penetrance file for multiple affection liability classes.
- Enhancement: Improved messages with respect to pedigree format (pre- or post-makeped) detection. Warns the user if there are 0s in the fifth column, and proceeds to read in post-makeped.

## Changes from Ver 2.5 R1 - >  Ver 2.5 R2 (Released August 8, 2003)

- Bug-Fix: ASPEX bug fixed - nuclear pedigree naming/numbering options did not allow numbering with extensions.
- Bug-Fix: SPLINK bug fixed - did not put all markers into one file, when user selected the global output file option from the file-selection menu.
- Bug-Fix: S.A.G.E. 4.x bug fixed - The format statement in the parameter file did not match pedigree records.
- Bug-Fix: R-graphics bug fixed - The output file was not renumbered according to the chromosome correctly.
- Enhancement: Combined shell-scripts - For SimWalk2, Merlin and Allegro options, a combined shell script is generated if there are multiple traits to loop over. This runs the trait-specific shell-scripts, then the R-graphics script if appropriate. See details on SimWalk2 for an example.
- Enhancement: R-graphics scripts are now more robust to missing files, incomplete analysis etc. Perl scripts now return an error if it doesn’t find the input file, and this error is trapped by the shell script. *nplplot()* now returns a “FALSE” value if the formatted data file is empty or has some problems. This is trapped by the R-script.
- Enhancement: Mega2 batch file is now more robust to errors such as empty value field, and value lists which do not contain enough items.

## Changes from Ver 2.5 - >  Ver 2.5 R1 (Released July 5th, 2003)

- Enhancement: Option selection for Merlin-only format - The user is prompted to enter a string consisting of Merlin analysis options. For details see the Merlin analysis section.
- Enhancement: Merlin graphics are set up only if either or both the --npl and --pairs options are selected, and the --markerNames option is not selected.
- Bug-Fix: Fixed S.A.G.E. 4 parameter file bug in the format statement.
- Bug-Fix: Fixed S.A.G.E. 3 counts parameter file bug in the format statement.
- Bug-Fix: Fixed PREST pedigree file bug, all lines were combined into one long line.
- Bug-Fix: Fixed Merlin/SW2-NPL pedigree naming mismatch. This option filed to work because the pedigree names in the Merlin NPL output did not match the names in the SimWalk2 pedigree file.
- Bug-Fix: Pedigree and individual id menu behavior has been fixed. The different options were not being selected and displayed correctly.
- Bug-Fix: Fixed SPLINK format bug - was writing individual IDs in place of mother’s IDs.
- Bug-Fix: Fixed bug in SimWalk2 for handling more than 100 liability classes.
- Bug-Fix: Fixed bug in the Win 32 version of Mega2, where the locus order line was not read in correctly. The 2.5 version will work correctly however, as long as there is an extra non-numeric character before the newline.
- Bug-Fix: Blank lines are allowed before the locus-order line again, the check for locus order in Ver 2.5 did not accept blank lines just before the locus order line.

## Changes from Ver 2.3 R4 - >  Ver 2.5 (Released June 3, 2003)

- Enhancement: New option for creating Loki format files.
- Enhancement: New Recode utility for converting a minimal locus data file with only locus types and names as input, then creating a linkage format locus file with allele frequencies computed from the pedigree data.
- Enhancement: New batch file options for greater automation   
  Default\_Outfile\_Names   
  Default\_Reset\_Invalid   
  Default\_Other\_Values   
  Default\_Ignore\_Nonfatal   
  Default\_Ignore\_Xlinked   
  See Batch file documentation for details.
- Enhancement: R-plot labels for each curve can now be input separately from a header file. Mega2 creates this header file by default, and instructs the nplplot() routine to skip the first line of the input plot data file.
- Bug-Fix: in SOLAR where the phenotype file and marker genotype file didn’t match the person IDs in the pedigree file (id choice was not implemented for the former).
- Bug-Fix: in locus reordering, for multiple chromosomes and multiple traits, traits were being output at the beginning of the order irrespective of the user’s ordering.
- Bug-Fix: in selection of loci for random genotype error detection from the batch file.
- Bug-Fix: in renaming chromosome number extension for output files.
- Bug-Fix: for segmentation fault for 0 recombination fractions (where there is only a single locus in premakeped format output files.
- Bug-Fix: existing vstrm files are removed before each Vitesse run.
- Enhancement: Flag too few locus numbers in locus order line of input linkage format locus file.
- Enhancement: Creating a marker frequency file for Merlin format.
- Enhancement: Improved user-interface for selecting pedigree and individual id for output files, these can be either selected from the input, or generated in some cases, e.g. unique IDs can be generated by mega2.
- Enhancement: Changed y/n user-prompt for per-chromosome files in various options to a numbered menu (with separate files as the default).
- Enhancement: Menu for selecting Allegro scores to plot now have more descriptive names.
- Enhancement: R-setup menu called only once for the SimWalk2-Merlin interface option.
- Enhancement: For Merlin, cannot handle generating plots for multiple traits if they are combined (each trait separately works).

## Changes from Ver 2.3 R3 - >  Ver 2.3 R4 (Released Feb 7, 2003)

- Bug-Fix: SimWalk2 penetrance file had invalid liability class values. Acceptable values are 1-99. Currently, if the input data has affection loci with 100 or more classes, Mega2 will produce a penetrance file that is not acceptable to SimWalk2. Efforts are underway to modify SimWalk2 to accept a larger number of liability classes.
- Bug-Fix: SPLINK option produced garbled individual IDs in the pedigree file if the user opted to combine all loci into one pedigree file. This has been rectified.

## Changes from Ver 2.3 R2 - >  Ver 2.3 R3 (Released Dec 13, 2002)

- Bug-Fix: For SOLAR, the HHID phenotype was being written into the phenotype file, this has been moved to the pedigree file.
- Enhancement: Number of allowable liability classes has been increased up to a million. Theoretically, there really is no limit, except for Mendel format output which has a field size limitation.
- Enhancement: New MEGA2.KEYS file now gives a mapping to pedigree and person IDs between the input and output.
- Bug-fix: Fixed major bug in loop reconnection - the older version assumed that the proband numbers were ordered in the pedigree file. Also, the renumbering of links (first\_off, sibs etc.) was off. For each loop-reconnection pair (delete\_person, keep\_person), we have to follow two steps:   
  1) Look at each occurrence of delete\_person and change it to the corresponding keep\_person.   
  2) Then look at the links fields of keep\_person, and make sure they match the delete\_person’s fields.
- New analysis option: Merlin 0.9.x format files merlin\_ped.01, merlin\_data.01, and merlin\_map.01.
- Changed the Merlin files for Simwalk2-Merlin option to sw2merlin\_ped.01, sw2merlin\_data.01 and sw2merlin\_map.01.
- Bug-fix: Fixed individual-count for pre-makeped format input pedigrees.
- Enhancement: Sex-specific map positions in the map file, This requires the two words, “male” and “female” in the header line of the map file AFTER the first three columns. The type of map (kosambi or not) is decided by the title of the sex-averaged distance column.
- Enhancement: Several options can now select which pedigree id to output (Ped: or linkage id) and which person id to output (Per:, linkage or ID:). If these fields (at least the Ped:, Per: and ID:) don’t exist in the input they are generated, and the user is informed of the fact. These options are Simwalk2, Mendel, Aspex, SAGE, SOLAR, Genotyping Summary, Pre-makeped, Merlin-SimWalk2, and Merlin. Refer to Using unique person IDs for details.
- Bug-fix: Selecting only trait loci in S.A.G.E. 4.0 resulted in a segfault. This has been fixed.
- Minor bug - Menu wording in SimWalk2 file names menu was a bit strange, so changed the wording.

## Changes from Ver 2.3 - >  Ver 2.3 R2 (Released July 20 2002)

- Bug-fix: Versions 2.3 and 2.3 R1 may have had a segmentation fault when creating nuclear pedigrees from post-makeped pedigrees which did not have person IDs numbered consecutively from 1 through the number of pedigree members. Nuclear pedigrees are also created in the S.A.G.E. 3.0, ASPEX, and GHMLB options. Pre-makeped pedigrees do not cause segmentation faults in either version 2.3.
- Bug-fix: Post-makeped pedigrees with non-ordinal entry IDs may have also created segmentation faults in the “create summary” option for segregation summary. Again pre-makeped files are not a problem.
- Bug-fix: Mendel pedigree file format statement has been fixed. The format statement for reading in pedigree members was incorrect.
- Enhancement: A totals line has been added to the affected sib count section of the sib\_sum.\* file.
- Enhancement: In addition to chromosome-specific genotyping success rates, an overall genotyping-success rate file is created (if analysis includes multiple chromosomes).
- Bug-fix: The script sage\_cnt.\*.sh was not working in case of a single affection trait. This script has been corrected.
- Bug-fix: In some cases, nuclear families included half-sibs This bug affects Aspex, Affected Sib Counts, GHMLB and S.A.G.E.

## Changes from Ver 2.2 R3 - >  Ver 2.3 (Released June 14 2002)

- Removed a bug from the loop-reconnection process. Mega2 failed to reconnect loops if proband number 1 did not appear before all other probands in the post-makeped format pedigree file.
- A mistyping simulation option can be activated via the first menu, so that random genotyping errors according to an error probability distribution is introduced within the known genotypes. Currently, there are 3 models, uniform (across all markers), uniform within each specific marker, and the SimWalk2 error model.
- Added a batch file which will automatically fill most user input up to the analysis-specific menus. See the Mega2 Batch section for details.
- PREST analysis option - Relationship estimation
- PAP analysis option
- Simwalk2/Merlin interface option for NPL This creates two sets of data files, Merlin format files and Simwalk2 format files with a modified npl.sh script that first executes Merlin, then feeds Merlin’s p-values into Simwalk2. See documentation for more details and on how to use this option.
- **SimWalk2 graphs:** For Simwalk2 - NPL, we can now create an R script to plot the LOD scores and store them in a postscript file. There are user menus to set options for these plots.
- **Allegro graphs:** We have added an R script for graphing Allegro results.
- The analysis menu is changed in appearance, it is in two columns with slightly different analysis names in order to accommodate the additional analysis options.
- We now allow an additional field (:ID) in the input linkage format pedigree file as well as pre-makeped format pedigree file. The post-makeped file can also contain the :Ped and :Per fields in addition to the :ID field.
- Linkage format now warns if there are “real” phenotypes which are 0.0 which is the default “unknown” value in Linkage.
- Mega2 was computing the biased (population) std-dev value, this has been changed to the unbiased (sample) std-dev.

## Changes from Ver 2.2 R2 - >  Ver 2.2 R3 (Released January 23rd, 2002)

- For X-linked markers, Mega2 Version 2.2 R2 was incorrectly finding inheritance errors that weren’t really there. This has been fixed.
- New allele frequency summary format: now the histogram is drawn along with the table. For sex-linked markers, we have an overall table without histograms, then the sex-specific tables with histograms.
- SAGE 3.0 option was buggy, the formats of the fsp and fcor files were incorrect. The shell file did not work correctly. New implementation now has correct formats. Handling of multiple trait loci is improved: Excerpt of mega2 message from reordering-traits menu: “If multiple affection status loci are selected, FCOR files will be set up in trait sub-directories. SIBPAL files will be set up in the main directory If multiple QTLs are selected, FCOR and SIBPAL files will be set up in the main directory.” Other changes include elimination of “no match” messages from the “rm” command inside the shell files. In general, this option has undergone major re-vamping. Also, if there are QTLs only, the file-names menu does not ask for sage\_cnt and cntpar file names. The list of files displayed as created at the end of a run also match this behavior.
- Mega2 LOG file has more details on the run options such as Mega2 version number, pedigree omission criteria, analysis option etc.
- Half-types have to be compulsorily set to 0/0 for Mendel and Simwalk options, so the reset-option menu is not presented to the user if Mega2 detects half-types, instead a notification is printed.
- Mega2 exits if no loci are selected via option 2 reordering.
- Mega2 now checks for empty input files in addition to DOS files. It terminates with an error message.
- For ASPEX, Simwalk and Sage4 options, the marker order is checked for negative intervals (only for user-input order). Files are created with a warning.
- The log and error files were being appended to instead of overwritten. This has been rectified.
- Mega2 crashed if no marker loci were selected, this has been corrected.

## Changes from version 2.2 - >  version 2.2 R2 (Released 28th June 2001)

- Expiry date checking has been fixed.
- Checking for too many alleles within a sibship produced spurious error messages because it did not handle half-siblings properly. It now only checks full siblings.
- Menu for resetting genotypes has been fixed, the EXIT option did not work properly, it failed to terminate Mega2.

## Changes from version 2.1 beta R3 - >  version 2.2 (Released 15 Jun 2001)

- Partial ordering of individuals inside a pedigree that simply makes sure that parents are numbered lower than offspring instead of the more rigorous sorting that was being used (tried to sort uncles half-siblings too), which caused infinite loops for highly inbred pedigrees.
- Added a new SimWalk2 option called Mistyping analysis, in anticipation of the release of SimWalk2 version 2.82.
- Other minor changes in the batch file for Simwalk2.
- Added check for DOS format input files, Mega2 exits with a message if any file is a DOS format file.
- Better detection of Mendelian inconsistencies in the presence of untyped parents.
- User has the option of specifying which set of invalid genotypes to set to (0,0), half-typed, Mendelian inconsistencies, neither or both. So the “Inconsistencies found - how shall we proceed” menu looks a little bit different now.
- Fixed SLINK to handle QTLs properly - does not ask about affecteds if trait chosen is a QTL.
- More information about unconnected components of a pedigree is displayed, consisting of some of the individuals that make up the pedigree.
- Fixed bugs in how Mendel handles a set of loci with only QTLs.
- Fixed handling of allele\_count option for untyped markers, these are skipped as they should be.
- Corrected shell file headers for ASPEX and SPLINK.

## Changes from version 2.1 beta R2 - >  version 2.1 beta R3 (Released 30 Mar 2001)

- Bug found and fixed in SPLINK whereby a space was missing between the affection status locus and following data.
- Added affection status definition menu inside the SPLINK option, since SPLINK does not handle multiple liability classes.
- More informative error messages for input Pedigree files.
- Fixed string overflow problem where the long version of output option name is stored.
- Further fix to Vitesse Linkmap files, ordinal locus numbers are NOT used anymore, so that the program “map” can create a composite map from several windows analyzed by Vitesse Linkmap option.

## Changes from version 2.1 beta - >  version 2.1 beta R2 (Released 16 Mar 2001)

- Bug in Vitesse LOG and STM files, the loci numbers and names were incorrect. These are generated correctly now.
- Bug in ASPEX sib-phase graph scripts for the theta grid option, the files gph.\*.\*.\*. < chr >  files did not have the proper curve data. This has been fixed.
- Added some checking in Vitesse that verifies user-input interval size inside Linkmap. If input consists of a single chromosome, this is checked within the Linkmap interval menu, otherwise, it is checked during the processing of each chromosome. If there are fewer markers on any chromosome than the requested interval size, the user is given the opportunity to define a smaller size for that chromosome, the original length is used for chromosomes that have the requisite number of markers.
- Bug in Output linkage format files (GeneHunter options, SLINK, Vitesse etc.), the affection status and liability class columns ran into each other. They are separated with a space now.

## Changes from version 2.05 to version 2.1 beta (Released 16 Feb 2001)

- Added support for pre-Makeped files.
- Automatic loop-breaker selection for options that need post-Makeped pedigree files.
- Multiple marriages are allowed, and one loop-breaker can break more than 1 loop.
- Added support for Allegro, MLBQTL and S.A.G.E. 4.0 formats.
- Map files with kosambi distances are allowed.
- Original person IDs can be output in Mendel and Aspex output files, if provided in the post-Makeped format pedigree file.
- Untyped pedigree omission options to specify minimum number of genotyped individuals in a pedigree for it to be included in the output.
- Easier way to select loci in option 2 of locus reordering menu by specifying a range e.g “1-10”. Ranges can be interspersed with specific locus numbers each separated by whitespace (e.g., 1-5 9 11 12-15).
- Handling of multiple trait loci, where the analysis option allows such data. Otherwise, selection of multiple traits causes separate sets of output files to be produced for each trait.
- Improved handling of QTLs. Now has checks to see that QTLs are included in output only if the analysis option allows them.
- Simwalk2 batch file now uses

# 29 List of fixed bugs

This section is no longer being updated because it is redundant with the ’Changes made to Mega2’ section above.

**Last updated: June 15, 2011**

## Bugs in Mega2 4.0 R5.2 Beta

The use of unique Ped\_Per IDs like 1\_21 was improperly turned on in PAP, so the resulting trip.dat was no longer in proper PAP format.

When doing quantitative summaries output to a sub-directory, the phenotyping table got written into the subdirectory, but the header got written into the root directory.

When converting annotated files to Merlin format, the MEGA2.ERR file had two strange extraneous lines at the end.

When converting to Merlin while zeroing out half-typed people, the “Half-typed genotypes” in MEGA2.RESET was incomplete.

When default batch file options are set to ’yes’, Mega2 no longer stops at the ’SPLINK option selection’ menu when in batch mode.

Under certain conditions, an array storing trait loci indicies was too short in the locus reordering function ReOrderLociByPositionNumber.

When only markers were selected, the SUP routine read off the end of the line and created incorrect files. SUP requires at least one trait locus, so this restriction has been implemented.

Conversion to SLINK format did not work correctly if the trait locus was not at the beginning of the locus list.

Corrected behavior of the ’Disease locus selection menu’ for the SLINK option. It was giving the wrong prompt and not correctly indicating the currently selected option.

Under certain conditions, one of the theta values was being written into the wrong position of the ’theta’ array when converting to SLINK format.

When setting up for a Quantitative Trait Summary in batch mode, the loci were reordered twice when they only needed to be reordered once.

The create\_nuked\_fids() function, when trying to rename the pedigree ID, wrote a string into itself, generating a ’source and destination overlap in memcpy’ error.

Failed to detect when the input batch file contained a trait number out of bounds. Added this check in.

In copy\_annotated\_to\_premake(), there was an error in the logic which led to reading a value from beyond the end of the persons[] array.

There was a failure to initialize the missing\_quant value correctly when none of the trait loci were quantitative and only the covariates were quantitative.

When manually-reordering using the GeneHunter option, the code contained an error where it tried to access the ’-1’ element of the global\_trait\_entries[] array.

The check\_map\_positions() function was not printing error messages properly.

The ’Locus Reordering Menu’ in the Quantitative Summary option was not working correctly.

When reading an annotated pedigree file that contains unmapped markers (chromosome ’U’), the cumulative counts of markers per chromosomes in the chromo\_loci\_counts array was missing an entry for the ’unknown’ chromosome.

The ’Select by locus number’ option of the ’Locus Reordering Menu’ was not working correctly when Mega2 was run interactively (It worked correctly when Mega2 was run in batch mode).

When attempting to break two loops in a pedigree, Mega2 incorrectly assigned proband/loop IDs 1 2 2 2, but this should have been (2,2) (3,3) or (1,2) (3,3). The person assigned proband ID 1 on input ended up being a member of the second loop. Since the user can avoid such an error by changing their choice of proband or by changing their loop breaker selection criteria, a partial solution was implemented so that Mega2 exits whenever it is trying to assign a loopbreaker who is the proband to a loop other than loop number 2.

When the user chose to put the output files in a new output folder, the resulting MEGA2run.html did not work properly because the time-dated summary folder was not being copied into the new output folder.

## Bugs in Mega2 4 R5.1

Merlin plots contained an extra straight line when the steps option is selected.

For X-linked and Y-linked data, homozygous genotypes were mistakenly flagged as being heterozygous and vice versa, and always labeled as X-chromosome errors.

The allele-frequency summary option gave wrong counts and frequencies on Y-linked markers.

## Bugs in Mega2 4 R5.0

The allele summary option did not include half-typed individuals’ genotypes in the counts, even if their genotypes are passed through to the output.

The l2a.py converter script did not check the input pedigree file to make sure that each line contains the required number of headers, this may have resulted in an invalid annotated format pedigree file.

Mega2 did not allow setting unknown phenotype value for covariates.

The linkage option was missing the Ped: value field at the end, if the input pedigree file did not contain a Ped: field.

## Bugs in Mega2 4 R4.0

Mendel8 format control file contained multiple affection definitions if the user opted for combined output of multiple affection traits, causing Mendel to fail. Now, only the first affection trait is listed in the control file.

Mega2 aborted right after starting up on Mac OS X Snow Leopard machines. This has been fixed.

## Bugs in Mega2 4 R3.1

Mendel7+ and X-linked markers: if the input map file contains only sex-averaged map positions, the Mendel map file positions would be set to 0 for annotated format input file. This has been fixed; average positions are taken to be the female positions, and male positions are set to 0.

## Bugs in Mega2 4 R3

Mendel 9.0 did not accept, nor use any special missing quantitative phenotype value when analyzing quantitative traits, instead, it used the default value of 0.0.

Linkage format options (LINKAGE, SLINK, SPLINK, SIMULATE, VITESSE etc.) erroneously set the first paternal and maternal sibling fields to 0, even when such siblings were present inside the pedigree.

## Bugs in Mega2 4.0 R1

PLINK long format output files were not named correctly, as per PLINK specifications.

PLINK shell script had problems and failed to run. This has been fixed.

Affection status inside the PLINK fam file was set to 0 for everyone.

Newest version of Mendel does not accept the variable file, which lists quantitative variables, instead quantitative loci are now added to the definition file (which is named mendel\_locus.\* by default).

Mega2 crashed if frequency file was not specified. This has been fixed.

The menu option for changing the unknown status and allele designator in the first menu was not working.

Conversion from pre-makeped format to post-makeped format output options (such as SLINK, Vitesse etc.) created wrong first-sibling links to be created for pedigrees that had half-siblings. This has been fixed.

Allegro, Gene Hunter, Gene Hunter-Plus and MLB-QTL output locus files had default trait penetrances set to 0.0 rather than the usual 0.5.

## Bugs in Mega2 4.0

Output file names were garbled when output folder name had a “.” inside it. This has been fixed.

SLINK crashed when multiple chromosomes were selected. This has been fixed.

Rsimwalk2.pl did not read in SimWalk2 scores correctly.

Quantitative summary gave wrong standard deviation values. This has been fixed.

Random simulation of genotyping errors did not work correctly from 4.0 beta onwards. This has been fixed.

## Bugs in Mega2 4.0 beta R1

Annotated files caused Mega2 to crash if a frequency file was not provided. This has been fixed, and Mega2 will now calculate observed allele frequencies from the pedigree data as usual.

Merlin format output files were missing covariates, if these were included in the selected traits.

For these options, Mega2 falsely detected Mendelian inheritance errors for male X-linked genotypes: PREST, HWE (all options), SUMMARIES (all options).

Several bugs in handling of X-linked data have been fixed for options that allow X-linked analysis on data that contains both autosomal and X-linked loci. These include setting special X-linked analysis flags for the X-chromosome output.

R plot menu behavior was incorrect.

## Bugs in Mega2 4.0 beta

These options caused mega2 to segfault:   
1) Nuclear pedigree option, 2) Aspex option crashed, 3) PAP option.

Sage 3.0 sibpal parameter file was wrong.

R plot menu wasn’t working for a single trait in trait-combine mode. Note that plots are still restricted to the trait-specific mode, if there are multiple trait loci.

Affected sib-pair summaries had wrong parental affection status.

In the Mendel formatted locus files, mendel\_locus (Mendel 7+), locus (Mendel 3.0) , and LOCUS (SimWalk2), long trait names were not shortened to 8 characters.

## Bugs in Mega2 3.0 R11

Nuclear pedigree files had redundant chromosome number extensions when multiple chromosomes were selected.

## Bugs in Mega2 3.0 R10

In some cases, large pedigrees in pre-makeped format files caused Mega2 to crash.

## Bugs in Mega2 3.0 R9

Several options produced wrong pedigree file with missing pedigree IDs when using a pre-makeped pedigree file as input. These involve options which skip pre-makeped to post-makeped conversion including Mendel-format options, Merlin, Prest, and Solar.

Reordering of traits contained a bug, where the trait locus was set to the wrong locus, if user changed the ordering from the input order.

Old SAGE-format files wrote spaces for missing trait values instead of a missing value.

HLOD scores were not read in from Allegro’s output for plotting. These scores are produced by Allegro’s parametric linkage analysis.

HWE files for Mendel’s Hardy-Weinberg estimation contained completely ungenotyped individuals.

Loop-breaking in premakeped format pedigrees produced false error messages about failure to break loops for pedigrees that did not contain loops.

## Bugs in Mega2 3.0 R8

The old Mendel-format marker file was incorrectly read in by the RELPAIR conversion Perl script because there was no space between the chromosome number and marker position.

The Rallegro.pl, Rmerlin.pl and Rsimwalk2.pl Perl scripts failed to distinguish between marker loci that had identical map positions on a chromosome (very dense markers would have the same problem, if the output did not have enough precision), producing incorrectly formatted plot-output files.

## Bugs in Mega2 3.0 R7

Premakeped-format pedigrees with affection status loci caused Mega2 to crash. This has been fixed in 3.0R8.

For pre-makeped format files, output files did not contain pedigree numbers, if user selected the premakeped pedigree IDs as the output pedigree id.

Mega2 failed to reset Mendelianly inconsistent genotypes even if the menu item for this action in the “Incorrect genotypes reset menu” was set to yes.

## Bugs in Mega2 3.0 R5, R6

Unconnected individuals in premakeped-format pedigrees went undetected, if the pedigrees consisted only of disconnected individuals. This has been fixed.

Mendel option did not label X-linked loci as such when the combined output option was selected.

## Bugs in Mega2 3.0 R4

Merlin format pedigree files created by Mega2 were not read in correctly by Merlin if affection status traits included a liability column, since there is no way to specify such loci using the QTDT-format names file. Now, when Mega2 is converting to Merlin-format and it encounters an affection status trait with a liability column, the user is prompted to select status-class labels to be designated as affected, and only the status is output in the pedigree file.

When a names file is used so that alleles are recoded, the random selection of individuals was not working correctly. Hence, allele frequencies computed via options 2 and 3 of the individual-selection menu may have been incorrect under certain conditions. Option 2 selects typed founders or a random member if a pedigree has no typed founders, and option 3 includes individuals selected via option 2 as well as one non-founder per pedigree with an allele not found by option 2. For more details, see the section on recode bug.

When recoding took place, Option 3 of of individual selection menu did not count all alleles present in data - it still gave 0 allele counts.

Incorrect displaying of omitted pedigree and person numbers has been fixed.

Allegro script for running and plotting results of analysis on multiple traits did not work. This has been fixed.

The R shell script contained mal-formed file-names when a single trait was selected for Merlin and SimWalk2 such as (null)/merlin.01.out. This has been fixed.

## Bugs in Mega2 3.0 R3

In recoding alleles, wrong frequencies were being assigned to the alleles if and only if the ORIGINAL alleles were numbered 1 to N (N  > = 10), making renaming unnecessary.

Due to floating-point precision error, the missing quantitative phenotype value was not read in correctly.

When recoding alleles, total allele count was wrong in MEGA2.RECODE file.

## Bugs in Mega2 3.0 R2

The R options for Hardy-Weinberg equilibrium testing were generating spurious error messages on skipping markers.

Wrong column names in the output table created by the HWE R exact option.

Unique IDs were not carried over during conversion from Pre-makeped format to post-makeped format.

## Bugs in Mega2 3.0 R1

The ID: field was not read in when multiple chromosomes were selected for analysis.

HWE-R EXACT option: R-script was wrong.

Mendel5 pedigree files contained quantitative variable for affection loci at the end of each record, which does not work with the new version of Mendel.

Mendel5 option did not produce a variable file for defining QTLs.

Selection of individuals for allele-frequency estimation using the founders + all unique alleles did not work. It still produced zero allele frequencies which should not be the case. for Mendel.

HWE R options: Spurious error message even when table was created correctly.

Linkage option - Only pedigrees with non-numeric names assigned numbers causing possible conflicts with pedigrees whose original numeric IDs were maintained in the output.

Merlin gave a segmentation-fault, if the user selected only one statistic for graphical output.

Vitesse shell script was missing its header and hence could not be run.

## Bugs in Mega2 3.0

Hardy-Weinberg - HWE option (Guo and Thompson) had a bug, it either crashed or produced wrong genotype counts.

SimWalk2-npl option crashed while setting R-plots

Merlin’s R-plot shell script had the wrong file names and didn’t work for a single trait.

In rare cases, Mega2 crashed while breaking loops.

In the Combine-traits mode, Mega2 would not allow selection of all the markers and traits.

HWE-R based options produced incorrect MEGA2.KEYS file.

Corrected handling of missing R-formatted data files for R-graphics - Allegro continued through the reading even when files were missing

HWE-Mendel file names menu had two options numbered 3.

## Bugs in Mega2 2.5 R4

SAGE4 parameter file was wrong. The field-widths were not set correctly, caused the pedigree data to be read in incorrectly.

X-linked analysis was not working, caused Mega2 to crash.

In the BATCH mode, Mega2 appended new declarations for Value\_MissingQuant to any existing MEGA2.BATCH file. This has been fixed.

SAGE 4.0 parameter file now includes a default 0 as missing genotype value to prevent error messages in the GENIBD output about unrecognized genotype value.

Mendel5 option created incorrect keys file.

Duplicate names in the names files caused Mega2 to run incorrectly. These are detected and causes Mega2 to exit.

## Bugs in Mega2 2.5 R3

SOLAR bug fixed: In the “loop-over traits” mode person IDs in the phenotype file did not match person IDs in the pedigree file.

## Bugs in Mega2 2.5 R2

SOLAR bug fixed: Mega2 crashed while looping over quantitative loci.

RECODE bug fixed: Mega2 crashed while recoding loci from *names file* , if there were no mapped loci.

MERLIN bug fixed: Merlin analysis option menu was displayed twice.

GENOTYPING ERROR simulation mode bug fixed: Locus selection was not working properly.

SIMWALK2 bug fixed: Location scores option produced an incorrect penetrance file for multiple affection liability classes.

## Bugs in Mega2 2.5 R1

ASPEX bug fixed - nuclear pedigree naming/numbering options did not allow numbering with extensions.

ASPEX bug fixed - nuclear pedigree naming/numbering options did not allow numbering with extensions.

SPLINK bug fixed - did not put all markers to be put into one file, when asked to do so.

S.A.G.E 4.x bug fixed - The format statement in the parameter file was incorrect.

R-graphics bug fixed - The output file was not renumbered according to the chromosome correctly.

## Bugs in Mega2 2.5

S.A.G.E. 3.x affected relative counts files are not set up correctly.

S.A.G.E. 4.x parameter file did not have the correct pedigree field formats.

PREST pedigree file was not set up correctly. It contained one big line.

Merlin/SimWalk2-NPL option did not work due to a mismatch in pedigree naming between the two sets of files.

Pedigree and individual id menu behavior has been fixed. The different options were not being selected and displayed correctly.

Fixed SPLINK format bug - was writing individual IDs in place of mother’s IDs.

Fixed bug in SimWalk2 for handling more than 100 liability classes.

Fixed bug in the Win 32 version of Mega2, where the locus order line was not read in correctly. The 2.5 version will work correctly however, as long as there is an extra non-numeric character before the newline.

Blank lines are allowed before the locus-order line again, the check for locus order in Ver 2.5 did not accept blank lines just before the locus order line.

## Bugs in Mega2 2.3 R4

In SOLAR, the phenotype file and marker genotype file don’t match the person IDs in the pedigree file (id choice was not implemented for the former).

In locus reordering, for multiple chromosomes and multiple traits, traits were being output at the beginning of the order irrespective of the user’s ordering.

Selection of loci for random genotype error detection from the batch file was incorrectly implemented.

>  Renaming chromosome number extension for some output files was wrong.

Segmentation fault for 0 recombination fractions (where there is only a single locus in premakeped-format output files.

Existing vstrm files were not removed before each Vitesse run. This would cause the old file to be used when Vitesse failed to create a new one.

## Bugs in Mega2 2.3 R3

SimWalk2 penetrance file had invalid liability class values. Acceptable values are 1-99.

SPLINK option produced garbled pedigree files if the user selected the option to combine all markers into one pedigree file.

## Bugs in Mega2 2.3 R2

For SOLAR, the HHID phenotype was being written into the phenotype file, this has been moved to the pedigree file.

Bug in loop reconnection - Mega2 assumed that the proband numbers were ordered in the pedigree file which caused the numbering of links (first\_off, sibs etc.) to be wrong.

Individual-count for pre-makeped format input pedigrees (in the screen output as well as log file) was wrong.

Selecting only trait loci in S.A.G.E. 4.0 resulted in a segfault.

## Bugs in Mega2 2.3 and Mega2 2.3 R1

Versions 2.3 and 2.3 R1 may have had a segmentation fault when creating nuclear pedigrees from post-makeped pedigrees which did not have person IDs numbered consecutively from 1 through the number of pedigree members. Nuclear pedigrees are also created in the S.A.G.E. 3.0, ASPEX, and GHMLB options. Pre-makeped pedigrees do not cause segmentation faults in either version 2.3.

Post-makeped pedigrees with non-ordinal entry IDs may have also created segmentation faults in the “create summary” option for segregation summary. Again pre-makeped files are not a problem.

Mendel pedigree file format statement has been fixed. The format statement for reading in pedigree members was incorrect.

The script sage\_cnt.\*.sh was not working in case of a single affection trait. This script has been corrected.

In some cases, nuclear families included half-sibs This bug affects Aspex, Affected Sib Counts, GHMLB and S.A.G.E.

## Bugs in Mega2 2.2 R3

Loop reconnection had a bug. If the individual with the proband field set to 1 appeared AFTER individuals with other proband numbers, the loops were not reconnected.

Computation of sample variance for QTLs was biased before.

HWE option crashed if any marker locus had less than 3 alleles using GUO’s hwe program.

## Bugs in Mega2 2.2 R2

Spurious inheritance errors for X-linked loci namely too many distinct alleles, even when there were at most three alleles in a mixed-gender sibship.

S.A.G.E. 3.0 fsp and fcor file formats were wrong. Shell file also had a bug. Handling of multiple trait loci was not correctly implemented.

The LOG and ERROR files were appended to in each run, instead of being overwritten.

Segmentation fault if no loci were selected.

## Bugs in Mega2 2.2

The Mendelian inconsistency checking routine produces spurious inconsistency messages because it does not handling half-siblings properly.

The “reset genotypes” menu does not EXIT, but continues to loop. After a few turns this will result in a buffer overflow as it keeps appending to the message at the bottom of the menu.

## Bugs in Mega2 2.1 beta

This is a list of bugs reported by users of Mega2 2.1 beta:

No spacing between the liability status and liability class if the selected affection locus had multiple liability classes. Affected options are:

4) Convert to GeneHunter-Plus format 5) Convert to GeneHunter-format 9) Convert to SLINK-format 11) Set up for homogeneity analyses 12) Convert to SIMULATE-format 17) Convert to Vitesse-format 18) Convert to Linkage-format 20) Convert to Allegro-format 21) Convert to MLBQTL format

\endverbatim

The sib-phase XMGR scripts do not work for a grid of risk-ratios, the curve-data is missing the second column.

The Vitesse LOG and STM files may contain bogus locus names and numbers, and, therefore, may not produce correct results.

The fix to the previous bug made locus numbers ordinal (i.e. 1..N, where N is the window size, including trait) with trait locus always as locus number 1. This prevents the program “map” from creating the correct combined map from all these sliding windows. So, the locus numbers have been changed back to the original numbers. Version beta 2.1 has an additional bug in this ordering, so use Version R3 for a complete fix.

A space was missing between the affection status column and the next locus data column in the SPLINK output pedigree file.

Mega2 ran into infinite loops trying to sort pedigree members if the structure was highly complicated with inbreeding and matings across generations.

SLINK option crashed if the only trait locus chosen was a QTL.

Mendel output the first marker locus twice in the locus file if no affection locus was selected.

Allele count option did not skip untyped markers, so it gave meaningless output due to divide-by-zero errors.

Aspex and Splink options did not have the correct headers in the c-shell scripts when more than one analysis was put into the same file.

# 30 License agreements

## 30.1 GNU General Public License Version 3 for Mega2

```
                    GNU GENERAL PUBLIC LICENSE
                       Version 3, 29 June 2007
 Copyright (C) 2007 Free Software Foundation, Inc. <http://fsf.org/>
 Everyone is permitted to copy and distribute verbatim copies
 of this license document, but changing it is not allowed.
                            Preamble
  The GNU General Public License is a free, copyleft license for
software and other kinds of works.
  The licenses for most software and other practical works are designed
to take away your freedom to share and change the works.  By contrast,
the GNU General Public License is intended to guarantee your freedom to
share and change all versions of a program--to make sure it remains free
software for all its users.  We, the Free Software Foundation, use the
GNU General Public License for most of our software; it applies also to
any other work released this way by its authors.  You can apply it to
your programs, too.
  When we speak of free software, we are referring to freedom, not
price.  Our General Public Licenses are designed to make sure that you
have the freedom to distribute copies of free software (and charge for
them if you wish), that you receive source code or can get it if you
want it, that you can change the software or use pieces of it in new
free programs, and that you know you can do these things.
  To protect your rights, we need to prevent others from denying you
these rights or asking you to surrender the rights.  Therefore, you have
certain responsibilities if you distribute copies of the software, or if
you modify it: responsibilities to respect the freedom of others.
  For example, if you distribute copies of such a program, whether
gratis or for a fee, you must pass on to the recipients the same
freedoms that you received.  You must make sure that they, too, receive
or can get the source code.  And you must show them these terms so they
know their rights.
  Developers that use the GNU GPL protect your rights with two steps:
(1) assert copyright on the software, and (2) offer you this License
giving you legal permission to copy, distribute and/or modify it.
  For the developers’ and authors’ protection, the GPL clearly explains
that there is no warranty for this free software.  For both users’ and
authors’ sake, the GPL requires that modified versions be marked as
changed, so that their problems will not be attributed erroneously to
authors of previous versions.
  Some devices are designed to deny users access to install or run
modified versions of the software inside them, although the manufacturer
can do so.  This is fundamentally incompatible with the aim of
protecting users’ freedom to change the software.  The systematic
pattern of such abuse occurs in the area of products for individuals to
use, which is precisely where it is most unacceptable.  Therefore, we
have designed this version of the GPL to prohibit the practice for those
products.  If such problems arise substantially in other domains, we
stand ready to extend this provision to those domains in future versions
of the GPL, as needed to protect the freedom of users.
  Finally, every program is threatened constantly by software patents.
States should not allow patents to restrict development and use of
software on general-purpose computers, but in those that do, we wish to
avoid the special danger that patents applied to a free program could
make it effectively proprietary.  To prevent this, the GPL assures that
patents cannot be used to render the program non-free.
  The precise terms and conditions for copying, distribution and
modification follow.
                       TERMS AND CONDITIONS
  0. Definitions.
  "This License" refers to version 3 of the GNU General Public License.
  "Copyright" also means copyright-like laws that apply to other kinds of
works, such as semiconductor masks.
  "The Program" refers to any copyrightable work licensed under this
License.  Each licensee is addressed as "you".  "Licensees" and
"recipients" may be individuals or organizations.
  To "modify" a work means to copy from or adapt all or part of the work
in a fashion requiring copyright permission, other than the making of an
exact copy.  The resulting work is called a "modified version" of the
earlier work or a work "based on" the earlier work.
  A "covered work" means either the unmodified Program or a work based
on the Program.
  To "propagate" a work means to do anything with it that, without
permission, would make you directly or secondarily liable for
infringement under applicable copyright law, except executing it on a
computer or modifying a private copy.  Propagation includes copying,
distribution (with or without modification), making available to the
public, and in some countries other activities as well.
  To "convey" a work means any kind of propagation that enables other
parties to make or receive copies.  Mere interaction with a user through
a computer network, with no transfer of a copy, is not conveying.
  An interactive user interface displays "Appropriate Legal Notices"
to the extent that it includes a convenient and prominently visible
feature that (1) displays an appropriate copyright notice, and (2)
tells the user that there is no warranty for the work (except to the
extent that warranties are provided), that licensees may convey the
work under this License, and how to view a copy of this License.  If
the interface presents a list of user commands or options, such as a
menu, a prominent item in the list meets this criterion.
  1. Source Code.
  The "source code" for a work means the preferred form of the work
for making modifications to it.  "Object code" means any non-source
form of a work.
  A "Standard Interface" means an interface that either is an official
standard defined by a recognized standards body, or, in the case of
interfaces specified for a particular programming language, one that
is widely used among developers working in that language.
  The "System Libraries" of an executable work include anything, other
than the work as a whole, that (a) is included in the normal form of
packaging a Major Component, but which is not part of that Major
Component, and (b) serves only to enable use of the work with that
Major Component, or to implement a Standard Interface for which an
implementation is available to the public in source code form.  A
"Major Component", in this context, means a major essential component
(kernel, window system, and so on) of the specific operating system
(if any) on which the executable work runs, or a compiler used to
produce the work, or an object code interpreter used to run it.
  The "Corresponding Source" for a work in object code form means all
the source code needed to generate, install, and (for an executable
work) run the object code and to modify the work, including scripts to
control those activities.  However, it does not include the work’s
System Libraries, or general-purpose tools or generally available free
programs which are used unmodified in performing those activities but
which are not part of the work.  For example, Corresponding Source
includes interface definition files associated with source files for
the work, and the source code for shared libraries and dynamically
linked subprograms that the work is specifically designed to require,
such as by intimate data communication or control flow between those
subprograms and other parts of the work.
  The Corresponding Source need not include anything that users
can regenerate automatically from other parts of the Corresponding
Source.
  The Corresponding Source for a work in source code form is that
same work.
  2. Basic Permissions.
  All rights granted under this License are granted for the term of
copyright on the Program, and are irrevocable provided the stated
conditions are met.  This License explicitly affirms your unlimited
permission to run the unmodified Program.  The output from running a
covered work is covered by this License only if the output, given its
content, constitutes a covered work.  This License acknowledges your
rights of fair use or other equivalent, as provided by copyright law.
  You may make, run and propagate covered works that you do not
convey, without conditions so long as your license otherwise remains
in force.  You may convey covered works to others for the sole purpose
of having them make modifications exclusively for you, or provide you
with facilities for running those works, provided that you comply with
the terms of this License in conveying all material for which you do
not control copyright.  Those thus making or running the covered works
for you must do so exclusively on your behalf, under your direction
and control, on terms that prohibit them from making any copies of
your copyrighted material outside their relationship with you.
  Conveying under any other circumstances is permitted solely under
the conditions stated below.  Sublicensing is not allowed; section 10
makes it unnecessary.
  3. Protecting Users’ Legal Rights From Anti-Circumvention Law.
  No covered work shall be deemed part of an effective technological
measure under any applicable law fulfilling obligations under article
11 of the WIPO copyright treaty adopted on 20 December 1996, or
similar laws prohibiting or restricting circumvention of such
measures.
  When you convey a covered work, you waive any legal power to forbid
circumvention of technological measures to the extent such circumvention
is effected by exercising rights under this License with respect to
the covered work, and you disclaim any intention to limit operation or
modification of the work as a means of enforcing, against the work’s
users, your or third parties’ legal rights to forbid circumvention of
technological measures.
  4. Conveying Verbatim Copies.
  You may convey verbatim copies of the Program’s source code as you
receive it, in any medium, provided that you conspicuously and
appropriately publish on each copy an appropriate copyright notice;
keep intact all notices stating that this License and any
non-permissive terms added in accord with section 7 apply to the code;
keep intact all notices of the absence of any warranty; and give all
recipients a copy of this License along with the Program.
  You may charge any price or no price for each copy that you convey,
and you may offer support or warranty protection for a fee.
  5. Conveying Modified Source Versions.
  You may convey a work based on the Program, or the modifications to
produce it from the Program, in the form of source code under the
terms of section 4, provided that you also meet all of these conditions:
    a) The work must carry prominent notices stating that you modified
    it, and giving a relevant date.
    b) The work must carry prominent notices stating that it is
    released under this License and any conditions added under section
    7.  This requirement modifies the requirement in section 4 to
    "keep intact all notices".
    c) You must license the entire work, as a whole, under this
    License to anyone who comes into possession of a copy.  This
    License will therefore apply, along with any applicable section 7
    additional terms, to the whole of the work, and all its parts,
    regardless of how they are packaged.  This License gives no
    permission to license the work in any other way, but it does not
    invalidate such permission if you have separately received it.
    d) If the work has interactive user interfaces, each must display
    Appropriate Legal Notices; however, if the Program has interactive
    interfaces that do not display Appropriate Legal Notices, your
    work need not make them do so.
  A compilation of a covered work with other separate and independent
works, which are not by their nature extensions of the covered work,
and which are not combined with it such as to form a larger program,
in or on a volume of a storage or distribution medium, is called an
"aggregate" if the compilation and its resulting copyright are not
used to limit the access or legal rights of the compilation’s users
beyond what the individual works permit.  Inclusion of a covered work
in an aggregate does not cause this License to apply to the other
parts of the aggregate.
  6. Conveying Non-Source Forms.
  You may convey a covered work in object code form under the terms
of sections 4 and 5, provided that you also convey the
machine-readable Corresponding Source under the terms of this License,
in one of these ways:
    a) Convey the object code in, or embodied in, a physical product
    (including a physical distribution medium), accompanied by the
    Corresponding Source fixed on a durable physical medium
    customarily used for software interchange.
    b) Convey the object code in, or embodied in, a physical product
    (including a physical distribution medium), accompanied by a
    written offer, valid for at least three years and valid for as
    long as you offer spare parts or customer support for that product
    model, to give anyone who possesses the object code either (1) a
    copy of the Corresponding Source for all the software in the
    product that is covered by this License, on a durable physical
    medium customarily used for software interchange, for a price no
    more than your reasonable cost of physically performing this
    conveying of source, or (2) access to copy the
    Corresponding Source from a network server at no charge.
    c) Convey individual copies of the object code with a copy of the
    written offer to provide the Corresponding Source.  This
    alternative is allowed only occasionally and noncommercially, and
    only if you received the object code with such an offer, in accord
    with subsection 6b.
    d) Convey the object code by offering access from a designated
    place (gratis or for a charge), and offer equivalent access to the
    Corresponding Source in the same way through the same place at no
    further charge.  You need not require recipients to copy the
    Corresponding Source along with the object code.  If the place to
    copy the object code is a network server, the Corresponding Source
    may be on a different server (operated by you or a third party)
    that supports equivalent copying facilities, provided you maintain
    clear directions next to the object code saying where to find the
    Corresponding Source.  Regardless of what server hosts the
    Corresponding Source, you remain obligated to ensure that it is
    available for as long as needed to satisfy these requirements.
    e) Convey the object code using peer-to-peer transmission, provided
    you inform other peers where the object code and Corresponding
    Source of the work are being offered to the general public at no
    charge under subsection 6d.
  A separable portion of the object code, whose source code is excluded
from the Corresponding Source as a System Library, need not be
included in conveying the object code work.
  A "User Product" is either (1) a "consumer product", which means any
tangible personal property which is normally used for personal, family,
or household purposes, or (2) anything designed or sold for incorporation
into a dwelling.  In determining whether a product is a consumer product,
doubtful cases shall be resolved in favor of coverage.  For a particular
product received by a particular user, "normally used" refers to a
typical or common use of that class of product, regardless of the status
of the particular user or of the way in which the particular user
actually uses, or expects or is expected to use, the product.  A product
is a consumer product regardless of whether the product has substantial
commercial, industrial or non-consumer uses, unless such uses represent
the only significant mode of use of the product.
  "Installation Information" for a User Product means any methods,
procedures, authorization keys, or other information required to install
and execute modified versions of a covered work in that User Product from
a modified version of its Corresponding Source.  The information must
suffice to ensure that the continued functioning of the modified object
code is in no case prevented or interfered with solely because
modification has been made.
  If you convey an object code work under this section in, or with, or
specifically for use in, a User Product, and the conveying occurs as
part of a transaction in which the right of possession and use of the
User Product is transferred to the recipient in perpetuity or for a
fixed term (regardless of how the transaction is characterized), the
Corresponding Source conveyed under this section must be accompanied
by the Installation Information.  But this requirement does not apply
if neither you nor any third party retains the ability to install
modified object code on the User Product (for example, the work has
been installed in ROM).
  The requirement to provide Installation Information does not include a
requirement to continue to provide support service, warranty, or updates
for a work that has been modified or installed by the recipient, or for
the User Product in which it has been modified or installed.  Access to a
network may be denied when the modification itself materially and
adversely affects the operation of the network or violates the rules and
protocols for communication across the network.
  Corresponding Source conveyed, and Installation Information provided,
in accord with this section must be in a format that is publicly
documented (and with an implementation available to the public in
source code form), and must require no special password or key for
unpacking, reading or copying.
  7. Additional Terms.
  "Additional permissions" are terms that supplement the terms of this
License by making exceptions from one or more of its conditions.
Additional permissions that are applicable to the entire Program shall
be treated as though they were included in this License, to the extent
that they are valid under applicable law.  If additional permissions
apply only to part of the Program, that part may be used separately
under those permissions, but the entire Program remains governed by
this License without regard to the additional permissions.
  When you convey a copy of a covered work, you may at your option
remove any additional permissions from that copy, or from any part of
it.  (Additional permissions may be written to require their own
removal in certain cases when you modify the work.)  You may place
additional permissions on material, added by you to a covered work,
for which you have or can give appropriate copyright permission.
  Notwithstanding any other provision of this License, for material you
add to a covered work, you may (if authorized by the copyright holders of
that material) supplement the terms of this License with terms:
    a) Disclaiming warranty or limiting liability differently from the
    terms of sections 15 and 16 of this License; or
    b) Requiring preservation of specified reasonable legal notices or
    author attributions in that material or in the Appropriate Legal
    Notices displayed by works containing it; or
    c) Prohibiting misrepresentation of the origin of that material, or
    requiring that modified versions of such material be marked in
    reasonable ways as different from the original version; or
    d) Limiting the use for publicity purposes of names of licensors or
    authors of the material; or
    e) Declining to grant rights under trademark law for use of some
    trade names, trademarks, or service marks; or
    f) Requiring indemnification of licensors and authors of that
    material by anyone who conveys the material (or modified versions of
    it) with contractual assumptions of liability to the recipient, for
    any liability that these contractual assumptions directly impose on
    those licensors and authors.
  All other non-permissive additional terms are considered "further
restrictions" within the meaning of section 10.  If the Program as you
received it, or any part of it, contains a notice stating that it is
governed by this License along with a term that is a further
restriction, you may remove that term.  If a license document contains
a further restriction but permits relicensing or conveying under this
License, you may add to a covered work material governed by the terms
of that license document, provided that the further restriction does
not survive such relicensing or conveying.
  If you add terms to a covered work in accord with this section, you
must place, in the relevant source files, a statement of the
additional terms that apply to those files, or a notice indicating
where to find the applicable terms.
  Additional terms, permissive or non-permissive, may be stated in the
form of a separately written license, or stated as exceptions;
the above requirements apply either way.
  8. Termination.
  You may not propagate or modify a covered work except as expressly
provided under this License.  Any attempt otherwise to propagate or
modify it is void, and will automatically terminate your rights under
this License (including any patent licenses granted under the third
paragraph of section 11).
  However, if you cease all violation of this License, then your
license from a particular copyright holder is reinstated (a)
provisionally, unless and until the copyright holder explicitly and
finally terminates your license, and (b) permanently, if the copyright
holder fails to notify you of the violation by some reasonable means
prior to 60 days after the cessation.
  Moreover, your license from a particular copyright holder is
reinstated permanently if the copyright holder notifies you of the
violation by some reasonable means, this is the first time you have
received notice of violation of this License (for any work) from that
copyright holder, and you cure the violation prior to 30 days after
your receipt of the notice.
  Termination of your rights under this section does not terminate the
licenses of parties who have received copies or rights from you under
this License.  If your rights have been terminated and not permanently
reinstated, you do not qualify to receive new licenses for the same
material under section 10.
  9. Acceptance Not Required for Having Copies.
  You are not required to accept this License in order to receive or
run a copy of the Program.  Ancillary propagation of a covered work
occurring solely as a consequence of using peer-to-peer transmission
to receive a copy likewise does not require acceptance.  However,
nothing other than this License grants you permission to propagate or
modify any covered work.  These actions infringe copyright if you do
not accept this License.  Therefore, by modifying or propagating a
covered work, you indicate your acceptance of this License to do so.
  10. Automatic Licensing of Downstream Recipients.
  Each time you convey a covered work, the recipient automatically
receives a license from the original licensors, to run, modify and
propagate that work, subject to this License.  You are not responsible
for enforcing compliance by third parties with this License.
  An "entity transaction" is a transaction transferring control of an
organization, or substantially all assets of one, or subdividing an
organization, or merging organizations.  If propagation of a covered
work results from an entity transaction, each party to that
transaction who receives a copy of the work also receives whatever
licenses to the work the party’s predecessor in interest had or could
give under the previous paragraph, plus a right to possession of the
Corresponding Source of the work from the predecessor in interest, if
the predecessor has it or can get it with reasonable efforts.
  You may not impose any further restrictions on the exercise of the
rights granted or affirmed under this License.  For example, you may
not impose a license fee, royalty, or other charge for exercise of
rights granted under this License, and you may not initiate litigation
(including a cross-claim or counterclaim in a lawsuit) alleging that
any patent claim is infringed by making, using, selling, offering for
sale, or importing the Program or any portion of it.
  11. Patents.
  A "contributor" is a copyright holder who authorizes use under this
License of the Program or a work on which the Program is based.  The
work thus licensed is called the contributor’s "contributor version".
  A contributor’s "essential patent claims" are all patent claims
owned or controlled by the contributor, whether already acquired or
hereafter acquired, that would be infringed by some manner, permitted
by this License, of making, using, or selling its contributor version,
but do not include claims that would be infringed only as a
consequence of further modification of the contributor version.  For
purposes of this definition, "control" includes the right to grant
patent sublicenses in a manner consistent with the requirements of
this License.
  Each contributor grants you a non-exclusive, worldwide, royalty-free
patent license under the contributor’s essential patent claims, to
make, use, sell, offer for sale, import and otherwise run, modify and
propagate the contents of its contributor version.
  In the following three paragraphs, a "patent license" is any express
agreement or commitment, however denominated, not to enforce a patent
(such as an express permission to practice a patent or covenant not to
sue for patent infringement).  To "grant" such a patent license to a
party means to make such an agreement or commitment not to enforce a
patent against the party.
  If you convey a covered work, knowingly relying on a patent license,
and the Corresponding Source of the work is not available for anyone
to copy, free of charge and under the terms of this License, through a
publicly available network server or other readily accessible means,
then you must either (1) cause the Corresponding Source to be so
available, or (2) arrange to deprive yourself of the benefit of the
patent license for this particular work, or (3) arrange, in a manner
consistent with the requirements of this License, to extend the patent
license to downstream recipients.  "Knowingly relying" means you have
actual knowledge that, but for the patent license, your conveying the
covered work in a country, or your recipient’s use of the covered work
in a country, would infringe one or more identifiable patents in that
country that you have reason to believe are valid.
  If, pursuant to or in connection with a single transaction or
arrangement, you convey, or propagate by procuring conveyance of, a
covered work, and grant a patent license to some of the parties
receiving the covered work authorizing them to use, propagate, modify
or convey a specific copy of the covered work, then the patent license
you grant is automatically extended to all recipients of the covered
work and works based on it.
  A patent license is "discriminatory" if it does not include within
the scope of its coverage, prohibits the exercise of, or is
conditioned on the non-exercise of one or more of the rights that are
specifically granted under this License.  You may not convey a covered
work if you are a party to an arrangement with a third party that is
in the business of distributing software, under which you make payment
to the third party based on the extent of your activity of conveying
the work, and under which the third party grants, to any of the
parties who would receive the covered work from you, a discriminatory
patent license (a) in connection with copies of the covered work
conveyed by you (or copies made from those copies), or (b) primarily
for and in connection with specific products or compilations that
contain the covered work, unless you entered into that arrangement,
or that patent license was granted, prior to 28 March 2007.
  Nothing in this License shall be construed as excluding or limiting
any implied license or other defenses to infringement that may
otherwise be available to you under applicable patent law.
  12. No Surrender of Others’ Freedom.
  If conditions are imposed on you (whether by court order, agreement or
otherwise) that contradict the conditions of this License, they do not
excuse you from the conditions of this License.  If you cannot convey a
covered work so as to satisfy simultaneously your obligations under this
License and any other pertinent obligations, then as a consequence you may
not convey it at all.  For example, if you agree to terms that obligate you
to collect a royalty for further conveying from those to whom you convey
the Program, the only way you could satisfy both those terms and this
License would be to refrain entirely from conveying the Program.
  13. Use with the GNU Affero General Public License.
  Notwithstanding any other provision of this License, you have
permission to link or combine any covered work with a work licensed
under version 3 of the GNU Affero General Public License into a single
combined work, and to convey the resulting work.  The terms of this
License will continue to apply to the part which is the covered work,
but the special requirements of the GNU Affero General Public License,
section 13, concerning interaction through a network will apply to the
combination as such.
  14. Revised Versions of this License.
  The Free Software Foundation may publish revised and/or new versions of
the GNU General Public License from time to time.  Such new versions will
be similar in spirit to the present version, but may differ in detail to
address new problems or concerns.
  Each version is given a distinguishing version number.  If the
Program specifies that a certain numbered version of the GNU General
Public License "or any later version" applies to it, you have the
option of following the terms and conditions either of that numbered
version or of any later version published by the Free Software
Foundation.  If the Program does not specify a version number of the
GNU General Public License, you may choose any version ever published
by the Free Software Foundation.
  If the Program specifies that a proxy can decide which future
versions of the GNU General Public License can be used, that proxy’s
public statement of acceptance of a version permanently authorizes you
to choose that version for the Program.
  Later license versions may give you additional or different
permissions.  However, no additional obligations are imposed on any
author or copyright holder as a result of your choosing to follow a
later version.
  15. Disclaimer of Warranty.
  THERE IS NO WARRANTY FOR THE PROGRAM, TO THE EXTENT PERMITTED BY
APPLICABLE LAW.  EXCEPT WHEN OTHERWISE STATED IN WRITING THE COPYRIGHT
HOLDERS AND/OR OTHER PARTIES PROVIDE THE PROGRAM "AS IS" WITHOUT WARRANTY
OF ANY KIND, EITHER EXPRESSED OR IMPLIED, INCLUDING, BUT NOT LIMITED TO,
THE IMPLIED WARRANTIES OF MERCHANTABILITY AND FITNESS FOR A PARTICULAR
PURPOSE.  THE ENTIRE RISK AS TO THE QUALITY AND PERFORMANCE OF THE PROGRAM
IS WITH YOU.  SHOULD THE PROGRAM PROVE DEFECTIVE, YOU ASSUME THE COST OF
ALL NECESSARY SERVICING, REPAIR OR CORRECTION.
  16. Limitation of Liability.
  IN NO EVENT UNLESS REQUIRED BY APPLICABLE LAW OR AGREED TO IN WRITING
WILL ANY COPYRIGHT HOLDER, OR ANY OTHER PARTY WHO MODIFIES AND/OR CONVEYS
THE PROGRAM AS PERMITTED ABOVE, BE LIABLE TO YOU FOR DAMAGES, INCLUDING ANY
GENERAL, SPECIAL, INCIDENTAL OR CONSEQUENTIAL DAMAGES ARISING OUT OF THE
USE OR INABILITY TO USE THE PROGRAM (INCLUDING BUT NOT LIMITED TO LOSS OF
DATA OR DATA BEING RENDERED INACCURATE OR LOSSES SUSTAINED BY YOU OR THIRD
PARTIES OR A FAILURE OF THE PROGRAM TO OPERATE WITH ANY OTHER PROGRAMS),
EVEN IF SUCH HOLDER OR OTHER PARTY HAS BEEN ADVISED OF THE POSSIBILITY OF
SUCH DAMAGES.
  17. Interpretation of Sections 15 and 16.
  If the disclaimer of warranty and limitation of liability provided
above cannot be given local legal effect according to their terms,
reviewing courts shall apply local law that most closely approximates
an absolute waiver of all civil liability in connection with the
Program, unless a warranty or assumption of liability accompanies a
copy of the Program in return for a fee.
                     END OF TERMS AND CONDITIONS
            How to Apply These Terms to Your New Programs
  If you develop a new program, and you want it to be of the greatest
possible use to the public, the best way to achieve this is to make it
free software which everyone can redistribute and change under these terms.
  To do so, attach the following notices to the program.  It is safest
to attach them to the start of each source file to most effectively
state the exclusion of warranty; and each file should have at least
the "copyright" line and a pointer to where the full notice is found.
    <one line to give the program’s name and a brief idea of what it does.>
    Copyright (C) <year>  <name of author>
    This program is free software: you can redistribute it and/or modify
    it under the terms of the GNU General Public License as published by
    the Free Software Foundation, either version 3 of the License, or
    (at your option) any later version.
    This program is distributed in the hope that it will be useful,
    but WITHOUT ANY WARRANTY; without even the implied warranty of
    MERCHANTABILITY or FITNESS FOR A PARTICULAR PURPOSE.  See the
    GNU General Public License for more details.
    You should have received a copy of the GNU General Public License
    along with this program.  If not, see <http://www.gnu.org/licenses/>.
Also add information on how to contact you by electronic and paper mail.
  If the program does terminal interaction, make it output a short
notice like this when it starts in an interactive mode:
    <program>  Copyright (C) <year>  <name of author>
    This program comes with ABSOLUTELY NO WARRANTY; for details type ‘show w’.
    This is free software, and you are welcome to redistribute it
    under certain conditions; type ‘show c’ for details.
The hypothetical commands ‘show w’ and ‘show c’ should show the appropriate
parts of the General Public License.  Of course, your program’s commands
might be different; for a GUI interface, you would use an "about box".
  You should also get your employer (if you work as a programmer) or school,
if any, to sign a "copyright disclaimer" for the program, if necessary.
For more information on this, and how to apply and follow the GNU GPL, see
<http://www.gnu.org/licenses/>.
  The GNU General Public License does not permit incorporating your program
into proprietary programs.  If your program is a subroutine library, you
may consider it more useful to permit linking proprietary applications with
the library.  If this is what you want to do, use the GNU Lesser General
Public License instead of this License.  But first, please read
<http://www.gnu.org/philosophy/why-not-lgpl.html>.
```

## 30.2 MIT License for VCFtools

We have included VCFTools source code in the srcdir/vcftools directory of the Mega2 distribution. This code has been minimally modified so as to integrate it with Mega2. While the VCFtools source code as a whole is licensed under the Lesser GPL license below, these files contain the MIT license: bgzf.c and bgzf.h.

```
/* The MIT License

   Copyright (c) 2008 Broad Institute / Massachusetts Institute of Technology
                 2011, 2012 Attractive Chaos <attractor@live.co.uk>
   Permission is hereby granted, free of charge, to any person obtaining a copy
   of this software and associated documentation files (the "Software"), to deal
   in the Software without restriction, including without limitation the rights
   to use, copy, modify, merge, publish, distribute, sublicense, and/or sell
   copies of the Software, and to permit persons to whom the Software is
   furnished to do so, subject to the following conditions:

   The above copyright notice and this permission notice shall be included in
   all copies or substantial portions of the Software.

   THE SOFTWARE IS PROVIDED "AS IS", WITHOUT WARRANTY OF ANY KIND, EXPRESS OR
   IMPLIED, INCLUDING BUT NOT LIMITED TO THE WARRANTIES OF MERCHANTABILITY,
   FITNESS FOR A PARTICULAR PURPOSE AND NONINFRINGEMENT. IN NO EVENT SHALL THE
   AUTHORS OR COPYRIGHT HOLDERS BE LIABLE FOR ANY CLAIM, DAMAGES OR OTHER
   LIABILITY, WHETHER IN AN ACTION OF CONTRACT, TORT OR OTHERWISE, ARISING FROM,
   OUT OF OR IN CONNECTION WITH THE SOFTWARE OR THE USE OR OTHER DEALINGS IN
   THE SOFTWARE.
*/
```

## 30.3 License for ZLIB

For ease of compilation, we have included zlib source code in the srcdir/zlib-1.2.8 directory of the Mega2 distribution. This code has been minimally modified so as to integrate it with Mega2. The zlib home page is http://zlib.net/. It contains useful information including the license reproduced below.

```
/* zlib.h — interface of the ’zlib’ general purpose compression library
  version 1.2.8, April 28th, 2013

  Copyright (C) 1995-2013 Jean-loup Gailly and Mark Adler

  This software is provided ’as-is’, without any express or implied
  warranty.  In no event will the authors be held liable for any damages
  arising from the use of this software.

  Permission is granted to anyone to use this software for any purpose,
  including commercial applications, and to alter it and redistribute it
  freely, subject to the following restrictions:

  1. The origin of this software must not be misrepresented; you must not
     claim that you wrote the original software. If you use this software
     in a product, an acknowledgment in the product documentation would be
     appreciated but is not required.
  2. Altered source versions must be plainly marked as such, and must not be
     misrepresented as being the original software.
  3. This notice may not be removed or altered from any source distribution.

  Jean-loup Gailly        Mark Adler
  jloup@gzip.org          madler@alumni.caltech.edu

*/
```

## 30.4 GNU Lesser General Public License Version 3 for VCFtools

We have included VCFTools source code in the srcdir/vcftools directory of the Mega2 distribution. This code has been minimally modified so as to integrate it with Mega2. While the VCFtools source code as a whole is licensed under the Lesser GPL license, two of files contain the MIT license, listed above.

GNU LESSER GENERAL PUBLIC LICENSE Version 3, 29 June 2007

Copyright (C) 2007 Free Software Foundation, Inc. <http://fsf.org/> Everyone is permitted to copy and distribute verbatim copies of this license document, but changing it is not allowed.

This version of the GNU Lesser General Public License incorporates the terms and conditions of version 3 of the GNU General Public License, supplemented by the additional permissions listed below.

0. Additional Definitions.

As used herein, "this License" refers to version 3 of the GNU Lesser General Public License, and the "GNU GPL" refers to version 3 of the GNU General Public License.

"The Library" refers to a covered work governed by this License, other than an Application or a Combined Work as defined below.

An "Application" is any work that makes use of an interface provided by the Library, but which is not otherwise based on the Library. Defining a subclass of a class defined by the Library is deemed a mode of using an interface provided by the Library.

A "Combined Work" is a work produced by combining or linking an Application with the Library. The particular version of the Library with which the Combined Work was made is also called the "Linked Version".

The "Minimal Corresponding Source" for a Combined Work means the Corresponding Source for the Combined Work, excluding any source code for portions of the Combined Work that, considered in isolation, are based on the Application, and not on the Linked Version.

The "Corresponding Application Code" for a Combined Work means the object code and/or source code for the Application, including any data and utility programs needed for reproducing the Combined Work from the Application, but excluding the System Libraries of the Combined Work.

1. Exception to Section 3 of the GNU GPL.

You may convey a covered work under sections 3 and 4 of this License without being bound by section 3 of the GNU GPL.

2. Conveying Modified Versions.

If you modify a copy of the Library, and, in your modifications, a facility refers to a function or data to be supplied by an Application that uses the facility (other than as an argument passed when the facility is invoked), then you may convey a copy of the modified version:

a) under this License, provided that you make a good faith effort to ensure that, in the event an Application does not supply the function or data, the facility still operates, and performs whatever part of its purpose remains meaningful, or

b) under the GNU GPL, with none of the additional permissions of this License applicable to that copy.

3. Object Code Incorporating Material from Library Header Files.

The object code form of an Application may incorporate material from a header file that is part of the Library. You may convey such object code under terms of your choice, provided that, if the incorporated material is not limited to numerical parameters, data structure layouts and accessors, or small macros, inline functions and templates (ten or fewer lines in length), you do both of the following:

a) Give prominent notice with each copy of the object code that the Library is used in it and that the Library and its use are covered by this License.

b) Accompany the object code with a copy of the GNU GPL and this license document.

4. Combined Works.

You may convey a Combined Work under terms of your choice that, taken together, effectively do not restrict modification of the portions of the Library contained in the Combined Work and reverse engineering for debugging such modifications, if you also do each of the following:

a) Give prominent notice with each copy of the Combined Work that the Library is used in it and that the Library and its use are covered by this License.

b) Accompany the Combined Work with a copy of the GNU GPL and this license document.

c) For a Combined Work that displays copyright notices during execution, include the copyright notice for the Library among these notices, as well as a reference directing the user to the copies of the GNU GPL and this license document.

d) Do one of the following:

0) Convey the Minimal Corresponding Source under the terms of this License, and the Corresponding Application Code in a form suitable for, and under terms that permit, the user to recombine or relink the Application with a modified version of the Linked Version to produce a modified Combined Work, in the manner specified by section 6 of the GNU GPL for conveying Corresponding Source.

1) Use a suitable shared library mechanism for linking with the Library. A suitable mechanism is one that (a) uses at run time a copy of the Library already present on the user’s computer system, and (b) will operate properly with a modified version of the Library that is interface-compatible with the Linked Version.

e) Provide Installation Information, but only if you would otherwise be required to provide such information under section 6 of the GNU GPL, and only to the extent that such information is necessary to install and execute a modified version of the Combined Work produced by recombining or relinking the Application with a modified version of the Linked Version. (If you use option 4d0, the Installation Information must accompany the Minimal Corresponding Source and Corresponding Application Code. If you use option 4d1, you must provide the Installation Information in the manner specified by section 6 of the GNU GPL for conveying Corresponding Source.)

5. Combined Libraries.

You may place library facilities that are a work based on the Library side by side in a single library together with other library facilities that are not Applications and are not covered by this License, and convey such a combined library under terms of your choice, if you do both of the following:

a) Accompany the combined library with a copy of the same work based on the Library, uncombined with any other library facilities, conveyed under the terms of this License.

b) Give prominent notice with the combined library that part of it is a work based on the Library, and explaining where to find the accompanying uncombined form of the same work.

6. Revised Versions of the GNU Lesser General Public License.

The Free Software Foundation may publish revised and/or new versions of the GNU Lesser General Public License from time to time. Such new versions will be similar in spirit to the present version, but may differ in detail to address new problems or concerns.

Each version is given a distinguishing version number. If the Library as you received it specifies that a certain numbered version of the GNU Lesser General Public License "or any later version" applies to it, you have the option of following the terms and conditions either of that published version or of any later version published by the Free Software Foundation. If the Library as you received it does not specify a version number of the GNU Lesser General Public License, you may choose any version of the GNU Lesser General Public License ever published by the Free Software Foundation.

If the Library as you received it specifies that a proxy can decide whether future versions of the GNU Lesser General Public License shall apply, that proxy’s public statement of acceptance of any version is permanent authorization for you to choose that version for the Library.

# 31 PDF documentation

**Note:** This documentation is available in PDF form from https://watson.hgen.pitt.edu/docs/mega2\_html/Mega2\_Documentation.pdf and it is also included in the mega2\_html folder of the Mega2 distribution.

# 32 Grant Acknowledgments

Mega2 is currently supported by the University of Pittsburgh and the following NIH grant: R01GM076667 (Weeks, Daniel E). Prior work on Mega2 was partly supported by R01AG16989 (D.E.W.), R01HG00932 (D.E.W.), and R01AG16992 (to Jeffrey R. O’Connell). In addition, parts of Mega2 were inspired by data generated under NIH grant R01DK55406 to Ranjan Deka.

# 33 References

Almasy L, Blangero J (1998) Multipoint quantitative-trait linkage analysis in general pedigrees. Am J Hum Genet 62:1198-211

Blangero J, Almasy L (1997) Multipoint oligogenic linkage analysis of quantitative traits. Genet Epidemiol 14:959-964

Boehnke M, Cox NJ (1997) Accurate inference of relationships in sib-pair linkage studies. Am J Hum Genet 61:423-9

Guo SW, Thompson EA (1992) Performing the exact test of Hardy-Weinberg proportion for multiple alleles. Biometrics 48:361-72

Hinds D, Risch N (1996) The ASPEX package: affected sib-pair mapping. ftp://lahmed.stanford.edu/pub/aspex.

Holmans P (1993) Asymptotic properties of affected-sib-pair linkage analysis. Am J Hum Genet 52:362-374

Kong A, Cox NJ (1997) Allele-sharing models: LOD scores and accurate linkage tests. Am J Hum Genet 61:1179-88

Kruglyak L, Daly MJ, Reeve-Daly MP, Lander ES (1996) Parametric and nonparametric linkage analysis: a unified multipoint approach. Am J Hum Genet 58:1347-1363

Lange K, Weeks D, Boehnke M (1988) Programs for pedigree analysis: MENDEL, FISHER, and dGENE. Genet Epidemiol 5:471-472

Lathrop GM, Lalouel J-M (1984) Easy calculations of lod scores and genetic risks on small computers. Am J Hum Genet 36:460-465

Lathrop GM, Lalouel JM (1988) Efficient computations in multilocus linkage analysis. Am J Hum Genet 42:498-505

Lathrop GM, Lalouel JM, White RL (1986) Construction of human linkage maps: likelihood calculations for multilocus analysis. Genet Epidemiol 3:39-52

Lazzeroni LC, Lange K (1997) Markov chains for Monte Carlo tests of genetic equilibrium in multidimensional contingency tables. Annals of Statistics 25:138-168

Morris AP, Curnow RN, Whittaker JC (1997) Randomization tests of disease-marker associations. Ann Hum Genet 61:49-60

O’Connell JR, Weeks DE (1995) The VITESSE algorithm for rapid exact multilocus linkage analysis via genotype set-recoding and fuzzy inheritance. Nat Genet 11:402-408

Ott J (1989) Computer-simulation methods in human linkage analysis. Proc Natl Acad Sci USA 86:4175-4178

Sobel E, Lange K (1996) Descent graphs in pedigree analysis: Applications to haplotyping, location scores, and marker-sharing statistics. Am J Hum Genet 58:1323-1337

Terwilliger JD (1996) The ANALYZE package. ftp://linkage.cpmc.columbia.edu.

Terwilliger JD, Ott J (1994) Handbook of human genetic linkage. Johns Hopkins University Press, Baltimore

Terwilliger JD, Speer M, Ott J (1993) Chromosome-based method for rapid computer simulation in human genetic linkage analysis. Genet Epidemiol 10:217-224

Weeks DE, Lange K (1988) The affected-pedigree-member method of linkage analysis. Am J Hum Genet 42:315-326

Weeks DE, Ott J, Lathrop GM (1990) SLINK: a general simulation program for linkage analysis. Am J Hum Genet 47:A204

---

Document generated by eLyXer 1.2.5 (2013-03-10) on 2014-10-17T18:01:28.116336
