# Supplementary material for: Mega2: validated data-reformatting for linkage and association analyses
Source: Source Code Biol Med. 2014 Dec 5;9:26. doi: 10.1186/s13029-014-0026-y (PMC4269913; doi:10.1186/s13029-014-0026-y)
Supplement: Additional file 1: — A zipped archive containing the Mega2 version 4.7.1 distribution package; both source and binary executables are included. [file 13029_2014_26_MOESM1_ESM.zip › mega2_v4.7.1_src/mega2_html/mega2_toc.html]

xml version="1.0" encoding="UTF-8"?


Converted document


1: Introduction

1.1: Graphical overview of Mega2

1.2: Supported formats

1.2.1: Input formats

1.2.2: Output formats

2: Download Mega2

3: Recent improvements and changes

3.1: Enhancements in Mega2 version 4.7.1

3.2: Enhancements in Mega2 version 4.7.0

3.3: Enhancements in Mega2 version 4.6.2

3.4: Enhancements in Mega2 version 4.6.1

4: Quick start - get Mega2 running in minutes

4.1: The fast, easy way to set up your files for Mega2

4.2: Tips on more complex data

4.3: Word of caution regarding input file names

5: Citing Mega2

6: Support, bug reports, and feedback

6.1: Mega2 feedback

6.2: Mega2 Google Group

6.3: Bug reports

7: Contact information

8: Installation

8.1: Download Instructions

8.1.1: Mega2 Bitbucket repository

8.2: Prerequisites for Mega2

8.2.1: R

8.2.2: R Libraries

8.3: Installing Mega2 from a Binary Package

8.3.1: Contents of a binary package

8.3.2: Mega2 Binaries

8.3.3: Installing Mega2

8.4: Compiling and Installing Mega2 from Source

8.5: Running Mega2 on Your Data

8.6: Mega2 Documentation

8.7: License Agreement

8.8: Feedback and Bug Reports

8.9: Macintosh-specific installation issues

8.9.1: Introduction to Command line programs for Mac users

8.9.2: Xcode compilation environment

8.10: Unix-specific installation issues

8.11: Windows Cygwin installation

8.11.1: Cygwin POSIX windows environment

8.11.2: R installation:

8.11.3: Mega2 Download instructions for Windows

8.12: Windows Mingw installation

8.12.1: Mingw POSIX windows environment

8.13: Native Windows installation

8.13.1: Native Windows environment

9: Input file formats

9.1: Mega2 input file formats

9.1.1: Mega2 Names file

9.1.2: Mega2 Pedigree file

9.1.3: Mega2 Map file

9.1.4: Omit file

9.1.5: Frequency file

9.1.6: Penetrance file

9.2: PLINK input file formats

9.2.1: PLINK PED input format

9.2.2: PLINK binary PED input format

9.3: LINKAGE input file formats

9.3.1: LINKAGE locus file

9.3.2: LINKAGE pedigree file

9.3.3: Handling of loops inside pedigrees

9.3.4: Simple Mega2 Map file

9.3.5: Specifying sex-specific maps

9.3.6: Omit file [Optional]

9.3.7: Header-less Names file

9.4: Variant Call Format (VCF, BCF, compressed VCF) input file formats

9.4.1: Variant Call File

9.4.2: PLINK family File

9.4.3: Augmented PLINK Phenotype File

9.4.4: Map File (Optional)

9.4.5: Omit File (Optional)

9.4.6: Frequency File (Optional)

9.4.7: Penetrance File (Optional)

10: Genetic Map Interpolator (GMI)

11: Converting to Mega2’s format

11.1: Pedigree file conversion

11.2: Locus file conversion

11.3: Map file conversion

12: The input menu

12.1: Input formats: Mega2 and Linkage

12.2: Common PLINK menu items

12.3: Input format: PLINK binary PED

12.4: Input format: PLINK ped

12.5: Common Variant Call Format menu items

12.6: Input format: Variant Call Format files

12.7: Input menu items: Omit, frequency and penetrance data files (optional):

12.8: Input menu item: Output Directory:

12.9: Input menu item: Simulating genotyping errors:

Subsection: Genotyping error simulation

Subsection: Parameter selection menu

Subsection: Error simulation output files

12.10: Input menu item: Untyped pedigree exclusion option:

12.11: Input menu item: Upper limit for squared deviation between input and observed allele frequencies

12.12: Input menu item: Unknown allele and affection definition

12.13: Input menu item: Maximum number of alleles per marker

12.14: Errors in input data

12.14.1: Problems with locus data

12.14.2: Problems with pedigree data.

12.14.3: Genotype reset menu

13: The analysis menu

13.1: Create SimWalk2 format files

13.2: Convert to Vintage Mendel format

13.3: Convert to ASPEX format

13.4: Convert to GeneHunter-Plus format

13.5: Convert to GeneHunter format

13.6: Convert to APM format [DISABLED]

13.7: Convert to APM MULT multiple locus format [DISABLED]

13.8: Create nuclear families

13.9: Convert to SLINK format

13.10: Convert to SPLINK format

13.11: Set up for homogeneity analyses

13.12: Convert to SIMULATE format

13.13: Create summary files

13.14: Convert to Old SAGE format

13.15: Set up for TDTMax analyses[DISABLED]

13.16: Convert to SOLAR format

13.17: Convert to Vitesse format

13.18: Convert to Linkage format

13.19: Test loci for Hardy-Weinberg Equilibrium

13.20: Convert to Allegro format

13.21: Convert to MLBQTL format

13.22: Convert to SAGE format

13.23: Convert to pre-makeped format

13.24: Setup for Merlin-SimWalk2 combined analysis

13.25: Convert to PREST format

13.26: Convert to PAP format

13.27: Convert to Merlin format

13.28: Convert to LOKI format

13.29: Convert to Mendel format

13.30: Convert to SUP format

13.31: Convert to PLINK format

13.32: Convert to CRANEFOOT format

13.33: Convert to Mega2 format

13.34: Convert to IQLS/Idcoefs format

13.35: Convert to FBAT format

13.36: Convert to Morgan format

13.37: Convert to Beagle format

13.38: Convert to Eigenstrat format

13.39: Convert to Structure format

13.40: Convert to PSEQ (PLINK/SEQ) format

14: The Missing Value Menu

15: The allele frequency menu

15.1: The recoding process

15.2: The recode log file

15.3: Penetrances for affection status loci

16: The locus reordering menu

16.1: Locus reordering option 1) Select all loci in map order on chromosome.

16.2: Locus reordering option 2) Select by locus number.

16.3: Locus reordering option 3) Select marker loci on multiple chromosomes.

17: Map Selection Menus

17.1: The Genetic Map Selection Menu

17.2: The Physical Map Selection Menu

18: The trait selection menu

18.1: Select multiple trait loci to loop across.

18.2: Use trait loci in specified order.

18.3: List of selected trait loci

18.4: Selection of covariates

18.5: More details on trait selection.

18.6: Affection status labels

19: Creating plots using Mega2

19.1: Statistics selection menu

19.2: R-plot parameters menu

19.3: General usage of nplplot and nplplot.multi

20: Custom tracks for the UCSC Browser

21: Additional Mega2 Output Files

21.1: Summary file directory

21.2: MEGA2.LOG

21.3: MEGA2.ERR

21.4: MEGA2.BATCH

21.5: MEGA2.KEYS

21.6: MEGA2.SIM

21.7: MEGA2.RECODE

21.8: MEGA2run.html and related files

22: Mega2 command-line arguments

22.1: Species options

22.2: Input format options

22.3: Missing value options

22.4: General options

22.5: Obsolete options

22.6: *Other arguments*

23: Running Mega2 in Batch mode

23.1: Overview

23.2: Using the *--*nosave option

23.3: Batch file format

23.4: Major classes of batch file options

23.5: Details on batch file items

24: Hints and troubleshooting

24.1: Hints on loci reordering

24.2: Problems commonly encountered with input files:

24.3: Pedigree file reading problem

24.4: Mega2 hangs while reading in input files

24.5: DOS format file related errors

24.5.1: Error messages

24.6: Non-DOS related problems

25: Detailed information on analysis options

25.1: Create SimWalk2 format files

25.2: Convert to MENDEL format

25.3: Convert to ASPEX format

25.4: Convert to GeneHunter-Plus format

25.5: Convert to GeneHunter format

25.6: Convert to APM format

25.7: Convert to APM MULT multiple locus format

25.8: Create nuclear families

25.9: Convert to SLINK format

25.10: Convert to SPLINK format

25.11: Set up for homogeneity analyses

25.12: Convert to SIMULATE format

25.13: Create summary files

25.14: Convert to SAGE format

25.15: Set up for TDTMax analyses

25.16: Convert to SOLAR format

25.17: Convert to Vitesse format

25.18: Convert to Linkage format

25.19: Test loci for Hardy-Weinberg Equilibrium

25.20: Convert to Allegro format

25.21: Convert to MLBQTL format

25.22: Convert to S.A.G.E. 4.0 format

25.23: Convert to pre-makeped format

25.24: Convert to Merlin-SimWalk2 format

25.25: Convert to PREST format

25.26: Convert to PAP format

25.27: Convert to Merlin format

25.28: Convert to Loki format

25.29: Convert to Mendel format

25.30: Convert to SUP format

25.31: Convert to PLINK format

25.32: Convert to Cranefoot format

25.33: Convert to Mega2 format

25.34: Convert to IQLS/Idcoefs format

25.35: Convert to FBAT format

25.36: Convert to PANGAEA MORGAN format

25.37: Convert to Beagle format

25.38: Convert to Eigenstrat format

25.39: Convert to Structure format

25.40: Convert to PSEQ (PLINK/SEQ) format

26: Utilities included with Mega2

26.1: Converting linkage format files to Mega2 format - l2a.py

26.2: Map making utilities

26.3: Creating a Mega2 omit file

26.4: Scripts to generate formatted output for Hardy-Weinberg test:

27: List of third-party applications used by Mega2

27.1: R statistical package and its libraries

27.1.1: R Installation

27.2: Python

27.2.1: Python Installation

27.3: Perl

27.3.1: Perl Installation

27.4: Awk

27.4.1: Awk Installation

27.5: C-shell

28: Changes made to Mega2

28.1: Recent releases

28.2: Changes from Version 4.6.2 to Version 4.7.1 (Released Oct 16, 2014)

28.3: Changes from Version 4.6.2 to Version 4.7.0 (Released May 15, 2014)

28.4: Changes from Version 4.6.1 to Version 4.6.2 (Released Feb 28, 2014)

28.5: Changes from Version 4.6.0 to Version 4.6.1 (Released Oct 21st, 2013)

28.6: Changes from Version 4.5.9 to Version 4.6.0 (Released Sept 6th, 2013)

28.7: Changes from Version 4.5.8 to Version 4.5.9 (Released July 5th, 2013)

28.8: Changes from Version 4.5.7 to Version 4.5.8 (Released June 6th, 2013)

28.9: Changes from Version 4.5.6 to Version 4.5.7 (Released January 11th, 2013)

28.10: Changes from Version 4.5.5 to Version 4.5.6 (Released July 6th, 2012)

28.11: Changes from Version 4.5.4 to Version 4.5.5 (Released June 15th, 2012)

28.12: Changes from Version 4.5.3 to Version 4.5.4 (Released July 26th, 2011)

28.13: Changes from Version 4.0 R5.2 Beta - Version 4.5.3 (Released June 15th, 2011)

Subsection: Changes from Ver 4.0 R5.1 - Ver 4.0 R5.2 Beta

Subsection: Changes from Ver 4.0 R5.0 - Ver 4.0 R5.1 (Released June 15th, 2010)

Subsection: Changes from Ver 4.0 R4.0 - Ver 4.0 R5.0 (Released Dec 31st, 2009)

Subsection: Changes from Ver 4.0 R3.1 - Ver 4.0 R4.0 (Released October 4th, 2009)

Subsection: Changes from Ver 4.0 R3 - Ver 4.0 R3.1 (Released July 12, 2009)

Subsection: Changes from Ver 4.0 R2 - Ver 4.0 R3 (Released Jun 15, 2009)

Subsection: Changes from Ver 4.0 R1 - Ver 4.0 R2 (Released April 15, 2009)

Subsection: Changes from Ver 4.0 - Ver 4.0 R1 (Released Jun 13, 2008)

Subsection: Changes from Ver 4.0 Beta R1 - Ver 4.0 (Released March 31, 2008)

Subsection: Changes from Ver 4.0 Beta- Ver 4.0 Beta R1 (Released August 7, 2007)

Subsection: Changes from Ver 3.0 R11- Ver 3.0 R12 and Ver 4.0 Beta (Released June 14, 2007)

Subsection: Changes from Ver 3.0 R10- Ver 3.0 R11 (Released May 17, 2007)

Subsection: Changes from Ver 3.0 R9- Ver 3.0 R10 (Released February 1, 2007)

Subsection: Very old versions

Subsection: Changes from Ver 3.0 R8- Ver 3.0 R9 (Released July 14, 2006)

Subsection: Changes from Ver 3.0 R7- Ver 3.0 R8 (Released June 19, 2006)

Subsection: Changes from Ver 3.0 R5,R6 - Ver 3.0 R7 (Released June 15, 2006)

Subsection: Changes from Ver 3.0 R4 - Ver 3.0 R5 (Released Feb 2, 2006)

Subsection: Changes from Ver 3.0 R3 - Ver 3.0 R4 (Released June 10, 2005)

Subsection: Changes from Ver 3.0 R2 - Ver 3.0 R3 (Released November 29, 2004)

Subsection: Changes from Ver 3.0 R1 - Ver 3.0 R2 (Released September 30, 2004)

Subsection: Changes from Ver 3.0 - Ver 3.0 R1 (Released August 15, 2004)

Subsection: Changes from Ver 2.5 R4 - Ver 3.0 (Released June 15, 2004)

Subsection: Changes from Ver 2.5 R3 - Ver 2.5 R4 (Released April 22, 2004)

Subsection: Changes from Ver 2.5 R2 - Ver 2.5 R3 (Released April 15, 2004)

Subsection: Changes from Ver 2.5 R1 - Ver 2.5 R2 (Released August 8, 2003)

Subsection: Changes from Ver 2.5 - Ver 2.5 R1 (Released July 5th, 2003)

Subsection: Changes from Ver 2.3 R4 - Ver 2.5 (Released June 3, 2003)

Subsection: Changes from Ver 2.3 R3 - Ver 2.3 R4 (Released Feb 7, 2003)

Subsection: Changes from Ver 2.3 R2 - Ver 2.3 R3 (Released Dec 13, 2002)

Subsection: Changes from Ver 2.3 - Ver 2.3 R2 (Released July 20 2002)

Subsection: Changes from Ver 2.2 R3 - Ver 2.3 (Released June 14 2002)

Subsection: Changes from Ver 2.2 R2 - Ver 2.2 R3 (Released January 23rd, 2002)

Subsection: Changes from version 2.2 - version 2.2 R2 (Released 28th June 2001)

Subsection: Changes from version 2.1 beta R3 - version 2.2 (Released 15 Jun 2001)

Subsection: Changes from version 2.1 beta R2 - version 2.1 beta R3 (Released 30 Mar 2001)

Subsection: Changes from version 2.1 beta - version 2.1 beta R2 (Released 16 Mar 2001)

Subsection: Changes from version 2.05 to version 2.1 beta (Released 16 Feb 2001)

29: List of fixed bugs

Subsection: Bugs in Mega2 4.0 R5.2 Beta

Subsection: Bugs in Mega2 4 R5.1

Subsection: Bugs in Mega2 4 R5.0

Subsection: Bugs in Mega2 4 R4.0

Subsection: Bugs in Mega2 4 R3.1

Subsection: Bugs in Mega2 4 R3

Subsection: Bugs in Mega2 4.0 R1

Subsection: Bugs in Mega2 4.0

Subsection: Bugs in Mega2 4.0 beta R1

Subsection: Bugs in Mega2 4.0 beta

Subsection: Bugs in Mega2 3.0 R11

Subsection: Bugs in Mega2 3.0 R10

Subsection: Bugs in Mega2 3.0 R9

Subsection: Bugs in Mega2 3.0 R8

Subsection: Bugs in Mega2 3.0 R7

Subsection: Bugs in Mega2 3.0 R5, R6

Subsection: Bugs in Mega2 3.0 R4

Subsection: Bugs in Mega2 3.0 R3

Subsection: Bugs in Mega2 3.0 R2

Subsection: Bugs in Mega2 3.0 R1

Subsection: Bugs in Mega2 3.0

Subsection: Bugs in Mega2 2.5 R4

Subsection: Bugs in Mega2 2.5 R3

Subsection: Bugs in Mega2 2.5 R2

Subsection: Bugs in Mega2 2.5 R1

Subsection: Bugs in Mega2 2.5

Subsection: Bugs in Mega2 2.3 R4

Subsection: Bugs in Mega2 2.3 R3

Subsection: Bugs in Mega2 2.3 R2

Subsection: Bugs in Mega2 2.3 and Mega2 2.3 R1

Subsection: Bugs in Mega2 2.2 R3

Subsection: Bugs in Mega2 2.2 R2

Subsection: Bugs in Mega2 2.2

Subsection: Bugs in Mega2 2.1 beta

30: License agreements

30.1: GNU General Public License Version 3 for Mega2

30.2: MIT License for VCFtools

30.3: License for ZLIB

30.4: GNU Lesser General Public License Version 3 for VCFtools

31: PDF documentation

32: Grant Acknowledgments

33: References

---

Document generated by eLyXer 1.2.5 (2013-03-10) on 2014-10-17T18:01:30.350654
