# Supplementary material for: Mega2: validated data-reformatting for linkage and association analyses
Source: Source Code Biol Med. 2014 Dec 5;9:26. doi: 10.1186/s13029-014-0026-y (PMC4269913; doi:10.1186/s13029-014-0026-y)
Supplement: Additional file 1: — A zipped archive containing the Mega2 version 4.7.1 distribution package; both source and binary executables are included. [file 13029_2014_26_MOESM1_ESM.zip › mega2_v4.7.1_src/srcdir/zlib-1.2.8/examples/zlib_how.html]

zlib Usage Example


## zlib Usage Example

We often get questions about how the deflate() and inflate() functions should be used.
Users wonder when they should provide more input, when they should use more output,
what to do with a Z\_BUF\_ERROR, how to make sure the process terminates properly, and
so on. So for those who have read zlib.h (a few times), and
would like further edification, below is an annotated example in C of simple routines to compress and decompress
from an input file to an output file using deflate() and inflate() respectively. The
annotations are interspersed between lines of the code. So please read between the lines.
We hope this helps explain some of the intricacies of *zlib*.

Without further adieu, here is the program zpipe.c:

```
/* zpipe.c: example of proper use of zlib's inflate() and deflate()
   Not copyrighted -- provided to the public domain
   Version 1.4  11 December 2005  Mark Adler */

/* Version history:
   1.0  30 Oct 2004  First version
   1.1   8 Nov 2004  Add void casting for unused return values
                     Use switch statement for inflate() return values
   1.2   9 Nov 2004  Add assertions to document zlib guarantees
   1.3   6 Apr 2005  Remove incorrect assertion in inf()
   1.4  11 Dec 2005  Add hack to avoid MSDOS end-of-line conversions
                     Avoid some compiler warnings for input and output buffers
 */
```

We now include the header files for the required definitions. From
stdio.h we use fopen(), fread(), fwrite(),
feof(), ferror(), and fclose() for file i/o, and
fputs() for error messages. From string.h we use
strcmp() for command line argument processing.
From assert.h we use the assert() macro.
From zlib.h
we use the basic compression functions deflateInit(),
deflate(), and deflateEnd(), and the basic decompression
functions inflateInit(), inflate(), and
inflateEnd().

```
#include <stdio.h>
#include <string.h>
#include <assert.h>
#include "zlib.h"
```

This is an ugly hack required to avoid corruption of the input and output data on
Windows/MS-DOS systems. Without this, those systems would assume that the input and output
files are text, and try to convert the end-of-line characters from one standard to
another. That would corrupt binary data, and in particular would render the compressed data unusable.
This sets the input and output to binary which suppresses the end-of-line conversions.
SET\_BINARY\_MODE() will be used later on stdin and stdout, at the beginning of main().

```
#if defined(MSDOS) || defined(OS2) || defined(WIN32) || defined(__CYGWIN__)
#  include <fcntl.h>
#  include <io.h>
#  define SET_BINARY_MODE(file) setmode(fileno(file), O_BINARY)
#else
#  define SET_BINARY_MODE(file)
#endif
```

CHUNK is simply the buffer size for feeding data to and pulling data
from the *zlib* routines. Larger buffer sizes would be more efficient,
especially for inflate(). If the memory is available, buffers sizes
on the order of 128K or 256K bytes should be used.

```
#define CHUNK 16384
```

The def() routine compresses data from an input file to an output file. The output data
will be in the *zlib* format, which is different from the *gzip* or *zip*
formats. The *zlib* format has a very small header of only two bytes to identify it as
a *zlib* stream and to provide decoding information, and a four-byte trailer with a fast
check value to verify the integrity of the uncompressed data after decoding.

```
/* Compress from file source to file dest until EOF on source.
   def() returns Z_OK on success, Z_MEM_ERROR if memory could not be
   allocated for processing, Z_STREAM_ERROR if an invalid compression
   level is supplied, Z_VERSION_ERROR if the version of zlib.h and the
   version of the library linked do not match, or Z_ERRNO if there is
   an error reading or writing the files. */
int def(FILE *source, FILE *dest, int level)
{
```

Here are the local variables for def(). ret will be used for *zlib*
return codes. flush will keep track of the current flushing state for deflate(),
which is either no flushing, or flush to completion after the end of the input file is reached.
have is the amount of data returned from deflate(). The strm structure
is used to pass information to and from the *zlib* routines, and to maintain the
deflate() state. in and out are the input and output buffers for
deflate().

```
    int ret, flush;
    unsigned have;
    z_stream strm;
    unsigned char in[CHUNK];
    unsigned char out[CHUNK];
```

The first thing we do is to initialize the *zlib* state for compression using
deflateInit(). This must be done before the first use of deflate().
The zalloc, zfree, and opaque fields in the strm
structure must be initialized before calling deflateInit(). Here they are
set to the *zlib* constant Z\_NULL to request that *zlib* use
the default memory allocation routines. An application may also choose to provide
custom memory allocation routines here. deflateInit() will allocate on the
order of 256K bytes for the internal state.
(See *zlib Technical Details*.)

deflateInit() is called with a pointer to the structure to be initialized and
the compression level, which is an integer in the range of -1 to 9. Lower compression
levels result in faster execution, but less compression. Higher levels result in
greater compression, but slower execution. The *zlib* constant Z\_DEFAULT\_COMPRESSION,
equal to -1,
provides a good compromise between compression and speed and is equivalent to level 6.
Level 0 actually does no compression at all, and in fact expands the data slightly to produce
the *zlib* format (it is not a byte-for-byte copy of the input).
More advanced applications of *zlib*
may use deflateInit2() here instead. Such an application may want to reduce how
much memory will be used, at some price in compression. Or it may need to request a
*gzip* header and trailer instead of a *zlib* header and trailer, or raw
encoding with no header or trailer at all.

We must check the return value of deflateInit() against the *zlib* constant
Z\_OK to make sure that it was able to
allocate memory for the internal state, and that the provided arguments were valid.
deflateInit() will also check that the version of *zlib* that the zlib.h
file came from matches the version of *zlib* actually linked with the program. This
is especially important for environments in which *zlib* is a shared library.

Note that an application can initialize multiple, independent *zlib* streams, which can
operate in parallel. The state information maintained in the structure allows the *zlib*
routines to be reentrant.

```
    /* allocate deflate state */
    strm.zalloc = Z_NULL;
    strm.zfree = Z_NULL;
    strm.opaque = Z_NULL;
    ret = deflateInit(&strm, level);
    if (ret != Z_OK)
        return ret;
```

With the pleasantries out of the way, now we can get down to business. The outer do-loop
reads all of the input file and exits at the bottom of the loop once end-of-file is reached.
This loop contains the only call of deflate(). So we must make sure that all of the
input data has been processed and that all of the output data has been generated and consumed
before we fall out of the loop at the bottom.

```
    /* compress until end of file */
    do {
```

We start off by reading data from the input file. The number of bytes read is put directly
into avail\_in, and a pointer to those bytes is put into next\_in. We also
check to see if end-of-file on the input has been reached. If we are at the end of file, then flush is set to the
*zlib* constant Z\_FINISH, which is later passed to deflate() to
indicate that this is the last chunk of input data to compress. We need to use feof()
to check for end-of-file as opposed to seeing if fewer than CHUNK bytes have been read. The
reason is that if the input file length is an exact multiple of CHUNK, we will miss
the fact that we got to the end-of-file, and not know to tell deflate() to finish
up the compressed stream. If we are not yet at the end of the input, then the *zlib*
constant Z\_NO\_FLUSH will be passed to deflate to indicate that we are still
in the middle of the uncompressed data.

If there is an error in reading from the input file, the process is aborted with
deflateEnd() being called to free the allocated *zlib* state before returning
the error. We wouldn't want a memory leak, now would we? deflateEnd() can be called
at any time after the state has been initialized. Once that's done, deflateInit() (or
deflateInit2()) would have to be called to start a new compression process. There is
no point here in checking the deflateEnd() return code. The deallocation can't fail.

```
        strm.avail_in = fread(in, 1, CHUNK, source);
        if (ferror(source)) {
            (void)deflateEnd(&strm);
            return Z_ERRNO;
        }
        flush = feof(source) ? Z_FINISH : Z_NO_FLUSH;
        strm.next_in = in;
```

The inner do-loop passes our chunk of input data to deflate(), and then
keeps calling deflate() until it is done producing output. Once there is no more
new output, deflate() is guaranteed to have consumed all of the input, i.e.,
avail\_in will be zero.

```
        /* run deflate() on input until output buffer not full, finish
           compression if all of source has been read in */
        do {
```

Output space is provided to deflate() by setting avail\_out to the number
of available output bytes and next\_out to a pointer to that space.

```
            strm.avail_out = CHUNK;
            strm.next_out = out;
```

Now we call the compression engine itself, deflate(). It takes as many of the
avail\_in bytes at next\_in as it can process, and writes as many as
avail\_out bytes to next\_out. Those counters and pointers are then
updated past the input data consumed and the output data written. It is the amount of
output space available that may limit how much input is consumed.
Hence the inner loop to make sure that
all of the input is consumed by providing more output space each time. Since avail\_in
and next\_in are updated by deflate(), we don't have to mess with those
between deflate() calls until it's all used up.

The parameters to deflate() are a pointer to the strm structure containing
the input and output information and the internal compression engine state, and a parameter
indicating whether and how to flush data to the output. Normally deflate will consume
several K bytes of input data before producing any output (except for the header), in order
to accumulate statistics on the data for optimum compression. It will then put out a burst of
compressed data, and proceed to consume more input before the next burst. Eventually,
deflate()
must be told to terminate the stream, complete the compression with provided input data, and
write out the trailer check value. deflate() will continue to compress normally as long
as the flush parameter is Z\_NO\_FLUSH. Once the Z\_FINISH parameter is provided,
deflate() will begin to complete the compressed output stream. However depending on how
much output space is provided, deflate() may have to be called several times until it
has provided the complete compressed stream, even after it has consumed all of the input. The flush
parameter must continue to be Z\_FINISH for those subsequent calls.

There are other values of the flush parameter that are used in more advanced applications. You can
force deflate() to produce a burst of output that encodes all of the input data provided
so far, even if it wouldn't have otherwise, for example to control data latency on a link with
compressed data. You can also ask that deflate() do that as well as erase any history up to
that point so that what follows can be decompressed independently, for example for random access
applications. Both requests will degrade compression by an amount depending on how often such
requests are made.

deflate() has a return value that can indicate errors, yet we do not check it here. Why
not? Well, it turns out that deflate() can do no wrong here. Let's go through
deflate()'s return values and dispense with them one by one. The possible values are
Z\_OK, Z\_STREAM\_END, Z\_STREAM\_ERROR, or Z\_BUF\_ERROR. Z\_OK
is, well, ok. Z\_STREAM\_END is also ok and will be returned for the last call of
deflate(). This is already guaranteed by calling deflate() with Z\_FINISH
until it has no more output. Z\_STREAM\_ERROR is only possible if the stream is not
initialized properly, but we did initialize it properly. There is no harm in checking for
Z\_STREAM\_ERROR here, for example to check for the possibility that some
other part of the application inadvertently clobbered the memory containing the *zlib* state.
Z\_BUF\_ERROR will be explained further below, but
suffice it to say that this is simply an indication that deflate() could not consume
more input or produce more output. deflate() can be called again with more output space
or more available input, which it will be in this code.

```
            ret = deflate(&strm, flush);    /* no bad return value */
            assert(ret != Z_STREAM_ERROR);  /* state not clobbered */
```

Now we compute how much output deflate() provided on the last call, which is the
difference between how much space was provided before the call, and how much output space
is still available after the call. Then that data, if any, is written to the output file.
We can then reuse the output buffer for the next call of deflate(). Again if there
is a file i/o error, we call deflateEnd() before returning to avoid a memory leak.

```
            have = CHUNK - strm.avail_out;
            if (fwrite(out, 1, have, dest) != have || ferror(dest)) {
                (void)deflateEnd(&strm);
                return Z_ERRNO;
            }
```

The inner do-loop is repeated until the last deflate() call fails to fill the
provided output buffer. Then we know that deflate() has done as much as it can with
the provided input, and that all of that input has been consumed. We can then fall out of this
loop and reuse the input buffer.

The way we tell that deflate() has no more output is by seeing that it did not fill
the output buffer, leaving avail\_out greater than zero. However suppose that
deflate() has no more output, but just so happened to exactly fill the output buffer!
avail\_out is zero, and we can't tell that deflate() has done all it can.
As far as we know, deflate()
has more output for us. So we call it again. But now deflate() produces no output
at all, and avail\_out remains unchanged as CHUNK. That deflate() call
wasn't able to do anything, either consume input or produce output, and so it returns
Z\_BUF\_ERROR. (See, I told you I'd cover this later.) However this is not a problem at
all. Now we finally have the desired indication that deflate() is really done,
and so we drop out of the inner loop to provide more input to deflate().

With flush set to Z\_FINISH, this final set of deflate() calls will
complete the output stream. Once that is done, subsequent calls of deflate() would return
Z\_STREAM\_ERROR if the flush parameter is not Z\_FINISH, and do no more processing
until the state is reinitialized.

Some applications of *zlib* have two loops that call deflate()
instead of the single inner loop we have here. The first loop would call
without flushing and feed all of the data to deflate(). The second loop would call
deflate() with no more
data and the Z\_FINISH parameter to complete the process. As you can see from this
example, that can be avoided by simply keeping track of the current flush state.

```
        } while (strm.avail_out == 0);
        assert(strm.avail_in == 0);     /* all input will be used */
```

Now we check to see if we have already processed all of the input file. That information was
saved in the flush variable, so we see if that was set to Z\_FINISH. If so,
then we're done and we fall out of the outer loop. We're guaranteed to get Z\_STREAM\_END
from the last deflate() call, since we ran it until the last chunk of input was
consumed and all of the output was generated.

```
        /* done when last data in file processed */
    } while (flush != Z_FINISH);
    assert(ret == Z_STREAM_END);        /* stream will be complete */
```

The process is complete, but we still need to deallocate the state to avoid a memory leak
(or rather more like a memory hemorrhage if you didn't do this). Then
finally we can return with a happy return value.

```
    /* clean up and return */
    (void)deflateEnd(&strm);
    return Z_OK;
}
```

Now we do the same thing for decompression in the inf() routine. inf()
decompresses what is hopefully a valid *zlib* stream from the input file and writes the
uncompressed data to the output file. Much of the discussion above for def()
applies to inf() as well, so the discussion here will focus on the differences between
the two.

```
/* Decompress from file source to file dest until stream ends or EOF.
   inf() returns Z_OK on success, Z_MEM_ERROR if memory could not be
   allocated for processing, Z_DATA_ERROR if the deflate data is
   invalid or incomplete, Z_VERSION_ERROR if the version of zlib.h and
   the version of the library linked do not match, or Z_ERRNO if there
   is an error reading or writing the files. */
int inf(FILE *source, FILE *dest)
{
```

The local variables have the same functionality as they do for def(). The
only difference is that there is no flush variable, since inflate()
can tell from the *zlib* stream itself when the stream is complete.

```
    int ret;
    unsigned have;
    z_stream strm;
    unsigned char in[CHUNK];
    unsigned char out[CHUNK];
```

The initialization of the state is the same, except that there is no compression level,
of course, and two more elements of the structure are initialized. avail\_in
and next\_in must be initialized before calling inflateInit(). This
is because the application has the option to provide the start of the zlib stream in
order for inflateInit() to have access to information about the compression
method to aid in memory allocation. In the current implementation of *zlib*
(up through versions 1.2.x), the method-dependent memory allocations are deferred to the first call of
inflate() anyway. However those fields must be initialized since later versions
of *zlib* that provide more compression methods may take advantage of this interface.
In any case, no decompression is performed by inflateInit(), so the
avail\_out and next\_out fields do not need to be initialized before calling.

Here avail\_in is set to zero and next\_in is set to Z\_NULL to
indicate that no input data is being provided.

```
    /* allocate inflate state */
    strm.zalloc = Z_NULL;
    strm.zfree = Z_NULL;
    strm.opaque = Z_NULL;
    strm.avail_in = 0;
    strm.next_in = Z_NULL;
    ret = inflateInit(&strm);
    if (ret != Z_OK)
        return ret;
```

The outer do-loop decompresses input until inflate() indicates
that it has reached the end of the compressed data and has produced all of the uncompressed
output. This is in contrast to def() which processes all of the input file.
If end-of-file is reached before the compressed data self-terminates, then the compressed
data is incomplete and an error is returned.

```
    /* decompress until deflate stream ends or end of file */
    do {
```

We read input data and set the strm structure accordingly. If we've reached the
end of the input file, then we leave the outer loop and report an error, since the
compressed data is incomplete. Note that we may read more data than is eventually consumed
by inflate(), if the input file continues past the *zlib* stream.
For applications where *zlib* streams are embedded in other data, this routine would
need to be modified to return the unused data, or at least indicate how much of the input
data was not used, so the application would know where to pick up after the *zlib* stream.

```
        strm.avail_in = fread(in, 1, CHUNK, source);
        if (ferror(source)) {
            (void)inflateEnd(&strm);
            return Z_ERRNO;
        }
        if (strm.avail_in == 0)
            break;
        strm.next_in = in;
```

The inner do-loop has the same function it did in def(), which is to
keep calling inflate() until has generated all of the output it can with the
provided input.

```
        /* run inflate() on input until output buffer not full */
        do {
```

Just like in def(), the same output space is provided for each call of inflate().

```
            strm.avail_out = CHUNK;
            strm.next_out = out;
```

Now we run the decompression engine itself. There is no need to adjust the flush parameter, since
the *zlib* format is self-terminating. The main difference here is that there are
return values that we need to pay attention to. Z\_DATA\_ERROR
indicates that inflate() detected an error in the *zlib* compressed data format,
which means that either the data is not a *zlib* stream to begin with, or that the data was
corrupted somewhere along the way since it was compressed. The other error to be processed is
Z\_MEM\_ERROR, which can occur since memory allocation is deferred until inflate()
needs it, unlike deflate(), whose memory is allocated at the start by deflateInit().

Advanced applications may use
deflateSetDictionary() to prime deflate() with a set of likely data to improve the
first 32K or so of compression. This is noted in the *zlib* header, so inflate()
requests that that dictionary be provided before it can start to decompress. Without the dictionary,
correct decompression is not possible. For this routine, we have no idea what the dictionary is,
so the Z\_NEED\_DICT indication is converted to a Z\_DATA\_ERROR.

inflate() can also return Z\_STREAM\_ERROR, which should not be possible here,
but could be checked for as noted above for def(). Z\_BUF\_ERROR does not need to be
checked for here, for the same reasons noted for def(). Z\_STREAM\_END will be
checked for later.

```
            ret = inflate(&strm, Z_NO_FLUSH);
            assert(ret != Z_STREAM_ERROR);  /* state not clobbered */
            switch (ret) {
            case Z_NEED_DICT:
                ret = Z_DATA_ERROR;     /* and fall through */
            case Z_DATA_ERROR:
            case Z_MEM_ERROR:
                (void)inflateEnd(&strm);
                return ret;
            }
```

The output of inflate() is handled identically to that of deflate().

```
            have = CHUNK - strm.avail_out;
            if (fwrite(out, 1, have, dest) != have || ferror(dest)) {
                (void)inflateEnd(&strm);
                return Z_ERRNO;
            }
```

The inner do-loop ends when inflate() has no more output as indicated
by not filling the output buffer, just as for deflate(). In this case, we cannot
assert that strm.avail\_in will be zero, since the deflate stream may end before the file
does.

```
        } while (strm.avail_out == 0);
```

The outer do-loop ends when inflate() reports that it has reached the
end of the input *zlib* stream, has completed the decompression and integrity
check, and has provided all of the output. This is indicated by the inflate()
return value Z\_STREAM\_END. The inner loop is guaranteed to leave ret
equal to Z\_STREAM\_END if the last chunk of the input file read contained the end
of the *zlib* stream. So if the return value is not Z\_STREAM\_END, the
loop continues to read more input.

```
        /* done when inflate() says it's done */
    } while (ret != Z_STREAM_END);
```

At this point, decompression successfully completed, or we broke out of the loop due to no
more data being available from the input file. If the last inflate() return value
is not Z\_STREAM\_END, then the *zlib* stream was incomplete and a data error
is returned. Otherwise, we return with a happy return value. Of course, inflateEnd()
is called first to avoid a memory leak.

```
    /* clean up and return */
    (void)inflateEnd(&strm);
    return ret == Z_STREAM_END ? Z_OK : Z_DATA_ERROR;
}
```

That ends the routines that directly use *zlib*. The following routines make this
a command-line program by running data through the above routines from stdin to
stdout, and handling any errors reported by def() or inf().

zerr() is used to interpret the possible error codes from def()
and inf(), as detailed in their comments above, and print out an error message.
Note that these are only a subset of the possible return values from deflate()
and inflate().

```
/* report a zlib or i/o error */
void zerr(int ret)
{
    fputs("zpipe: ", stderr);
    switch (ret) {
    case Z_ERRNO:
        if (ferror(stdin))
            fputs("error reading stdin\n", stderr);
        if (ferror(stdout))
            fputs("error writing stdout\n", stderr);
        break;
    case Z_STREAM_ERROR:
        fputs("invalid compression level\n", stderr);
        break;
    case Z_DATA_ERROR:
        fputs("invalid or incomplete deflate data\n", stderr);
        break;
    case Z_MEM_ERROR:
        fputs("out of memory\n", stderr);
        break;
    case Z_VERSION_ERROR:
        fputs("zlib version mismatch!\n", stderr);
    }
}
```

Here is the main() routine used to test def() and inf(). The
zpipe command is simply a compression pipe from stdin to stdout, if
no arguments are given, or it is a decompression pipe if zpipe -d is used. If any other
arguments are provided, no compression or decompression is performed. Instead a usage
message is displayed. Examples are zpipe < foo.txt > foo.txt.z to compress, and
zpipe -d < foo.txt.z > foo.txt to decompress.

```
/* compress or decompress from stdin to stdout */
int main(int argc, char **argv)
{
    int ret;

    /* avoid end-of-line conversions */
    SET_BINARY_MODE(stdin);
    SET_BINARY_MODE(stdout);

    /* do compression if no arguments */
    if (argc == 1) {
        ret = def(stdin, stdout, Z_DEFAULT_COMPRESSION);
        if (ret != Z_OK)
            zerr(ret);
        return ret;
    }

    /* do decompression if -d specified */
    else if (argc == 2 && strcmp(argv[1], "-d") == 0) {
        ret = inf(stdin, stdout);
        if (ret != Z_OK)
            zerr(ret);
        return ret;
    }

    /* otherwise, report usage */
    else {
        fputs("zpipe usage: zpipe [-d] < source > dest\n", stderr);
        return 1;
    }
}
```

---

*Copyright (c) 2004, 2005 by Mark Adler  
Last modified 11 December 2005*
