# Supplementary material for: Mega2: validated data-reformatting for linkage and association analyses
Source: Source Code Biol Med. 2014 Dec 5;9:26. doi: 10.1186/s13029-014-0026-y (PMC4269913; doi:10.1186/s13029-014-0026-y)
Supplement: Additional file 1: — A zipped archive containing the Mega2 version 4.7.1 distribution package; both source and binary executables are included. [file 13029_2014_26_MOESM1_ESM.zip › mega2_v4.7.1_src/srcdir/zlib-1.2.8/zlib.3.pdf]

NAME

zlib − compression/decompression library

SYNOPSIS

[see *zlib.h* for full description]

DESCRIPTION

The *zlib* library is a general purpose data compression library. The code is thread safe, assuming that the standard library functions used are thread safe, such as memory allocation routines. It provides in-memory compression and decompression functions, including integrity checks of the uncompressed data. This version of the library supports only one compression method (deflation) but other algorithms may be added later with the same stream interface.

Compression can be done in a single step if the buffers are large enough or can be done by repeated calls of the compression function. In the latter case, the application must provide more input and/or consume the output (providing more output space) before each call.

The library also supports reading and writing files in *gzip*(1) (.gz) format with an interface similar to that of stdio.

The library does not install any signal handler. The decoder checks the consistency of the compressed data, so the library should never crash even in the case of corrupted input.

All functions of the compression library are documented in the file *zlib.h*. The distribution source includes examples of use of the library in the files *test/example.c* and *test/minigzip.c,* as well as other examples in the *examples/* directory.

Changes to this version are documented in the file *ChangeLog* that accompanies the source.

zlib is available in Java using the java.util.zip package:

http://java.sun.com/developer/technicalArticles/Programming/compression/

A Perl interface to *zlib*, written by Paul Marquess (pmqs@cpan.org), is available at CPAN (Comprehensive Perl Archive Network) sites, including:

http://search.cpan.org/˜pmqs/IO-Compress-Zlib/

A Python interface to *zlib*, written by A.M. Kuchling (amk@magnet.com), is available in Python 1.5 and later versions:

http://docs.python.org/library/zlib.html

zlib is built into *tcl:*

http://wiki.tcl.tk/4610

An experimental package to read and write files in .zip format, written on top of *zlib* by Gilles Vollant (info@winimage.com), is available at:

http://www.winimage.com/zLibDll/minizip.html and also in the *contrib/minizip* directory of the main *zlib* source distribution.

SEE ALSO

The *zlib* web site can be found at:

http://zlib.net/

The data format used by the zlib library is described by RFC (Request for Comments) 1950 to 1952 in the files:

http://tools.ietf.org/html/rfc1950 (for the zlib header and trailer format)
http://tools.ietf.org/html/rfc1951 (for the deflate compressed data format)
http://tools.ietf.org/html/rfc1952 (for the gzip header and trailer format)

Mark Nelson wrote an article about *zlib* for the Jan. 1997 issue of Dr. Dobb's Journal; a copy of the article is available at:

http://marknelson.us/1997/01/01/zlib-engine/

REPORTING PROBLEMS

Before reporting a problem, please check the *zlib* web site to verify that you have the latest version of *zlib*; otherwise, obtain the latest version and see if the problem still exists. Please read the *zlib* FAQ at:

http://zlib.net/zlib_faq.html

before asking for help. Send questions and/or comments to zlib@gzip.org, or (for the Windows DLL version) to Gilles Vollant (info@winimage.com).

AUTHORS

Version 1.2.8 Copyright (C) 1995-2013 Jean-loup Gailly (jloup@gzip.org) and Mark Adler (madler@alumni.caltech.edu).

This software is provided "as-is," without any express or implied warranty. In no event will the authors be held liable for any damages arising from the use of this software. See the distribution directory with respect to requirements governing redistribution. The deflate format used by *zlib* was defined by Phil Katz. The deflate and *zlib* specifications were written by L. Peter Deutsch. Thanks to all the people who reported problems and suggested various improvements in *zlib*; who are too numerous to cite here.

UNIX manual page by R. P. C. Rodgers, U.S. National Library of Medicine (rodgers@nlm.nih.gov).
